# NOX4 has the potential to be a biomarker associated with colon cancer ferroptosis and immune infiltration based on bioinformatics analysis

**DOI:** 10.3389/fonc.2022.968043

**Published:** 2022-09-28

**Authors:** Xiaoping Yang, Yi Yu, Zirui Wang, Pingfan Wu, Xiaolu Su, Zhiping Wu, Jianxin Gan, Dekui Zhang

**Affiliations:** ^1^ Key Laboratory of Digestive Diseases of Gansu Province, Lanzhou University Second Hospital, Lanzhou, China; ^2^ The Second Clinical Medical College, Lanzhou University, Lanzhou, China; ^3^ Department of Gastroenterology, Lanzhou University Second Hospital, Lanzhou, China; ^4^ Department of Pathology, The 940th Hospital of the Joint Logistic Support of the People’s Liberation Army, Lanzhou, China; ^5^ Department of Pathology, Lanzhou University Second Hospital, Lanzhou, China; ^6^ Department of general surgery, Lanzhou University Second Hospital, Lanzhou, China

**Keywords:** colon cancer (CC), ceRNA, NOX4, ferroptosis, immune infiltration, prognosis

## Abstract

**Background:**

Colon cancer (CC) is a common tumor, but its pathogenesis is still not well understood. Competitive endogenous RNA (ceRNA) theory, ferroptosis and tumor immune infiltration may be the mechanisms of the development of cancer. The purpose of the study is to seek genes connected with both immunity and ferroptosis, and provide important molecular basis for early noninvasive diagnosis and immunotherapy of CC.

**Methods:**

We extracted messenger RNA (mRNA), microRNA (miRNA), and long noncoding RNA (lncRNA) data of CC from The Cancer Genome Atlas database (TCGA), identified the differentially expressed mRNA (DEmRNA), miRNA (DEmiRNA) and lncRNA (DElncRNA), then constructed a ceRNA network. Venn overlap analysis was used to identify genes associated with immunity and ferroptosis in ceRNA network. The expression and prognosis of target genes were analyzed *via* Gene Expression Profiling Interactive Analysis (GEPIA) and PrognoScan database, and we analysed the related functions and signaling pathways of target genes by enrichment analysis. The correlation between target genes and tumor immune infiltrating was explored by CIBERSORT and spearman correlation analysis. Finally, the expression of target genes was detected *via* quantitative reverse transcription-PCR (qRT-PCR) in CC and normal colon tissues.

**Results:**

Results showed that there were 4 DElncRNA, 4 DEmiRNA and 126 DEmRNA in ceRNA network. NADPH oxidase 4 protein (NOX4) was a DEmRNA associated with immunity and ferroptosis in ceRNA network. NOX4 was highly expressed in CC and connected with unfavourable prognosis. NOX4 was obviously enriched in pathways connected with carcinogenesis and significantly correlated with six kinds of immune cells. Immune checkpoints and NOX4 spearman correlation analysis showed that the expression of NOX4 was positively related to programmed cell death protein 1 (PD-1)-PDCD1, programmed cell death-Ligand 1 (PD-L1)-CD274 and cytotoxic T-lymphocyte-associated protein 4 (CTLA4).

**Conclusions:**

To conclude, our study suggests that NOX4 is associated with both ferroptosis and tumor immunity, and might be a biomarker associated with the carcinogenesis, prognosis of CC and a potential target of CC immunotherapy.

## Introduction

CC is the third most common cancer worldwide. From 2014 to 2019, the incidence of CC was 36.5 percent and the death rate was 13.4 percent in the United States (1). In recent years, due to the lack of clear early symptoms and concern about the pain of colonoscopy, most CC patients are in advanced stage at the time of diagnosis and have developed distant metastases with poor prognosis (2–4). Therefore, non-invasive early diagnosis and treatment of CC are urgently needed to improve the early recognition rate and prolong the lifetime of CC patients. Now, the standard treatment for advanced CC combines neoadjuvant chemoradiotherapy, molecular-targeted therapy and immunotherapy (5–10). In recent years, the development of cancer immunotherapy is very rapid. The therapy aims to activate the immune system to attack cancer cells through natural mechanisms and improve anti-tumor immune response with fewer off-target effects (11). For example, the remarkable development of immune checkpoint inhibitors has shown surprising clinical efficacy. To be specific, the therapy uses antibodies that block the CTLA-4 and PD-1 pathways to treat cancer patients (12). Similarly, Chen et al. show that PD-L1 expression is upregulated in many human tumors, and the antibodies block the PD-L1/PD-1 interaction, resulting in tumor regression in mice (13). However, the majority of CC patients are not eligible for the treatment, suggesting that cancer immunotherapy still needs more research to elucidate the molecular mechanisms and to identify useful biomarkers (14).

It is well known noncoding RNA, including lncRNA and miRNA, are among the major components of the human transcriptome (15, 16). According to the ceRNA theory, lncRNA and mRNA competitively bind to miRNA, due to the strong affinity between lncRNA and miRNA, the binding of miRNA and mRNA is inhibited, resulting in the weakened inhibitory effect of miRNA on mRNA and the increase of mRNA expression closely related to tumorigenesis (17, 18). The bioinformatic analyses about ceRNA related with CC or other cancers have been studied by many researchers in recent years (19–24).

Ferroptosis is an iron-dependent process of modulating cell death with excessive oxidation of phospholipids (25). In recent years, many studies have reported a correlation between ferroptosis and cancer. For example, Wang et al. report that the ferroptosis-related gene circRNA_101093 is essential for protecting lung adenocarcinoma cells from ferroptosis injury (26). Zhang et al. show that adenylate cyclase10 (ADCY10) is an ferroptosis-related gene and promotes the formation of lung adenocarcinoma. Therefore, lung adenocarcinoma patients with high ADCY10 expression may benefit from ferroptosis therapy. The authors conclude that further studies are needed to identify other genes associated with prognosis and ferroptosis of cancer patients (25). On the other hand, studies have shown that reactive oxygen species (ROS) is produced accompanied by ferroptosis, which is closely related to carcinogenesis by inducing DNA double-strands break and oncogene activation (27–31). Study has shown that ferroptotic cancer cells may secret the immune modulators such as Prostaglandin E2 to disturb the effect of immunotherapy, which raises the correlation between ferroptosis and tumor immunity (32, 33). However, the relationship between ferroptosis and immune infiltration in CC has been less studied.

The purpose of the study is to seek genes connected with both immunity and ferroptosis and provide important molecular basis for early noninvasive diagnosis and immunotherapy of CC. In our study, we constructed a ceRNA network and identified NOX4 as target genes associated with immunity and ferroptosis. We used GEPIA, PrognoScan database and qRT-PCR to analyze the NOX4 expression and prognosis, we analysed the related functions and signaling pathways of NOX4 by enrichment analysis. The correlation between NOX4 and tumor immune infiltrating was explored by CIBERSORT and spearman correlation analysis. The flow chart of the whole article was shown in **Supplementary 1**.

## Methods

### Data download

We downloaded the clinical and RNA sequencing data of 371 CC patients from TCGA database (https://portal.gdc.cancer.gov/) using the Data Transfer Tool (34), including 387 CC tissues and 38 normal colon tissues.

### Identification of differentially expressed RNA as well as construction of the ceRNA network

“Limma” package of R language (35, 36) was used to obtain DElncRNA, DEmiRNA and DEmRNA with P < 0.05 and absolute value of log2 fold change (|logFC|) > 2.0, “ggplot2” package (37) was used for visualization of volcanos. We used RNAInter database (www.rnainter.org) to identify potential relationships between lncRNA–miRNA and miRNA–mRNA (38), “Venn Diagram” package (39) was used to draw the interaction between DEmiRNA–mRNA an DEmRNA. Finally, we used the Cytoscape to construct a ceRNA network (40).

### Acquisition of target genes associated with immunity and ferroptosis in ceRNA network

We used the FerrDb database (http://www.zhounan.org/ferrdb/current/) to acquire the ferroptosis-related genes (41), and the ImmPort database (https://www.immport.org/shared/home) to acquire the immune-related genes (42). The overlapping target genes were identified by Venn overlap analysis.

### Expression profile analysis of target genes

We applied TIMER database (https://cistrome.shinyapps.io/timer/) (43) to analyze the expression of target genes in pan-cancer, GEPIA database (http://gepia.cancer-pku.cn/) to analyze the target genes’ expression in CC (44).

### Survival analysis

GEPIA database (44) and PrognoScan database (http://dna00.bio.kyutech.ac.jp/PrognoScan/) (45) were applied to analysis the prognosis of target genes. Median expression of target genes was used as cut off value.

### Functional enrichment analysis

The Gene Ontology (GO) as well as Kyoto Encyclopedia of Genes and Genomes (KEGG) enrichment analysis were performed by the “clusterProfiler” package (46).

### Gene set enrichment analysis

We used GSEA to explore functions of target genes (47). Firstly, the expression data of mRNA gene sets were obtained by R language. We divided CC patients into low expression group and high expression group according to the median of target genes expression. Then, we used GSEA _4.2.3 software to analysis.

### Immune−related analysis of target genes

Twenty-two types of immune cells in different tissue samples were evaluated by CIBERSORT algorithm (48). Spearman analysis was used to evaluate the correlations among the expression of target genes selected in ceRNA network, immune cells, and immune checkpoints CTLA4, PD-1 and PD-L1. We performed visualization of correlations using “Ggplot2” package and “pheatmap” package (49).

### Clinical tissues collection

We collected normal colon and CC specimens from 19 CC patients, during colectomy between April 25, 2021 and June 6, 2021, in the Lanzhou University Second Hospital.

### qRT-PCR

We used RNA lysis solution to extract total RNA (Accurate Biotechnology, China). Evo M-MLV RT Kit II was used to synthesize the first-strand cDNA of mRNA (Accurate Biotechnology, China). CFX 96 real-time PCR system was used to amplify the cDNA. Primer sequences were as follows, β-actin, forward: TGGAACGCTTCACGAATTTGCG, revers: CTAAGTCATAGTCCGCCTAGAAGCA; NOX4, forward: CAGATGTTGGGGCTAGGATTG, revers: GAGTGTTCGGCACATGGGTA.

### Statistical analysis

P < 0.05 was statistically significant. In SPSS23.0 software, we used T-test, Mann-Whitney U test or Wilcoxon rank sum test to count the results.

## Results

### Acquisition of differentially expressed RNA

**Figure 1:**
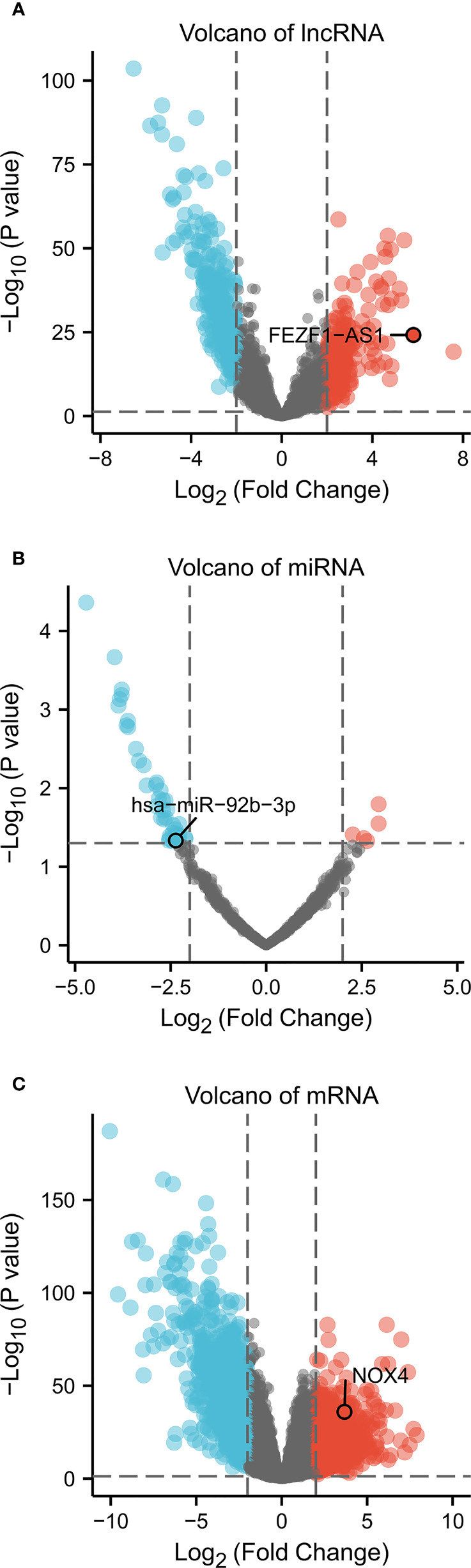
Volcano of differentially expressed RNA in CC (absolute of logFC > 2, P Value < 0.05). Volcano plots of **(A)** 507 DElncRNA, **(B)** 45 DEmiRNA, and **(C)** 1,732 DEmRNA. Red indicates upregulated RNA, and blue represents downregulated RNA. The x-axis depicts log2 Fold Change, the y-axis depicts -log10 (P Value).

We identified 1732 DEmRNA (684 upregulated and 1,048 downregulated), 507 DElncRNA (193 upregulated and 314 downregulated), 45 DEmiRNA (5 upregulated and 40 downregulated) between CC and colon normal specimens. The maps of volcanic distribution were shown in **Figure 1**.

### Prediction of the target DElncRNA/DEmRNA of miRNA as well as construction of the ceRNA network

**Figure 2:**
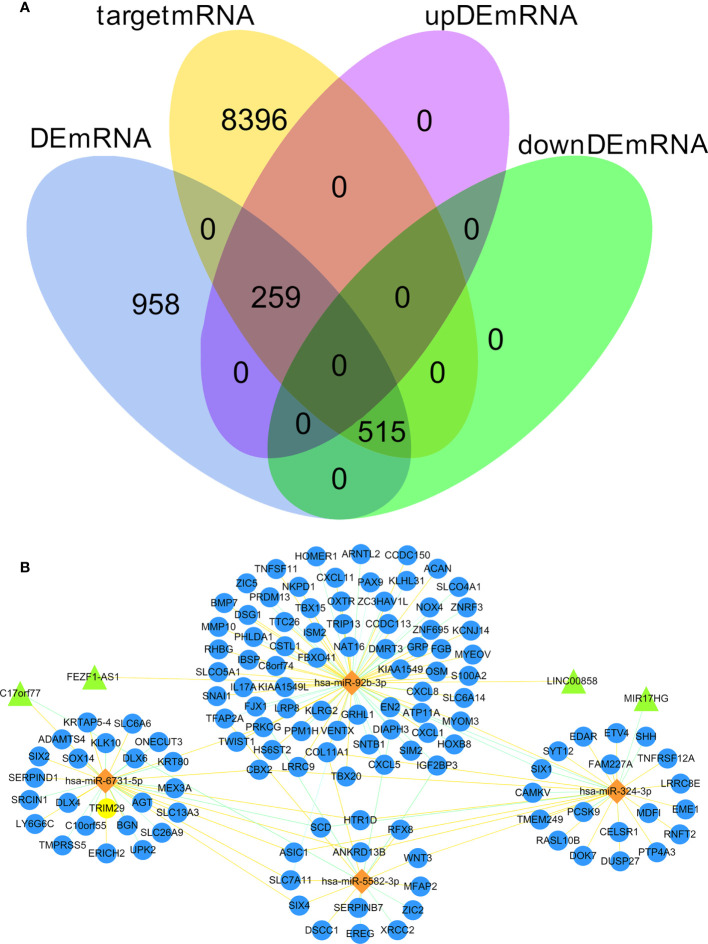
Venn diagram of gene interactions and ceRNA network. **(A)** In the 774 differentially expressed target genes, 259 were up-regulated and 515 down-regulated. The yellow circle represents 9170 target mRNA, the blue circle represents 1732 DEmRNA, the pink circle represents 259 up-regulated differentially expressed target genes, and the green circle represents 515 down-regulated differentially expressed target genes. **(B)** The ceRNA network in CC. Triangles denote lncRNA, diamonds denote miRNA, and circles denote mRNA.

We predicted 1,686 lncRNA–miRNA interactions and 19,185 mRNA–miRNA interactions by using RNAInter database and input the 8 DEmiRNA into the RNAInter database to identify the target DElncRNA/DEmRNA. There were 9170 mRNA predicted as the target genes of 8 DEmiRNA, the intersection of these 9,170 mRNA and 1732 DEmRNA resulted in 774 DEmRNA (259 upregulated and 515 downregulated) (**Figure 2A**). There were 19 DElncRNA predicted as the target genes of 8 DEmiRNA. Thus, we obtained the interaction between DElncRNA–DEmiRNA and DEmiRNA–DEmRNA. To identify highly expressed oncogenes (logFC of lncRNA and mRNA >2, and logFC of miRNA< -2), we identified 4 DElncRNA, 4 DEmiRNA, and 126 DEmRNA to construct the ceRNA network (**Figure 2B**).

### Acquisition of genes association with ferroptosis and immune in ceRNA network

**Figure 3:**
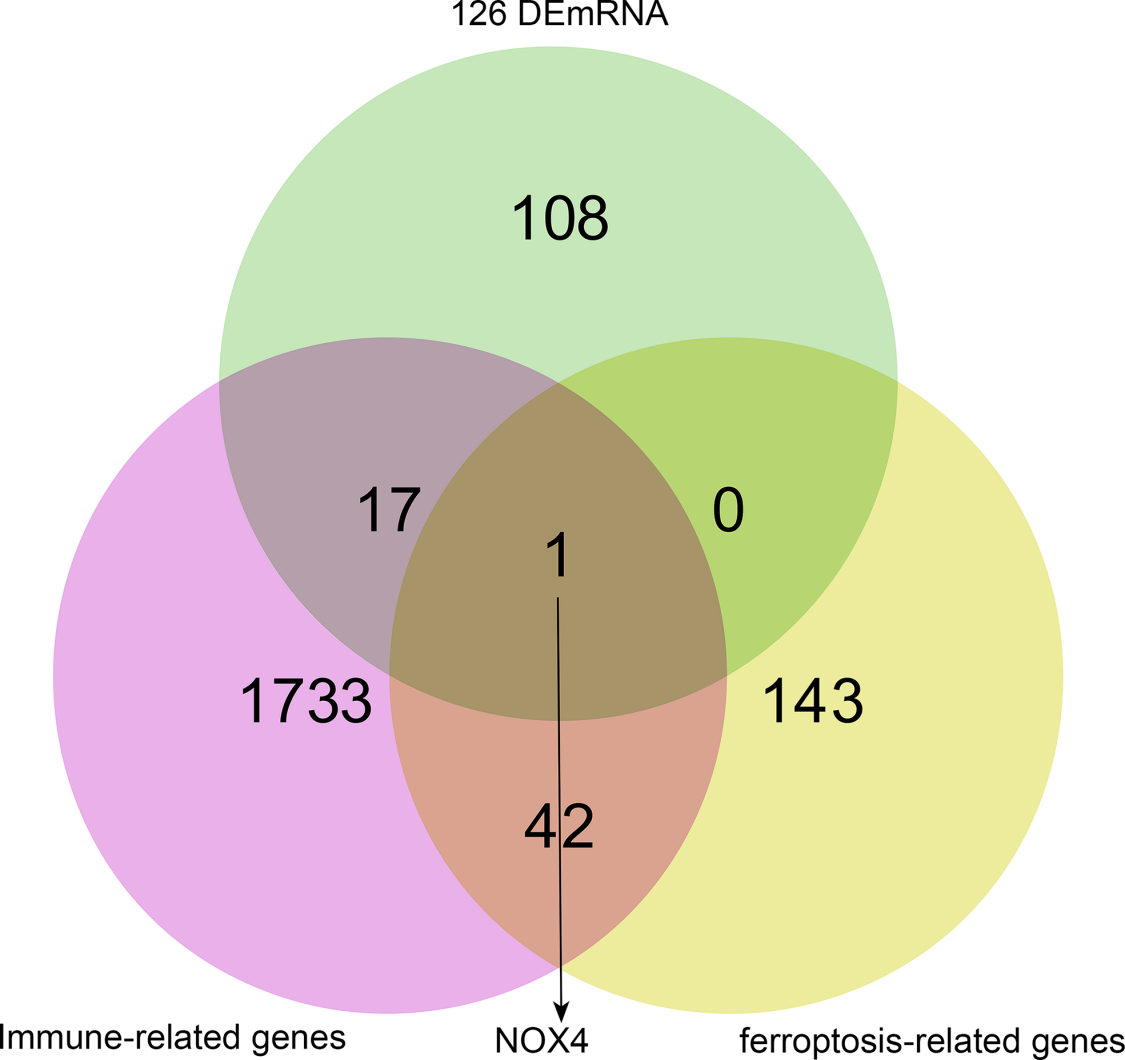
NOX4 was both ferroptosis-related gene and immune-related gene in ceRNA network. The pink circle represents immune-related genes, the yellow circle represents ferroptosis-related genes, the green circle represents 126 DEmRNA.

Genes associated with immune and ferroptosis were downloaded from ImmPort and FerrDb databases, as shown in **Supplementary 2**. We used Venn overlap analysis to obtain the overlapping target genes among immune-related genes, ferroptosis-related genes, and 126 DEmRNA of ceRNA network. The results showed that NOX4 was overlapping target gene (**Figure 3**).

### NOX4 was highly expressed in CC and was connected with bad prognosis

**Figure 4:**
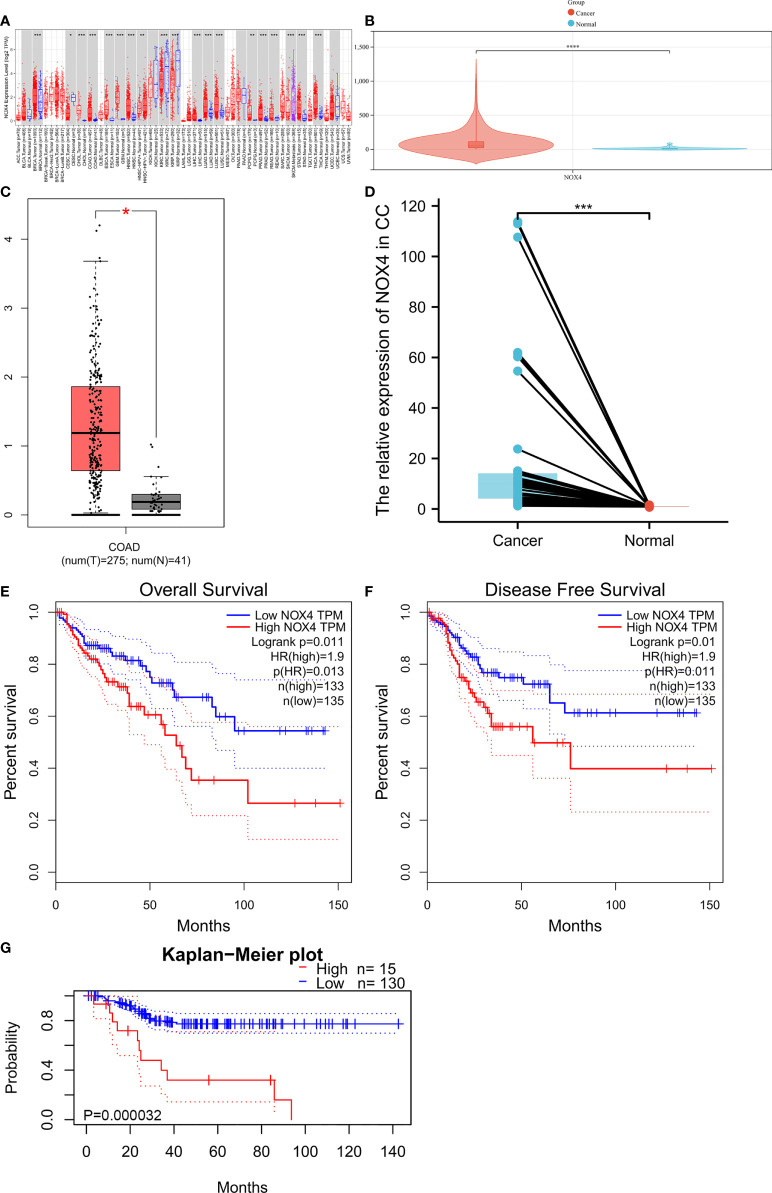
NOX4 was upregulated in CC and associated with poor prognosis. **(A)** The expression of NOX4 in pan-cancer from TIMER database. **(B)** The expression of NOX4 in CC from TCGA database. **(C)** The expression of NOX4 in CC from GEPIA database. **(D)** The expression of NOX4 in 19 fresh normal colon and CC tissues (n=19). **(E)** Overall survival curve for NOX4 in GEPIA database. **(F)** Disease free survival curve for NOX4 in GEPIA database. **(G)** Overall survival curve for NOX4 in PrognoScan database. *P < 0.05, **P < 0.01, ***P < 0.001, ****P < 0.0001.

The expression of NOX4 in pan-cancer from TIMER database was shown in **Figure 4A**. We visualized the expression of NOX4 from TCGA database and GEPIA database, as shown in **Figures 4B and C**. Then, we collected 19 paired fresh CC and normal colon tissues for qRT-PCR analysis (**Figure 4D**). The results showed that compared with normal colon tissues, NOX4 was higher expressed in CC. Survival analysis showed that CC patients with high NOX4 expression had poor disease-free survival and overall survival, as shown in **Figures 4E–G**.

### Functional enrichment of NOX4 and its co-expressed mRNA

**Figure 5:**
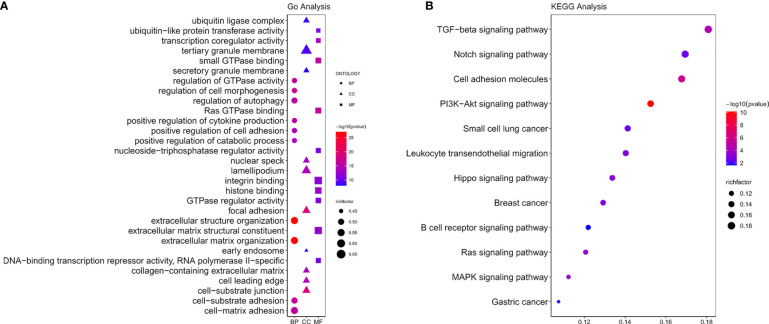
Functional enrichment analysis. **(A)** GO functions of NOX4 and its co-expressed mRNA. **(B)** The KEGG pathway analysis of NOX4 and its co-expressed mRNA.

GO includes three components: molecular functions (MF), cellular components (CC), and biological processes (BP). KEGG analysis indicated NOX4 and its co-expressed mRNA were enriched in a total of 12 signaling pathways, the results were shown in **Figures 5A, B**. Co-expressed mRNA were shown in **Supplementary 3**.

### NOX4 related signaling pathways were analyzed by GSEA

**Figure 6:**
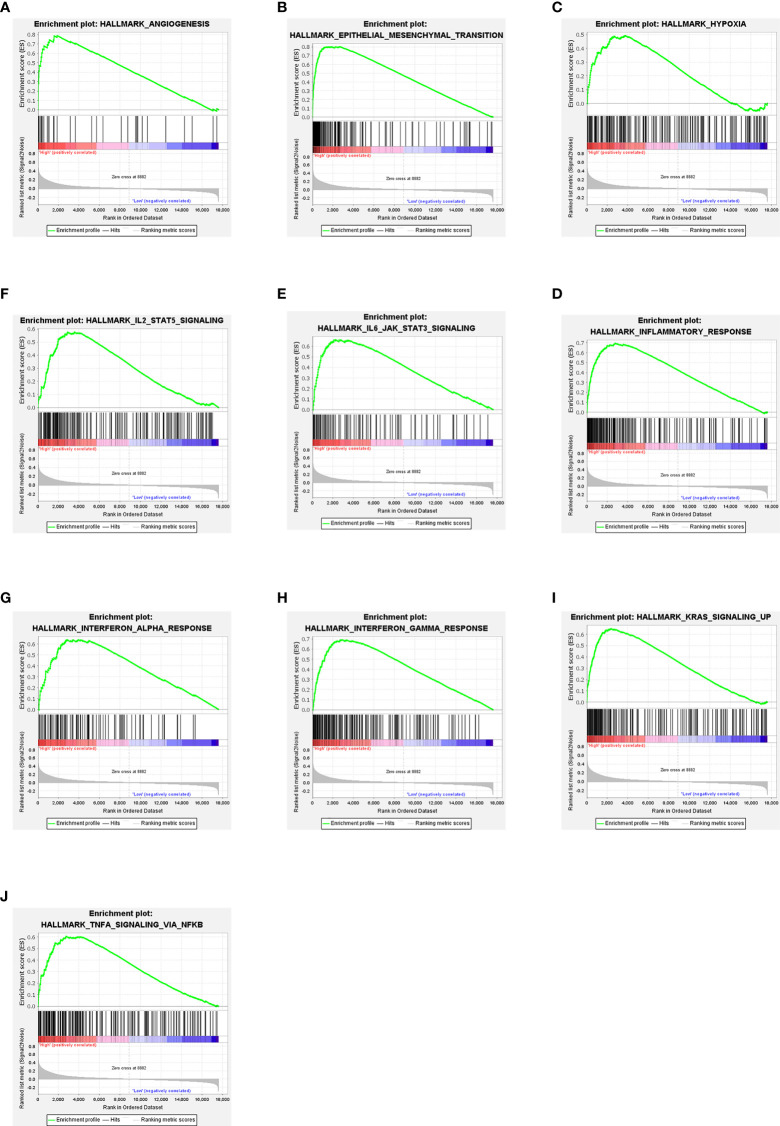
GSEA. **(A–J)** Ten signaling pathways enriched in CC patients with high NOX4 expression.

**Figure 7:**
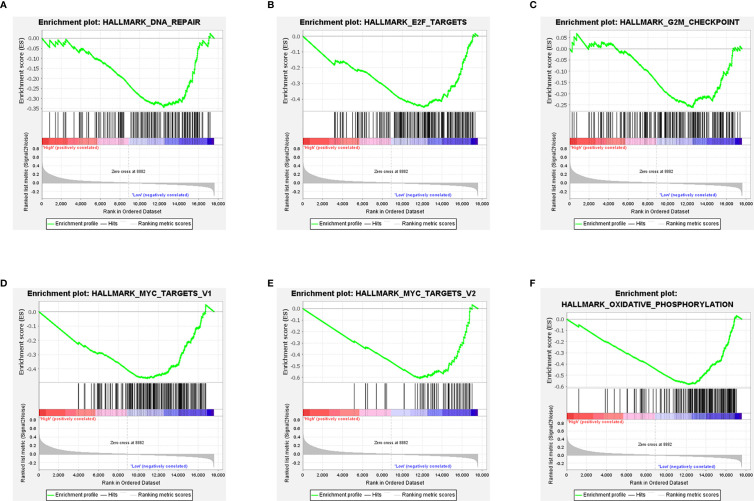
GSEA. **(A–F)** Six signaling pathways enriched in CC patients with low NOX4 expression.

In CC patients with high NOX4 expression, GSEA analysis indicated the upregulated hallmark gene sets were mainly enriched to pathways connected with tumorigenesis and immune response, mainly including IL6 JAK STAT3 signaling, interferon gamma response, TNF-α signaling *via* NF-κB, angiogenesis, KRAS signaling up, and interferon alpha response (**Figure 6**). However, in CC patients with low NOX4 expression, the significantly downregulated hallmark gene sets were enriched to MYC Targets V1, G2M Checkpoint, MYC Targets V2, DNA Repair, and E2F Targets (**Figure 7**).

### Correlation between NOX4 expression and tumor immunity

**Figure 8:**
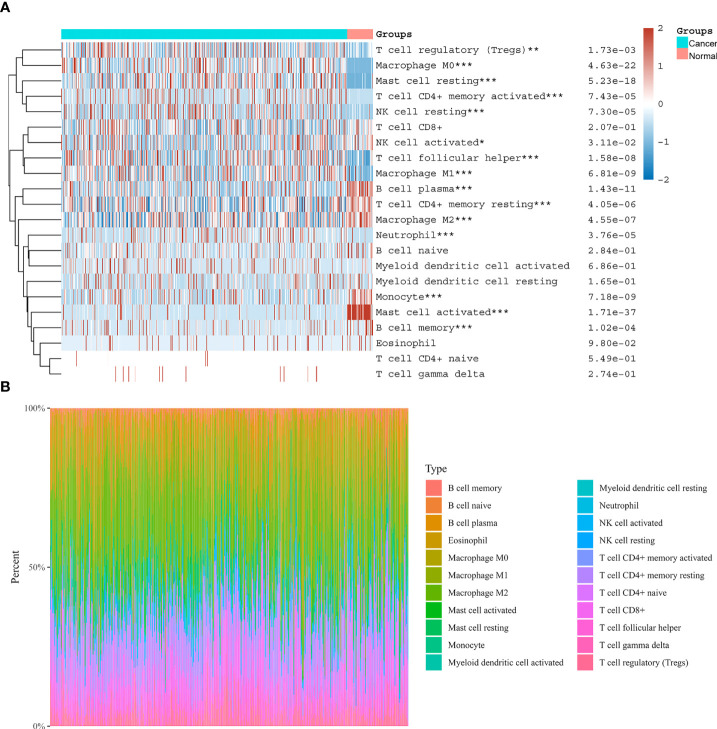
Distribution of immune cells in CC and normal colon tissues. **(A)** Heatmap of immune cell scores. Abscissa represents different groups, ordinate represents immune cell types. Cancer represents CC tissues, Normal represents normal colon tissues. **(B)** The proportion of tumor infiltrating immune cells in each CC sample by the CIBERSORT algorithm. Different colors represent different immune cell types. The horizontal axis represents samples, and the vertical axis represents the percentage of each immune cell in each sample. *P < 0.05, **P < 0.01, ***P < 0.001.

**Figure 9:**
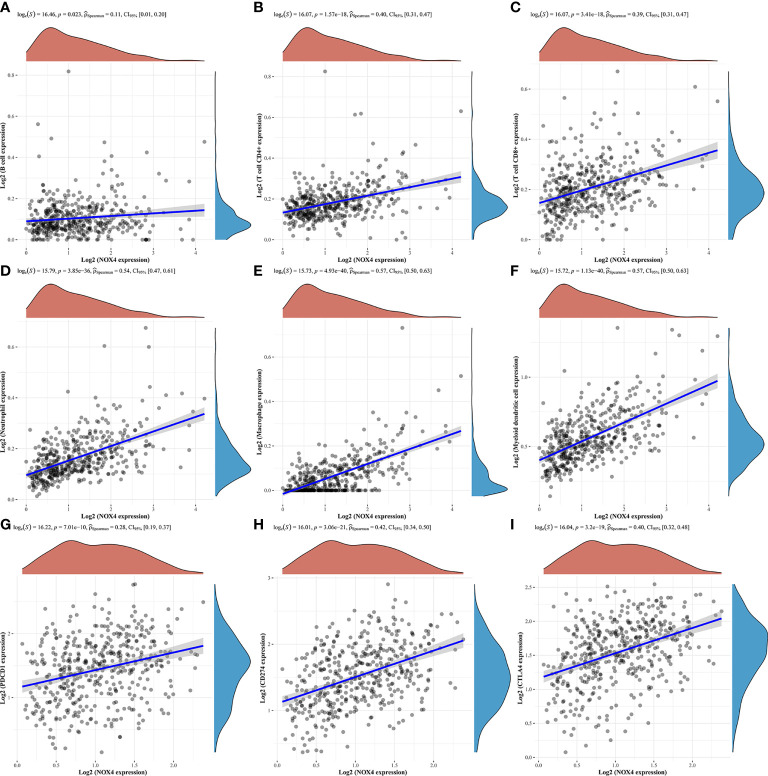
Correlation of NOX4 expression with tumor infiltrating immune cells and immune checkpoints in the tumor microenvironment. NOX4 was significantly correlated with **(A)** B cells, **(B)** CD4+ T cells, **(C)** CD8+ T cells, **(D)** Neutrophil cells, **(E)** Macrophage cells, **(F)** Myeloid dendritic cells, **(G)** PD-1 (PDCD1), **(H)** PD-L1 (CD274), and **(I)** CTLA4 in the TCGA database. P < 0.05.

We evaluated the tumor infiltrating immune cell composition in tissues by CIBERSORT algorithm, as shown in **Figures 8A, B**. The expression of NOX4 was significantly correlated with different types of immune cells, such as CD4+ T cells (p =1.57e-18, r = 0.40), B cells (p =0.023, r = 0.11), CD8+ T cells (p = 3.41e-18, r = 0.39), Neutrophil cells (p = 3.85e-36, r = 0.54), Macrophage cells (p = 4.93e-40, r = 0.57), and Myeloid dendritic cells (p = 1.13e-40, r = 0.57) (**Figures 9A–F**). Spearman correlation analysis indicated NOX4 expression was positively correlated with PD-1 (PDCD1) expression (p = 7.01e-10, r = 0.28), PD-L1 (CD274) expression (p = 3.06 e-21, r = 0.42), and CTLA4 (p = 3.2e-19, r = 0.40) (**Figures 9G-I**).

## Discussion

The early diagnosis rate of CC is low, many patients develop drug resistance, and the mortality rate of CC patients has not decreased (2). Immunotherapy is regarded as an innovative treatment for cancer patients. However, due to lack of effective predictive biomarkers, limited clinical efficacy, and treatment-related adverse events, the broader clinical application of immunotherapy is limited (50). Therefore, it is necessary to further search for immune-related genes with diagnostic and therapeutic value for CC.

We acquired clinic information and transcriptome data of CC patients from the TCGA database, obtained the DElncRNA, DEmiRNA and DEmRNA, then we constructed the ceRNA network. There were 126 DEmRNA in ceRNA network, and finally we have found NOX4, a DEmRNA, was related to both ferroptosis and immunity. NOX4 is a member of NADPH oxidase, and can be induced when cells are in a state of ischemia and hypoxia (51–53). Studies have shown that ROS participates in many key cellular processes, such as proliferation, DNA damage response and angiogenesis, which are obviously connected with the occurrence of tumors (54, 55). NOX4 is a key enzyme in ROS production, which makes it to be more and more attractive to researchers (56). It has been indicated that NOX4 promotes cells migration in CC (57), breast cancer (58–60) as well as pancreatic cancer (61). NOX4 also can regulate the cell cycle to promote the carcinogenesis of melanoma and urothelium (62, 63). Xiao et al. (14) has confirmed that NOX4, as an ferroptosis-related gene, is an effective biomarker for the occurrence of gastric cancer. According to the TIMER database, TCGA database and GEPIA database, NOX4 expression was higher in CC than in colon normal samples, moreover, we confirmed the high expression of NOX4 in CC by collecting fresh clinical surgical specimens. Results from the PrognoScan database and GEPIA database showed that NOX4 was related to disease free survival and overall survival of CC patients, suggesting that NOX4 could be a biomarker to assess the prognosis of CC patients.

Based on GO and KEGG pathway enrichment analysis, we found that NOX4 and its co-expressed mRNA exhibited enrichments for signaling pathways such as PI3K/AKT, TGF-β, MAPK signaling and many more, which were connected with the tumor immune evasion as well as the occurrence of tumors. On the one hand, Fridlender et al. confirm that TGF-β signal transduction in tumor microenvironment promotes the aggregation of N2-like tumor-associated neutrophils, further increasing the tumor immune evasion (64). Regulatory T cells (Tregs) are immune-regulatory subsets of T cells. In the tumor microenvironment, abnormally activated TGF-β signaling makes T cells into Tregs, inhibiting natural killer (NK) cells from killing tumor cells and promoting immunosuppressive effect (65, 66). Conversely, NK cells are activated by inhibition of TGF-β signal transduction (67). On the other hand, Zonneville and Itatani et al. demonstrate that TGF-β signaling in the microenvironment of a tumor plays a key role in angiogenesis (68, 69). Study has shown that over activated TGF-β pathway induces MAPK signaling and PI3K/AKT signaling (70). As we all known, PI3K/AKT is one of the signaling pathways in connection with tumorigenesis. Its overactivation leads to abnormal cell cycle progression, inhibits apoptosis, and inducts angiogenesis (71, 72). Bishnupuri et al. (73) report PI3K/AKT signaling pathway is connected with the occurrence of CC. The above results also reflected the relationship between NOX4 and tumor development, and tumor immunosuppression.

In the CC patients with high NOX4 expression, GSEA analysis enriched to IFN-γ response, angiogenesis, IL6 JAK STAT3 signaling, KRAS signaling up, IFN-α response, and TNFα signaling *via* NF-κB, which were related to tumorigenesis and immune response. Our correlation analysis showed that NOX4 was significantly related to the expressions of CTLA4, PD-L1 (CD274), and PD-1 (PDCD1), acting as immune checkpoints. Elise et al. (74) suggests that IFN-γ increases the expression of PD-L1 in cancer cells, indirectly over-activating the PD-1/PD-L1 signaling pathway. PD-1, which belongs to the CD28 family, is mostly expressed on activated T cells and inhibits host immunity by binding to PD-L1 (75–79). Studies report that cancer cells are protected from the toxic effects of CD8+ T cells by activation of PD-1/PD-L1 signaling pathway, leading to apoptosis and depletion of T cells (75, 80). CTLA-4 is also a T cell inhibitory receptor that binds to CD80 and CD86 expressed by myeloid dendritic cells and inhibits early activation of T cells (81). Based on the above studies, it may be possible to combine PD-1/PD-L1 with NOX4 as immunotherapy targets for CC. Pro-inflammatory mediators such as IL-22, IL-6, IL-2, and IL-17 play a key part in maintaining tumor microenvironment, promoting immunosuppression, angiogenesis, and coordinating the interactions between immune cells (81). It shows IL-6 induces myelogenesis through the STAT3 signaling pathway, inhibits mature bone marrow cells from differentiating, and stimulates the formation of Myeloid-derived suppressor cells (MDSCs) (82). In addition, IL-6 activates MDSCs through the STAT3-NF-κB-IDO pathway in invasive breast cancer (83). MDSCs are immunosuppressive cells that can directly promote the formation of tumor stem cells and protect proliferating tumor cells from apoptosis (84, 85). In the CC patients with low NOX4 expression, GSEA analysis enriched to MYC Targets V1, MYC Targets V2, oxidative phosphorylation, DNA Repair and so on. It has been shown oxidative phosphorylation promotes tumor growth *in vivo (* 86). As a oncogene, MYC can regulates tumor DNA repair, growth, metabolism and apoptosis (87). These findings indicated that NOX4 had potential value in the tumorigenesis and immunosuppression of CC.

We used CIBERSORT algorithm to evaluate the infiltrating immune cell composition in tissues. The results showed that a large proportion of immunosuppressive cells infiltrated in CC tissues. Tumor cells with high expression of ferroptosis related genes recruit and reprogram numerous immunosuppressive cells by secreting cytokines, chemokines, ROS and other proinflammatory mediators. Studies have shown that tumor cells, immunosuppressive cells and tumor stromal cells form an acidic tumor microenvironment, which makes tumor cells more conducive to escape immune surveillance of the host and promotes growth, invasion and metastasis of tumor cells (32, 81, 88). In tumor microenvironment, tumor immunosuppression includes inhibition of activity of CD8+T cells and NK, abnormal function of Myeloid dendritic cells, abnormal transformation from Th1 to Th2, as well as enhanced activity of immunosuppressive cells, including Tregs and MDSCs (81). Our correlation analysis indicated NOX4 was positively connected with the expressions of Neutrophile, CD4+T cells, Macrophage, CD8+T, Myeloid dendritic cells, and B cells. Oshima’s results show cancer cells expressing membrane-Type1-matrix metalloproteinase (MMP) are surrounded by macrophages, leading to the activation of MMP2, which increases the migration and invasion of cancer cells (89). Researches have demonstrated that a tumor’s microenvironment can control tumor occurrence and development through macrophages and myeloid dendritic cells (90, 91). Our results indicated that in the microenvironment of CC tumors, NOX4, a ferroptosis-related gene, was associated closely with immune infiltration and immunosuppression.

## Conclusions

To conclude, our study suggests that NOX4 is associated with both ferroptosis and tumor immunity, and may be a biomarker associated with the prognosis and tumorigenesis of CC and a potential target for immunotherapy of CC in the future.

## Data availability statement

Publicly available datasets were analyzed in this study. This data can be found here: TCGA database (https://portal.gdc.cancer.gov/).

## Ethics statement

The studies involving human participants were reviewed and approved by Lanzhou University Second Hospital medical ethics committee (approval number: 2021A-468). The patients/participants provided their written informed consent to participate in this study.

## Author contributions

XY, PW, and ZRW: software learning, data analysis, and results visualization. XY, ZPW, and JG: experimental verification. XY and XS: manuscript writing. DZ and YY: conceptualization and design, and administration and funding acquisition. All authors contributed to the article and approved the submitted version.

## Funding

This study was supported by Cuiying Scientific and Technological Innovation Program of Lanzhou University Second Hospital (NO. CY2021-QN-A05); Lanzhou Science and technology project (NO. 2022-ZD-104); Cuiying Scientific and Technological Key Cultivation Program of Lanzhou University Second Hospital (CY2018-ZD01). Industrial support and guidance project for institutions of higher learning in Gansu province (2019C-21).

## Acknowledgments

Thanks to TCGA database and RNAInter database for data acquisition, Cuiying Biomedical Research Center for their support of our laboratory equipment and laboratory technology.

## Conflict of interest

The authors declare that the research was conducted in the absence of any commercial or financial relationships that could be construed as a potential conflict of interest.

## Publisher’s note

All claims expressed in this article are solely those of the authors and do not necessarily represent those of their affiliated organizations, or those of the publisher, the editors and the reviewers. Any product that may be evaluated in this article, or claim that may be made by its manufacturer, is not guaranteed or endorsed by the publisher.
