# Supplementary material for: NOX4 has the potential to be a biomarker associated with colon cancer ferroptosis and immune infiltration based on bioinformatics analysis
Source: Front Oncol. 2022 Sep 28;12:968043. doi: 10.3389/fonc.2022.968043 (PMC9554470; doi:10.3389/fonc.2022.968043)
Supplement: Supplementary file 1 [file DataSheet_1.pdf]

Supplementary 1. The flowchart of the whole study

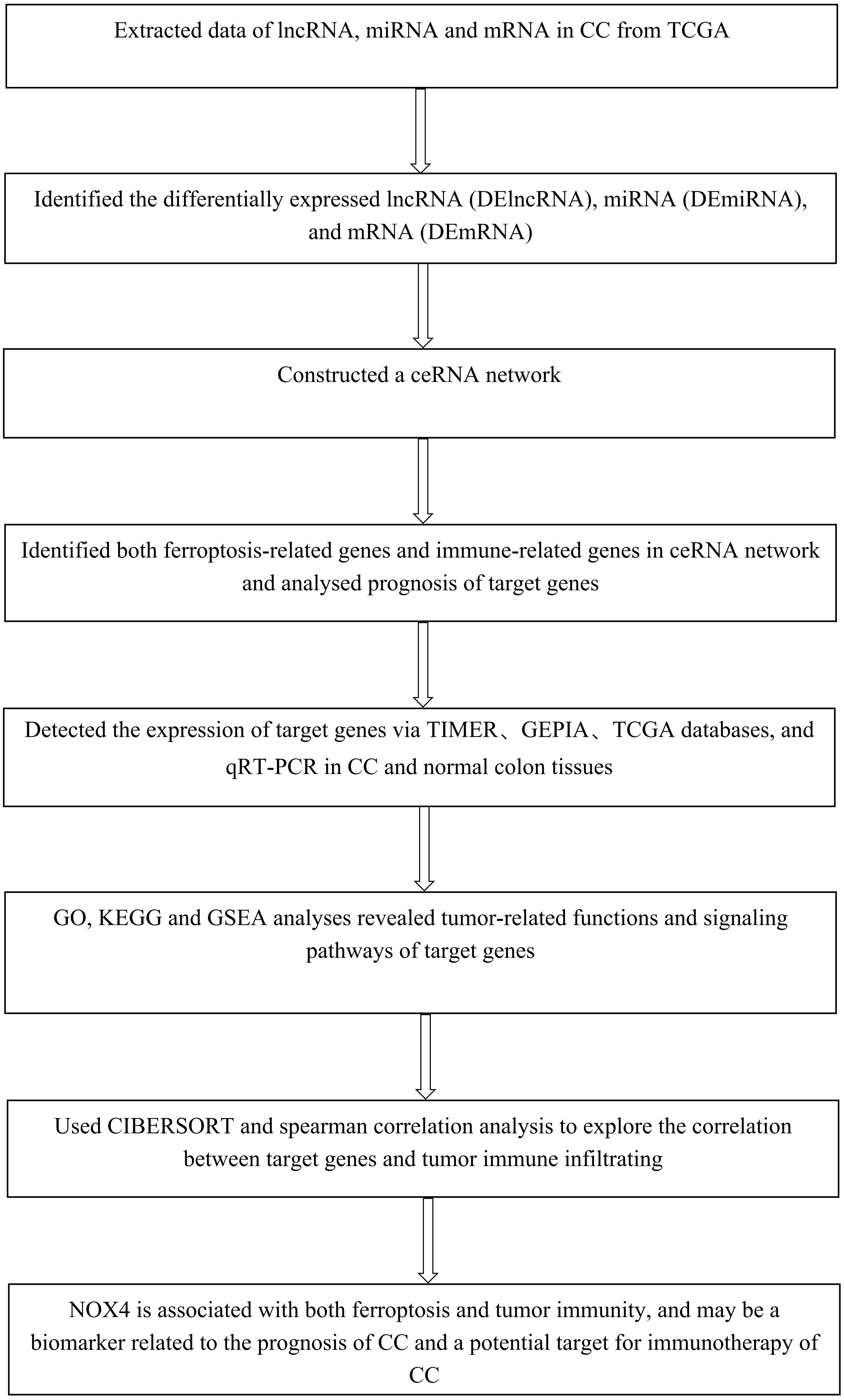

Supplementary 2.1 ferroptosis-related genes

symbol	name	hgncid	evidence	testmethod	testin	testsetting	pathway	proteinfulln	uniprotac	uniprotid	pmid
RPL8	Ribosomal protein L8	HGNC:103	Required in erast	shRNA screening, qPC	Human	NRAS mut	RPL8 :+: F	60S ribosor	P62917	RL8_HUM	22632970
IREB2	iron responsive element binding prote	HGNC:611	Required in erast	shRNA screening, qPC	Human	NRAS mut	IREB2 :+: I	Iron-respon	P48200	IREB2_HU	22632970
ATP5MC3	ATP synthase membrane subunit c lo	HGNC:843	Required in erast	shRNA screening, qPC	Human	NRAS mut	ATP5MC3	ATP syntha	P48201	AT5G3_HU	22632970
CS	Citrate synthase	HGNC:242	Required in erast	shRNA screening, qPC	Human	NRAS mut	CS :+: Ferr	Citrate synt	O75390	CISY_HUN	22632970
EMC2	ER membrane protein complex subun	HGNC:289	Required in erast	shRNA screening, qPC	Human	NRAS mut	EMC2 :+: I	ER membra	Q15006	EMC2_HU	22632970
ACSF2	Acyl-CoA synthetase family member	HGNC:261	Required in erast	shRNA screening, qPC	Human	NRAS mut	ACSF2 :+:	Medium-ch	Q96CM8	ACSF2_HU	22632970
NOX1	Nicotinamide adenine dinucleotide ph	HGNC:788	Suppresses erasti	Inhibition test by diphe	Human	KRAS mut	NOX1 :+: I	NADPH ox	Q9Y5S8	NOX1_HU	22632970
CYBB	Cytochrome b-245 beta chain	HGNC:257	Suppresses erasti	Inhibition test by diphe	Human	KRAS mut	CYBB :+: I	Cytochrom	P04839	CY24B_HU	22632970
NOX3	Nicotinamide adenine dinucleotide ph	HGNC:789	Suppresses erasti	Inhibition test by diphe	Human	KRAS mut	NOX3 :+: I	NADPH ox	Q9HBY0	NOX3_HU	22632970
NOX4	Nicotinamide adenine dinucleotide ph	HGNC:789	Suppresses erasti	Inhibition test by diphe	Human	KRAS mut	NOX4 :+: I	NADPH ox	Q9NPH5	NOX4_HU	22632970
NOX5	Nicotinamide adenine dinucleotide ph	HGNC:148	Suppresses erasti	Inhibition test by diphe	Human	KRAS mut	NOX5 :+: I	NADPH ox	Q96PH1	NOX5_HU	22632970
DUOX1	Dual oxidase 1	HGNC:306	Suppresses erasti	Inhibition test by diphe	Human	KRAS mut	DUOX1 :+	Dual oxida	Q9NRD9	DUOX1_H	22632970
DUOX2	Dual oxidase 2	HGNC:132	Suppresses erasti	Inhibition test by diphe	Human	KRAS mut	DUOX2 :+	Dual oxida	Q9NRD8	DUOX2_H	22632970
G6PD	Glucose-6-phosphate dehydrogenase	HGNC:405	Required in erast	shRNA silencing, RT-	Human	KRAS mut	G6PD :+: F	Glucose-6-	P11413	G6PD_HU	22632970
PGD	Phosphoglycerate dehydrogenase	HGNC:889	Required in erast	shRNA silencing, RT-	Human	KRAS mut	PGD :+: Fe	6-phosphog	P52209	6PGD_HU	22632970
VDAC2	Valtage-dependent anion channels 2	HGNC:126	Required in erast	shRNA silencing, RT-	Human	KRAS mut	VDAC2 :+:	Voltage-de	P45880	VDAC2_H	22632970
PIK3CA	Phosphatidylinositol-4,5-bisphosphate	HGNC:897	Inhibitor blocked	Cell survival assays	Mice	HT22 cells	PIK3CA :-:	Phosphatid	P42336	PK3CA_HU	24739485
FLT3	Fms related tyrosine kinase 3	HGNC:376	Inhibitor blocked	Cell survival assays	Mice	HT22 cells	FLT3 :+: L	Receptor-ty	P36888	FLT3_HUN	24739485
SCP2	Sterol carrier protein 2	HGNC:106	SCP-2 inhibitors	Cell viability test, siRN	Mice	PZL cells	SCP2 :+: F	Non-specifi	P22307	NLTP_HU	25402683
TP53	Tumor protein p53	HGNC:119	Inhibits cystine u	Cell death kinetics, sh	Human, mi	H1299, U2	TP53 :-: SL	Cellular tur	P04637	P53_HUM	25799988
ACSL4	Acyl-CoA synthetase long chain fami	HGNC:357	Deletion of this g	Retroviral-mediated in	Human	Chronic my	ACSL4 :+:	Long-chain	O60488	ACSL4_HU	25965523
LPCAT3	Lysophosphatidylcholine acyltransfer	HGNC:302	Deletion of this g	Retroviral-mediated in	Human	Chronic my	LPCAT3 :+	Lysophospl	Q6P1A2	MBOA5_H	25965523
NRAS	NRAS proto-oncogene, GTPase	HGNC:798	NRAS12V mutar	Cell viability assessed	Human	RMS13 cel	NRAS :+: I	GTPase NF	P01111	RASN_HU	26157704
KRAS	KRAS proto-oncogene, GTPase	HGNC:640	KRAS12V mutar	Cell viability assessed	Human	RMS13 cel	KRAS :+: I	GTPase KF	P01116	RASK_HU	26157704
HRAS	HRas proto-oncogene, GTPase	HGNC:517	HRAS12V mutar	Cell viability assessed	Human	RMS13 cel	HRAS :+: I	GTPase HF	P01112	RASH_HU	26157704
TF	Transferrin	HGNC:117	Essential for the	Killing activity	Mice	Mouse emb	TF :+: Ferr	Serotransfe	P02787	TRFE_HU	26166707
TFRC	Transferrin receptor	HGNC:117	RNAi of transfer	RNAi	Mice	Mouse emb	TFRC :+: F	Transferrin	P02786	TFR1_HUN	26166707
TFR2	Transferrin receptor 2	HGNC:117	RNAi of transfer	RNAi	Mice	Mouse emb	TFR2 :+: F	Transferrin	Q9UP52	TFR2_HUN	26166707
SLC38A1	Solute carrier family 38 member 1	HGNC:134	RNAi knockdow	RNAi knockdown, cell	Mice	Mouse emb	SLC38A1 :	Sodium-co	Q9H2H9	S38A1_HU	26166707
SLC1A5	Solute carrier family 1 member 5	HGNC:109	Pharmacological	RNAi knockdown, cell	Mice	Mouse emb	SLC1A5 :+	Neutral ami	Q15758	AAAT_HU	26166707
GLS2	Glutaminase 2	HGNC:295	Both inhibitor inl	shRNA, western blot,	Mice	Mouse emb	GSL2 :+: G	Glutaminas	Q9UI32	GLSL_HU	26166707
GOT1	Glutamic-oxaloacetic transaminase 1	HGNC:443	RNAi reduced fe	shRNA, qPCR, cell via	Mice	Mouse emb	GOT1 :+: a	Aspartate a	P17174	AATC_HU	26166707
CARS1	Cysteinyl-tRNA synthetase 1	HGNC:149	Required for ferr	siRNA screen, shRNA	Human, rat	HT-1080 fi	CARS1 :-:	'Cysteine--t	P49589	SYCC_HU	26184909
TP53	Tumor protein p53	HGNC:119	Wild type p53 ca	Cell death quantificatic	Human	H1299 cell	TP53 :-: SL	Cellular tur	P04637	P53_HUM	26218928
ALOX5	Arachidonate 5-lipoxygenase	HGNC:435	The 5-Lipoxygen	Cell Viability Assay, R	Mice	HT22 mous	ALOX5 :+:	Arachidona	P09917	LOX5_HU	26235588
KEAP1	Kelch like ECH associated protein 1	HGNC:231	Knockdown of K	Western blot, shRNA	Human, mi	HepG2, He	KEAP1 :-:	Kelch-like	Q14145	KEAP1_HU	26403645
HMOX1	Heme oxygenase 1	HGNC:501	Zinc protoporphy	Cell viability assay, qR	Human, mi	HT-1080 fi	HMOX1 :+	Heme oxyg	P09601	HMOX1_H	26405158
TP53	Tumor protein p53	HGNC:119	p53^3KR/3KR X	Western blot, qRT-PC	Mice	p53^3KR/3	TP53 :-: SL	Cellular tur	P04637	P53_HUM	26943586
TP53	Tumor protein p53	HGNC:119	Incubation with e	Cell viability assay, ph	Human, mi	Mouse emb	TP53 :-: SL	Cellular tur	P04637	P53_HUM	27034505
GLS2	Glutaminase 2	HGNC:295	Upregulated in e	Cell viability assay,we	Mice	Mouse emb	GLS2 :+: F	Glutaminas	Q9UI32	GLSL_HU	27034505
ATG5	Autophagy related 5	HGNC:589	Knockout or kno	shRNA	Human, mi	Mouse emb	ATG5 :+: F	Autophagy	Q9H1Y0	ATG5_HU	27245739
ATG7	Autophagy related 7	HGNC:169	Knockout or kno	shRNA	Human, mi	Mouse emb	ATG7 :+: F	Ubiquitin-li	O95352	ATG7_HU	27245739
NCOA4	Nuclear receptor coactivator 4	HGNC:767	Inhibition suppre	shRNA, gene transfect	Human	PANC1 or	NCOA4 :-:	Nuclear rec	Q13772	NCOA4_H	27245739
TF	Transferrin	HGNC:117	Involved in siran	Western blot, densitom	Human	MDA MB 2	TF :+: Lipi	Serotransfe	P02787	TRFE_HU	27441659
ALOX5	Arachidonate 5-lipoxygenase	HGNC:435	Silencing ALOX	qPCR	Human	G-401 cells	ALOX5 :+:	Arachidona	P09917	LOX5_HU	27506793
ALOX12	Arachidonate 12-lipoxygenase, 12S ty	HGNC:429	Silencing ALOX	qPCR	Human	G-401 cells	ALOX12 :+	Arachidona	P18054	LOX12_HU	27506793
ALOX12B	Arachidonate 12-lipoxygenase, 12R ty	HGNC:430	Silencing ALOX	qPCR	Human	G-401 cells	ALOX12B	Arachidona	O75342	LX12B_HU	27506793
ALOX15	Arachidonate 15-lipoxygenase	HGNC:433	Silencing ALOX	qPCR	Human	G-401 cells	ALOX15 :+	Arachidona	P16050	LOX15_HU	27506793
ALOX15B	Arachidonate 15-lipoxygenase type B	HGNC:434	Silencing ALOX	qPCR, siRNA	Human	G-401, BJe	ALOX15B	Arachidona	O15296	LX15B_HU	27506793
ALOXE3	Arachidonate lipoxygenase 3	HGNC:137	Silencing ALOX	qPCR, siRNA	Human	G-401, BJe	ALOXE3 :-	Hydroperox	Q9BYJ1	LOXE3_HU	27506793
PHKG2	Phosphorylase kinase catalytic subuni	HGNC:893	U-2-OS cells bec	shRNA suppressor scre	Human	U-2-OS, H	PHKG2 :+:	Phosphoryl	P15735	PHKG2_HU	27506793
TFRC	Transferrin receptor	HGNC:117	The gene targets	RNAi screening	Mice	Mouse emb	TFRC :+: L	Transferrin	P02786	TFR1_HUN	27514700
ACO1	Aconitase 1	HGNC:117	The gene targets	RNAi screening	Mice	Mouse emb	ACO1 :+: I	Cytoplasmi	P21399	ACOC_HU	27514700
IREB2	iron responsive element binding prote	HGNC:611	The gene targets	RNAi screening	Mice	Mouse emb	IREB2 :+: I	Iron-respon	P48200	IREB2_HU	27514700
SLC38A1	Solute carrier family 38 member 1	HGNC:134	The gene targets	RNAi screening	Mice	Mouse emb	SLC38A1 :	Sodium-co	Q9H2H9	S38A1_HU	27514700
GLS2	Glutaminase 2	HGNC:295	The gene targets	RNAi screening	Mice	Mouse emb	GLS2 :+: L	Glutaminas	Q9UI32	GLSL_HU	27514700
G6PDX	_NA_	_NA_	The gene targets	RNAi screening	Mice	Mouse emb	G6PDX :+:	_NA_	_NA_		27514700
ULK1	Unc-51 like autophagy activating kina	HGNC:125	Potential positive	RNAi screening, gene	Mice	Mouse emb	ULK1 :+: I	Serine/thre	O75385	ULK1_HU	27514700

Supplementary 2.2 immune-related genes

<table>
<tr><th>Symbol</th><th>Name</th><th>Synonyms</th><th>Chromoson</th><th>Category</th></tr>
<tr><td>AZGP1</td><td>alpha-2-gly</td><td>ZA2G|ZAC</td><td>7</td><td>Antigen_Processing_and_Presentation</td></tr>
<tr><td>B2M</td><td>beta-2-micr</td><td>IMD43</td><td>15</td><td>Antigen_Processing_and_Presentation</td></tr>
<tr><td>CALR</td><td>calreticulin</td><td>CRT|HEL-S</td><td>19</td><td>Antigen_Processing_and_Presentation</td></tr>
<tr><td>CANX</td><td>calnexin</td><td>CNX|IP90|I</td><td>5</td><td>Antigen_Processing_and_Presentation</td></tr>
<tr><td>CD1A</td><td>CD1a mole</td><td>CD1|FCB6|</td><td>1</td><td>Antigen_Processing_and_Presentation</td></tr>
<tr><td>CD1B</td><td>CD1b mole</td><td>CD1|CD1A</td><td>1</td><td>Antigen_Processing_and_Presentation</td></tr>
<tr><td>CD1C</td><td>CD1c mole</td><td>BDCA1|CD</td><td>1</td><td>Antigen_Processing_and_Presentation</td></tr>
<tr><td>CD1D</td><td>CD1d mole</td><td>CD1A|R3|R</td><td>1</td><td>Antigen_Processing_and_Presentation</td></tr>
<tr><td>CD1E</td><td>CD1e mole</td><td>CD1A|R2</td><td>1</td><td>Antigen_Processing_and_Presentation</td></tr>
<tr><td>CD4</td><td>CD4 molec</td><td>CD4mut</td><td>12</td><td>Antigen_Processing_and_Presentation</td></tr>
<tr><td>CD8A</td><td>CD8a mole</td><td>CD8|Leu2|p</td><td>2</td><td>Antigen_Processing_and_Presentation</td></tr>
<tr><td>CD8B</td><td>CD8b mole</td><td>CD8B1|LE</td><td>2</td><td>Antigen_Processing_and_Presentation</td></tr>
<tr><td>CD74</td><td>CD74 mole</td><td>DHLAG|HI</td><td>5</td><td>Antigen_Processing_and_Presentation</td></tr>
<tr><td>CREB1</td><td>cAMP resp</td><td>CREB|CRE</td><td>2</td><td>Antigen_Processing_and_Presentation</td></tr>
<tr><td>CTSB</td><td>cathepsin B</td><td>APPS|CPSI</td><td>8</td><td>Antigen_Processing_and_Presentation</td></tr>
<tr><td>CTSE</td><td>cathepsin E</td><td>CATE</td><td>1</td><td>Antigen_Processing_and_Presentation</td></tr>
<tr><td>CTSL</td><td>cathepsin L</td><td>CATL|CTS</td><td>9</td><td>Antigen_Processing_and_Presentation</td></tr>
<tr><td>CTSS</td><td>cathepsin S</td><td>-</td><td>1</td><td>Antigen_Processing_and_Presentation</td></tr>
<tr><td>FCER1G</td><td>Fc fragmen</td><td>FCRG</td><td>1</td><td>Antigen_Processing_and_Presentation</td></tr>
<tr><td>FCGRT</td><td>Fc fragmen</td><td>FCRN|alph</td><td>19</td><td>Antigen_Processing_and_Presentation</td></tr>
<tr><td>PDIA3</td><td>protein disu</td><td>ER60|ERp5</td><td>15</td><td>Antigen_Processing_and_Presentation</td></tr>
<tr><td>HFE</td><td>homeostatic</td><td>HFE1|HH|H</td><td>6</td><td>Antigen_Processing_and_Presentation</td></tr>
<tr><td>HLA-A</td><td>major histo</td><td>HLAA</td><td>6</td><td>Antigen_Processing_and_Presentation</td></tr>
<tr><td>HLA-B</td><td>major histo</td><td>AS|B-4901|</td><td>6</td><td>Antigen_Processing_and_Presentation</td></tr>
<tr><td>HLA-C</td><td>major histo</td><td>D6S204|HL</td><td>6</td><td>Antigen_Processing_and_Presentation</td></tr>
<tr><td>HLA-DMA</td><td>major histo</td><td>D6S222E|D</td><td>6</td><td>Antigen_Processing_and_Presentation</td></tr>
<tr><td>HLA-DMB</td><td>major histo</td><td>D6S221E|R</td><td>6</td><td>Antigen_Processing_and_Presentation</td></tr>
<tr><td>HLA-DOA</td><td>major histo</td><td>HLA-DNA</td><td>6</td><td>Antigen_Processing_and_Presentation</td></tr>
<tr><td>HLA-DOB</td><td>major histo</td><td>DOB|HLA_</td><td>6</td><td>Antigen_Processing_and_Presentation</td></tr>
<tr><td>HLA-DPA</td><td>major histo</td><td>DP(W3)|DI</td><td>6</td><td>Antigen_Processing_and_Presentation</td></tr>
<tr><td>HLA-DPB1</td><td>major histo</td><td>DPB1|HLA</td><td>6</td><td>Antigen_Processing_and_Presentation</td></tr>
<tr><td>HLA-DQA</td><td>major histo</td><td>CELIAC1|I</td><td>6</td><td>Antigen_Processing_and_Presentation</td></tr>
<tr><td>HLA-DQA</td><td>major histo</td><td>DC-alpha|D</td><td>6</td><td>Antigen_Processing_and_Presentation</td></tr>
<tr><td>HLA-DQB</td><td>major histo</td><td>CELIAC1|I</td><td>6</td><td>Antigen_Processing_and_Presentation</td></tr>
<tr><td>HLA-DRA</td><td>major histo</td><td>HLA-DRA</td><td>6</td><td>Antigen_Processing_and_Presentation</td></tr>
<tr><td>HLA-DRB</td><td>major histo</td><td>DRB1|HLA</td><td>6</td><td>Antigen_Processing_and_Presentation</td></tr>
<tr><td>HLA-DRB</td><td>major histo</td><td>DRB3|HLA</td><td>6</td><td>Antigen_Processing_and_Presentation</td></tr>
<tr><td>HLA-DRB</td><td>major histo</td><td>DR4|DRB4</td><td>6</td><td>Antigen_Processing_and_Presentation</td></tr>
<tr><td>HLA-DRB</td><td>major histo</td><td>-</td><td>6</td><td>Antigen_Processing_and_Presentation</td></tr>
<tr><td>HLA-E</td><td>major histo</td><td>HLA-6.2|Q</td><td>6</td><td>Antigen_Processing_and_Presentation</td></tr>
<tr><td>HLA-F</td><td>major histo</td><td>CDA12|HL</td><td>6</td><td>Antigen_Processing_and_Presentation</td></tr>
<tr><td>HLA-G</td><td>major histo</td><td>MHC-G</td><td>6</td><td>Antigen_Processing_and_Presentation</td></tr>
<tr><td>HLA-H</td><td>major histo</td><td>HLAHP</td><td>6</td><td>Antigen_Processing_and_Presentation</td></tr>
<tr><td>MR1</td><td>major histo</td><td>HLALS</td><td>1</td><td>Antigen_Processing_and_Presentation</td></tr>
<tr><td>HSPA1A</td><td>heat shock</td><td>HEL-S-103</td><td>6</td><td>Antigen_Processing_and_Presentation</td></tr>
<tr><td>HSPA1B</td><td>heat shock</td><td>HSP70-1|H</td><td>6</td><td>Antigen_Processing_and_Presentation</td></tr>
<tr><td>HSPA1L</td><td>heat shock</td><td>HSP70-1L|</td><td>6</td><td>Antigen_Processing_and_Presentation</td></tr>
<tr><td>HSPA2</td><td>heat shock</td><td>HSP70-2|H</td><td>14</td><td>Antigen_Processing_and_Presentation</td></tr>
<tr><td>HSPA4</td><td>heat shock</td><td>APG-2|HEI</td><td>5</td><td>Antigen_Processing_and_Presentation</td></tr>
<tr><td>HSPA5</td><td>heat shock</td><td>BIP|GRP78</td><td>9</td><td>Antigen_Processing_and_Presentation</td></tr>
<tr><td>HSPA6</td><td>heat shock</td><td>HSP70B'</td><td>1</td><td>Antigen_Processing_and_Presentation</td></tr>
<tr><td>HSPA8</td><td>heat shock</td><td>HEL-33|HE</td><td>11</td><td>Antigen_Processing_and_Presentation</td></tr>
<tr><td>HSP90AA1</td><td>heat shock</td><td>EL52|HEL-</td><td>14</td><td>Antigen_Processing_and_Presentation</td></tr>
<tr><td>HSP90AB1</td><td>heat shock</td><td>D6S182|HS</td><td>6</td><td>Antigen_Processing_and_Presentation</td></tr>
<tr><td>ICAM1</td><td>intercellula</td><td>BB2|CD54|</td><td>19</td><td>Antigen_Processing_and_Presentation</td></tr>
<tr><td>IFNA1</td><td>interferon a</td><td>IFL|IFN|IFI</td><td>9</td><td>Antigen_Processing_and_Presentation</td></tr>
<tr><td>IFNA2</td><td>interferon a</td><td>IFN-alpha-</td><td>9</td><td>Antigen_Processing_and_Presentation</td></tr>
<tr><td>IFNA4</td><td>interferon a</td><td>IFN-alpha4</td><td>9</td><td>Antigen_Processing_and_Presentation</td></tr>
</table>

ATG3	Autophagy related 3	HGNC:209	Potential positive	RNAi screening, gene	Mice	Mouse emb	ATG3 :+: I	Ubiquitin-li	Q9NT62	ATG3_HU	27514700	IFNA5	interferon a	IFN-alpha-:	9	Antigen_Processing_and_Presentation
ATG4D	Autophagy related 4D cysteine peptid	HGNC:207	Potential positive	RNAi screening	Mice	Mouse emb	ATG4D :+:	Cysteine pr	Q86TL0	ATG4D_H	27514700	IFNA6	interferon a	IFN-alphaK	9	Antigen_Processing_and_Presentation
ATG5	Autophagy related 5	HGNC:589	Potential positive	RNAi screening	Mice	Mouse emb	ATG5 :+: I	Autophagy	Q9H1Y0	ATG5_HU	27514700	IFNA7	interferon a	IFN-alphaJ|	9	Antigen_Processing_and_Presentation
BECN1	Beclin 1	HGNC:103	Potential positive	RNAi screening	Mice	Mouse emb	BECN1 :+:	Beclin-1	Q14457	BECN1_HU	27514700	IFNA8	interferon a	IFN-alphaB	9	Antigen_Processing_and_Presentation
MAP1LC3.	Microtubule associated protein 1 light	HGNC:683	Potential positive	RNAi screening	Mice	Mouse emb	MAP1LC3.	Microtubul	Q9H492	MLP3A_H	27514700	IFNA10	interferon a	IFN-alphaC	9	Antigen_Processing_and_Presentation
GABARAF	GABA type A receptor associated pro	HGNC:132	Potential positive	RNAi screening	Mice	Mouse emb	GABARAF	Gamma-am	P60520	GBRL2_HU	27514700	IFNA13	interferon a	-	9	Antigen_Processing_and_Presentation
GABARAF	GABA type A receptor associated pro	HGNC:406	Potential positive	RNAi screening	Mice	Mouse emb	GABARAF	Gamma-am	Q9H0R8	GBRL1_HU	27514700	IFNA14	interferon a	IFN-alphaH	9	Antigen_Processing_and_Presentation
ATG16L1	Autophagy related 16 like 1	HGNC:214	Potential positive	RNAi screening	Mice	Mouse emb	ATG16L1 :	Autophagy-	Q676U5	A16L1_HU	27514700	IFNA16	interferon a	IFN-alpha-	9	Antigen_Processing_and_Presentation
WIPI1	WD repeat domain, phosphoinositide	HGNC:254	Potential positive	RNAi screening	Mice	Mouse emb	WIPI1 :+: I	WD repeat	Q5MNZ9	WIPI1_HU	27514700	IFNA17	interferon a	IFN-alphaI|	9	Antigen_Processing_and_Presentation
WIPI2	WD repeat domain, phosphoinositide	HGNC:322	Potential positive	RNAi screening	Mice	Mouse emb	WIPI2 :+: I	WD repeat	Q9Y4P8	WIPI2_HU	27514700	IFNA21	interferon a	IFN-alphaI|	9	Antigen_Processing_and_Presentation
SNX4	Sorting nexin 4	HGNC:111	Potential positive	RNAi screening	Mice	Mouse emb	SNX4 :+: L	Sorting nex	O95219	SNX4_HU	27514700	IFNG	interferon g	IFG|IFI	12	Antigen_Processing_and_Presentation
ATG13	Autophagy related 13	HGNC:290	Knockout of AT(	Gene knockout	Mice	Mouse emb	ATG13 :+:	Autophagy-	O75143	ATG13_HU	27514700	KIR2DL1	killer cell ir	CD158A|K	19	Antigen_Processing_and_Presentation
ULK2	Unc-51 like autophagy activating kina	HGNC:134	Knockout of ULI	Gene knockout	Mice	Mouse emb	ULK2 :+: I	Serine/thre	Q8IYT8	ULK2_HU	27514700	KIR2DL2	killer cell ir	CD158B1|C	19	Antigen_Processing_and_Presentation
NCOA4	Nuclear receptor coactivator 4	HGNC:767	Elimination of N	RNAi knockdown, wes	Human, mi	Mouse emb	NCOA4 :+:	Nuclear rec	Q13772	NCOA4_H	27514700	KIR2DL3	killer cell ir	CD158B2|C	19	Antigen_Processing_and_Presentation
ACSL4	Acyl-CoA synthetase long chain fami	HGNC:357	Knockdown inhil	shRNA, gene transfect	Human	LNCaP (hu	ACSL4 :+:	Long-chain	O60488	ACSL4_HU	27565726	KIR2DL4	killer cell ir	CD158D|G	19	Antigen_Processing_and_Presentation
TP53	Tumor protein p53	HGNC:119	p53 acetylation h	Mass spectrometry scr	Human, mi	H1299 cells	TP53 :-: SL	Cellular tur	P04637	P53_HUMA	27705786	KIR2DS1	killer cell ir	CD158H|C	19	Antigen_Processing_and_Presentation
SAT1	Spermidine/spermine N1-acetyltransf	HGNC:105	p53-mediated act	qRT-PCR, siRNA, CR	Human, mi	H1299, U2(	SAT1 :+: A	Diamine ac	P21673	SAT1_HUM	27698118	KIR2DS3	killer cell ir	NKAT7	19	Antigen_Processing_and_Presentation
ALOX15	Arachidonate 15-lipoxygenase	HGNC:433	SAT1- and ROS-	Cell death count, qRT-	Human	H1299, U2(	ALOX15 :-	Arachidona	P16050	LOX15_HU	27698118	KIR2DS4	killer cell ir	CD158I|KI	19	Antigen_Processing_and_Presentation
ACSL4	Acyl-CoA synthetase long chain fami	HGNC:357	Inhibition of AC	Cell death count, LC-M	Mice	Mouse emb	ACSL4 :+:	Long-chain	O60488	ACSL4_HU	27842066	KIR2DS5	killer cell ir	CD158G|N	19	Antigen_Processing_and_Presentation
LPCAT3	Lysophosphatidylcholine acyltransfer	HGNC:302	Knockdown of L	shRNA	Mice	Mouse lung	LPCAT3 :+	Lysophospl	Q6P1A2	MBOA5_H	27842066	KIR3DL1	killer cell ir	CD158E1|K	19	Antigen_Processing_and_Presentation
ALOX15	Arachidonate 15-lipoxygenase	HGNC:433	Liproxstatin-1 in	Identification of oxyge	Mice	Kidney of (	ALOX15 :-	Arachidona	P16050	LOX15_HU	27842066	KIR3DL2	killer cell ir	3DL2|CD1	19	Antigen_Processing_and_Presentation
ACSL4	Acyl-CoA synthetase long chain fami	HGNC:357	An essential prof	Cell viability, immuno	Human, mi	Mouse emb	ACSL4 :+:	Long-chain	O60488	ACSL4_HU	27842070	KLRC1	killer cell le	CD159A|N	12	Antigen_Processing_and_Presentation
KEAP1	Kelch like ECH associated protein 1	HGNC:231	Keap 1 silencing	Cell viability assay, we	Mice	Head and n	KEAP1 :-:	Kelch-like	Q14145	KEAP1_HU	28012440	KLRC2	killer cell le	CD159c|NK	12	Antigen_Processing_and_Presentation
EGFR	Epidermal growth factor receptor	HGNC:323	Cell death in acti	Fluorescence-activated	Human	Human ma	EGFR :+: N	Epidermal g	P00533	EGFR_HU	28297659	KLRC3	killer cell le	NKG2-E|N	12	Antigen_Processing_and_Presentation
NOX4	NADPH oxidase 4	HGNC:789	Inhibition of NA	Fluorescence-activated	Human	Human ma	NOX4 :+: I	NADPH ox	Q9NPH5	NOX4_HU	28297659	KLRD1	killer cell le	CD94	12	Antigen_Processing_and_Presentation
MAPK3	Mitogen-activated protein kinase 3	HGNC:687	Inhibiting EGFR	Immunoblots, lucifer y	Human	Human ma	MAPK :-: (	Mitogen-ac	P27361	MK03_HU	28297659	LTA	lymphotoxi	LT|TNFB|T	6	Antigen_Processing_and_Presentation
MAPK1	Mitogen-activated protein kinase 1	HGNC:687	Inhibiting EGFR	Immunoblots, lucifer y	Human	Human ma	MAPK :-: (	Mitogen-ac	P28482	MK01_HU	28297659	CIITA	class II maj	C2TA|CIIT	16	Antigen_Processing_and_Presentation
BID	BH3 interacting domain death agonist	HGNC:105	BID deletion pre	Cell viability, western	Mice	HT-22 cells	BID :+: Fer	BH3-intera	P55957	BID_HUM	28384611	MICA	MHC class	MIC-A|PEI	6	Antigen_Processing_and_Presentation
ACSL4	Acyl-CoA synthetase long chain fami	HGNC:357	Knockout of Acs	CRISPR/Cas9, western	Human, mi	NIH3T3, H	ACSL4 :+:	Long-chain	O60488	ACSL4_HU	28551825	MICB	MHC class	PERB11.2	6	Antigen_Processing_and_Presentation
ZEB1	Zinc finger E-box binding homeobox	HGNC:116	Knockout of ZEI	sgRNA, cell viability	Human	KP4 pancre	ZEB1 :+: L	Zinc finger	P37275	ZEB1_HUN	28678785	NFYA	nuclear tran	CBF-A|CB	6	Antigen_Processing_and_Presentation
KEAP1	Kelch like ECH associated protein 1	HGNC:231	Keap1 inhibition	Cell viability analysis	Human, rat	F98, U87 g	KEAP1 :+:	Kelch-like	Q14145	KEAP1_HU	28805788	NFYB	nuclear tran	CBF-A|CB	12	Antigen_Processing_and_Presentation
DPP4	Dipeptidyl peptidase 4	HGNC:300	Required for ferr	Cell death, RNAi, west	Human, mi	TP53-/- HC	DPP4 :+: L	Dipeptidyl	P27487	DPP4_HUN	28813679	NFYC	nuclear tran	CBF-C|CBI	1	Antigen_Processing_and_Presentation
ALOX15	Arachidonate 15-lipoxygenase	HGNC:433	Suppression of fe	siRNA, transfection, w	Human	HT1080, Pa	ALOX15 :-	Arachidona	P16050	LOX15_HU	28837253	LGMN	legumain	AEP|LGMN	14	Antigen_Processing_and_Presentation
ALOX12	Arachidonate 12-lipoxygenase, 12S ty	HGNC:429	12-LOX inhibito	siRNA, transfection, w	Human	HT1080, Pa	ALOX12 :-	Arachidona	P18054	LOX12_HU	28837253	PSMB8	proteasome	ALDD|D6S	6	Antigen_Processing_and_Presentation
CDKN2A	Cyclin dependent kinase inhibitor 2A	HGNC:178	Combination of /	RNAi, immunoblot, ph	Human, mi	H1299, Sac	CDKN2A :	Cyclin-dep	P42771	CDN2A_H	28985506	PSMC1	proteasome	P26S4|S4|p	14	Antigen_Processing_and_Presentation
PEBP1	Phosphatidylethanolamine binding pro	HGNC:863	Elevated levels o	Western blot, siRNA, c	Human, mi	HK2, HAE	(PEBP1/15	Phosphatid	P30086	PEBP1_HU	29053969	PSMC2	proteasome	MSS1|Nbla	7	Antigen_Processing_and_Presentation
SOCS1	Suppressor of cytokine signaling 1	HGNC:193	Expression of SC	Cell death assay, cell v	Human	U2OS or IN	SOCS1 :+:	Suppressor	O15524	SOCS1_HU	29081404	PSMC3	proteasome	TBP1	11	Antigen_Processing_and_Presentation
CDO1	Cysteine dioxygenase type 1	HGNC:179	CDO1 suppressio	siRNA, western blot, N	Human	Gastric can	CDO1 :-: G	Cysteine di	Q16878	CDO1_HU	29144989	PSMC4	proteasome	MIP224|RP	19	Antigen_Processing_and_Presentation
MYB	MYB proto-oncogene, transcription fa	HGNC:754	Erastin-induced f	Western blot, siRNA, c	Human	Gastric can	MYB :+: C	Transcripti	P10242	MYB_HUN	29144989	PSMC5	proteasome	S8|SUG-1|S	17	Antigen_Processing_and_Presentation
HMOX1	Heme oxygenase 1	HGNC:501	Inhibiting HO-1 (	Cell viability assay, qR	Human	MDA-MB-	HMOX1 :+	Heme oxyg	P09601	HMOX1_H	29274359	PSMC6	proteasome	SUG2|p42	14	Antigen_Processing_and_Presentation
MAPK8	Mitogen-activated protein kinase 8	HGNC:688	JNK1/2 inhibitor	MitoSOX probe, lumin	Rat	PC12 cells	MAPK8 :+	Mitogen-ac	P45983	MK08_HU	29330409	PSMD1	proteasome	P112|Rpn2|	2	Antigen_Processing_and_Presentation
MAPK9	Mitogen-activated protein kinase 9	HGNC:688	JNK1/2 inhibitor	MitoSOX probe, lumin	Rat	PC12 cells	MAPK9 :+	Mitogen-ac	P45984	MK09_HU	29330409	PSMD2	proteasome	P97|RPN1|S	3	Antigen_Processing_and_Presentation
MAPK1	Mitogen-activated protein kinase 1	HGNC:687	ERK1/2 inhibitor	MitoSOX probe, lumin	Rat	PC12 cells	MAPK1 :+	Mitogen-ac	P28482	MK01_HU	29330409	PSMD3	proteasome	P58|RPN3|S	17	Antigen_Processing_and_Presentation
MAPK3	Mitogen-activated protein kinase 3	HGNC:687	ERK1/2 inhibitor	MitoSOX probe, lumin	Rat	PC12 cells	MAPK3 :+	Mitogen-ac	P27361	MK03_HU	29330409	PSMD4	proteasome	AF|AF-1|A	1	Antigen_Processing_and_Presentation
SLC1A5	Solute carrier family 1 member 5	HGNC:109	Overexpression c	Cell viability assay, lip	Human	Melanoma	SLC1A5 :+	Neutral am	Q15758	AAAT_HU	29348676	PSMD5	proteasome	S5B	9	Antigen_Processing_and_Presentation
CHAC1	ChaC glutathione specific gamma-glu	HGNC:286	CHAC1 degradat	Western blot, real-time	Human	MDA-MB-	CHAC1 :-:	Glutathione	Q9BUX1	CHAC1_H	29383104	PSMD7	proteasome	MOV34|P4	16	Antigen_Processing_and_Presentation
MAPK14	Mitogen-activated protein kinase 14	HGNC:687	Ferroptosis was b	Cell viability assay, lip	Mice	TM4 Sertol	MAPK14 :-	Mitogen-ac	Q16539	MK14_HU	29436589	PSMD8	proteasome	HEL-S-91n	19	Antigen_Processing_and_Presentation
LINC0047	Long intergenic non-protein coding R	HGNC:213	Increases erastin-	Bisulfite sequencing, R	Human	Lung cance	LINC0047	Putative un	Q9H8W2	CF155_HU	29588351	PSMD10	proteasome	dJ889N15.2X		Antigen_Processing_and_Presentation
NOX4	NADPH oxidase 4	HGNC:789	Activated Nox4	Western blot, LDH rel	Human, rat	Rat C6, hur	NOX4 :+: I	NADPH ox	Q9NPH5	NOX4_HU	29702192	PSMD11	proteasome	Rpn6|S9|p4	17	Antigen_Processing_and_Presentation
GOT1	Glutamic-oxaloacetic transaminase 1	HGNC:443	Overexpression c	Immunoblotting, lucife	Human	A375 and (	GOT1 :+: a	Aspartate a	P17174	AATC_HU	30035324	PSMD13	proteasome	HSPC027|F	11	Antigen_Processing_and_Presentation
BECN1	Beclin 1	HGNC:103	Knockdown inhil	shRNA, western blot, c	Human, mi	HCT116, C	BECN1 :-:	Beclin-1	Q14457	BECN1_HU	30057310	PSME1	proteasome	HEL-S-129	14	Antigen_Processing_and_Presentation
PRKAA2	Protein kinase AMP-activated catalyti	HGNC:937	Inhibition of PRI	siRNA, western blot, c	Human	HCT116 an	PRKAA2 :	5'-AMP-act	P54646	AAPK2_HU	30057310	PSME1	proteasome	HEL-S-129	14	Antigen_Processing_and_Presentation
PRKAA1	Protein kinase AMP-activated catalyti	HGNC:937	Inhibition of PRI	siRNA, western blot, c	Human	HCT116 an	PRKAA1 :	5'-AMP-act	Q13131	AAPK1_HU	30057310	PSME2	proteasome	PA28B|PA2	14	Antigen_Processing_and_Presentation
ELAVL1	ELAV like RNA binding protein 1	HGNC:331	ELAVL1 siRNA	Immunohistochemistry	Human, mi	Human live	ELAVL1 :-	ELAV-like	Q15717	ELAV1_HU	30081711	PSME2	proteasome	PA28B|PA2	14	Antigen_Processing_and_Presentation
BAP1	BRCA1 associated protein 1	HGNC:950	Suppresses SLC7	Flow cytometry, cell v	Human	HEK-293T	BAP1 :-: Sl	Ubiquitin c	Q92560	BAP1_HUI	30202049	RELB	RELB prot	I-REL|IMD	19	Antigen_Processing_and_Presentation
TP53	Tumor protein p53	HGNC:119	Facilitates ART-i	Western blot, immuno	Rat	Hepatic ste	TP53 :+: F	Cellular tur	P04637	P53_HUMA	30321484	RFX5	regulatory f	-	1	Antigen_Processing_and_Presentation
ABCC1	ATP binding cassette subfamily C me	HGNC:51	Accelerates ferro	Western blot, GSH ass	Human	HAP1, H12	ABCC1 :-:	Multidrug r	P33527	MRP1_HU	30726737	RFXAP	regulatory f	-	13	Antigen_Processing_and_Presentation
ACSL4	Acyl-CoA synthetase long chain fami	HGNC:357	Inhibition suppre	siRNA, western blot, n	Human, mi	Male C57B	ACSL4 :+:	Long-chain	O60488	ACSL4_HU	30737476	SLC10A2	solute carri	ASBT|IBA	13	Antigen_Processing_and_Presentation
MIR6852	microRNA 6852	HGNC:499	Promotes ferropt	qRT-PCR, gene knock	Human	A549 and S	MIR6852 :-	_NA_	_NA_		30787392	TAP1	transporter	ABC17|AB	6	Antigen_Processing_and_Presentation
ACVR1B	Activin A receptor type 1B	HGNC:172	Inhibition attenu	Western blot, siRNA, g	Human	HK-2 cells	ACVR1B :	Activin rec	P36896	ACV1B_H	30804470	TAP2	transporter	ABC18|AB	6	Antigen_Processing_and_Presentation

TGFBR1	Transforming growth factor beta recep	HGNC:117	Inhibition attenua	Western blot, siRNA, g	Human	HK-2 cells	TGFBR1 :-	TGF-beta r	P36897	TGFR1_HU	30804470	TAPBP	TAP bindin	NGS17|TA	6	Antigen_Processing_and_Presentation
BAP1	BRCA1 associated protein 1	HGNC:950	Promotes ferropto	Immunoprecipitation, v	Human	HEK293T (	BAP1 :-: Sl	Ubiquitin c	Q92560	BAP1_HUI	30907299	THBS1	thrombospo	THBS|THB	15	Antigen_Processing_and_Presentation
EPAS1	Endothelial PAS domain protein 1	HGNC:337	A driver of ferrop	CRISPR, sgRNA, shRI	Mice	786-O cells	EPAS1 :+:	Endothelial	Q99814	EPAS1_HU	30962421	SEM1	SEM1 26S	C7orf76|DS	7	Antigen_Processing_and_Presentation
HILPDA	Hypoxia inducible lipid droplet associ	HGNC:288	Promotes ferropto	RNA-Seq, western blo	Mice	786-O cells	HILPDA :+	Hypoxia-in	Q9Y5L2	HLPDA_H	30962421	KLRC4	killer cell le	NKG2-F|NI	12	Antigen_Processing_and_Presentation
HIF1A	Hypoxia inducible factor 1 subunit alp	HGNC:491	Re-sensitized HI	cDNA screenin, cell vi	Mice	786-O cells	HIF1A :+: I	Hypoxia-in	Q16665	HIF1A_HU	30962421	AP3B1	adaptor rela	ADTB3|AD	5	Antigen_Processing_and_Presentation
ALOX12	Arachidonate 12-lipoxygenase, 12S ty	HGNC:429	An essential facto	RNAi, qRT-PCR, west	Human, mi	H1299 cells	ALOX12 :+	Arachidona	P18054	LOX12_HU	30962574	RFXANK	regulatory f	ANKRA1|F	19	Antigen_Processing_and_Presentation
ACSL4	Acyl-CoA synthetase long chain fami	HGNC:357	Required for ferro	Western blot, cell deat	Human	U2OS	ACSL4 :+:	Long-chain	O60488	ACSL4_HU	30962574	PSMD6	proteasome	Rpn7|S10|S	3	Antigen_Processing_and_Presentation
HMOX1	Heme oxygenase 1	HGNC:501	Enhances the fer	Gene transfection, wes	Human	H1299 cells	HMOX1 :+	Heme oxyg	P09601	HMOX1_H	31036877	PSME3	proteasome	HEL-S-283	17	Antigen_Processing_and_Presentation
IFNG	Interferon gamma	HGNC:543	Interferon gamma	BODIPY-C11, liperflu	Human, mi	HT-1080, A	IFNG :-: SI	Interferon g	P01579	IFNG_HUN	31043744	PSMD14	proteasome	PAD1|POH	2	Antigen_Processing_and_Presentation
ANO6	Anoctamin 6	HGNC:252	Essential for ferro	Immunocytochemistry,	Human, mi	A549, Cal2	ANO6 :+: I	Anoctamin-	Q4KMQ2	ANO6_HU	31060306	CLEC4M	C-type lecti	CD209L|CI	19	Antigen_Processing_and_Presentation
LPIN1	Lipin 1	HGNC:133	Overexpression c	Enzyme-linked immur	Mice	C57BL/6 m	LPIN1 :+: I	Phosphatid	Q14693	LPIN1_HU	31061954	IFI30	IFI30 lysos	GILT|IFI-3	19	Antigen_Processing_and_Presentation
HMGB1	High mobility group box 1	HGNC:498	Required for eras	Gene transfection, RN	Human, mi	HL-60 cell,	HMGB1 :+	High mobil	P09429	HMGB1_H	31105999	PROCR	protein C re	CCCA|CCI	20	Antigen_Processing_and_Presentation
TNFAIP3	TNF alpha induced protein 3	HGNC:118	Overexpression i	Lentivirus transductior	Human	HUVEC ce	TNFAIP3 :	Tumor necr	P21580	TNAP3_HU	31160087	ADRM1	adhesion re	ARM-1|AR	20	Antigen_Processing_and_Presentation
TLR4	Toll like receptor 4	HGNC:118	Knockdown inhil	Immunohistochemistry	Rat	Sprague Da	TLR4 :+: F	Toll-like re	O00206	TLR4_HUI	31196626	ECPAS	Ecm29 prot	ECM29|KL	9	Antigen_Processing_and_Presentation
NOX4	NADPH oxidase 4	HGNC:789	Knockdown inhil	Immunohistochemistry	Rat	Sprague Da	NOX4 :+: I	NADPH ox	Q9NPH5	NOX4_HU	31196626	TRPC4AP	transient re	C20orf188|	20	Antigen_Processing_and_Presentation
ATF3	Activating transcription factor 3	HGNC:785	Promotes ferropto	Cell viability assay, tra	Human	HT1080 ce	ATF3 :-: SI	Cyclic AM	P18847	ATF3_HUI	31273299	CD209	CD209 mol	CDSIGN|C	19	Antigen_Processing_and_Presentation
ATM	ATM serine/threonine kinase	HGNC:795	Essential for ferro	siRNA, western blot, n	Human	MDA-MB-	ATM :-: M	Serine-prot	Q13315	ATM_HUM	31320750	UBXN1	UBX doma	2B28|SAKS	11	Antigen_Processing_and_Presentation
YY1AP1	YY1 associated protein 1	HGNC:309	Makes cells more	Cell death, cell viabilit	Human, mi	Mouse emb	YAP :+: A(	YY1-associ	Q9H869	YYAP1_HI	31341276	ERAP1	endoplasmi	A-LAP|AL	5	Antigen_Processing_and_Presentation
EGLN2	Egl-9 family hypoxia inducible factor	HGNC:146	Inhibiting EGLN	Cytotoxicity assays, w	Human, mi	Calu-1, TH	EGLN2 :-:	Egl nine ho	Q96KS0	EGLN2_HI	31355331	TAPBPL	TAP bindin	TAPBP-R|T	12	Antigen_Processing_and_Presentation
MIOX	Myo-inositol oxygenase	HGNC:145	Overexpression e	MTT assay, measurem	Human, mi	HK-2 cells,	MIOX :-: C	Inositol oxy	Q9UGB7	MIOX_HU	31437128	KIR2DL5A	killer cell ir	CD158F|KI	19	Antigen_Processing_and_Presentation
TAZ	Tafazzin	HGNC:115	TAZ removal co	Cell viability, cell deat	Human, mi	RCC4, 786	TAZ :+: EN	Tafazzin	Q16635	TAZ_HUM	31484063	ERAP2	endoplasmi	L-RAP|LR	5	Antigen_Processing_and_Presentation
MTDH	Metadherin	HGNC:296	Can enhance sens	Cell viability, qRT-PC	Human, mi	Cell lines A	MTDH :-: (	Protein LY	Q86UE4	LYRIC_HU	31527591	ULBP3	UL16 bindi	N2DL-3|NI	6	Antigen_Processing_and_Presentation
IDH1	Isocitrate dehydrogenase (NADP(+))	HGNC:538	Deletion of the m	Cell transfection, cell v	Human	HEK293T,	IDH1 :-: GI	Isocitrate d	O75874	IDHC_HUI	31591388	ULBP2	UL16 bindi	ALCAN-al	6	Antigen_Processing_and_Presentation
SIRT1	Sirtuin 1	HGNC:149	Knockout partial	Lipid peroxidation, iro	Mice	Mice in C5	SIRT1 :+: I	NAD-deper	Q96EB6	SIR1_HUM	31610175	ULBP1	UL16 bindi	N2DL-1|NI	6	Antigen_Processing_and_Presentation
TAZ	Tafazzin	HGNC:115	TAZ removal co	siRNA, qRT-PCR, wes	Human	TOV-21G,	TAZ :+: AI	Tafazzin	Q16635	TAZ_HUM	31641008	KIR3DL3	killer cell ir	CD158Z|KI	19	Antigen_Processing_and_Presentation
BECN1	Beclin 1	HGNC:103	Overexpression a	Cell viability, RNAi, R	Human	SH-SY5Y (	(Beclin1/SI	Beclin-1	Q14457	BECN1_HI	31650158	RAET1E	retinoic aci	LETAL|N2	6	Antigen_Processing_and_Presentation
FBXW7	F-box and WD repeat domain contain	HGNC:167	FBXW7 plasmid	Cell viability, lipid per	Human, mi	Hepatic ste	FBXW7 :+	F-box/WD	Q969H0	FBXW7_H	31679460	RAET1L	retinoic aci	ULBP6	6	Antigen_Processing_and_Presentation
PANX1	Pannexin 1	HGNC:859	Deletion protects	Cell viability, cell deat	Human, mi	HK-2 cells,	PANX1 :-:	Pannexin-1	Q96RD7	PANX1_HI	31694915	UBR1	ubiquitin pr	JBS	15	Antigen_Processing_and_Presentation
DNAJB6	DnaJ heat shock protein family (Hsp4	HGNC:148	Promotes ferropto	Western blot, immunol	Human, mi	Cell lines T	DNAJB6 :-	DnaJ homo	O75190	DNJB6_HU	31701262	RAET1G	retinoic aci	ULBP5	6	Antigen_Processing_and_Presentation
BACH1	BTB domain and CNC homolog 1	HGNC:935	Promotes ferropto	Western blotting, qPCI	Mice	C57BL/6J r	BACH1 :+:	Transcriptio	O14867	BACH1_H	31740582	PDIA2	protein disu	PDA2|PDI|	16	Antigen_Processing_and_Presentation
ACSL4	Acyl-CoA synthetase long chain fami	HGNC:357	Overexpression i	Western blotting, immu	Human	HEB, T98C	ACSL4 :-: (	Long-chain	O60488	ACSL4_HU	31789401	HAMP	hepcidin an	HEPC|HFE	19	Antimicrobials
LONP1	Lon peptidase 1, mitochondrial	HGNC:947	Inhibition of LON	Cell viability, western	Human	PANC1, Bx	LONP1 :-:	Lon proteas	P36776	LONM_HU	31822343	PI3	peptidase ir	ESI|SKALI	20	Antimicrobials
CD82	CD82 molecule	HGNC:621	This study identi	western blotting, cck-8	Human	PC cell line	KAI1 :+: Ferroptosis				33655331	CAMP	cathelicidin	CAP-18|CA	3	Antimicrobials
IL1B	interleukin 1 beta	HGNC:599	We observed that	_NA_	Human, mi	chondrocyt	IL1B :+: R(	_NA_	_NA_		33376672	DEFB4A	defensin be	BD-2|DEFI	8	Antimicrobials
CTSB	cathepsin B	HGNC:252	Cathepsin B is ar	_NA_	_NA_	_NA_	CTSB :+: F	_NA_	_NA_		33340545	PPBP	pro-platelet	B-TG1|Beta	4	Antimicrobials
POR	cytochrome p450 oxidoreductase	HGNC:920	The oxidoreducta	CRISPR-Cas9, immun	Human, mi	HeLa, HT-1	POR :+: hy	_NA_	_NA_		33321093	REG3G	regeneratin	LPPM429|I	2	Antimicrobials
CYB5R1	cytochrome b5 reductase 1	HGNC:133	The oxidoreducta	shRNA, gene expressio	Human	HeLa cells	CYB5R1 :+	_NA_	_NA_		33321093	CXCL14	C-X-C mot	BMAC|BR	5	Antimicrobials
ELOVL5	ELOVL fatty acid elongase 5	HGNC:213	Biochemical and	Cysteine/Methionine D	Human	YCC-16 ce	ELOVL5 :+	_NA_	_NA_		33288688	CXCL16	C-X-C mot	CXCLG16|	17	Antimicrobials
FADS1	fatty acid desaturase 1	HGNC:357	Biochemical and	Cysteine/Methionine D	Human	YCC-16 ce	FADS1 :+:	_NA_	_NA_		33288688	SLPI	secretory le	ALK1|ALP	20	Antimicrobials
ALOX12	arachidonate 12-lipoxygenase, 12S ty	HGNC:429	Hydrogen sulfide	Cell viability assay, M	Mice	myoblasts a	ALOX12 :+	_NA_	_NA_		33290842	CXCL8	C-X-C mot	GCP-1|GCI	4	Antimicrobials
FBW7	F-box and WD repeat domain contain	HGNC:167	Results revealed	Cell viability and cell c	Human	cell lines P	FBW7 :-: (I	_NA_	_NA_		33271455	CXCL10	C-X-C mot	C7|IFI10|IN	4	Antimicrobials
PTEN	phosphatase and tensin homolog	HGNC:958	Notably, we foun	Cell death	Human	BT474, MD	PTEN :-: PI	_NA_	_NA_		33229547	CXCL9	C-X-C mot	CMK|Humi	4	Antimicrobials
NR1D1	nuclear receptor subfamily 1 group D	HGNC:796	Targeted inhibitio	_NA_	Mice	_NA_	(NR1D1/R(	_NA_	_NA_		33068011	CXCL5	C-X-C mot	ENA-78|SC	4	Antimicrobials
NR1D2	nuclear receptor subfamily 1 group D	HGNC:796	Targeted inhibitio	_NA_	Mice	_NA_	(NR1D2/R(	_NA_	_NA_		33068011	CXCL11	C-X-C mot	H174|I-TA(	4	Antimicrobials
TBK1	TANK binding kinase 1	HGNC:115	hTBK1-c.978T>A	_NA_	Mice	_NA_	TBK1-c.97	_NA_	_NA_		33312375	CXCL6	C-X-C mot	CKA-3|GC	4	Antimicrobials
IL6	interleukin 6	HGNC:601	IL-6 exposure ca	_NA_	Human, mi	_NA_	IL-6 :-: miR	_NA_	_NA_		33166496	CXCL1	C-X-C mot	FSP|GRO1|	4	Antimicrobials
USP7	ubiquitin specific peptidase 7	HGNC:126	USP7 promotes f	_NA_	Rat	_NA_	USP7 :+: p	_NA_	_NA_		33157209	CXCL12	C-X-C mot	IRH|PBSF|S	10	Antimicrobials
miR-182-5p	_NA_	_NA_	In conclusion, we	_NA_	Human, rat	_NA_	miR-182-5p	_NA_	_NA_		33116120	CXCL13	C-X-C mot	ANGIE|AN	4	Antimicrobials
miR-378a-3	_NA_	_NA_	In conclusion, we	_NA_	Human, rat	_NA_	miR-378a-3	_NA_	_NA_		33116120	CXCL2	C-X-C mot	CINC-2a|G	4	Antimicrobials
CTSB	cathepsin B	HGNC:252	Mechanistically,	_NA_	Human, mi	_NA_	CTSB :+: D	_NA_	_NA_		33268027	PF4	platelet fact	CXCL4|PF-	4	Antimicrobials
ACSL4	acyl-CoA synthetase long chain famil	HGNC:357	Inhibition of ACS	_NA_	Human, mi	_NA_	ACSL4 :-: (	_NA_	_NA_		33070393	XCL1	X-C motif c	ATAC|LPT	1	Antimicrobials
ATF4	activating transcription factor 4	HGNC:786	We identify ATF	_NA_	Human, mi	_NA_	ATF4 :+: F	_NA_	_NA_		33055209	CXCL3	C-X-C mot	CINC-2b|G	4	Antimicrobials
BECN1	Beclin 1	HGNC:103	Beclin1 haploinsu	_NA_	Mice	_NA_	BECN1 :+:	_NA_	_NA_		33058849	DEFB103B	defensin be	BD-3|DEFI	8	Antimicrobials
AQP3	aquaporin 3 (Gill blood group)	HGNC:636	Further, we obser	_NA_	_NA_	_NA_	(AQP3/NO	_NA_	_NA_		33017631	CCL13	C-C motif c	CKb10|MC	17	Antimicrobials
AQP5	aquaporin 5	HGNC:638	Further, we obser	_NA_	_NA_	_NA_	(AQP5/NO	_NA_	_NA_		33017631	CCL1	C-C motif c	I-309|P500|	17	Antimicrobials
AQP8	aquaporin 8	HGNC:642	Further, we obser	_NA_	_NA_	_NA_	(AQP8/NO	_NA_	_NA_		33017631	DEFB1	defensin be	BD1|DEFB	8	Antimicrobials
LINC00618	long intergenic non-protein coding RN	HGNC:201	LINC00618 also	_NA_	Human, mi	_NA_	LINC00618	_NA_	_NA_		33002417	CCL8	C-C motif c	HC14|MCP	17	Antimicrobials
IREB2	iron responsive element binding prote	HGNC:611	Further studies h	_NA_	Human	_NA_	IRP2 :+: Fe	_NA_	_NA_		32947011	ELANE	elastase, ne	ELA2|GE|H	19	Antimicrobials
MT1DP	metallothionein 1D, pseudogene	HGNC:739	We found that ec	_NA_	Human	_NA_	MT1DP :+:	_NA_	_NA_		32929075	DEFB103A	defensin be	BD-3|DEFI	8	Antimicrobials
ACSL4	acyl-CoA synthetase long chain famil	HGNC:357	_NA_	_NA_	Human	_NA_	ACSL4 :+:	_NA_	_NA_		32939090	DEFA3	defensin alp	DEF3|HNP	8	Antimicrobials

PEX10	peroxisomal biogenesis factor 10	HGNC:885	Genome-wide CI	_NA_	Human, mi	_NA_	PEX10 :+:	_NA_	_NA_		32939090	DEFA1	defensin al	DEF1|DEF.	8	Antimicrobials
KEAP1	kelch like ECH associated protein 1	HGNC:231	_NA_	_NA_	Human	_NA_	KEAP1 :+:	_NA_	_NA_		32939090	TMSB10	thymosin b	MIG12|TB	2	Antimicrobials
AGPAT3	1-acylglycerol-3-phosphate O-acyltra	HGNC:326	_NA_	_NA_	Human	_NA_	AGPAT3 :-	_NA_	_NA_		32939090	DEFA6	defensin al	DEF6|HD-(	8	Antimicrobials
PEX12	peroxisomal biogenesis factor 12	HGNC:885	Genome-wide CI	_NA_	Human	_NA_	PEX12 :+:	_NA_	_NA_		32939090	DEFA5	defensin al	DEF5|HD-5	8	Antimicrobials
CHP1	calcineurin like EF-hand protein 1	HGNC:174	_NA_	_NA_	Human	_NA_	CHP1 :+: F	_NA_	_NA_		32939090	DEFA4	defensin al	DEF4|HNP	8	Antimicrobials
GPAT4	glycerol-3-phosphate acyltransferase	HGNC:208	_NA_	_NA_	Human	_NA_	AGPAT6 :-	_NA_	_NA_		32939090	LCN2	lipocalin 2	24p3|MSFI	9	Antimicrobials
BRPF1	bromodomain and PHD finger contai	HGNC:142	_NA_	_NA_	Human	_NA_	BRPF1 :+:	_NA_	_NA_		32939090	LCN1	lipocalin 1	PMFA|TLC	9	Antimicrobials
OSBPL9	oxysterol binding protein like 9	HGNC:163	_NA_	_NA_	Human	_NA_	OSBPL9 :+	_NA_	_NA_		32939090	COLEC10	collectin su	3MC3|CL-3	8	Antimicrobials
INTS2	integrator complex subunit 2	HGNC:292	_NA_	_NA_	Human	_NA_	INTS2 :+: I	_NA_	_NA_		32939090	BPI	bactericidal	BPIFD1|rB	20	Antimicrobials
MMD	monocyte to macrophage differentiati	HGNC:715	_NA_	_NA_	Human	_NA_	MMD :+: F	_NA_	_NA_		32939090	S100A9	S100 calciu	60B8AG|C.	1	Antimicrobials
CYP4F8	cytochrome P450 family 4 subfamily	HGNC:264	_NA_	_NA_	Human	_NA_	CYP4F8 :+	_NA_	_NA_		32939090	S100A8	S100 calciu	60B8AG|C.	1	Antimicrobials
MLLT1	MLLT1 super elongation complex su	HGNC:713	_NA_	_NA_	Human	_NA_	MLLT1 :+:	_NA_	_NA_		32939090	DCD	dermcidin	AIDD|DCD	12	Antimicrobials
TTPA	alpha tocopherol transfer protein	HGNC:124	_NA_	_NA_	Human	_NA_	TTPA :+: F	_NA_	_NA_		32939090	LCN6	lipocalin 6	LCN5|UNC	9	Antimicrobials
GRIA3	glutamate ionotropic receptor AMPA	HGNC:457	_NA_	_NA_	Human	_NA_	GRIA3 :+:	_NA_	_NA_		32939090	S100A12	S100 calciu	CAAF1|CA	1	Antimicrobials
EPT1	_NA_	_NA_	_NA_	_NA_	Human	_NA_	EPT1 :+: F	_NA_	_NA_		32939090	HTN3	histatin 3	HIS2|HTN	4	Antimicrobials
POM121L1	POM121 transmembrane nucleoporin	HGNC:253	_NA_	_NA_	Human	_NA_	POM121L1	_NA_	_NA_		32939090	LCN8	lipocalin 8	EP17|LCN5	9	Antimicrobials
LIG3	DNA ligase 3	HGNC:660	_NA_	_NA_	Human, mi	_NA_	LIG3 :+: F	_NA_	_NA_		32939090	DEFA1B	defensin al	HNP-1|HP-	8	Antimicrobials
AEBP2	AE binding protein 2	HGNC:240	_NA_	_NA_	Human	_NA_	AEBP2 :+:	_NA_	_NA_		32939090	CCR10	C-C motif c	GPR2	17	Antimicrobials
AGPS	alkylglycerone phosphate synthase	HGNC:327	Genome-wide CI	_NA_	Human	_NA_	AGPS :+: F	_NA_	_NA_		32939090	CELA1	chymotryps	ELA1	12	Antimicrobials
CDCA3	cell division cycle associated 3	HGNC:146	_NA_	_NA_	Human	_NA_	CDCA3 :+:	_NA_	_NA_		32939090	DEFB106A	defensin be	BD-6|DEFI	8	Antimicrobials
PEX2	peroxisomal biogenesis factor 2	HGNC:971	Genome-wide CI	_NA_	Human	_NA_	PEX2 :+: P	_NA_	_NA_		32939090	PENK	proenkepha	PE|PENK-A	8	Antimicrobials
LPCAT3	lysophosphatidylcholine acyltransfera	HGNC:302	_NA_	_NA_	Human	_NA_	LPCAT3 :+	_NA_	_NA_		32939090	BPIFC	BPI fold co	BPIL2	22	Antimicrobials
PEX6	peroxisomal biogenesis factor 6	HGNC:885	_NA_	_NA_	Human	_NA_	PEX6 :+: F	_NA_	_NA_		32939090	MMP12	matrix meta	HME|ME|M	11	Antimicrobials
TIMM9	translocase of inner mitochondrial me	HGNC:118	_NA_	_NA_	Human	_NA_	TIMM9 :+:	_NA_	_NA_		32939090	BPIFB6	BPI fold co	BPIL3|LPL	20	Antimicrobials
DCAF7	DDB1 and CUL4 associated factor 7	HGNC:309	_NA_	_NA_	Human	_NA_	DCAF7 :+:	_NA_	_NA_		32939090	LEAP2	liver enrich	LEAP-2	5	Antimicrobials
LCE2C	late cornified envelope 2C	HGNC:294	_NA_	_NA_	Human	_NA_	LCE2C :+:	_NA_	_NA_		32939090	SFTPD	surfactant p	COLEC7|P	10	Antimicrobials
FAR1	fatty acyl-CoA reductase 1	HGNC:262	Genome-wide CI	_NA_	Human	_NA_	FAR1 :+: P	_NA_	_NA_		32939090	LCN9	lipocalin 9	HEL129	9	Antimicrobials
PHF21A	PHD finger protein 21A	HGNC:241	_NA_	_NA_	Human	_NA_	PHF21A :+	_NA_	_NA_		32939090	BPIFB2	BPI fold co	BPIL1|C20	20	Antimicrobials
SMAD7	SMAD family member 7	HGNC:677	_NA_	_NA_	Human	_NA_	SMAD7 :+	_NA_	_NA_		32939090	PTGDS	prostagland	L-PGDS|LI	9	Antimicrobials
LYRM1	LYR motif containing 1	HGNC:250	_NA_	_NA_	Human	_NA_	LYRM1 :+	_NA_	_NA_		32939090	TMSB4X	thymosin b	FX|PTMB4	X	Antimicrobials
AMN	amnion associated transmembrane pr	HGNC:146	_NA_	_NA_	Human	_NA_	AMN :+: F	_NA_	_NA_		32939090	PGLYRP1	peptidoglyc	PGLYRP|P	19	Antimicrobials
PEX3	peroxisomal biogenesis factor 3	HGNC:885	Genome-wide CI	_NA_	Human	_NA_	PEX3 :+: P	_NA_	_NA_		32939090	ZC3HAV1	zinc finger	ARTD13|F.	7	Antimicrobials
MTCH1	mitochondrial carrier 1	HGNC:175	_NA_	_NA_	Human	_NA_	MTCH1 :+	_NA_	_NA_		32939090	TMSB15A	thymosin b	TMSB15|T	X	Antimicrobials
ZEB1	zinc finger E-box binding homeobox	HGNC:116	CDH1 silencing	_NA_	Human, mi	_NA_	ZEB1 :+: F	_NA_	_NA_		32896720	S100B	S100 calciu	NEF|S100|S	21	Antimicrobials
SIRT1	sirtuin 1	HGNC:149	Histone deacetyl	_NA_	Human, mi	_NA_	SIRT1 :+: I	_NA_	_NA_		32896720	S100A13	S100 calciu	-	1	Antimicrobials
ACADSB	acyl-CoA dehydrogenase short/branc	HGNC:91	Overexpression c	_NA_	Human	_NA_	ACADSB :	_NA_	_NA_		32776663	S100A6	S100 calciu	2A9|5B10|C	1	Antimicrobials
PVT1	Pvt1 oncogene	HGNC:970	PVT1 silencing c	_NA_	Human, mi	_NA_	PVT1 :+: F	_NA_	_NA_		32827544	DEFB119	defensin be	DEFB-19|D	20	Antimicrobials
hsa_circ_0(	_NA_	_NA_	These evidences	_NA_	Human	_NA_	hsa_circ_0(	_NA_	_NA_		32802409	DEFB107A	defensin be	BD-7|DEFI	8	Antimicrobials
SLC39A14	solute carrier family 39 member 14	HGNC:208	Loss of hepatic S	_NA_	Mice	_NA_	SLC39A14	_NA_	_NA_		32374849	DEFB105A	defensin be	BD-5|DEFI	8	Antimicrobials
NCOA4	nuclear receptor coactivator 4	HGNC:767	Additionally, NC	_NA_	Human	_NA_	NCOA4 :-:	Nuclear rec	Q13772	NCOA4_H	32810738	SERPIND1	serpin fami	D22S673|H	22	Antimicrobials
MAP3K11	mitogen-activated protein kinase kina	HGNC:685	Inhibition of ML	_NA_	Mice	_NA_	MAP3K11	Mitogen-ac	Q16584	M3K11_HU	32710001	DEFB129	defensin be	C20orf87|D	20	Antimicrobials
GSK3B	glycogen synthase kinase 3 beta	HGNC:461	Silence of GSK-3	_NA_	Human	_NA_	GSK3B :+:	Glycogen s	P49841	GSK3B_HU	32642794	DEFB127	defensin be	C20orf73|D	20	Antimicrobials
MAPK8	mitogen-activated protein kinase 8	HGNC:688	Knockdown of JN	_NA_	Human	_NA_	MAPK8 :+	Mitogen-ac	P45983	MK08_HU	32782585	S100P	S100 calciu	MIG9	4	Antimicrobials
BRD7	bromodomain containing 7	HGNC:143	Upregulated BRI	_NA_	Human, mi	_NA_	BRD7 :+: I	Bromodom	Q9NPI1	BRD7_HU	32863216	S100A7	S100 calciu	PSOR1|S10	1	Antimicrobials
TP53	tumor protein p53	HGNC:119	Additionally, p53	_NA_	Human, mi	_NA_	TP53 :+: Li	Cellular tur	P04637	P53_HUM	32863216	DEFB104A	defensin be	BD-4|DEFI	8	Antimicrobials
SLC25A28	solute carrier family 25 member 28	HGNC:234	As expected, shR	_NA_	Human, mi	_NA_	SLC25A28	Mitoferrin-	Q96A46	MFRN2_H	32863216	DEFB126	defensin be	C20orf8|DI	20	Antimicrobials
ACSL4	acyl-CoA synthetase long chain famil	HGNC:357	Inhibition of ACS	_NA_	Rat	_NA_	ACSL4 :+:	Long-chain	O60488	ACSL4_HU	32593899	DEFB106B	defensin be	BD-6|DEFI	8	Antimicrobials
MFN2	mitofusin 2	HGNC:168	Thus, Mfn2 appe	_NA_	Rat	_NA_	MFN2 :+: 5	Mitofusin-2	O95140	MFN2_HU	32593899	DEFB104B	defensin be	BD-4|DEFI	8	Antimicrobials
ACSL4	acyl-CoA synthetase long chain famil	HGNC:357	Knockdown of A	_NA_	Human	_NA_	ACSL4 :+:	Long-chain	O60488	ACSL4_HU	32541921	DEFB107B	defensin be	HsT21816	8	Antimicrobials
SLC11A2	solute carrier family 11 member 2	HGNC:109	Overexpression c	_NA_	Human	_NA_	SLC11A2 :	Natural resi	P49281	NRAM2_H	32535745	PGLYRP3	peptidoglyc	PGLYRPIa	1	Antimicrobials
ZFAS1	ZNFX1 antisense RNA 1	HGNC:331	lncRNA ZFAS1	_NA_	Human, rat	_NA_	ZFAS1 :+:	_NA_	_NA_	_NA_	32453709	PGLYRP2	peptidoglyc	HMFT0141	19	Antimicrobials
SLC38A1	solute carrier family 38 member 1	HGNC:134	Mechanistically,	_NA_	Human, rat	_NA_	SLC38A1 :	Sodium-co	Q9H2H9	S38A1_HU	32453709	S100A10	S100 calciu	42C|ANX2	1	Antimicrobials
TSC1	TSC complex subunit 1	HGNC:123	Haploinsufficien	_NA_	Human, mi	_NA_	TSC1 :+: F	Hamartin	Q92574	TSC1_HUN	32404875	S100A2	S100 calciu	CAN19|S1(	1	Antimicrobials
PEBP1	phosphatidylethanolamine binding pr	HGNC:863	We found that PI	_NA_	Human	_NA_	PEBP1 :+:	Phosphatid	P30086	PEBP1_HU	32513718	DEFB125	defensin be	DEFB-25	20	Antimicrobials
TGFB1	transforming growth factor beta 1	HGNC:117	Therefore, PLC/I	_NA_	Human	_NA_	TGFB1 :-:	Transformi	P01137	TGFB1_HU	32471991	DEFB123	defensin be	DEFB-23|D	20	Antimicrobials
SNCA	synuclein alpha	HGNC:111	Alpha synuclein	_NA_	Human	_NA_	SNCA :+: I	Alpha-synu	P37840	SYUA_HU	32341450	DEFB105B	defensin be	BD-5|DEFI	8	Antimicrobials
SIRT3	sirtuin 3	HGNC:149	SIRT3 deficiency	_NA_	Human, po	_NA_	SIRT3 :-: C	NAD-deper	Q9NTG7	SIR3_HUM	32329068	DEFB132	defensin be	BD-32|DEF	20	Antimicrobials
PRKAA2	protein kinase AMP-activated catalyti	HGNC:937	Suppression of A	_NA_	Human, po	_NA_	PRKAA2 :	5'-AMP-act	P54646	AAPK2_HU	32329068	BPIFB3	BPI fold co	C20orf185|	20	Antimicrobials
TFRC	transferrin receptor	HGNC:117	Knockdown of T	_NA_	Human	_NA_	TFRC :+: F	Transferrin	P02786	TFR1_HUN	32283255	LCN12	lipocalin 12	-	9	Antimicrobials
CGAS	cyclic GMP-AMP synthase	HGNC:213	The CGAS-STIN	_NA_	Human	_NA_	CGAS :+: N	Cyclic GM	Q8N884	CGAS_HU	32186434	PGLYRP4	peptidoglyc	PGLYRPIb	1	Antimicrobials

STING1	stimulator of interferon response cGA	HGNC:279	The CGAS-STIN	_NA_	Human	_NA_	STING1 :+	Stimulator	Q86WV6	STING_HU	32186434
HDDC3	HD domain containing 3	HGNC:305	Ferroptosis-induc	_NA_	Human	_NA_	HDDC3 :-:	Guanosine-	Q8N4P3	MESH1_H	32462112
MIR761	microRNA 761	HGNC:373	Additionally, circ	_NA_	Human	_NA_	MIR761 :-:	_NA_	_NA_	_NA_	32196629
MDM2	MDM2 proto-oncogene	HGNC:697	MDM2 and MDI	_NA_	Human	_NA_	MDM2 :+:	E3 ubiquiti	Q00987	MDM2_HU	32079652
MDM4	MDM4 regulator of p53	HGNC:697	MDM2 and MDI	_NA_	Human	_NA_	MDM4 :+:	Protein Md	O15151	MDM4_HU	32079652
ALOX15	arachidonate 15-lipoxygenase	HGNC:433	Indeed, knocking	_NA_	Mice	_NA_	ALOX15 :+	Polyunsatu	P16050	LOX15_HU	32080625
POR	cytochrome p450 oxidoreductase	HGNC:920	These data indica	_NA_	Human	_NA_	POR :+: Li	NADPH--c	P16435	NCPR_HU	32080622
MIR214	microRNA 214	HGNC:315	MicroRNA-214-	_NA_	Human	_NA_	miR-214 :+	_NA_	_NA_	_NA_	31960438
DLD	dihydrolipoamide dehydrogenase	HGNC:289	DLD links to ferr	_NA_	Human	_NA_	DLD :+: Li	Dihydrolipo	P09622	DLDH_HU	31931284
LONP1	lon peptidase 1, mitochondrial	HGNC:947	Inhibition of LON	_NA_	Human	_NA_	LONP1 :-:	Lon proteas	P36776	LONM_HU	31822343
ACSL4	acyl-CoA synthetase long chain famil	HGNC:357	ACSL4 suppresse	_NA_	Human	_NA_	ACSL4 :-:	Long-chain	O60488	ACSL4_HU	31789401
BACH1	BTB domain and CNC homolog 1	HGNC:935	These results sho	_NA_	Mice	_NA_	BACH1 :-:	Transcripti	O14867	BACH1_H	31740582
DNAJB6	DnaJ heat shock protein family (Hsp4	HGNC:148	DNAJB6 Promot	_NA_	Human	_NA_	DNAJB6a :	DnaJ homo	O75190	DNJB6_HU	31701262
WWTR1	WW domain containing transcription	HGNC:240	TAZ removal cor	_NA_	Human	_NA_	WWTR1 :+	WW domai	Q9GZV5	WWTR1_H	31641008
SIRT1	sirtuin 1	HGNC:149	Intestinal SIRT1	_NA_	Mice	_NA_	SIRT1 :+: I	NAD-deper	Q96EB6	SIR1_HUM	31610175
ATM	ATM serine/threonine kinase	HGNC:795	Taken together, v	_NA_	Human	_NA_	ATM :-: FP	Serine-prot	Q13315	ATM_HUM	31320750
PRKCA	protein kinase C alpha	HGNC:939	Similarly, geneti	_NA_	Human	_NA_	PRKCA :+:	Protein kin	P17252	KPCA_HU	31173656

S100A11	S100 calciu	HEL-S-43|I		1	Antimicrobials
S100A5	S100 calciu	S100D		1	Antimicrobials
S100A3	S100 calciu	S100E		1	Antimicrobials
S100A1	S100 calciu	S100|S100-		1	Antimicrobials
DEFB128	defensin be	DEFB-28|D		20	Antimicrobials
DEFB108B	defensin be	DEFB-8|hB		11	Antimicrobials
HTN1	histatin 1	HIS1		4	Antimicrobials
LMBR1L	limb develo	LIMR		12	Antimicrobials
S100A7A	S100 calciu	NICE-2|NI		1	Antimicrobials
DEFB118	defensin be	C20orf63|D		20	Antimicrobials
COLEC12	collectin su	CLP1|NSR		18	Antimicrobials
TMSB4Y	thymosin be	TB4Y	Y		Antimicrobials
DEFB131A	defensin be	DEFB-31|D		4	Antimicrobials
DEFB134	defensin be	-		8	Antimicrobials
DEFB130A	defensin be	DEFB-30|D		8	Antimicrobials
DEFB124	defensin be	DEFB-24		20	Antimicrobials
DEFB121	defensin be	DEFB21|ES		20	Antimicrobials
DEFB116	defensin be	DEFB-16		20	Antimicrobials
DEFB115	defensin be	DEFB-15		20	Antimicrobials
DEFB114	defensin be	DEFB-14|D		6	Antimicrobials
DEFB113	defensin be	DEFB-13		6	Antimicrobials
DEFB112	defensin be	DEFB-12		6	Antimicrobials
DEFB110	defensin be	DEFB-10|D		6	Antimicrobials
TMSB15B	thymosin be	TMSB15A|	X		Antimicrobials
DEFB133	defensin be	-		6	Antimicrobials
S100Z	S100 calciu	Gm625|S10		5	Antimicrobials
MAVS	mitochondr	CARDIF|IF		20	Antimicrobials
TMSB4XP	TMSB4X p	TMSL3		4	Antimicrobials
S100A14	S100 calciu	BCMP84|S		1	Antimicrobials
LCN10	lipocalin 10	-		9	Antimicrobials
S100A16	S100 calciu	AAG13|DT		1	Antimicrobials
DEFB136	defensin be	DEFB137		8	Antimicrobials
DEFB135	defensin be	DEFB136		8	Antimicrobials
DEFB117	defensin be	DEFB-17		20	Antimicrobials
DEFB110	defensin be	DEFB-10|D		6	Antimicrobials
ZC3HAV1L	zinc finger	C7orf39		7	Antimicrobials
S100A7L2	S100 calciu	S100a7b		1	Antimicrobials
MBL3P	mannose-bi	COLEC2|M		10	Antimicrobials
DEFB4B	defensin be	DEFB4P		8	Antimicrobials
BPIFB4	BPI fold co	C20orf186|		20	Antimicrobials
IFNAR1	interferon a	AVP|IFN-a		21	Antimicrobials
AZU1	azurocidin	AZAMP|AZ		19	Antimicrobials
DEFB131B	defensin be	-		11	Antimicrobials
DEFA1A3	defensin alp	DEFA1|DE		8	Antimicrobials
LCN1P1	lipocalin 1 p	LCN1L1|bA		9	Antimicrobials
S100G	S100 calciu	CABP|CAE	X		Antimicrobials
DEFA7P	defensin alp	DEFA7		8	Antimicrobials
DEFB130B	defensin be	-		8	Antimicrobials
DEFB108F	defensin be	DEFB108P		4	Antimicrobials
DEFB131C	defensin be	-		8	Antimicrobials
TCHHL1	trichohyalir	S100A17|T		1	Antimicrobials
TINAGL1	tubulointers	ARG1|LCN		1	Antimicrobials
IFNGR1	interferon g	CD119|IFN		6	Antimicrobials
SLC22A17	solute carri	24p3R|BOC		14	Antimicrobials
WFIKKN1	WAP, follis	C16orf12|R		16	Antimicrobials
WFDC2	WAP four-	EDDM4|HE		20	Antimicrobials
IL6	interleukin	BSF-2|BSF		7	Antimicrobials
UMODL1	uromodulin	-		21	Antimicrobials
TGFB1	transformin	CED|DPD1		19	Antimicrobials
PF4V1	platelet fact	CXCL4L1|		4	Antimicrobials

MMP9	matrix meta	CLG4B|GE	20	Antimicrobials
ANOS1	anosmin 1	ADMLX|H	X	Antimicrobials
TLR4	toll like rec	ARMD10|C	9	Antimicrobials
IFNG	interferon g	IFG|IFI	12	Antimicrobials
SPAG11B	sperm asso	EDDM2B|I	8	Antimicrobials
A2M	alpha-2-ma	A2MD|CPA	12	Antimicrobials
CTSL	cathepsin L	CATL|CTS	9	Antimicrobials
NFKB1	nuclear fact	CVID12|EI	4	Antimicrobials
APOBEC3	apolipoprot	A3G|ARCI	22	Antimicrobials
FABP6	fatty acid b	I-15P|I-BA	5	Antimicrobials
NOD2	nucleotide	ACUG|BLA	16	Antimicrobials
MBL2	mannose bi	COLEC1|H	10	Antimicrobials
SFTPA1	surfactant p	COLEC4|P	10	Antimicrobials
RBP1	retinol bind	CRABP-I|C	3	Antimicrobials
TLR2	toll like rec	CD282|TIL	4	Antimicrobials
SLC40A1	solute carri	FPN1|HFE4	2	Antimicrobials
PLAU	plasminoge	ATF|BDPL	10	Antimicrobials
IL1B	interleukin	IL-1|IL1-BI	2	Antimicrobials
PAEP	progestager	GD|GdA|G	9	Antimicrobials
HJV	hemojuveli	HFE2|HFE	1	Antimicrobials
MUC5AC	mucin 5AC	MUC5|TBM	11	Antimicrobials
CTSS	cathepsin S	-	1	Antimicrobials
OBP2A	odorant bin	LCN13|OB	9	Antimicrobials
PLTP	phospholipi	BPIFE|HDI	20	Antimicrobials
MX1	MX dynam	IFI-78K|IFI	21	Antimicrobials
DDX58	DExD/H-bo	RIG-I|RIG1	9	Antimicrobials
IFNL1	interferon l	IL-29|IL29	19	Antimicrobials
IRF3	interferon r	IIAE7	19	Antimicrobials
SFTPA2	surfactant p	COLEC5|P	10	Antimicrobials
LPA	lipoprotein(	AK38|APO	6	Antimicrobials
LBP	lipopolysac	BPIFD2	20	Antimicrobials
RBP4	retinol bind	MCOPCB1	10	Antimicrobials
SFTPA1	surfactant p	COLEC4|P	10	Antimicrobials
NOX4	NADPH ox	KOX|KOX	11	Antimicrobials
LTF	lactotransfe	GIG12|HEI	3	Antimicrobials
IFNB1	interferon b	IFB|IFF|IFN	9	Antimicrobials
RBP5	retinol bind	CRBP-III|C	12	Antimicrobials
FABP7	fatty acid b	B-FABP|BI	6	Antimicrobials
FABP5	fatty acid b	E-FABP|EI	8	Antimicrobials
FABP3	fatty acid b	FABP11|H-	1	Antimicrobials
FABP2	fatty acid b	FABPI|I-FA	4	Antimicrobials
FABP4	fatty acid b	A-FABP|A	8	Antimicrobials
R3HDML	R3H domai	dJ881L22.3	20	Antimicrobials
BPIFA3	BPI fold co	C20orf71|S	20	Antimicrobials
BPIFB1	BPI fold co	C20orf114|	20	Antimicrobials
OASL	2'-5'-oligoa	OASL1|OA	12	Antimicrobials
CRABP2	cellular reti	CRABP-II|	1	Antimicrobials
CRABP1	cellular reti	CRABP|CR	15	Antimicrobials
RBP7	retinol bind	CRABP4|C	1	Antimicrobials
DUOX1	dual oxidas	LNOX1|NC	15	Antimicrobials
OBP2B	odorant bin	LCN14|OB	9	Antimicrobials
RBP2	retinol bind	CRABP-II|	3	Antimicrobials
LCN15	lipocalin 15	PRO6093|U	9	Antimicrobials
CETP	cholesteryl	BPIFF|HDI	16	Antimicrobials
FABP12	fatty acid b	-	8	Antimicrobials
FABP9	fatty acid b	PERF|PERI	8	Antimicrobials
BPIFA1	BPI fold co	LUNX|NAS	20	Antimicrobials
LCNL1	lipocalin lik	-	9	Antimicrobials
C8G	complemen	C8C	9	Antimicrobials
SPAG11A	sperm asso	EDDM2A|I	8	Antimicrobials

PI15	peptidase ir	CRISP8|P2	8	Antimicrobials
NOX1	NADPH ox	GP91-2|MC	X	Antimicrobials
PMP2	peripheral r	CMT1G|FA	8	Antimicrobials
APOD	apolipoprot	-	3	Antimicrobials
ORM2	orosomucoi	AGP-B|AG	9	Antimicrobials
ORM1	orosomucoi	AGP-A|AG	9	Antimicrobials
TNF	tumor necro	DIF|TNF-a	6	Antimicrobials
CTSG	cathepsin G	CATG|CG	14	Antimicrobials
PRTN3	proteinase 3	ACPA|AGI	19	Antimicrobials
MAPK1	mitogen-act	ERK|ERK-	22	Antimicrobials
PML	PML nucle	MYL|PP86	15	Antimicrobials
AEN	apoptosis e	ISG20L1|p	15	Antimicrobials
CYBB	cytochrome	AMCBX2|C	X	Antimicrobials
BPIFA2	BPI fold co	C20orf70|P	20	Antimicrobials
ISG20	interferon s	CD25|HEM	15	Antimicrobials
BCL3	BCL3 trans	BCL4|D19S	19	Antimicrobials
ISG20L2	interferon s	HSD38	1	Antimicrobials
NOX5	NADPH ox	-	15	Antimicrobials
NOX3	NADPH ox	GP91-3|MC	6	Antimicrobials
DUOX2	dual oxidas	LNOX2|NC	15	Antimicrobials
TLR3	toll like rec	CD283|IIA	4	Antimicrobials
TFRC	transferrin r	CD71|IMD	3	Antimicrobials
IFIH1	interferon i	AGS7|Hlcd	2	Antimicrobials
LRP1	LDL recept	A2MR|APC	12	Antimicrobials
TRIM5	tripartite m	RNF88|TRI	11	Antimicrobials
IDO1	indoleamin	IDO|IDO-1	8	Antimicrobials
GDF15	growth diff	GDF-15|MI	19	Antimicrobials
NEDD4	NEDD4 E3	NEDD4-1|I	15	Antimicrobials
ADIPOQ	adiponectin	ACDC|ACI	3	Antimicrobials
STAT3	signal trans	ADMIO|AI	17	Antimicrobials
STAT1	signal trans	CANDF7|II	2	Antimicrobials
IFNL2	interferon la	IL-28A|IL2	19	Antimicrobials
SOCS3	suppressor	ATOD4|CI	17	Antimicrobials
SEMG1	semenogeli	CT103|SEM	20	Antimicrobials
TNFSF10	TNF superf	APO2L|Ap	3	Antimicrobials
CCL20	C-C motif c	CKb4|Exod	2	Antimicrobials
SOCS1	suppressor	CIS1|CISH	16	Antimicrobials
RNASEL	ribonucleas	PRCA1|RN	1	Antimicrobials
IRF1	interferon r	IRF-1|MAR	5	Antimicrobials
IL15	interleukin	IL-15	4	Antimicrobials
APOBEC3	apolipoprot	A3F|ARP8|	22	Antimicrobials
PLAAT4	phospholipa	HRASLS4|	11	Antimicrobials
CHIT1	chitinase 1	CHI3|CHIT	1	Antimicrobials
IFNA1	interferon a	IFL|IFN|IFI	9	Antimicrobials
CD40	CD40 mole	Bp50|CDW	20	Antimicrobials
TLR7	toll like rec	TLR7-like	X	Antimicrobials
PPIA	peptidylpro	CYPA|CYF	7	Antimicrobials
HFE	homeostatic	HFE1|HH|H	6	Antimicrobials
ZYX	zyxin	ESP-2|HED	7	Antimicrobials
NLRX1	NLR family	CLR11.3|D	11	Antimicrobials
PGC	progastricsi	PEPC|PGII	6	Antimicrobials
VEGFA	vascular en	MVCD1|VI	6	Antimicrobials
IKBKE	inhibitor of	IKK-E|IKK	1	Antimicrobials
ISG15	ISG15 ubiq	G1P2|IFI15	1	Antimicrobials
DHX58	DExH-box	D11LGP2|I	17	Antimicrobials
TNFAIP3	TNF alpha	A20|AISBI	6	Antimicrobials
TFR2	transferrin r	HFE3|TFR	7	Antimicrobials
FCN2	ficolin 2	EBP-37|FC	9	Antimicrobials
MUC4	mucin 4, ce	ASGP|HSA	3	Antimicrobials
F2R	coagulation	CF2R|HTR	5	Antimicrobials

ELN	elastin	ADCL1|SV	7	Antimicrobials
IL27	interleukin	IL-27|IL-27	16	Antimicrobials
MAPT	microtubul	DDPAC|FT	17	Antimicrobials
LYZ	lysozyme	LYZF1|LZ	12	Antimicrobials
CCL5	C-C motif	D17S136E|	17	Antimicrobials
LEP	leptin	LEPD|OB|	7	Antimicrobials
CYLD	CYLD lysi	BRSS|CDM	16	Antimicrobials
KLKB1	kallikrein B	KLK3|PKK	4	Antimicrobials
CST4	cystatin S	-	20	Antimicrobials
CSRP1	cysteine an	CRP|CRP1|	1	Antimicrobials
MAPK14	mitogen-ac	CSBP|CSB	6	Antimicrobials
JUN	Jun proto-o	AP-1|AP1|c	1	Antimicrobials
ITGAV	integrin sub	CD51|MSK	2	Antimicrobials
IRF5	interferon r	SLEB10	7	Antimicrobials
CCR6	C-C motif	BN-1|C-C	6	Antimicrobials
IL12B	interleukin	CLMF|CL	5	Antimicrobials
TLR8	toll like rec	CD288	X	Antimicrobials
GNLY	granulysin	D2S69E|L	2	Antimicrobials
CD81	CD81 mole	CVID6|S5.	11	Antimicrobials
EIF2AK2	eukaryotic	EIF2AK1|L	2	Antimicrobials
APOM	apolipoprot	G3a|HSPC	6	Antimicrobials
CACYBP	calcyclin bi	GIG5|PNA	1	Antimicrobials
NOD1	nucleotide	CARD4|CL	7	Antimicrobials
MAPK8	mitogen-ac	JNK|JNK-4	10	Antimicrobials
MAPK3	mitogen-ac	ERK-1|ER	16	Antimicrobials
BST2	bone marro	CD317|TE	19	Antimicrobials
BPHL	biphenyl hy	BPH-RP|M	6	Antimicrobials
PLA2G2A	phospholip	MOM1|PL	1	Antimicrobials
GRN	granulin pr	CLN11|GE	17	Antimicrobials
NEWENTF	-	-	-	Antimicrobials
PDGFRA	platelet der	CD140A|P	4	Antimicrobials
GNAI1	G protein s	Gi	7	Antimicrobials
WNT5A	Wnt family	hWNT5A	3	Antimicrobials
FURIN	furin, paire	FUR|PACE	15	Antimicrobials
ADAR	adenosine d	ADAR1|A	1	Antimicrobials
TYK2	tyrosine kin	IMD35|JT	19	Antimicrobials
NOS2	nitric oxide	HEP-NOS|	17	Antimicrobials
TRAF3	TNF recept	CAP-1|CA	14	Antimicrobials
TPT1	tumor prote	HRF|TCTP	13	Antimicrobials
TPM2	tropomyosi	AMCD1|D	9	Antimicrobials
NEO1	neogenin 1	IGDCC2|N	15	Antimicrobials
AHNAK	AHNAK nu	AHNAKRS	11	Antimicrobials
TLR1	toll like rec	CD281|TIL	4	Antimicrobials
TK2	thymidine k	MTDPS2|M	16	Antimicrobials
PRDX2	peroxiredo	HEL-S-2a|	19	Antimicrobials
MX2	MX dynam	MXB	21	Antimicrobials
FGF2	fibroblast g	BFGF|FGF	4	Antimicrobials
FGA	fibrinogen a	Fib2	4	Antimicrobials
TCF7L2	transcriptio	TCF-4|TCF	10	Antimicrobials
F2RL1	F2R like try	GPR11|PA	5	Antimicrobials
TKFC	triokinase a	DAK|NET	11	Antimicrobials
MSR1	macrophag	CD204|SC	8	Antimicrobials
NFKBIZ	NFKB inhi	IKBZ|INA	3	Antimicrobials
LMBR1	limb develo	ACHP|C7o	7	Antimicrobials
EPPIN	epididymal	CT71|CT72	20	Antimicrobials
SRC	SRC proto-	ASV|SRC1	20	Antimicrobials
MPO	myeloperox	-	17	Antimicrobials
ELAVL1	ELAV like	ELAV1|HU	19	Antimicrobials
ROBO3	roundabout	HGPPS|HG	11	Antimicrobials
SP1	Sp1 transcr	-	12	Antimicrobials

SOD1	superoxide	ALS|ALS1|	21	Antimicrobials
PDF	peptide defo	-	16	Antimicrobials
DLL4	delta like ca	AOS6|delta	15	Antimicrobials
ECD	ecdysoneles	GCR2|HSG	10	Antimicrobials
SLC11A1	solute carri	LSH|NRAM	2	Antimicrobials
DMBT1	deleted in m	GP340|SAG	10	Antimicrobials
STING1	stimulator o	ERIS|MITA	5	Antimicrobials
SKIV2L	Ski2 like R	170A|DDX	6	Antimicrobials
SEMG2	semenogeli	SGII	20	Antimicrobials
LTA	lymphotoxi	LT|TNFB|T	6	Antimicrobials
DES	desmin	CDCD3|CS	2	Antimicrobials
DCK	deoxycytidi	-	4	Antimicrobials
DAXX	death doma	BING2|DA	6	Antimicrobials
TNFRSF10	TNF recept	APO2|CD2	8	Antimicrobials
TNFRSF10	TNF recept	CD262|DR	8	Antimicrobials
EED	embryonic	COGIS|HE	11	Antimicrobials
CCL4	C-C motif c	ACT2|AT7	17	Antimicrobials
LIMS1	LIM zinc fi	PINCH|PIN	2	Antimicrobials
LALBA	lactalbumin	LYZG	12	Antimicrobials
APOBEC3	apolipoprot	A3H|ARP-	22	Antimicrobials
TMPRSS6	transmembr	IRIDA|MT	22	Antimicrobials
SPINK5	serine pepti	LEKTI|LE	5	Antimicrobials
MARCO	macrophage	SCARA2|S	2	Antimicrobials
BECN1	beclin 1	ATG6|VPS	17	Antimicrobials
TNFSF11	TNF superf	CD254|OD	13	Antimicrobials
KNG1	kininogen 1	BDK|BK|H	3	Antimicrobials
CSK	C-terminal	-	15	Antimicrobials
KLRK1	killer cell le	CD314|D12	12	Antimicrobials
KCNH2	potassium v	ERG-1|ERG	7	Antimicrobials
JUND	JunD proto-	AP-1	19	Antimicrobials
JAK1	Janus kinas	JAK1A|JAI	1	Antimicrobials
CREB1	cAMP resp	CREB|CRE	2	Antimicrobials
CLDN4	claudin 4	CPE-R|CPE	7	Antimicrobials
CCL28	C-C motif c	CCK1|MEC	5	Antimicrobials
RNASE3	ribonucleas	ECP|RAF1|	14	Antimicrobials
RN7SL1	RNA comp	7L1a|7SL|R	14	Antimicrobials
IRF7	interferon r	IMD39|IRF	11	Antimicrobials
IREB2	iron respon	ACO3|IRE-	15	Antimicrobials
ILK	integrin linl	HEL-S-28|I	11	Antimicrobials
IL18	interleukin	IGIF|IL-18|	11	Antimicrobials
IL17A	interleukin	CTLA-8|CT	6	Antimicrobials
LTB4R	leukotriene	BLT1|BLT	14	Antimicrobials
APOBEC3	apolipoprot	A3A|ARP3	22	Antimicrobials
MASP2	mannan bin	MAP19|MA	1	Antimicrobials
TRIM27	tripartite m	RFP|RNF7	6	Antimicrobials
RELA	RELA proto	CMCU|NFI	11	Antimicrobials
IL7R	interleukin	CD127|CD	5	Antimicrobials
IL1A	interleukin	IL-1 alpha|I	2	Antimicrobials
PTX3	pentraxin 3	TNFAIP5|T	3	Antimicrobials
IFNAR2	interferon a	IFN-R|IFN-	21	Antimicrobials
IFN1@	-	IFNA	9	Antimicrobials
SYTL1	synaptotagn	JFC1|SLP1	1	Antimicrobials
APOBEC3	apolipoprot	A3C|APOB	22	Antimicrobials
DDX17	DEAD-box	P72|RH70	22	Antimicrobials
PTGS2	prostagland	COX-2|CO	1	Antimicrobials
HTR1A	5-hydroxytr	5-HT-1A|5-	5	Antimicrobials
SEPTIN7	septin 7	CDC10|CD	7	Antimicrobials
CD40LG	CD40 ligan	CD154|CD	X	Antimicrobials
CD14	CD14 mole	-	5	Antimicrobials
CD8A	CD8a mole	CD8|Leu2|p	2	Antimicrobials

<table>
<tr><th></th><th></th><th></th><th></th><th></th></tr>
<tr><td>CD4</td><td>CD4 molec</td><td>CD4mut</td><td>12</td><td>Antimicrobials</td></tr>
<tr><td>MASP1</td><td>mannan bin</td><td>3MC1|CRA</td><td>3</td><td>Antimicrobials</td></tr>
<tr><td>PROC</td><td>protein C, i</td><td>APC|PC|PR</td><td>2</td><td>Antimicrobials</td></tr>
<tr><td>MAP2K2</td><td>mitogen-ac</td><td>CFC4|MAI</td><td>19</td><td>Antimicrobials</td></tr>
<tr><td>MAP2K1</td><td>mitogen-ac</td><td>CFC3|MAI</td><td>15</td><td>Antimicrobials</td></tr>
<tr><td>HRG</td><td>histidine ric</td><td>HPRG|HR</td><td>3</td><td>Antimicrobials</td></tr>
<tr><td>NDRG1</td><td>N-myc dow</td><td>CAP43|CM</td><td>8</td><td>Antimicrobials</td></tr>
<tr><td>IRF9</td><td>interferon r</td><td>IRF-9|ISGI</td><td>14</td><td>Antimicrobials</td></tr>
<tr><td>TRIM22</td><td>tripartite m</td><td>GPSTAF50</td><td>11</td><td>Antimicrobials</td></tr>
<tr><td>LANCL1</td><td>LanC like 1</td><td>GPR69A|p4</td><td>2</td><td>Antimicrobials</td></tr>
<tr><td>PPP4C</td><td>protein pho</td><td>PP-X|PP4|I</td><td>16</td><td>Antimicrobials</td></tr>
<tr><td>HMOX1</td><td>heme oxyg</td><td>HMOX1D|</td><td>22</td><td>Antimicrobials</td></tr>
<tr><td>HMGB1</td><td>high mobili</td><td>HMG-1|HM</td><td>13</td><td>Antimicrobials</td></tr>
<tr><td>HLA-B</td><td>major histo</td><td>AS|B-4901|</td><td>6</td><td>Antimicrobials</td></tr>
<tr><td>RNASE7</td><td>ribonucleas</td><td>RAE1</td><td>14</td><td>Antimicrobials</td></tr>
<tr><td>ABCC4</td><td>ATP bindin</td><td>MOAT-B|M</td><td>13</td><td>Antimicrobials</td></tr>
<tr><td>HGF</td><td>hepatocyte</td><td>DFNB39|F-</td><td>7</td><td>Antimicrobials</td></tr>
<tr><td>HDAC1</td><td>histone dea</td><td>GON-10|HI</td><td>1</td><td>Antimicrobials</td></tr>
<tr><td>IFNLR1</td><td>interferon l</td><td>CRF2/12|IF</td><td>1</td><td>Antimicrobials</td></tr>
<tr><td>PLSCR1</td><td>phospholipi</td><td>MMTRA1I</td><td>3</td><td>Antimicrobials</td></tr>
<tr><td>B2M</td><td>beta-2-micr</td><td>IMD43</td><td>15</td><td>Antimicrobials</td></tr>
<tr><td>BACH2</td><td>BTB domai</td><td>BTBD25|IN</td><td>6</td><td>Antimicrobials</td></tr>
<tr><td>TANK</td><td>TRAF fami</td><td>I-TRAF|ITI</td><td>2</td><td>Antimicrobials</td></tr>
<tr><td>PIK3CG</td><td>phosphatid</td><td>PI3CG|PI3I</td><td>7</td><td>Antimicrobials</td></tr>
<tr><td>ARRB1</td><td>arrestin bet</td><td>ARB1|ARR</td><td>11</td><td>Antimicrobials</td></tr>
<tr><td>RSAD2</td><td>radical S-ad</td><td>2510004L0</td><td>2</td><td>Antimicrobials</td></tr>
<tr><td>STAB2</td><td>stabilin 2</td><td>FEEL2|FEI</td><td>12</td><td>Antimicrobials</td></tr>
<tr><td>TBK1</td><td>TANK binc</td><td>FTDALS4|</td><td>12</td><td>Antimicrobials</td></tr>
<tr><td>PDYN</td><td>prodynorph</td><td>ADCA|PEN</td><td>20</td><td>Antimicrobials</td></tr>
<tr><td>PDGFRB</td><td>platelet der</td><td>CD140B|IB</td><td>5</td><td>Antimicrobials</td></tr>
<tr><td>PDCD1</td><td>programme</td><td>CD279|PD-</td><td>2</td><td>Antimicrobials</td></tr>
<tr><td>PCSK2</td><td>proprotein</td><td>NEC 2|NEC</td><td>20</td><td>Antimicrobials</td></tr>
<tr><td>PCSK1</td><td>proprotein</td><td>BMIQ12|N</td><td>5</td><td>Antimicrobials</td></tr>
<tr><td>ARG2</td><td>arginase 2</td><td>-</td><td>14</td><td>Antimicrobials</td></tr>
<tr><td>AQP9</td><td>aquaporin 9</td><td>AQP-9|HsT</td><td>15</td><td>Antimicrobials</td></tr>
<tr><td>FASLG</td><td>Fas ligand</td><td>ALPS1B|A</td><td>1</td><td>Antimicrobials</td></tr>
<tr><td>APOH</td><td>apolipoprot</td><td>B2G1|B2GI</td><td>17</td><td>Antimicrobials</td></tr>
<tr><td>BIRC5</td><td>baculoviral</td><td>API4|EPR-</td><td>17</td><td>Antimicrobials</td></tr>
<tr><td>ANXA6</td><td>annexin A6</td><td>ANX6|CBF</td><td>5</td><td>Antimicrobials</td></tr>
<tr><td>IL22</td><td>interleukin</td><td>IL-21|IL-22</td><td>12</td><td>Antimicrobials</td></tr>
<tr><td>VTN</td><td>vitronectin</td><td>V75|VN|VN</td><td>17</td><td>Antimicrobials</td></tr>
<tr><td>VIM</td><td>vimentin</td><td>-</td><td>10</td><td>Antimicrobials</td></tr>
<tr><td>VCAM1</td><td>vascular ce</td><td>CD106|INC</td><td>1</td><td>Antimicrobials</td></tr>
<tr><td>PRDX1</td><td>peroxiredo</td><td>MSP23|NK</td><td>1</td><td>Antimicrobials</td></tr>
<tr><td>GFAP</td><td>glial fibrilla</td><td>ALXDRD</td><td>17</td><td>Antimicrobials</td></tr>
<tr><td>GBP2</td><td>guanylate b</td><td>-</td><td>1</td><td>Antimicrobials</td></tr>
<tr><td>ALB</td><td>albumin</td><td>HSA|PRO0</td><td>4</td><td>Antimicrobials</td></tr>
<tr><td>SLC29A3</td><td>solute carri</td><td>ENT3|HCL</td><td>10</td><td>Antimicrobials</td></tr>
<tr><td>OAS1</td><td>2'-5'-oligoa</td><td>E18/E16|IF</td><td>12</td><td>Antimicrobials</td></tr>
<tr><td>AGER</td><td>advanced g</td><td>RAGE|SCA</td><td>6</td><td>Antimicrobials</td></tr>
<tr><td>UNC93B1</td><td>unc-93 hon</td><td>IIAE1|UNC</td><td>11</td><td>Antimicrobials</td></tr>
<tr><td>TNFSF4</td><td>TNF superf</td><td>CD134L|CI</td><td>1</td><td>Antimicrobials</td></tr>
<tr><td>NOS1</td><td>nitric oxide</td><td>IHPS1|N-N</td><td>12</td><td>Antimicrobials</td></tr>
<tr><td>ACTG1</td><td>actin gamm</td><td>ACT|ACTC</td><td>17</td><td>Antimicrobials</td></tr>
<tr><td>ACTA1</td><td>actin alpha</td><td>ACTA|ASM</td><td>1</td><td>Antimicrobials</td></tr>
<tr><td>ACO1</td><td>aconitase 1</td><td>ACONS|HI</td><td>9</td><td>Antimicrobials</td></tr>
<tr><td>SERPINA3</td><td>serpin fami</td><td>AACT|ACT</td><td>14</td><td>Antimicrobials</td></tr>
<tr><td>CXCR1</td><td>C-X-C mot</td><td>C-C|C-C-C</td><td>2</td><td>Antimicrobials</td></tr>
<tr><td>CCL15</td><td>C-C motif</td><td>HCC-2|HM</td><td>17</td><td>Antimicrobials</td></tr>
<tr><td>CCL14</td><td>C-C motif</td><td>CC-1|CC-3</td><td>17</td><td>Antimicrobials</td></tr>
</table>

CCL4	C-C motif c	ACT2|AT7	17	Antimicrobials
CCL16	C-C motif c	CKb12|HC	17	Antimicrobials
CCL19	C-C motif c	CKb11|EL	9	Antimicrobials
CCL13	C-C motif c	CKb10|MC	17	Antimicrobials
CCL18	C-C motif c	AMAC-1|A	17	Antimicrobials
CCL17	C-C motif c	A-152E5.3|	16	Antimicrobials
CCL26	C-C motif c	IMAC|MIP	7	Antimicrobials
CCL22	C-C motif c	A-152E5.1|	16	Antimicrobials
CCR3	C-C motif c	C C CKR3|	3	Antimicrobials
CCL28	C-C motif c	CCK1|ME	5	Antimicrobials
CCL4L1	C-C motif c	AT744.2|C	17	Antimicrobials
ACKR2	atypical ch	CCBP2|CC	3	Antimicrobials
CCR7	C-C motif c	BLR2|CC-	17	Antimicrobials
CCL27	C-C motif c	ALP|CTAC	9	Antimicrobials
CCR8	C-C motif c	CC-CKR-8	3	Antimicrobials
ACKR4	atypical ch	CC-CKR-1	3	Antimicrobials
CCR10	C-C motif c	GPR2	17	Antimicrobials
CCL2	C-C motif c	GDCF-2|H	17	Antimicrobials
CCL21	C-C motif c	6Ckine|CK	9	Antimicrobials
CCL7	C-C motif c	FIC|MARC	17	Antimicrobials
CCL5	C-C motif c	D17S136E|	17	Antimicrobials
CCL3	C-C motif c	G0S19-1|L	17	Antimicrobials
CCL20	C-C motif c	CKb4|Exod	2	Antimicrobials
CCL11	C-C motif c	SCYA11	17	Antimicrobials
CCR5	C-C motif c	CC-CKR-5	3	Antimicrobials
CCL23	C-C motif c	CK-BETA-	17	Antimicrobials
CCL25	C-C motif c	Ckb15|SCY	19	Antimicrobials
CCL1	C-C motif c	I-309|P500|	17	Antimicrobials
CCL3L3	C-C motif c	464.2|D17S	17	Antimicrobials
CCL4L2	C-C motif c	AT744.2|C	17	Antimicrobials
CXCL12	C-X-C mot	IRH|PBSF|	10	Antimicrobials
XCL1	X-C motif c	ATAC|LPT	1	Antimicrobials
CCL8	C-C motif c	HC14|MCP	17	Antimicrobials
CCL3L1	C-C motif c	464.2|D17S	17	Antimicrobials
CCR1	C-C motif c	CD191|CK	3	Antimicrobials
CCL24	C-C motif c	Ckb-6|MPI	7	Antimicrobials
XCL2	X-C motif c	SCM-1b|SC	1	Antimicrobials
CXCL1	C-X-C mot	FSP|GRO1|	4	Antimicrobials
CXCL10	C-X-C mot	C7|IFI10|IN	4	Antimicrobials
CXCR4	C-X-C mot	CD184|D2S	2	Antimicrobials
CXCL2	C-X-C mot	CINC-2a|G	4	Antimicrobials
CXCR6	C-X-C mot	BONZO|CI	3	Antimicrobials
CCR4	C-C motif c	CC-CKR-4	3	Antimicrobials
CXCL11	C-X-C mot	H174|I-TA	4	Antimicrobials
TAFA5	TAFA cher	FAM19A5|	22	Antimicrobials
TAFA3	TAFA cher	FAM19A3|	1	Antimicrobials
TAFA4	TAFA cher	FAM19A4|	3	Antimicrobials
TAFA1	TAFA cher	FAM19A1|	3	Antimicrobials
TAFA2	TAFA cher	FAM19A2|	12	Antimicrobials
CCL15-CC	CCL15-CC	CCL15|HC	17	Antimicrobials
IL6	interleukin	BSF-2|BSF	7	Antimicrobials
TNF	tumor necr	DIF|TNF-a	6	Antimicrobials
IL1B	interleukin	IL-1|IL1-B	2	Antimicrobials
IL18	interleukin	IGIF|IL-18|	11	Antimicrobials
PTK2B	protein tyro	CADTK|C	8	Antimicrobials
VEGFA	vascular en	MVCD1|V	6	Antimicrobials
IL4	interleukin	BCGF-1|B	5	Antimicrobials
CDH1	cadherin 1	Arc-1|BCD	16	Antimicrobials
CD40	CD40 mole	Bp50|CDW	20	Antimicrobials
DEFB103B	defensin be	BD-3|DEF	8	Antimicrobials

F2RL1	F2R like try	GPR11|PAI	5	Antimicrobials
MMP9	matrix meta	CLG4B|GE	20	Antimicrobials
LTBP1	latent transf	-	2	Antimicrobials
DEFB4A	defensin be	BD-2|DEFI	8	Antimicrobials
TNFSF10	TNF superf	APO2L|Ap	3	Antimicrobials
IL13	interleukin	IL-13|P600	5	Antimicrobials
IL10	interleukin	CSIF|GVH	1	Antimicrobials
IL2	interleukin	IL-2|TCGF	4	Antimicrobials
PPARG	peroxisome	CIMT1|GL	3	Antimicrobials
FGR	FGR proto-	SRC2|c-fgr	1	Antimicrobials
MIF	macrophag	GIF|GLIF|N	22	Antimicrobials
CRP	C-reactive	PTX1	1	Antimicrobials
JAK2	Janus kinas	JTK10|THC	9	Antimicrobials
IL1A	interleukin	IL-1 alpha|I	2	Antimicrobials
PTK2	protein tyro	FADK|FAK	8	Antimicrobials
PTGDR	prostagland	AS1|ASRT	14	Antimicrobials
CD86	CD86 mole	B7-2|B7.2|I	3	Antimicrobials
HCK	HCK proto-	JTK9|p59H	20	Antimicrobials
ARRB1	arrestin bet	ARB1|ARR	11	Antimicrobials
GNAI1	G protein s	Gi	7	Antimicrobials
VDR	vitamin D r	NR1I1|PPP	12	Antimicrobials
OLR1	oxidized lo	CLEC8A|L	12	Antimicrobials
GRK2	G protein-c	ADRBK1|E	11	Antimicrobials
TXK	TXK tyrosi	BTKL|PSC	4	Antimicrobials
RNASE2	ribonucleas	EDN|RAF3	14	Antimicrobials
CD79A	CD79a mol	IGA|MB-1	19	BCRSignalingPathway
CD79B	CD79b mol	AGM6|B29	17	BCRSignalingPathway
LYN	LYN proto-	JTK8|p53L	8	BCRSignalingPathway
SYK	spleen asso	p72-Syk	9	BCRSignalingPathway
BTK	Bruton tyro	AGMX1|A X		BCRSignalingPathway
BLNK	B cell linke	AGM4|BAS	10	BCRSignalingPathway
VAV3	vav guanin	-	1	BCRSignalingPathway
VAV1	vav guanin	VAV	19	BCRSignalingPathway
VAV2	vav guanin	VAV-2	9	BCRSignalingPathway
RAC1	Rac family	MIG5|MRD	7	BCRSignalingPathway
RAC2	Rac family	EN-7|Gx|H	22	BCRSignalingPathway
RAC3	Rac family	-	17	BCRSignalingPathway
PPP3CA	protein pho	ACCIID|CA	4	BCRSignalingPathway
PPP3CB	protein pho	CALNA2|C	10	BCRSignalingPathway
PPP3CC	protein pho	CALNA3|C	8	BCRSignalingPathway
CHP1	calcineurin	CHP|SLC9	15	BCRSignalingPathway
PPP3R1	protein pho	CALNB1|C	2	BCRSignalingPathway
PPP3R2	protein pho	PPP3RL	9	BCRSignalingPathway
CHP2	calcineurin	-	16	BCRSignalingPathway
NFAT5	nuclear fact	NF-AT5|NI	16	BCRSignalingPathway
NFATC1	nuclear fact	NF-ATC|N	18	BCRSignalingPathway
NFATC2	nuclear fact	NFAT1|NF	20	BCRSignalingPathway
NFATC3	nuclear fact	NF-AT4c|N	16	BCRSignalingPathway
NFATC4	nuclear fact	NF-AT3|NI	14	BCRSignalingPathway
HRAS	HRas proto	C-BAS/HA	11	BCRSignalingPathway
KRAS	KRAS prot	'C-K-RAS|(	12	BCRSignalingPathway
NRAS	NRAS prot	ALPS4|CM	1	BCRSignalingPathway
FOS	Fos proto-o	AP-1|C-FO	14	BCRSignalingPathway
JUN	Jun proto-o	AP-1|AP1|c	1	BCRSignalingPathway
CARD11	caspase rec	BENTA|BI	7	BCRSignalingPathway
BCL10	BCL10 imm	CARMEN|(	1	BCRSignalingPathway
MALT1	MALT1 pa	IMD12|ML	18	BCRSignalingPathway
CHUK	component	IKBKA|IKI	10	BCRSignalingPathway
IKBKB	inhibitor of	IKK-beta|II	8	BCRSignalingPathway
IKBKG	inhibitor of	AMCBX1|IX		BCRSignalingPathway

NFKB1	nuclear fact	CVID12|E	4	BCRSignalingPathway
RELA	RELA prot	CMCU|NF	11	BCRSignalingPathway
NFKBIA	NFKB inhi	EDAID2|I	14	BCRSignalingPathway
NFKBIB	NFKB inhi	IKBB|TRI	19	BCRSignalingPathway
NFKBIE	NFKB inhi	IKBE	6	BCRSignalingPathway
CD81	CD81 mole	CVID6|S5.	11	BCRSignalingPathway
CD19	CD19 mole	B4|CVID3	16	BCRSignalingPathway
CR2	complemen	C3DR|CD2	1	BCRSignalingPathway
PIK3R5	phosphoino	F730038I1	17	BCRSignalingPathway
PIK3R1	phosphoino	AGM7|GR	5	BCRSignalingPathway
PIK3R2	phosphoino	MPPH|MP	19	BCRSignalingPathway
PIK3R3	phosphoino	p55|p55-G	1	BCRSignalingPathway
PIK3CA	phosphatid	CLAPO|CL	3	BCRSignalingPathway
PIK3CB	phosphatid	P110BETA	3	BCRSignalingPathway
PIK3CD	phosphatid	APDS|IMD	1	BCRSignalingPathway
PIK3CG	phosphatid	PI3CG|PI3	7	BCRSignalingPathway
AKT3	AKT serine	MPPH|MP	1	BCRSignalingPathway
AKT1	AKT serine	AKT|CWS	14	BCRSignalingPathway
AKT2	AKT serine	HIHGHH|P	19	BCRSignalingPathway
GSK3B	glycogen s	-	3	BCRSignalingPathway
INPP5D	inositol pol	SHIP|SHIP	2	BCRSignalingPathway
CD22	CD22 mole	SIGLEC-2|	19	BCRSignalingPathway
CD72	CD72 mole	CD72b|LY	9	BCRSignalingPathway
PTPN6	protein tyro	HCP|HCPH	12	BCRSignalingPathway
LILRB3	leukocyte i	CD85A|HL	19	BCRSignalingPathway
FCGR2B	Fc fragmen	CD32|CD3	1	BCRSignalingPathway
RASGRP3	RAS guany	GRP3	2	BCRSignalingPathway
PLCG2	phospholip	APLAID|F	16	BCRSignalingPathway
PRKCB	protein kin	PKC-beta|P	16	BCRSignalingPathway
IFITM1	interferon i	9-27|CD22	11	BCRSignalingPathway
IGH	immunoglo	IGD1|IGH.	14	BCRSignalingPathway
IGHA1	immunoglo	IgA1	14	BCRSignalingPathway
IGHA2	immunoglo	-	14	BCRSignalingPathway
IGHD	immunoglo	-	14	BCRSignalingPathway
IGHD1-1	immunoglo	IGHD11	14	BCRSignalingPathway
IGHD1-14	immunoglo	DM2|IGH	14	BCRSignalingPathway
IGHD1-20	immunoglo	IGHD120	14	BCRSignalingPathway
IGHD1-26	immunoglo	IGHD126	14	BCRSignalingPathway
IGHD1-7	immunoglo	DM1|IGH	14	BCRSignalingPathway
IGHD2-15	immunoglo	D2|IGHD2	14	BCRSignalingPathway
IGHD2-2	immunoglo	IGHD22	14	BCRSignalingPathway
IGHD2-21	immunoglo	IGHD221	14	BCRSignalingPathway
IGHD2-8	immunoglo	DLR1|IGH	14	BCRSignalingPathway
IGHD3-10	immunoglo	DXP'1|IGH	14	BCRSignalingPathway
IGHD3-16	immunoglo	IGHD316	14	BCRSignalingPathway
IGHD3-22	immunoglo	IGHD322	14	BCRSignalingPathway
IGHD3-3	immunoglo	DXP4|IGH	14	BCRSignalingPathway
IGHD3-9	immunoglo	DXP1|IGH	14	BCRSignalingPathway
IGHD4-11	immunoglo	DA1|IGHD	14	BCRSignalingPathway
IGHD4-17	immunoglo	IGHD417	14	BCRSignalingPathway
IGHD4-23	immunoglo	IGHD423	14	BCRSignalingPathway
IGHD4-4	immunoglo	DA4|IGHD	14	BCRSignalingPathway
IGHD5-12	immunoglo	DK1|IGHD	14	BCRSignalingPathway
IGHD5-18	immunoglo	IGHD518	14	BCRSignalingPathway
IGHD5-24	immunoglo	IGHD524	14	BCRSignalingPathway
IGHD5-5	immunoglo	DK4|IGHD	14	BCRSignalingPathway
IGHD6-13	immunoglo	DN1|IGHD	14	BCRSignalingPathway
IGHD6-19	immunoglo	IGHD619	14	BCRSignalingPathway
IGHD6-25	immunoglo	IGHD625	14	BCRSignalingPathway
IGHD6-6	immunoglo	D(N4)|IGH	14	BCRSignalingPathway

IGHD7-27	immunoglo	DHQ52|IG	14	BCRSignalingPathway
IGHE	immunoglo	IgE	14	BCRSignalingPathway
IGHG1	immunoglo	-	14	BCRSignalingPathway
IGHG2	immunoglo	-	14	BCRSignalingPathway
IGHG3	immunoglo	IgG3	14	BCRSignalingPathway
IGHG4	immunoglo	-	14	BCRSignalingPathway
IGHJ1	immunoglo	JH1	14	BCRSignalingPathway
IGHJ2	immunoglo	JH2	14	BCRSignalingPathway
IGHJ3	immunoglo	JH3b	14	BCRSignalingPathway
IGHJ4	immunoglo	JH4b	14	BCRSignalingPathway
IGHJ5	immunoglo	JH5b	14	BCRSignalingPathway
IGHJ6	immunoglo	JH6b	14	BCRSignalingPathway
IGHM	immunoglo	AGM1|MU	14	BCRSignalingPathway
IGH	immunoglo	IGD1|IGH.	14	BCRSignalingPathway
IGHV1-18	immunoglo	IGHV118	14	BCRSignalingPathway
IGHV1-2	immunoglo	IGHV12|V	14	BCRSignalingPathway
IGHV1-24	immunoglo	IGHV124|	14	BCRSignalingPathway
IGHV1-3	immunoglo	IGHV13|V	14	BCRSignalingPathway
IGHV1-45	immunoglo	IGHV145|	14	BCRSignalingPathway
IGHV1-46	immunoglo	IGHV146	14	BCRSignalingPathway
IGHV1-58	immunoglo	IGHV158|	14	BCRSignalingPathway
IGHV1-69	immunoglo	IGHV1-E|I	14	BCRSignalingPathway
IGHV1-8	immunoglo	IGHV18	14	BCRSignalingPathway
IGHV1-38-	immunoglo	IGHV1-C|I	14	BCRSignalingPathway
IGHV1-69-	immunoglo	IGHV1-F|I	14	BCRSignalingPathway
IGHV2-26	immunoglo	IGHV226|	14	BCRSignalingPathway
IGHV2-5	immunoglo	IGHV25|V	14	BCRSignalingPathway
IGHV2-70	immunoglo	IGHV270|	14	BCRSignalingPathway
IGHV3-11	immunoglo	IGHV311|	14	BCRSignalingPathway
IGHV3-13	immunoglo	IGHV313	14	BCRSignalingPathway
IGHV3-15	immunoglo	IGHV315|	14	BCRSignalingPathway
IGHV3-16	immunoglo	IGHV316|	14	BCRSignalingPathway
IGHV3-20	immunoglo	IGHV320|	14	BCRSignalingPathway
IGHV3-21	immunoglo	IGHV321|	14	BCRSignalingPathway
IGHV3-23	immunoglo	DP47|IGH	14	BCRSignalingPathway
IGHV3-30	immunoglo	IGHV330|	14	BCRSignalingPathway
IGHV3-30-	immunoglo	IGHV3-3|I	14	BCRSignalingPathway
IGHV3-30-	immunoglo	IGHV3-3|I	14	BCRSignalingPathway
IGHV3-33	immunoglo	IGHV333|	14	BCRSignalingPathway
IGHV3-35	immunoglo	IGHV335|	14	BCRSignalingPathway
IGHV3-38	immunoglo	IGHV338|	14	BCRSignalingPathway
IGHV3-43	immunoglo	IGHV343|	14	BCRSignalingPathway
IGHV3-48	immunoglo	IGHV348|	14	BCRSignalingPathway
IGHV3-49	immunoglo	IGHV349|	14	BCRSignalingPathway
IGHV3-53	immunoglo	IGHV353|	14	BCRSignalingPathway
IGHV3-64	immunoglo	IGHV364|	14	BCRSignalingPathway
IGHV3-66	immunoglo	IGHV366|	14	BCRSignalingPathway
IGHV3-7	immunoglo	IGHV37|V	14	BCRSignalingPathway
IGHV3-72	immunoglo	IGHV372|	14	BCRSignalingPathway
IGHV3-73	immunoglo	IGHV373|	14	BCRSignalingPathway
IGHV3-74	immunoglo	IGHV374|	14	BCRSignalingPathway
IGHV3-9	immunoglo	IGHV39|V	14	BCRSignalingPathway
IGHV3-38-	immunoglo	IGHV3-D|I	14	BCRSignalingPathway
IGHV3-69-	immunoglo	IGH|IGHM	14	BCRSignalingPathway
IGHV4-28	immunoglo	IGHV428|	14	BCRSignalingPathway
IGHV4-30-	immunoglo	IGHV4-3	14	BCRSignalingPathway
IGHV4-30-	immunoglo	IGHV4-3|I	14	BCRSignalingPathway
IGHV4-30-	immunoglo	IGHV4-3|I	14	BCRSignalingPathway
IGHV4-31	immunoglo	IGHV431	14	BCRSignalingPathway
IGHV4-34	immunoglo	IGHV434|	14	BCRSignalingPathway

IGHV4-39	immunoglo	IGHV439|V	14	BCRSignalingPathway
IGHV4-4	immunoglo	IGHV44|V	14	BCRSignalingPathway
IGHV4-59	immunoglo	IGHV459|V	14	BCRSignalingPathway
IGHV4-61	immunoglo	IGHV461|V	14	BCRSignalingPathway
IGHV4-38-	immunoglo	IGHV4-B|I	14	BCRSignalingPathway
IGHV5-51	immunoglo	IGHV551|V	14	BCRSignalingPathway
IGHV5-10-	immunoglo	IGHV5-A|I	14	BCRSignalingPathway
IGHV6-1	immunoglo	IGHV61|V	14	BCRSignalingPathway
IGHV7-4-1	immunoglo	IGHV7-41|	14	BCRSignalingPathway
IGHV7-81	immunoglo	IGHV781	14	BCRSignalingPathway
IGK	immunoglo	IGK@	2	BCRSignalingPathway
IGKC	immunoglo	HCAK1|IG	2	BCRSignalingPathway
IGKDEL	immunoglo	IGKDE	2	BCRSignalingPathway
IGKJ	-	IGKJ@	2	BCRSignalingPathway
IGKJ1	immunoglo	J1	2	BCRSignalingPathway
IGKJ2	immunoglo	J2	2	BCRSignalingPathway
IGKJ3	immunoglo	J3	2	BCRSignalingPathway
IGKJ4	immunoglo	J4	2	BCRSignalingPathway
IGKJ5	immunoglo	J5	2	BCRSignalingPathway
IGKV@	-	IGKV|IGK	2	BCRSignalingPathway
IGKV1-12	immunoglo	IGKV112|I	2	BCRSignalingPathway
IGKV1-13	immunoglo	IGKV113|I	2	BCRSignalingPathway
IGKV1-16	immunoglo	IGKV116|I	2	BCRSignalingPathway
IGKV1-17	immunoglo	A30|IGKV	2	BCRSignalingPathway
IGKV1-27	immunoglo	A20|IGKV	2	BCRSignalingPathway
IGKV1-33	immunoglo	IGKV133|	2	BCRSignalingPathway
IGKV1-37	immunoglo	IGKV137|	2	BCRSignalingPathway
IGKV1-39	immunoglo	IGKV139|	2	BCRSignalingPathway
IGKV1-5	immunoglo	IGKV|IGK	2	BCRSignalingPathway
IGKV1-6	immunoglo	IGKV16|L	2	BCRSignalingPathway
IGKV1-8	immunoglo	IGKV18|L	2	BCRSignalingPathway
IGKV1-9	immunoglo	IGKV19|L	2	BCRSignalingPathway
IGKV1D-1	immunoglo	IGKV1D12	2	BCRSignalingPathway
IGKV1D-1	immunoglo	IGKV1D13	2	BCRSignalingPathway
IGKV1D-1	immunoglo	IGKV1D16	2	BCRSignalingPathway
IGKV1D-1	immunoglo	IGKV1D17	2	BCRSignalingPathway
IGKV1D-3	immunoglo	IGKV1D33	2	BCRSignalingPathway
IGKV1D-3	immunoglo	IGKV1D37	2	BCRSignalingPathway
IGKV1D-3	immunoglo	IGKV1D39	2	BCRSignalingPathway
IGKV1D-4	immunoglo	IGKV1D42	2	BCRSignalingPathway
IGKV1D-4	immunoglo	IGKV1D43	2	BCRSignalingPathway
IGKV1D-8	immunoglo	IGKV1D8|	2	BCRSignalingPathway
IGKV2-24	immunoglo	A23|IGKV	2	BCRSignalingPathway
IGKV2-28	immunoglo	A19|IGKV	2	BCRSignalingPathway
IGKV2-30	immunoglo	A17|IGKV	2	BCRSignalingPathway
IGKV2-40	immunoglo	IGKV240|	2	BCRSignalingPathway
IGKV2D-2	immunoglo	A7|IGKV2	2	BCRSignalingPathway
IGKV2D-2	immunoglo	A3|IGKV2	2	BCRSignalingPathway
IGKV2D-2	immunoglo	A2a|A2c|I	2	BCRSignalingPathway
IGKV2D-3	immunoglo	A1|IGKV2	2	BCRSignalingPathway
IGKV2D-4	immunoglo	IGKV2D40	2	BCRSignalingPathway
IGKV3-11	immunoglo	IGKV311|I	2	BCRSignalingPathway
IGKV3-15	immunoglo	IGKV315|I	2	BCRSignalingPathway
IGKV3-20	immunoglo	13K18|A27	2	BCRSignalingPathway
IGKV3-7	immunoglo	IGKV37|L	2	BCRSignalingPathway
IGKV3D-1	immunoglo	IGKV3D11	2	BCRSignalingPathway
IGKV3D-1	immunoglo	IGKV3D15	2	BCRSignalingPathway
IGKV3D-2	immunoglo	A11|A11a|I	2	BCRSignalingPathway
IGKV3D-7	immunoglo	IGKV3D7|	2	BCRSignalingPathway
IGKV4-1	immunoglo	B3|IGKV4	2	BCRSignalingPathway

IGKV5-2	immunoglo	B2|IGKV52	2	BCRSignalingPathway
IGKV6-21	immunoglo	A26|IGKV6	2	BCRSignalingPathway
IGKV6D-2	immunoglo	A10|IGKV6	2	BCRSignalingPathway
IGKV6D-4	immunoglo	A14	2	BCRSignalingPathway
IGL	immunoglo	IGL@|IGL(	22	BCRSignalingPathway
IGLC1	immunoglo	IGLC	22	BCRSignalingPathway
IGLC2	immunoglo	IGLC	22	BCRSignalingPathway
IGLC3	immunoglo	IGLC	22	BCRSignalingPathway
IGLC6	immunoglo	IGLC	22	BCRSignalingPathway
IGLC7	immunoglo	C7	22	BCRSignalingPathway
IGLJ	-	IGLJ@	22	BCRSignalingPathway
IGLJ1	immunoglo	J1	22	BCRSignalingPathway
IGLJ2	immunoglo	J2	22	BCRSignalingPathway
IGLJ3	immunoglo	J3	22	BCRSignalingPathway
IGLJ4	immunoglo	-	22	BCRSignalingPathway
IGLJ5	immunoglo	-	22	BCRSignalingPathway
IGLJ6	immunoglo	-	22	BCRSignalingPathway
IGLJ7	immunoglo	J7	22	BCRSignalingPathway
IGLV@	-	IGLV	22	BCRSignalingPathway
IGLV1-36	immunoglo	IGLV136|V	22	BCRSignalingPathway
IGLV1-40	immunoglo	IGLV140|V	22	BCRSignalingPathway
IGLV1-44	immunoglo	IGLV144|V	22	BCRSignalingPathway
IGLV1-47	immunoglo	IGLV147|V	22	BCRSignalingPathway
IGLV1-50	immunoglo	IGLV150|V	22	BCRSignalingPathway
IGLV1-51	immunoglo	IGLV151|V	22	BCRSignalingPathway
IGLV10-54	immunoglo	IGLV1054|	22	BCRSignalingPathway
IGLV11-55	immunoglo	IGLV1155|	22	BCRSignalingPathway
IGLV2-11	immunoglo	IGLV211|V	22	BCRSignalingPathway
IGLV2-14	immunoglo	IGLV214|V	22	BCRSignalingPathway
IGLV2-18	immunoglo	IGLV218|V	22	BCRSignalingPathway
IGLV2-23	immunoglo	IGLV223|V	22	BCRSignalingPathway
IGLV2-33	immunoglo	IGLV233|V	22	BCRSignalingPathway
IGLV2-8	immunoglo	IGLV28|V1	22	BCRSignalingPathway
IGLV3-1	immunoglo	IGLV31|V2	22	BCRSignalingPathway
IGLV3-10	immunoglo	IGLV310|V	22	BCRSignalingPathway
IGLV3-12	immunoglo	IGLV312|V	22	BCRSignalingPathway
IGLV3-16	immunoglo	IGLV316|V	22	BCRSignalingPathway
IGLV3-19	immunoglo	IGLV319|V	22	BCRSignalingPathway
IGLV3-21	immunoglo	IGLV321|V	22	BCRSignalingPathway
IGLV3-22	immunoglo	IGLV322|V	22	BCRSignalingPathway
IGLV3-25	immunoglo	IGLV325|V	22	BCRSignalingPathway
IGLV3-27	immunoglo	IGLV327|V	22	BCRSignalingPathway
IGLV3-32	immunoglo	IGLV332|V	22	BCRSignalingPathway
IGLV3-9	immunoglo	IGLV39|V2	22	BCRSignalingPathway
IGLV4-3	immunoglo	IGLV43|V5	22	BCRSignalingPathway
IGLV4-60	immunoglo	IGLV460|V	22	BCRSignalingPathway
IGLV4-69	immunoglo	IGLV469|V	22	BCRSignalingPathway
IGLV5-37	immunoglo	IGLV537|V	22	BCRSignalingPathway
IGLV5-39	immunoglo	IGLV539	22	BCRSignalingPathway
IGLV5-45	immunoglo	IGLV545|V	22	BCRSignalingPathway
IGLV5-48	immunoglo	IGLV548|V	22	BCRSignalingPathway
IGLV5-52	immunoglo	IGLV552|V	22	BCRSignalingPathway
IGLV6-57	immunoglo	IGLV657|V	22	BCRSignalingPathway
IGLV7-43	immunoglo	IGLV743|V	22	BCRSignalingPathway
IGLV7-46	immunoglo	IGLV746|V	22	BCRSignalingPathway
IGLV8-61	immunoglo	IGLV861|V	22	BCRSignalingPathway
IGLV9-49	immunoglo	IGLV949|V	22	BCRSignalingPathway
C3	complemen	AHUS5|AR	19	Chemokines
C5	complemen	C5D|C5a|C	9	Chemokines
CAMP	cathelicidin	CAP-18|CA	3	Chemokines

CCL1	C-C motif c	I-309|P500|	17	Chemokines
CCL11	C-C motif c	SCYA11	17	Chemokines
CCL13	C-C motif c	CKb10|MC	17	Chemokines
CCL14	C-C motif c	CC-1|CC-3	17	Chemokines
CCL15-CC	CCL15-CC	CCL15|HC	17	Chemokines
CCL15	C-C motif c	HCC-2|HM	17	Chemokines
CCL16	C-C motif c	CKb12|HC	17	Chemokines
CCL17	C-C motif c	A-152E5.3|	16	Chemokines
CCL18	C-C motif c	AMAC-1|A	17	Chemokines
CCL19	C-C motif c	CKb11|EL(	9	Chemokines
CCL2	C-C motif c	GDCF-2|H	17	Chemokines
CCL20	C-C motif c	CKb4|Exod	2	Chemokines
CCL21	C-C motif c	6Ckine|CK	9	Chemokines
CCL22	C-C motif c	A-152E5.1|	16	Chemokines
CCL23	C-C motif c	CK-BETA-	17	Chemokines
CCL24	C-C motif c	Ckb-6|MPI	7	Chemokines
CCL25	C-C motif c	Ckb15|SCY	19	Chemokines
CCL26	C-C motif c	IMAC|MIP	7	Chemokines
CCL27	C-C motif c	ALP|CTAC	9	Chemokines
CCL28	C-C motif c	CCK1|MEC	5	Chemokines
CCL3	C-C motif c	G0S19-1|L	17	Chemokines
CCL3L1	C-C motif c	464.2|D17S	17	Chemokines
CCL3P1	C-C motif c	CCL3L2|G	17	Chemokines
CCL3L3	C-C motif c	464.2|D17S	17	Chemokines
CCL4	C-C motif c	ACT2|AT7	17	Chemokines
CCL4L2	C-C motif c	AT744.2|C	17	Chemokines
CCL4L1	C-C motif c	AT744.2|C	17	Chemokines
CCL5	C-C motif c	D17S136E|	17	Chemokines
CCL7	C-C motif c	FIC|MARC	17	Chemokines
CCL8	C-C motif c	HC14|MCP	17	Chemokines
CKLF	chemokine	C32|CKLF	16	Chemokines
CMA1	chymase 1	CYH|MCT	14	Chemokines
CTSG	cathepsin G	CATG|CG	14	Chemokines
CX3CL1	C-X3-C mc	ABCD-3|C	16	Chemokines
CXCL1	C-X-C mot	FSP|GRO1|	4	Chemokines
CXCL10	C-X-C mot	C7|IFI10|IN	4	Chemokines
CXCL11	C-X-C mot	H174|I-TA	4	Chemokines
CXCL12	C-X-C mot	IRH|PBSF|	10	Chemokines
CXCL13	C-X-C mot	ANGIE|AN	4	Chemokines
CXCL14	C-X-C mot	BMAC|BR	5	Chemokines
CXCL16	C-X-C mot	CXCLG16|	17	Chemokines
CXCL17	C-X-C mot	DMC|Dcip	19	Chemokines
CXCL2	C-X-C mot	CINC-2a|G	4	Chemokines
CXCL3	C-X-C mot	CINC-2b|G	4	Chemokines
CXCL5	C-X-C mot	ENA-78|SC	4	Chemokines
CXCL6	C-X-C mot	CKA-3|GC	4	Chemokines
CXCL9	C-X-C mot	CMK|Hum	4	Chemokines
CCN1	cellular con	CYR61|GI(	1	Chemokines
DEFA1	defensin al	DEF1|DEF	8	Chemokines
DEFA3	defensin al	DEF3|HNP	8	Chemokines
DEFA5	defensin al	DEF5|HD-	8	Chemokines
DEFB1	defensin be	BD1|DEFB	8	Chemokines
DEFB103B	defensin be	BD-3|DEF	8	Chemokines
DEFB104A	defensin be	BD-4|DEF	8	Chemokines
DEFB4A	defensin be	BD-2|DEF	8	Chemokines
EDN1	endothelin	ARCND3|E	6	Chemokines
EDN2	endothelin	ET-2|ET2|P	1	Chemokines
EDN3	endothelin	ET-3|ET3|H	20	Chemokines
FGF10	fibroblast g	-	5	Chemokines
FGF2	fibroblast g	BFGF|FGF	4	Chemokines

HTN3	histatin 3	HIS2|HTN2	4	Chemokines
CXCL8	C-X-C mot	GCP-1|GCI	4	Chemokines
LECT2	leukocyte c	chm-II|chm	5	Chemokines
PF4	platelet fact	CXCL4|PF-	4	Chemokines
PF4V1	platelet fact	CXCL4L1|	4	Chemokines
PLAU	plasminoge	ATF|BDPL	10	Chemokines
PPBP	pro-platelet	B-TG1|Beta	4	Chemokines
PPBPP1	pro-platelet	PPBPL1|TC	4	Chemokines
PROK2	prokineticir	BV8|HH4|K	3	Chemokines
RNASE2	ribonucleas	EDN|RAF3	14	Chemokines
SAA1	serum amyl	PIG4|SAA|	11	Chemokines
SAA2	serum amyl	SAA|SAA1	11	Chemokines
SBDS	SBDS ribos	CGI-97|SD	7	Chemokines
SEMA3A	semaphorin	COLL1|HH	7	Chemokines
SEMA3B	semaphorin	LUCA-1|SE	3	Chemokines
SEMA3C	semaphorin	SEMAE|Se	7	Chemokines
SEMA3D	semaphorin	Sema-Z2|cc	7	Chemokines
SEMA3E	semaphorin	M-SEMAH	7	Chemokines
SEMA3F	semaphorin	SEMA-IV|S	3	Chemokines
SEMA3G	semaphorin	sem2	3	Chemokines
SEMA4A	semaphorin	CORD10|R	1	Chemokines
SEMA4B	semaphorin	SEMAC|Se	15	Chemokines
SEMA4C	semaphorin	M-SEMA-I	2	Chemokines
SEMA4D	semaphorin	A8|BB18|C	9	Chemokines
SEMA4F	ssemaphori	M-SEMA|F	2	Chemokines
SEMA4G	semaphorin	-	10	Chemokines
SEMA5A	semaphorin	SEMAF|ser	5	Chemokines
SEMA5B	semaphorin	SEMAG|Se	3	Chemokines
SEMA6A	semaphorin	HT018|SEM	5	Chemokines
SEMA6B	semaphorin	EPM11|SEI	19	Chemokines
SEMA6C	semaphorin	SEMAY|m-	1	Chemokines
SEMA6D	semaphorin	-	15	Chemokines
SEMA7A	semaphorin	CD108|CD	15	Chemokines
SLIT1	slit guidanc	MEGF4|SL	10	Chemokines
SLIT2	slit guidanc	SLIL3|Slit-	4	Chemokines
TNC	tenascin C	150-225|DI	9	Chemokines
TYMP	thymidine p	ECGF|ECG	22	Chemokines
XCL1	X-C motif c	ATAC|LPT	1	Chemokines
XCL2	X-C motif c	SCM-1b|SC	1	Chemokines
C5AR1	complemen	C5A|C5AR	19	Chemokine_Receptors
ACKR2	atypical che	CCBP2|CC	3	Chemokine_Receptors
CCR1	C-C motif c	CD191|CK	3	Chemokine_Receptors
CCR10	C-C motif c	GPR2	17	Chemokine_Receptors
CCR3	C-C motif c	C C CKR3|	3	Chemokine_Receptors
CCR4	C-C motif c	CC-CKR-4	3	Chemokine_Receptors
CCR5	C-C motif c	CC-CKR-5	3	Chemokine_Receptors
CCR6	C-C motif c	BN-1|C-C C	6	Chemokine_Receptors
CCR7	C-C motif c	BLR2|CC-C	17	Chemokine_Receptors
CCR8	C-C motif c	CC-CKR-8	3	Chemokine_Receptors
CCR9	C-C motif c	CC-CKR-9	3	Chemokine_Receptors
ACKR4	atypical che	CC-CKR-1	3	Chemokine_Receptors
CCRL2	C-C motif c	ACKR5|CK	3	Chemokine_Receptors
CMKLR1	chemerin cl	CHEMERI	12	Chemokine_Receptors
CX3CR1	C-X3-C mc	CCRL1|CM	3	Chemokine_Receptors
CXCR3	C-X-C mot	CD182|CD	X	Chemokine_Receptors
CXCR4	C-X-C mot	CD184|D2S	2	Chemokine_Receptors
CXCR5	C-X-C mot	BLR1|CD1	11	Chemokine_Receptors
CXCR6	C-X-C mot	BONZO|CI	3	Chemokine_Receptors
ACKR3	atypical che	CMKOR1|C	2	Chemokine_Receptors
CYSLTR1	cysteinyl le	CYSLT1|C	X	Chemokine_Receptors

CYSLTR2	cysteinyl le	CYSLT2|C		13	Chemokine_Receptors
ACKR1	atypical che	CCBP1|CD		1	Chemokine_Receptors
EDNRA	endothelin r	ET-A|ETA|		4	Chemokine_Receptors
EDNRB	endothelin r	ABCDS|ET		13	Chemokine_Receptors
FPR1	formyl pept	FMLP|FPR		19	Chemokine_Receptors
FPR2	formyl pept	ALXR|FMI		19	Chemokine_Receptors
FPR2	formyl pept	ALXR|FMI		19	Chemokine_Receptors
GPR17	G protein-c	-		2	Chemokine_Receptors
GPR32	G protein-c	RVDR1		19	Chemokine_Receptors
GPR33	G protein-c	-		14	Chemokine_Receptors
PTGDR2	prostagland	CD294|CR		11	Chemokine_Receptors
C5AR2	complemen	C5L2|GPF		19	Chemokine_Receptors
CXCR1	C-X-C mot	C-C|C-C-C		2	Chemokine_Receptors
CXCR2	C-X-C mot	CD182|CD		2	Chemokine_Receptors
LTB4R	leukotriene	BLT1|BLT		14	Chemokine_Receptors
LTB4R2	leukotriene	BLT2|BLT		14	Chemokine_Receptors
PLAUR	plasminoge	CD87|U-PA		19	Chemokine_Receptors
PLXNA1	plexin A1	NOV|NOV		3	Chemokine_Receptors
PLXNA2	plexin A2	OCT|PLXN		1	Chemokine_Receptors
PLXNA3	plexin A3	6.3|HSSEX	X		Chemokine_Receptors
PLXNA4	plexin A4	FAYV2820		7	Chemokine_Receptors
PLXNB1	plexin B1	PLEXIN-B		3	Chemokine_Receptors
PLXNB2	plexin B2	MM1|Nbla		22	Chemokine_Receptors
PLXNB3	plexin B3	PLEXB3|P	X		Chemokine_Receptors
PLXNC1	plexin C1	CD232|PL		12	Chemokine_Receptors
PLXND1	plexin D1	PLEXD1		3	Chemokine_Receptors
PTAFR	platelet acti	PAFR		1	Chemokine_Receptors
ROBO1	roundabout	DUTT1|SA		3	Chemokine_Receptors
ROBO2	roundabout	SAX3		3	Chemokine_Receptors
ROBO3	roundabout	HGPPS|HG		11	Chemokine_Receptors
RXFP3	relaxin fam	GPCR135|I		5	Chemokine_Receptors
XCR1	X-C motif c	CCXCR1|C		3	Chemokine_Receptors
ADIPOQ	adiponectin	ACDC|ACI		3	Cytokines
ADM	adrenomed	AM|PAMP		11	Cytokines
ADM2	adrenomed	AM2|dJ579		22	Cytokines
AGRP	agouti relat	AGRT|ART		16	Cytokines
AGT	angiotensin	ANHU|SEF		1	Cytokines
AMBN	ameloblasti	AI1F		4	Cytokines
AMELX	amelogenin	AI1E|AIH1	X		Cytokines
AMH	anti-Muller	MIF|MIS		19	Cytokines
ANGPTL5	angiopoieti	-		11	Cytokines
ANGPTL7	angiopoieti	AngX|CDT		1	Cytokines
APLN	apelin	APEL|XNP	X		Cytokines
AREG	amphiregul	AR|AREGI		4	Cytokines
MANF	mesenceph	ARMET|AI		3	Cytokines
CDNF	cerebral do	ARMETL1		10	Cytokines
ARTN	artemin	ART|ENOV		1	Cytokines
AVP	arginine va	ADH|ARV		20	Cytokines
AZU1	azurocidin	AZAMP|A		19	Cytokines
BDNF	brain deriv	ANON2|BU		11	Cytokines
BMP1	bone morph	OI13|PCOL		8	Cytokines
BMP10	bone morph	-		2	Cytokines
BMP15	bone morph	GDF9B|OD	X		Cytokines
BMP2	bone morph	BDA2|BMI		20	Cytokines
BMP3	bone morph	BMP-3A		4	Cytokines
BMP4	bone morph	BMP2B|BM		14	Cytokines
BMP5	bone morph	-		6	Cytokines
BMP6	bone morph	VGR|VGR		6	Cytokines
BMP7	bone morph	OP-1		20	Cytokines
BMP8A	bone morph	OP-2		1	Cytokines

BMP8B	bone morph	BMP8|OP2	1	Cytokines
BTC	betacellulin	-	4	Cytokines
MYDGF	myeloid de	C19orf10|E	19	Cytokines
C3	complemen	AHUS5|AR	19	Cytokines
C5	complemen	C5D|C5a|C	9	Cytokines
CALCA	calcitonin r	CALC1|CG	11	Cytokines
CALCB	calcitonin r	CALC2|CG	11	Cytokines
CAMP	cathelicidin	CAP-18|CA	3	Cytokines
CAT	catalase	-	11	Cytokines
CCK	cholecystok	-	3	Cytokines
CCL1	C-C motif c	I-309|P500|	17	Cytokines
CCL11	C-C motif c	SCYA11	17	Cytokines
CCL13	C-C motif c	CKb10|MC	17	Cytokines
CCL14	C-C motif c	CC-1|CC-3	17	Cytokines
CCL15-CC	CCL15-CC	CCL15|HC	17	Cytokines
CCL15	C-C motif c	HCC-2|HM	17	Cytokines
CCL16	C-C motif c	CKb12|HC	17	Cytokines
CCL17	C-C motif c	A-152E5.3|	16	Cytokines
CCL18	C-C motif c	AMAC-1|A	17	Cytokines
CCL19	C-C motif c	CKb11|EL	9	Cytokines
CCL2	C-C motif c	GDCF-2|H	17	Cytokines
CCL20	C-C motif c	CKb4|Exod	2	Cytokines
CCL21	C-C motif c	6Ckine|CK	9	Cytokines
CCL22	C-C motif c	A-152E5.1|	16	Cytokines
CCL23	C-C motif c	CK-BETA-	17	Cytokines
CCL24	C-C motif c	Ckb-6|MPI	7	Cytokines
CCL25	C-C motif c	Ckb15|SCY	19	Cytokines
CCL26	C-C motif c	IMAC|MIP	7	Cytokines
CCL27	C-C motif c	ALP|CTAC	9	Cytokines
CCL28	C-C motif c	CCK1|MEC	5	Cytokines
CCL3	C-C motif c	G0S19-1|L	17	Cytokines
CCL3L1	C-C motif c	464.2|D17S	17	Cytokines
CCL3P1	C-C motif c	CCL3L2|G	17	Cytokines
CCL3L3	C-C motif c	464.2|D17S	17	Cytokines
CCL4	C-C motif c	ACT2|AT7	17	Cytokines
CCL4L2	C-C motif c	AT744.2|C	17	Cytokines
CCL4L1	C-C motif c	AT744.2|C	17	Cytokines
CCL5	C-C motif c	D17S136E|	17	Cytokines
CCL7	C-C motif c	FIC|MARC	17	Cytokines
CCL8	C-C motif c	HC14|MCP	17	Cytokines
CD320	CD320 mol	8D6|8D6A|	19	Cytokines
CD40LG	CD40 ligan	CD154|CD	X	Cytokines
CD70	CD70 mole	CD27-L|CD	19	Cytokines
ADA2	adenosine d	ADGF|CEC	22	Cytokines
CER1	cerberus 1,	DAND4	9	Cytokines
CGA	glycoprotei	CG-ALPHA	6	Cytokines
CGB3	chorionic g	CGB|CGB5	19	Cytokines
CGB1	chorionic g	-	19	Cytokines
CGB2	chorionic g	-	19	Cytokines
CGB5	chorionic g	CGB|HCG|	19	Cytokines
CGB7	chorionic g	CG-beta-a|	19	Cytokines
CGB8	chorionic g	-	19	Cytokines
CHGA	chromograr	CGA	14	Cytokines
CHGB	chromograr	SCG1	20	Cytokines
CKLF	chemokine	C32|CKLF	16	Cytokines
CLCF1	cardiotroph	BSF-3|BSF	11	Cytokines
CLEC11A	C-type lecti	CLECSF3|I	19	Cytokines
CMA1	chymase 1	CYH|MCT	14	Cytokines
CMTM1	CKLF like	CKLFH|CK	16	Cytokines
CMTM2	CKLF like	CKLFSF2	16	Cytokines

CMTM3	CKLF like	BNAS2|CK	16	Cytokines
CMTM4	CKLF like	CKLFSF4	16	Cytokines
CMTM5	CKLF like	CKLFSF5	14	Cytokines
CMTM6	CKLF like	CKLFSF6|	3	Cytokines
CMTM7	CKLF like	CKLFSF7	3	Cytokines
CMTM8	CKLF like	CKLFSF8|	3	Cytokines
CNTF	ciliary neur	HCNTF	11	Cytokines
CORT	cortistatin	CST-14|CS	1	Cytokines
CRH	corticotropi	CRF|CRH1	8	Cytokines
CSF1	colony stim	CSF-1|MC	1	Cytokines
CSF2	colony stim	CSF|GMC	5	Cytokines
CSF3	colony stim	C17orf33|C	17	Cytokines
CSH1	chorionic s	CS-1|CSA|	17	Cytokines
CSH2	chorionic s	CS-2|CSB|	17	Cytokines
CSHL1	chorionic s	CS-5|CSH	17	Cytokines
CSPG5	chondroitin	NGC	3	Cytokines
CTF1	cardiotroph	CT-1|CT1	16	Cytokines
CCN2	cellular con	CTGF|HCS	6	Cytokines
CTSG	cathepsin G	CATG|CG	14	Cytokines
CX3CL1	C-X3-C mc	ABCD-3|C	16	Cytokines
CXCL1	C-X-C mot	FSP|GRO1|	4	Cytokines
CXCL10	C-X-C mot	C7|IFI10|I	4	Cytokines
CXCL11	C-X-C mot	H174|I-TA	4	Cytokines
CXCL12	C-X-C mot	IRH|PBSF|	10	Cytokines
CXCL13	C-X-C mot	ANGIE|AN	4	Cytokines
CXCL14	C-X-C mot	BMAC|BR	5	Cytokines
CXCL16	C-X-C mot	CXCLG16|	17	Cytokines
CXCL17	C-X-C mot	DMC|Dcip	19	Cytokines
CXCL2	C-X-C mot	CINC-2a|G	4	Cytokines
CXCL3	C-X-C mot	CINC-2b|G	4	Cytokines
CXCL5	C-X-C mot	ENA-78|SC	4	Cytokines
CXCL6	C-X-C mot	CKA-3|GC	4	Cytokines
CXCL9	C-X-C mot	CMK|Hum	4	Cytokines
CCN1	cellular con	CYR61|GI	1	Cytokines
DEFA1	defensin al	DEF1|DEF	8	Cytokines
DEFA3	defensin al	DEF3|HNP	8	Cytokines
DEFA5	defensin al	DEF5|HD-	8	Cytokines
DEFB1	defensin be	BD1|DEFB	8	Cytokines
DEFB103B	defensin be	BD-3|DEF	8	Cytokines
DEFB104A	defensin be	BD-4|DEF	8	Cytokines
DEFB4A	defensin be	BD-2|DEF	8	Cytokines
DKK1	dickkopf W	DKK-1|SK	10	Cytokines
EBI3	Epstein-Ba	IL-27B|IL2	19	Cytokines
EDN1	endothelin	ARCND3|	6	Cytokines
EDN2	endothelin	ET-2|ET2|	1	Cytokines
EDN3	endothelin	ET-3|ET3|	20	Cytokines
EGF	epidermal g	HOMG4|U	4	Cytokines
EPGN	epithelial m	ALGV3072	4	Cytokines
EPO	erythropoie	DBAL|EC	7	Cytokines
EREG	epiregulin	EPR|ER|Ep	4	Cytokines
ESM1	endothelial	endocan	5	Cytokines
FAM3B	FAM3 met	2-21|C21or	21	Cytokines
FAM3C	FAM3 met	GS3786|IL	7	Cytokines
FAM3D	FAM3 met	EF7|OIT1	3	Cytokines
FASLG	Fas ligand	ALPS1B|A	1	Cytokines
FGF1	fibroblast g	AFGF|ECG	5	Cytokines
FGF10	fibroblast g	-	5	Cytokines
FGF11	fibroblast g	FGF-11|FH	17	Cytokines
FGF12	fibroblast g	EIEE47|FG	3	Cytokines
FGF13	fibroblast g	FGF-13|FG	X	Cytokines

FGF14	fibroblast g	FGF-14|FH	13	Cytokines
FGF16	fibroblast g	FGF-16|MF	X	Cytokines
FGF17	fibroblast g	FGF-13|FG	8	Cytokines
FGF18	fibroblast g	FGF-18|ZF	5	Cytokines
FGF19	fibroblast g	-	11	Cytokines
FGF2	fibroblast g	BFGF|FGF	4	Cytokines
FGF20	fibroblast g	FGF-20|RH	8	Cytokines
FGF21	fibroblast g	-	19	Cytokines
FGF22	fibroblast g	-	19	Cytokines
FGF23	fibroblast g	ADHR|FGF	12	Cytokines
FGF3	fibroblast g	HBGF-3|IN	11	Cytokines
FGF4	fibroblast g	FGF-4|HBC	11	Cytokines
FGF5	fibroblast g	HBGF-5|Sr	4	Cytokines
FGF6	fibroblast g	HBGF-6|HS	12	Cytokines
FGF7	fibroblast g	HBGF-7|KC	15	Cytokines
FGF8	fibroblast g	AIGF|FGF-	10	Cytokines
FGF9	fibroblast g	FGF-9|GAF	13	Cytokines
VEGFD	vascular en	FIGF|VEGI	X	Cytokines
FIGNL2	fidgetin like	-	12	Cytokines
FLT3LG	fms related	FL|FLG3L|	19	Cytokines
FSHB	follicle stin	HH24	11	Cytokines
GAL	galanin and	ETL8|GAL	11	Cytokines
GALP	galanin like	-	19	Cytokines
GAST	gastrin	GAS	17	Cytokines
GCG	glucagon	GLP-1|GLP	2	Cytokines
GDF1	growth diff	CERS1|CH	19	Cytokines
GDF10	growth diff	BIP|BMP-3	10	Cytokines
GDF11	growth diff	BMP-11|BN	12	Cytokines
GDF15	growth diff	GDF-15|MI	19	Cytokines
GDF2	growth diff	BMP-9|BM	10	Cytokines
GDF3	growth diff	KFS3|MCC	12	Cytokines
GDF5	growth diff	BDA1C|BN	20	Cytokines
GDF6	growth diff	BMP-13|BN	8	Cytokines
GDF7	growth diff	BMP12	2	Cytokines
GDF9	growth diff	POF14	5	Cytokines
GDNF	glial cell de	ATF|ATF1|	5	Cytokines
GH1	growth hor	GH|GH-N|C	17	Cytokines
GH2	growth hor	GH-V|GHE	17	Cytokines
GHRH	growth hor	GHRF|GRF	20	Cytokines
GHRL	ghrelin and	MTLRP	3	Cytokines
GIP	gastric inhil	-	17	Cytokines
GKN1	gastrokine	AMP18|BR	2	Cytokines
GMFB	glia matura	GMF	14	Cytokines
GMFG	glia matura	GMF-GAM	19	Cytokines
GNRH1	gonadotrop	GNRH|GRI	8	Cytokines
GNRH2	gonadotrop	GnRH-II|LI	20	Cytokines
GPHA2	glycoprotei	A2|GPA2|Z	11	Cytokines
GPHB5	glycoprotei	B5|GPB5|Z	14	Cytokines
GPI	glucose-6-p	AMF|GNPI	19	Cytokines
GREM1	gremlin 1,	IC15DUPq|C	15	Cytokines
GREM2	gremlin 2,	ICKTSF1B2	1	Cytokines
GRN	granulin pro	CLN11|GE	17	Cytokines
GRP	gastrin rele	BN|GRP-10	18	Cytokines
GUCA2A	guanylate c	GCAP-I|GU	1	Cytokines
HAMP	hepcidin an	HEPC|HFE	19	Cytokines
HBEGF	heparin bin	DTR|DTS|I	5	Cytokines
HDGF	heparin bin	HMG1L2	1	Cytokines
HDGFL3	HDGF like	CGI-142|H	15	Cytokines
HGF	hepatocyte	DFNB39|F-	7	Cytokines
HTN3	histatin 3	HIS2|HTN2	4	Cytokines

IAPP	islet amyloi	DAP|IAP	12	Cytokines
IFNA1	interferon a	IFL|IFN|IFI	9	Cytokines
IFNA10	interferon a	IFN-alphaC	9	Cytokines
IFNA13	interferon a	-	9	Cytokines
IFNA14	interferon a	IFN-alphaH	9	Cytokines
IFNA16	interferon a	IFN-alpha-	9	Cytokines
IFNA17	interferon a	IFN-alphaI|	9	Cytokines
IFNA2	interferon a	IFN-alpha-:	9	Cytokines
IFNA21	interferon a	IFN-alphaI|	9	Cytokines
IFNA4	interferon a	IFN-alpha4	9	Cytokines
IFNA5	interferon a	IFN-alpha-:	9	Cytokines
IFNA6	interferon a	IFN-alphaK	9	Cytokines
IFNA7	interferon a	IFN-alphaJ|	9	Cytokines
IFNA8	interferon a	IFN-alphaB	9	Cytokines
IFNB1	interferon b	IFB|IFF|IFN	9	Cytokines
IFNE	interferon e	IFN-E|IFNI	9	Cytokines
IFNG	interferon g	IFG|IFI	12	Cytokines
IFNK	interferon k	IFNT1|INF.	9	Cytokines
IFNW1	interferon o	-	9	Cytokines
IGF1	insulin like	IGF|IGF-I|I	12	Cytokines
IGF2	insulin like	C11orf43|G	11	Cytokines
IL10	interleukin	CSIF|GVHI	1	Cytokines
IL11	interleukin	AGIF|IL-11	19	Cytokines
IL12A	interleukin	CLMF|IL-1	3	Cytokines
IL12B	interleukin	CLMF|CLN	5	Cytokines
IL13	interleukin	IL-13|P600	5	Cytokines
IL15	interleukin	IL-15	4	Cytokines
IL16	interleukin	LCF|NIL16	15	Cytokines
IL17A	interleukin	CTLA-8|CT	6	Cytokines
IL17B	interleukin	IL-17B|IL-:	5	Cytokines
IL17C	interleukin	CX2|IL-17C	16	Cytokines
IL17D	interleukin	IL-17D	13	Cytokines
IL17F	interleukin	CANDF6|II	6	Cytokines
IL18	interleukin	IGIF|IL-18|	11	Cytokines
IL19	interleukin	IL-10C|MD	1	Cytokines
IL1A	interleukin	IL-1 alpha|I	2	Cytokines
IL1B	interleukin	IL-1|IL1-BI	2	Cytokines
IL1F10	interleukin	FIL1-theta|	2	Cytokines
IL36RN	interleukin	FIL1|FIL1(	2	Cytokines
IL36A	interleukin	FIL1|FIL1(	2	Cytokines
IL37	interleukin	FIL1|FIL1(	2	Cytokines
IL36B	interleukin	FIL1|FIL1-	2	Cytokines
IL36G	interleukin	IL-1F9|IL-1	2	Cytokines
IL1RN	interleukin	DIRA|ICIL	2	Cytokines
IL2	interleukin	IL-2|TCGF	4	Cytokines
IL20	interleukin	IL-20|IL10I	1	Cytokines
IL21	interleukin	CVID11|IL	4	Cytokines
IL22	interleukin	IL-21|IL-22	12	Cytokines
IL23A	interleukin	IL-23|IL-23	12	Cytokines
IL24	interleukin	C49A|FISP	1	Cytokines
IL25	interleukin	IL17E	14	Cytokines
IL26	interleukin	AK155|IL-:	12	Cytokines
IL27	interleukin	IL-27|IL-27	16	Cytokines
IFNL2	interferon l	IL-28A|IL2	19	Cytokines
IFNL3	interferon l	IFN-lambda	19	Cytokines
IFNL1	interferon l	IL-29|IL29	19	Cytokines
IL3	interleukin	IL-3|MCGF	5	Cytokines
IL31	interleukin	IL-31	12	Cytokines
IL32	interleukin	IL-32alpha|	16	Cytokines
IL33	interleukin	C9orf26|DV	9	Cytokines

IL34	interleukin	C16orf77|IL	16	Cytokines
IL4	interleukin	BCGF-1|BC	5	Cytokines
IL5	interleukin	EDF|IL-5|T	5	Cytokines
IL6	interleukin	BSF-2|BSF	7	Cytokines
IL6ST	interleukin	CD130|CD	5	Cytokines
IL7	interleukin	IL-7	8	Cytokines
CXCL8	C-X-C mot	GCP-1|GCI	4	Cytokines
IL9	interleukin	HP40|IL-9|	5	Cytokines
INHA	inhibin sub	-	2	Cytokines
INHBA	inhibin sub	EDF|FRP	7	Cytokines
INHBB	inhibin sub	-	2	Cytokines
INHBC	inhibin sub	IHBC	12	Cytokines
INHBE	inhibin sub	-	12	Cytokines
INS	insulin	IDDM|IDD	11	Cytokines
INS-IGF2	INS-IGF2 r	INSIGF	11	Cytokines
INSL3	insulin like	RLF|RLNL	19	Cytokines
INSL4	insulin like	EPIL|PLAC	9	Cytokines
INSL5	insulin like	PRO182|UI	1	Cytokines
INSL6	insulin like	RIF1	9	Cytokines
JAG1	jagged canc	AGS|AGS1	20	Cytokines
JAG2	jagged canc	HJ2|SER2	14	Cytokines
FGF7P6	fibroblast g	KGFLP1	9	Cytokines
FGF7P3	fibroblast g	KGFLP2	9	Cytokines
KITLG	KIT ligand	DCUA|DFN	12	Cytokines
KL	klotho	HFTC3	13	Cytokines
LACRT	lacritin	-	12	Cytokines
LECT2	leukocyte c	chm-II|chm	5	Cytokines
LEFTY1	left-right de	LEFTB|LE	1	Cytokines
LEFTY2	left-right de	EBAF|LEF	1	Cytokines
LEP	leptin	LEPD|OB|(	7	Cytokines
LHB	luteinizing	CGB4|HH2	19	Cytokines
LIF	LIF interleu	CDF|DIA|H	22	Cytokines
LRSAM1	leucine rich	CMT2P|RI	9	Cytokines
LTA	lymphotoxi	LT|TNFB|T	6	Cytokines
LTB	lymphotoxi	TNFC|TNF	6	Cytokines
LTBP1	latent trans	-	2	Cytokines
LTBP2	latent trans	C14orf141|	14	Cytokines
LTBP3	latent trans	DASS|GPH	11	Cytokines
LTBP4	latent trans	ARCL1C|L	19	Cytokines
MDK	midkine	ARAP|MK|	11	Cytokines
MIA	MIA SH3 d	CD-RAP	19	Cytokines
MIF	macrophag	GIF|GLIF|N	22	Cytokines
MLN	motilin	-	6	Cytokines
MSTN	myostatin	GDF8|MSL	2	Cytokines
NAMPT	nicotinamic	1110035O1	7	Cytokines
NDP	norrin cysti	EVR2|FEV X		Cytokines
NENF	neudesin ne	CIR2|SCIR	1	Cytokines
NGF	nerve grow	Beta-NGF|I	1	Cytokines
NMB	neuromedir	-	15	Cytokines
NODAL	nodal grow	HTX5	10	Cytokines
CCN3	cellular con	IBP-9|IGFE	8	Cytokines
NPFF	neuropeptic	FMRFAL	12	Cytokines
NPPA	natriuretic p	ANF|ANP|A	1	Cytokines
NPPB	natriuretic p	BNP	1	Cytokines
NPPC	natriuretic p	CNP|CNP2	2	Cytokines
NPY	neuropeptic	PYY4	7	Cytokines
NRG1	neuregulin	ARIA|GGF	8	Cytokines
NRG2	neuregulin	DON1|HRC	5	Cytokines
NRG3	neuregulin	HRG3|pro-	10	Cytokines
NRG4	neuregulin	HRG4	15	Cytokines

NRTN	neurturin	NTN	19	Cytokines
NTF3	neurotrophi	HDNF|NG	12	Cytokines
NTF4	neurotrophi	GLC10|GL	19	Cytokines
NTS	neurotensin	NMN-125|	12	Cytokines
NUDT6	nudix hydr	ASFGF2|F	4	Cytokines
OGN	osteoglycin	OG|OIF|SL	9	Cytokines
OSGIN1	oxidative st	BDGI|OKL	16	Cytokines
OSM	oncostatin	-	22	Cytokines
OSTN	osteocrin	MUSCLIN	3	Cytokines
OXT	oxytocin/ne	OT|OT-NP	20	Cytokines
ENDOU	endonuclea	P11|PP11|P	12	Cytokines
PDGFA	platelet der	PDGF-A|P	7	Cytokines
PDGFB	platelet der	IBGC5|PD	22	Cytokines
PDGFC	platelet der	FALLOTE	4	Cytokines
PDGFD	platelet der	IEGF|MST	11	Cytokines
PDGFRA	platelet der	CD140A|P	4	Cytokines
PDGFRB	platelet der	CD140B|IB	5	Cytokines
PDGFRL	platelet der	PDGRL|PR	8	Cytokines
PDYN	prodynorph	ADCA|PE	20	Cytokines
PENK	proenkepha	PE|PENK-	8	Cytokines
PF4	platelet fact	CXCL4|PF	4	Cytokines
PF4V1	platelet fact	CXCL4L1|	4	Cytokines
PGF	placental gr	D12S1900|	14	Cytokines
PLAU	plasminoge	ATF|BDPL	10	Cytokines
PMCH	pro-melani	MCH|ppM	12	Cytokines
PNOC	prepronoci	N/OFQ|NO	8	Cytokines
POMC	proopiomel	ACTH|CLI	2	Cytokines
PPBP	pro-platelet	B-TG1|Bet	4	Cytokines
PPBPP1	pro-platelet	PPBPL1|T	4	Cytokines
PPBPP2	pro-platelet	PPBPL2|S	4	Cytokines
PPY	pancreatic	PNP|PP	17	Cytokines
PRL	prolactin	GHA1	6	Cytokines
PRLH	prolactin re	PRH|PRRP	2	Cytokines
PROK1	prokineticir	EGVEGF|P	1	Cytokines
PROK2	prokineticir	BV8|HH4|	3	Cytokines
PSPN	persephin	PSP	19	Cytokines
PTH	parathyroid	FIH1|PTH1	11	Cytokines
PTH2	parathyroid	TIP39	19	Cytokines
PTHLH	parathyroid	BDE2|HH	12	Cytokines
PTN	pleiotrophi	HARP|HB-	7	Cytokines
PYY	peptide YY	PYY-I|PY	17	Cytokines
QRFP	pyroglutam	26RFa|P51	9	Cytokines
RABEP1	rabaptin, R.	RAB5EP|R	17	Cytokines
RABEP2	rabaptin, R.	FRA	16	Cytokines
REG1A	regeneratin	ICRF|P19|P	2	Cytokines
RETN	resistin	ADSF|FIZ	19	Cytokines
RETNLB	resistin like	FIZZ1|FIZ	3	Cytokines
RLN1	relaxin 1	H1|H1RLX	9	Cytokines
RLN2	relaxin 2	H2|H2-RL	9	Cytokines
RLN3	relaxin 3	H3|RXN3|	19	Cytokines
RNASE2	ribonucleas	EDN|RAF3	14	Cytokines
S100A6	S100 calciu	2A9|5B10|	1	Cytokines
SAA1	serum amyl	PIG4|SAA|	11	Cytokines
SAA2	serum amyl	SAA|SAA1	11	Cytokines
SBDS	SBDS ribos	CGI-97|SD	7	Cytokines
SCG2	secretogran	CHGC|EM	2	Cytokines
SCGB3A1	secretoglob	HIN-1|HIN	5	Cytokines
SCT	secretin	-	11	Cytokines
AIMP1	aminoacyl t	EMAP2|EM	4	Cytokines
SECTM1	secreted an	K12|SECT	17	Cytokines

SEMA3A	semaphorin	COLL1|HH	7	Cytokines
SEMA3B	semaphorin	LUCA-1|SE	3	Cytokines
SEMA3C	semaphorin	SEMAE|Se	7	Cytokines
SEMA3D	semaphorin	Sema-Z2|co	7	Cytokines
SEMA3E	semaphorin	M-SEMAH	7	Cytokines
SEMA3F	semaphorin	SEMA-IV|S	3	Cytokines
SEMA3G	semaphorin	sem2	3	Cytokines
SEMA4A	semaphorin	CORD10|R	1	Cytokines
SEMA4B	semaphorin	SEMAC|Se	15	Cytokines
SEMA4C	semaphorin	M-SEMA-F	2	Cytokines
SEMA4D	semaphorin	A8|BB18|C	9	Cytokines
SEMA4F	ssemaphori	M-SEMA|P	2	Cytokines
SEMA4G	semaphorin	-	10	Cytokines
SEMA5A	semaphorin	SEMAF|ser	5	Cytokines
SEMA5B	semaphorin	SEMAG|Se	3	Cytokines
SEMA6A	semaphorin	HT018|SEM	5	Cytokines
SEMA6B	semaphorin	EPM11|SEI	19	Cytokines
SEMA6C	semaphorin	SEMAY|m-	1	Cytokines
SEMA6D	semaphorin	-	15	Cytokines
SEMA7A	semaphorin	CD108|CD	15	Cytokines
SLIT1	slit guidanc	MEGF4|SL	10	Cytokines
SLIT2	slit guidanc	SLIL3|Slit-	4	Cytokines
SLURP1	secreted LY	ANUP|ARS	8	Cytokines
SPP1	secreted ph	BNSP|BSP	4	Cytokines
SST	somatostati	SMST	3	Cytokines
STC1	stanniocalc	STC	8	Cytokines
STC2	stanniocalc	STC-2|STC	5	Cytokines
TAC1	tachykinin p	Hs.2563|NK	7	Cytokines
TDGF1	teratocarcin	CR|CR-1|C	3	Cytokines
TDGF1P3	teratocarcin	CR-3|CRIP	X	Cytokines
TG	thyroglobul	AITD3|TGI	8	Cytokines
TGFA	transformin	TFGA	2	Cytokines
TGFB1	transformin	CED|DPD1	19	Cytokines
TGFB2	transformin	G-TSF|LDS	1	Cytokines
TGFB3	transformin	ARVD|ARV	14	Cytokines
THPO	thrombopoi	MGDF|MK	3	Cytokines
TNC	tenascin C	150-225|DF	9	Cytokines
TNF	tumor necro	DIF|TNF-al	6	Cytokines
TNFRSF11	TNF recept	OCIF|OPG|	8	Cytokines
TNFSF10	TNF superf	APO2L|Ap	3	Cytokines
TNFSF11	TNF superf	CD254|OD	13	Cytokines
TNFSF12	TNF superf	APO3L|DR	17	Cytokines
TNFSF13	TNF superf	APRIL|CD	17	Cytokines
TNFSF13B	TNF superf	BAFF|BLY	13	Cytokines
TNFSF14	TNF superf	CD258|HV	19	Cytokines
TNFSF15	TNF superf	TL1|TL1A|	9	Cytokines
TNFSF18	TNF superf	AITRL|GIT	1	Cytokines
TNFSF4	TNF superf	CD134L|CI	1	Cytokines
TNFSF8	TNF superf	CD153|CD	9	Cytokines
TNFSF9	TNF superf	4-1BB-L|C	19	Cytokines
TOR2A	torsin famil	TORP1	9	Cytokines
TRH	thyrotropin	Pro-TRH|T	3	Cytokines
TSHB	thyroid stin	TSH-B|TSH	1	Cytokines
TSLP	thymic stro	-	5	Cytokines
TXLNA	taxilin alph	IL14|TXLN	1	Cytokines
TYMP	thymidine p	ECGF|ECG	22	Cytokines
UCN	urocortin	UI|UROC	2	Cytokines
UCN2	urocortin 2	SRP|UCN-I	3	Cytokines
UCN3	urocortin 3	SCP|SPC|U	10	Cytokines
UTS2	urotensin 2	PRO1068|U	1	Cytokines

UTS2B	urotensin 2	U2B|URP|U		3	Cytokines
VEGFA	vascular en	MVCD1|VI		6	Cytokines
VEGFB	vascular en	VEGFL|VR		11	Cytokines
VEGFC	vascular en	Flt4-L|LMI		4	Cytokines
VGF	VGF nerve	SCG7|SgVI		7	Cytokines
VIP	vasoactive i	PHM27		6	Cytokines
XCL1	X-C motif c	ATAC|LPT		1	Cytokines
XCL2	X-C motif c	SCM-1b|SC		1	Cytokines
ACVR1B	activin A re	ACTRIB|A		12	Cytokine_Receptors
ACVR1C	activin A re	ACVRLK7		2	Cytokine_Receptors
ACVR2A	activin A re	ACTRII|AC		2	Cytokine_Receptors
ACVR2B	activin A re	ACTRIIB|A		3	Cytokine_Receptors
ACVRL1	activin A re	ACVRLK1		12	Cytokine_Receptors
ADCYAP1	ADCYAP r	PAC1|PAC		7	Cytokine_Receptors
ADIPOR1	adiponectin	ACDCR1|C		1	Cytokine_Receptors
ADIPOR2	adiponectin	ACDCR2|P		12	Cytokine_Receptors
ADRB1	adrenocepto	ADRB1R|B		10	Cytokine_Receptors
ADRB2	adrenocepto	ADRB2R|A		5	Cytokine_Receptors
AGTR1	angiotensin	AG2S|AGT		3	Cytokine_Receptors
AGTR2	angiotensin	AT2|ATGR	X		Cytokine_Receptors
AMHR2	anti-Muller	AMHR|MIS		12	Cytokine_Receptors
ANGPT1	angiopoieti	AGP1|AGP		8	Cytokine_Receptors
ANGPT4	angiopoieti	ANG3|ANC		20	Cytokine_Receptors
ANGPTL1	angiopoieti	ANG3|ANC		1	Cytokine_Receptors
ANGPTL2	angiopoieti	ARP2|HAR		9	Cytokine_Receptors
ANGPTL3	angiopoieti	ANG-5|AN		1	Cytokine_Receptors
ANGPTL4	angiopoieti	ARP4|FIAF		19	Cytokine_Receptors
ANGPTL6	angiopoieti	AGF|ARP5		19	Cytokine_Receptors
APLNR	apelin recep	AGTRL1|A		11	Cytokine_Receptors
AR	androgen re	AIS|AR8|D	X		Cytokine_Receptors
AVPR1A	arginine vas	AVPR V1a		12	Cytokine_Receptors
AVPR1B	arginine vas	AVPR3|V1		1	Cytokine_Receptors
AVPR2	arginine vas	ADHR|DI1	X		Cytokine_Receptors
BMPR1A	bone morph	10q23del|A		10	Cytokine_Receptors
BMPR1B	bone morph	ALK-6|ALI		4	Cytokine_Receptors
BMPR2	bone morph	BMPR-II|B		2	Cytokine_Receptors
BRD8	bromodoma	SMAP|SMA		5	Cytokine_Receptors
C3AR1	complemen	AZ3B|C3A		12	Cytokine_Receptors
C5AR1	complemen	C5A|C5AR		19	Cytokine_Receptors
CALCR	calcitonin r	CRT|CT-R|		7	Cytokine_Receptors
CALCRL	calcitonin r	CGRPR|CR		2	Cytokine_Receptors
ACKR2	atypical che	CCBP2|CC		3	Cytokine_Receptors
CCR1	C-C motif c	CD191|CKI		3	Cytokine_Receptors
CCR10	C-C motif c	GPR2		17	Cytokine_Receptors
CCR3	C-C motif c	C C CKR3|		3	Cytokine_Receptors
CCR4	C-C motif c	CC-CKR-4		3	Cytokine_Receptors
CCR5	C-C motif c	CC-CKR-5		3	Cytokine_Receptors
CCR6	C-C motif c	BN-1|C-C C		6	Cytokine_Receptors
CCR7	C-C motif c	BLR2|CC-C		17	Cytokine_Receptors
CCR8	C-C motif c	CC-CKR-8		3	Cytokine_Receptors
CCR9	C-C motif c	CC-CKR-9		3	Cytokine_Receptors
ACKR4	atypical che	CC-CKR-1		3	Cytokine_Receptors
CCRL2	C-C motif c	ACKR5|CK		3	Cytokine_Receptors
CD40	CD40 mole	Bp50|CDW		20	Cytokine_Receptors
CMKLR1	chemerin cl	CHEMERI		12	Cytokine_Receptors
CNTFR	ciliary neur	-		9	Cytokine_Receptors
CRHR1	corticotropi	CRF-R|CRI		17	Cytokine_Receptors
CRHR2	corticotropi	CRF-RB|CI		7	Cytokine_Receptors
CRIM1	cysteine ric	CRIM-1|S5		2	Cytokine_Receptors
CRLF1	cytokine re	CISS|CISS		19	Cytokine_Receptors

<table>
<tr><td></td><td></td><td></td><td></td><td></td></tr>
<tr><td>CRLF2</td><td>cytokine re</td><td>CRL2|CRL</td><td>X|Y</td><td>Cytokine_Receptors</td></tr>
<tr><td>CRLF3</td><td>cytokine re</td><td>CREME-9|</td><td>17</td><td>Cytokine_Receptors</td></tr>
<tr><td>CSF1R</td><td>colony stim</td><td>BANDDOS</td><td>5</td><td>Cytokine_Receptors</td></tr>
<tr><td>CSF2RA</td><td>colony stim</td><td>CD116|CD</td><td>X|Y</td><td>Cytokine_Receptors</td></tr>
<tr><td>CSF2RB</td><td>colony stim</td><td>CD131|CD</td><td>22</td><td>Cytokine_Receptors</td></tr>
<tr><td>CSF3R</td><td>colony stim</td><td>CD114|GC</td><td>1</td><td>Cytokine_Receptors</td></tr>
<tr><td>CX3CR1</td><td>C-X3-C mo</td><td>CCRL1|CM</td><td>3</td><td>Cytokine_Receptors</td></tr>
<tr><td>CXCR3</td><td>C-X-C mot</td><td>CD182|CD</td><td>X</td><td>Cytokine_Receptors</td></tr>
<tr><td>CXCR4</td><td>C-X-C mot</td><td>CD184|D2S</td><td>2</td><td>Cytokine_Receptors</td></tr>
<tr><td>CXCR5</td><td>C-X-C mot</td><td>BLR1|CD1</td><td>11</td><td>Cytokine_Receptors</td></tr>
<tr><td>CXCR6</td><td>C-X-C mot</td><td>BONZO|CI</td><td>3</td><td>Cytokine_Receptors</td></tr>
<tr><td>ACKR3</td><td>atypical che</td><td>CMKOR1|</td><td>2</td><td>Cytokine_Receptors</td></tr>
<tr><td>CYSLTR1</td><td>cysteinyl le</td><td>CYSLT1|C</td><td>X</td><td>Cytokine_Receptors</td></tr>
<tr><td>CYSLTR2</td><td>cysteinyl le</td><td>CYSLT2|C</td><td>13</td><td>Cytokine_Receptors</td></tr>
<tr><td>ACKR1</td><td>atypical che</td><td>CCBP1|CD</td><td>1</td><td>Cytokine_Receptors</td></tr>
<tr><td>EDNRA</td><td>endothelin</td><td>ET-A|ETA|</td><td>4</td><td>Cytokine_Receptors</td></tr>
<tr><td>EDNRB</td><td>endothelin</td><td>ABCDS|ET</td><td>13</td><td>Cytokine_Receptors</td></tr>
<tr><td>EGFR</td><td>epidermal g</td><td>ERBB|ERB</td><td>7</td><td>Cytokine_Receptors</td></tr>
<tr><td>ENG</td><td>endoglin</td><td>END|HHT1</td><td>9</td><td>Cytokine_Receptors</td></tr>
<tr><td>EPOR</td><td>erythropoie</td><td>EPO-R</td><td>19</td><td>Cytokine_Receptors</td></tr>
<tr><td>ESR1</td><td>estrogen re</td><td>ER|ESR|ES</td><td>6</td><td>Cytokine_Receptors</td></tr>
<tr><td>ESR2</td><td>estrogen re</td><td>ER-BETA|</td><td>14</td><td>Cytokine_Receptors</td></tr>
<tr><td>ESRRA</td><td>estrogen rel</td><td>ERR1|ERR</td><td>11</td><td>Cytokine_Receptors</td></tr>
<tr><td>ESRRB</td><td>estrogen rel</td><td>DFNB35|E</td><td>14</td><td>Cytokine_Receptors</td></tr>
<tr><td>ESRRG</td><td>estrogen rel</td><td>ERR-gamm</td><td>1</td><td>Cytokine_Receptors</td></tr>
<tr><td>FGFR1</td><td>fibroblast g</td><td>BFGFR|CD</td><td>8</td><td>Cytokine_Receptors</td></tr>
<tr><td>FGFR2</td><td>fibroblast g</td><td>BBDS|BEK</td><td>10</td><td>Cytokine_Receptors</td></tr>
<tr><td>FGFR3</td><td>fibroblast g</td><td>ACH|CD33</td><td>4</td><td>Cytokine_Receptors</td></tr>
<tr><td>FGFR4</td><td>fibroblast g</td><td>CD334|JTK</td><td>5</td><td>Cytokine_Receptors</td></tr>
<tr><td>FGFRL1</td><td>fibroblast g</td><td>FGFR-5|FC</td><td>4</td><td>Cytokine_Receptors</td></tr>
<tr><td>FLT1</td><td>fms related</td><td>FLT|FLT-1</td><td>13</td><td>Cytokine_Receptors</td></tr>
<tr><td>FLT3</td><td>fms related</td><td>CD135|FLK</td><td>13</td><td>Cytokine_Receptors</td></tr>
<tr><td>FLT4</td><td>fms related</td><td>CHTD7|FL</td><td>5</td><td>Cytokine_Receptors</td></tr>
<tr><td>FPR1</td><td>formyl pept</td><td>FMLP|FPR</td><td>19</td><td>Cytokine_Receptors</td></tr>
<tr><td>FPR2</td><td>formyl pept</td><td>ALXR|FMI</td><td>19</td><td>Cytokine_Receptors</td></tr>
<tr><td>FPR2</td><td>formyl pept</td><td>ALXR|FMI</td><td>19</td><td>Cytokine_Receptors</td></tr>
<tr><td>FSHR</td><td>follicle stin</td><td>FSHR1|FSI</td><td>2</td><td>Cytokine_Receptors</td></tr>
<tr><td>GALR2</td><td>galanin rece</td><td>GAL2-R|G</td><td>17</td><td>Cytokine_Receptors</td></tr>
<tr><td>GALR3</td><td>galanin rece</td><td>-</td><td>22</td><td>Cytokine_Receptors</td></tr>
<tr><td>GCGR</td><td>glucagon re</td><td>GGR|GL-R</td><td>17</td><td>Cytokine_Receptors</td></tr>
<tr><td>GHR</td><td>growth hor</td><td>GHBP|GHI</td><td>5</td><td>Cytokine_Receptors</td></tr>
<tr><td>GHRHR</td><td>growth hor</td><td>GHRFR|GF</td><td>7</td><td>Cytokine_Receptors</td></tr>
<tr><td>GHSR</td><td>growth hor</td><td>GHDP</td><td>3</td><td>Cytokine_Receptors</td></tr>
<tr><td>GIPR</td><td>gastric inhil</td><td>PGQTL2</td><td>19</td><td>Cytokine_Receptors</td></tr>
<tr><td>GLP1R</td><td>glucagon lil</td><td>GLP-1|GLI</td><td>6</td><td>Cytokine_Receptors</td></tr>
<tr><td>GLP2R</td><td>glucagon lil</td><td>-</td><td>17</td><td>Cytokine_Receptors</td></tr>
<tr><td>GNRHR</td><td>gonadotrop</td><td>GNRHR1|</td><td>4</td><td>Cytokine_Receptors</td></tr>
<tr><td>GPER1</td><td>G protein-c</td><td>CEPR|CMK</td><td>7</td><td>Cytokine_Receptors</td></tr>
<tr><td>GPR17</td><td>G protein-c</td><td>-</td><td>2</td><td>Cytokine_Receptors</td></tr>
<tr><td>GPR32</td><td>G protein-c</td><td>RVDR1</td><td>19</td><td>Cytokine_Receptors</td></tr>
<tr><td>GPR33</td><td>G protein-c</td><td>-</td><td>14</td><td>Cytokine_Receptors</td></tr>
<tr><td>PTGDR2</td><td>prostagland</td><td>CD294|CR</td><td>11</td><td>Cytokine_Receptors</td></tr>
<tr><td>C5AR2</td><td>complemen</td><td>C5L2|GPF</td><td>19</td><td>Cytokine_Receptors</td></tr>
<tr><td>HNF4A</td><td>hepatocyte</td><td>FRTS4|HN</td><td>20</td><td>Cytokine_Receptors</td></tr>
<tr><td>HNF4G</td><td>hepatocyte</td><td>NR2A2|NR</td><td>8</td><td>Cytokine_Receptors</td></tr>
<tr><td>HTR3A</td><td>5-hydroxyt</td><td>5-HT-3|5-H</td><td>11</td><td>Cytokine_Receptors</td></tr>
<tr><td>HTR3B</td><td>5-hydroxyt</td><td>5-HT3B</td><td>11</td><td>Cytokine_Receptors</td></tr>
<tr><td>HTR3C</td><td>5-hydroxyt</td><td>-</td><td>3</td><td>Cytokine_Receptors</td></tr>
<tr><td>HTR3D</td><td>5-hydroxyt</td><td>5HT3D</td><td>3</td><td>Cytokine_Receptors</td></tr>
<tr><td>HTR3E</td><td>5-hydroxyt</td><td>5-HT3-E|5-</td><td>3</td><td>Cytokine_Receptors</td></tr>
</table>

IFNAR1	interferon a	AVP|IFN-a	21	Cytokine_Receptors
IFNAR2	interferon a	IFN-R|IFN-	21	Cytokine_Receptors
IFNGR1	interferon g	CD119|IFN	6	Cytokine_Receptors
IFNGR2	interferon g	AF-1|IFGR	21	Cytokine_Receptors
IGF1R	insulin like	CD221|IGF	15	Cytokine_Receptors
IGF2R	insulin like	CD222|CI-I	6	Cytokine_Receptors
IL10RA	interleukin	CD210|CD	11	Cytokine_Receptors
IL10RB	interleukin	CDW210B|	21	Cytokine_Receptors
IL11RA	interleukin	CRSDA	9	Cytokine_Receptors
IL12RB1	interleukin	CD212|IL-1	19	Cytokine_Receptors
IL12RB2	interleukin	-	1	Cytokine_Receptors
IL13RA1	interleukin	CD213A1|(	X	Cytokine_Receptors
IL13RA2	interleukin	CD213A2|(	X	Cytokine_Receptors
IL15RA	interleukin	CD215	10	Cytokine_Receptors
IL2RB	interleukin	CD122|IL1	22	Cytokine_Receptors
IL17RA	interleukin	CANDF5|C	22	Cytokine_Receptors
IL17RB	interleukin	CRL4|EVI2	3	Cytokine_Receptors
IL17RC	interleukin	CANDF9|II	3	Cytokine_Receptors
IL17RD	interleukin	HH18|IL-1	3	Cytokine_Receptors
IL17RE	interleukin	-	3	Cytokine_Receptors
IL18R1	interleukin	CD218a|CI	2	Cytokine_Receptors
IL18RAP	interleukin	ACPL|CD2	2	Cytokine_Receptors
IL1R1	interleukin	CD121A|D	2	Cytokine_Receptors
IL1R2	interleukin	CD121b|CI	2	Cytokine_Receptors
IL1RAP	interleukin	C3orf13|IL-	3	Cytokine_Receptors
IL1RL1	interleukin	DER4|FIT-	2	Cytokine_Receptors
IL1RL2	interleukin	IL-1Rrp2|II	2	Cytokine_Receptors
IL20RA	interleukin	CRF2-8|IL-	6	Cytokine_Receptors
IL20RB	interleukin	DIRS1|FNI	3	Cytokine_Receptors
IL21R	interleukin	CD360|IMI	16	Cytokine_Receptors
IL22RA1	interleukin	CRF2-9|IL2	1	Cytokine_Receptors
IL22RA2	interleukin	CRF2-10|C	6	Cytokine_Receptors
IL23R	interleukin	-	1	Cytokine_Receptors
IL27RA	interleukin	CRL1|IL-2	19	Cytokine_Receptors
IFNLR1	interferon la	CRF2/12|IF	1	Cytokine_Receptors
IL2RA	interleukin	CD25|IDDI	10	Cytokine_Receptors
IL2RB	interleukin	CD122|IL1	22	Cytokine_Receptors
IL2RG	interleukin	CD132|CID	X	Cytokine_Receptors
IL31RA	interleukin	CRL|CRL3	5	Cytokine_Receptors
IL3RA	interleukin	CD123|IL3	X|Y	Cytokine_Receptors
IL4R	interleukin	CD124|IL-4	16	Cytokine_Receptors
IL5RA	interleukin	CD125|CD	3	Cytokine_Receptors
IL6R	interleukin	CD126|IL-6	1	Cytokine_Receptors
IL7R	interleukin	CD127|CD	5	Cytokine_Receptors
CXCR1	C-X-C mot	C-C|C-C-C	2	Cytokine_Receptors
CXCR2	C-X-C mot	CD182|CD	2	Cytokine_Receptors
IL9R	interleukin	CD129|IL-9	X|Y	Cytokine_Receptors
INSR	insulin rece	CD220|HH	19	Cytokine_Receptors
KDR	kinase inser	CD309|FLK	4	Cytokine_Receptors
LEPR	leptin recep	CD295|LEF	1	Cytokine_Receptors
LGR4	leucine rich	BNMD17|C	11	Cytokine_Receptors
LGR5	leucine rich	FEX|GPR4	12	Cytokine_Receptors
LGR6	leucine rich	GPCR|VTS	1	Cytokine_Receptors
LHCGR	luteinizing	HHG|LCGI	2	Cytokine_Receptors
LIFR	LIF recepto	CD118|LIF	5	Cytokine_Receptors
LTB4R	leukotriene	BLT1|BLT	14	Cytokine_Receptors
LTB4R2	leukotriene	BLT2|BLT	14	Cytokine_Receptors
LTBR	lymphotoxi	D12S370|L	12	Cytokine_Receptors
MC1R	melanocort	CMM5|MS	16	Cytokine_Receptors
MC2R	melanocort	ACTHR	18	Cytokine_Receptors

MC3R	melanocort	BMIQ9|MC	20	Cytokine_Receptors
MC4R	melanocort	BMIQ20	18	Cytokine_Receptors
MCHR1	melanin co	GPR24|MC	22	Cytokine_Receptors
MCHR2	melanin co	GPR145|GI	6	Cytokine_Receptors
MET	MET proto-	AUTS9|DF	7	Cytokine_Receptors
MLNR	motilin rece	GPR38|MT	13	Cytokine_Receptors
MPL	MPL proto-	C-MPL|CD	1	Cytokine_Receptors
MTNR1A	melatonin r	MEL-1A-R	4	Cytokine_Receptors
MTNR1B	melatonin r	FGQTL2|M	11	Cytokine_Receptors
NGFR	nerve grow	CD271|Gp8	17	Cytokine_Receptors
NMBR	neuromedir	BB1|BB1R	6	Cytokine_Receptors
NPR1	natriuretic p	ANPRA|AN	1	Cytokine_Receptors
NPR3	natriuretic p	ANP-C|AN	5	Cytokine_Receptors
NR0B1	nuclear rece	AHC|AHC1	X	Cytokine_Receptors
NR0B2	nuclear rece	SHP|SHP1	1	Cytokine_Receptors
NR1D1	nuclear rece	EAR1|REV	17	Cytokine_Receptors
NR1D2	nuclear rece	BD73|EAR	3	Cytokine_Receptors
NR1H2	nuclear rece	LXR-b|LXI	19	Cytokine_Receptors
NR1H3	nuclear rece	LXR-a|LXI	11	Cytokine_Receptors
NR1H4	nuclear rece	BAR|FXR|I	12	Cytokine_Receptors
NR1I2	nuclear rece	BXR|ONR1	3	Cytokine_Receptors
NR1I3	nuclear rece	CAR|CAR1	1	Cytokine_Receptors
NR2C1	nuclear rece	TR2	12	Cytokine_Receptors
NR2C2	nuclear rece	TAK1|TR4	3	Cytokine_Receptors
NR2E1	nuclear rece	TLL|TLX|X	6	Cytokine_Receptors
NR2E3	nuclear rece	ESCS|PNR	15	Cytokine_Receptors
NR2F1	nuclear rece	BBOAS|BE	5	Cytokine_Receptors
NR2F2	nuclear rece	ARP-1|ARI	15	Cytokine_Receptors
NR2F6	nuclear rece	EAR-2|EAI	19	Cytokine_Receptors
NR3C1	nuclear rece	GCCR|GCI	5	Cytokine_Receptors
NR3C2	nuclear rece	MCR|MLR	4	Cytokine_Receptors
NR4A1	nuclear rece	GFRP1|HM	12	Cytokine_Receptors
NR4A2	nuclear rece	HZF-3|NOT	2	Cytokine_Receptors
NR4A3	nuclear rece	CHN|CSMI	9	Cytokine_Receptors
NR5A1	nuclear rece	AD4BP|EL	9	Cytokine_Receptors
NR5A2	nuclear rece	B1F|B1F2|(	1	Cytokine_Receptors
NR6A1	nuclear rece	CT150|GCI	9	Cytokine_Receptors
NRP1	neuropilin 1	BDCA4|CD	10	Cytokine_Receptors
NRP2	neuropilin 2	NP2|NPN2|	2	Cytokine_Receptors
OGFR	opioid grow	-	20	Cytokine_Receptors
OPRD1	opioid rece	DOP|DOR|	1	Cytokine_Receptors
OPRK1	opioid rece	K-OR-1|KC	8	Cytokine_Receptors
OPRL1	opioid relat	KOR-3|KO	20	Cytokine_Receptors
OPRM1	opioid rece	LMOR|M-(	6	Cytokine_Receptors
OSMR	oncostatin I	IL-31R-bet	5	Cytokine_Receptors
OXTR	oxytocin re	OT-R	3	Cytokine_Receptors
PGR	progesteron	NR3C3|PR	11	Cytokine_Receptors
PGRMC2	progesteron	DG6|PMBI	4	Cytokine_Receptors
PLAUR	plasminoge	CD87|U-PA	19	Cytokine_Receptors
PLXNA1	plexin A1	NOV|NOV	3	Cytokine_Receptors
PLXNA2	plexin A2	OCT|PLXN	1	Cytokine_Receptors
PLXNA3	plexin A3	6.3|HSSEX	X	Cytokine_Receptors
PLXNA4	plexin A4	FAYV2820	7	Cytokine_Receptors
PLXNB1	plexin B1	PLEXIN-B	3	Cytokine_Receptors
PLXNB2	plexin B2	MM1|Nbla(	22	Cytokine_Receptors
PLXNB3	plexin B3	PLEXB3|PI	X	Cytokine_Receptors
PLXNC1	plexin C1	CD232|PLX	12	Cytokine_Receptors
PLXND1	plexin D1	PLEXD1	3	Cytokine_Receptors
PPARA	peroxisome	NR1C1|PPA	22	Cytokine_Receptors
PPARD	peroxisome	FAAR|NR1	6	Cytokine_Receptors

PPARG	peroxisome	CIMT1|GL	3	Cytokine_Receptors
PRLHR	prolactin re	GPR10|GR	10	Cytokine_Receptors
PRLR	prolactin re	HPRL|MFA	5	Cytokine_Receptors
PTAFR	platelet acti	PAFR	1	Cytokine_Receptors
PTGDR	prostagland	AS1|ASRT	14	Cytokine_Receptors
PTGDS	prostagland	L-PGDS|LI	9	Cytokine_Receptors
PTGER1	prostagland	EP1	19	Cytokine_Receptors
PTGER2	prostagland	EP2	14	Cytokine_Receptors
PTGER3	prostagland	EP3|EP3-I|I	1	Cytokine_Receptors
PTGER4	prostagland	EP4|EP4R	5	Cytokine_Receptors
PTGFR	prostagland	FP	1	Cytokine_Receptors
PTH1R	parathyroid	EKNS|PFE	3	Cytokine_Receptors
PTH2R	parathyroid	PTHR2	2	Cytokine_Receptors
RARA	retinoic aci	NR1B1|RA	17	Cytokine_Receptors
RARB	retinoic aci	HAP|MCO	3	Cytokine_Receptors
RARG	retinoic aci	NR1B3|RA	12	Cytokine_Receptors
ROBO1	roundabout	DUTT1|SA	3	Cytokine_Receptors
ROBO2	roundabout	SAX3	3	Cytokine_Receptors
ROBO3	roundabout	HGPPS|HG	11	Cytokine_Receptors
RORA	RAR relate	IDDECA|N	15	Cytokine_Receptors
RORB	RAR relate	EIG15|NR1	9	Cytokine_Receptors
RORC	RAR relate	IMD42|NR	1	Cytokine_Receptors
RXFP1	relaxin fam	LGR7|RXF	4	Cytokine_Receptors
RXFP2	relaxin fam	GPR106|GI	13	Cytokine_Receptors
RXFP3	relaxin fam	GPCR135|I	5	Cytokine_Receptors
RXRA	retinoid X r	NR2B1	9	Cytokine_Receptors
RXRB	retinoid X r	DAUDI6|H	6	Cytokine_Receptors
RXRG	retinoid X r	NR2B3|RX	1	Cytokine_Receptors
S1PR1	sphingosine	CD363|CH	1	Cytokine_Receptors
S1PR2	sphingosine	AGR16|DF	19	Cytokine_Receptors
SCTR	secretin rec	SR	2	Cytokine_Receptors
SDC1	syndecan 1	CD138|SD	2	Cytokine_Receptors
SDC2	syndecan 2	CD362|HSI	8	Cytokine_Receptors
SDC3	syndecan 3	SDCN|SYN	1	Cytokine_Receptors
SDC4	syndecan 4	SYND4	20	Cytokine_Receptors
SORT1	sortilin 1	Gp95|LDL	1	Cytokine_Receptors
SSTR1	somatostati	SRIF-2|SS-	14	Cytokine_Receptors
SSTR2	somatostati	-	17	Cytokine_Receptors
SSTR5	somatostati	SS-5-R	16	Cytokine_Receptors
ST2	-	-	11	Cytokine_Receptors
TACR1	tachykinin r	NK1R|NKI	2	Cytokine_Receptors
TEK	TEK recept	CD202B|G	9	Cytokine_Receptors
TGFBR1	transformin	AAT5|ACV	9	Cytokine_Receptors
TGFBR2	transformin	AAT3|FAA	3	Cytokine_Receptors
TGFBR3	transformin	BGCAN|be	1	Cytokine_Receptors
THRA	thyroid hor	AR7|CHNC	17	Cytokine_Receptors
THRB	thyroid hor	C-ERBA-2|	3	Cytokine_Receptors
TIE1	tyrosine kir	JTK14|TIE	1	Cytokine_Receptors
TNFRSF10	TNF recept	APO2|CD2	8	Cytokine_Receptors
TNFRSF10	TNF recept	CD262|DR	8	Cytokine_Receptors
TNFRSF10	TNF recept	CD263|DC	8	Cytokine_Receptors
TNFRSF10	TNF recept	CD264|DC	8	Cytokine_Receptors
TNFRSF11	TNF recept	CD265|FE	18	Cytokine_Receptors
TNFRSF12	TNF recept	CD266|FN	16	Cytokine_Receptors
TNFRSF13	TNF recept	CD267|CV	17	Cytokine_Receptors
TNFRSF13	TNF recept	BAFF-R|BA	22	Cytokine_Receptors
TNFRSF14	TNF recept	ATAR|CD2	1	Cytokine_Receptors
TNFRSF17	TNF recept	BCM|BCM	16	Cytokine_Receptors
TNFRSF18	TNF recept	AITR|CD3	1	Cytokine_Receptors
TNFRSF19	TNF recept	TAJ|TAJ-al	13	Cytokine_Receptors

TNFRSF1A	TNF recept	CD120a|FP	12	Cytokine_Receptors
TNFRSF1B	TNF recept	CD120b|TB	1	Cytokine_Receptors
TNFRSF21	TNF recept	BM-018|CI	6	Cytokine_Receptors
TNFRSF25	TNF recept	APO-3|DD	1	Cytokine_Receptors
TNFRSF4	TNF recept	ACT35|CD	1	Cytokine_Receptors
TNFRSF6B	TNF recept	DCR3|DJ58	20	Cytokine_Receptors
TNFRSF8	TNF recept	CD30|D1S1	1	Cytokine_Receptors
TNFRSF9	TNF recept	4-1BB|CD1	1	Cytokine_Receptors
TRHR	thyrotropin	CHNG7|TR	8	Cytokine_Receptors
TSHR	thyroid stin	CHNG1|LC	14	Cytokine_Receptors
TUBB3	tubulin beta	CDCBM|C	16	Cytokine_Receptors
VDR	vitamin D r	NR1I1|PPP	12	Cytokine_Receptors
VIPR1	vasoactive i	HVR1|II|PA	3	Cytokine_Receptors
VIPR2	vasoactive i	C16DUPq3	7	Cytokine_Receptors
XCR1	X-C motif c	CCXCR1|G	3	Cytokine_Receptors
IFNA10	interferon a	IFN-alphaC	9	Interferons
IFNA13	interferon a	-	9	Interferons
IFNA14	interferon a	IFN-alphaH	9	Interferons
IFNA16	interferon a	IFN-alpha-	9	Interferons
IFNA17	interferon a	IFN-alphaI|	9	Interferons
IFNA2	interferon a	IFN-alpha-2	9	Interferons
IFNA21	interferon a	IFN-alphaI|	9	Interferons
IFNA4	interferon a	IFN-alpha4	9	Interferons
IFNA5	interferon a	IFN-alpha-3	9	Interferons
IFNA6	interferon a	IFN-alphaK	9	Interferons
IFNA7	interferon a	IFN-alphaJ|	9	Interferons
IFNA8	interferon a	IFN-alphaB	9	Interferons
IFNB1	interferon b	IFB|IFF|IFN	9	Interferons
IFNE	interferon e	IFN-E|IFNI	9	Interferons
IFNG	interferon g	IFG|IFI	12	Interferons
IFNK	interferon k	IFNT1|INF	9	Interferons
IFNW1	interferon o	-	9	Interferons
IFNAR2	interferon a	IFN-R|IFN-	21	Interferon_Receptor
IFNGR1	interferon g	CD119|IFN	6	Interferon_Receptor
IFNGR2	interferon g	AF-1|IFGR	21	Interferon_Receptor
IL11	interleukin	AGIF|IL-11	19	Interleukins
IL12A	interleukin	CLMF|IL-1	3	Interleukins
IL12B	interleukin	CLMF|CLM	5	Interleukins
IL13	interleukin	IL-13|P600	5	Interleukins
IL15	interleukin	IL-15	4	Interleukins
IL16	interleukin	LCF|NIL16	15	Interleukins
IL17A	interleukin	CTLA-8|CT	6	Interleukins
IL17B	interleukin	IL-17B|IL-2	5	Interleukins
IL17C	interleukin	CX2|IL-17C	16	Interleukins
IL17D	interleukin	IL-17D	13	Interleukins
IL17F	interleukin	CANDF6|IL	6	Interleukins
IL18	interleukin	IGIF|IL-18|	11	Interleukins
IL19	interleukin	IL-10C|MD	1	Interleukins
IL1A	interleukin	IL-1 alpha|I	2	Interleukins
IL1B	interleukin	IL-1|IL1-BI	2	Interleukins
IL1F10	interleukin	FIL1-theta|	2	Interleukins
IL36RN	interleukin	FIL1|FIL1(	2	Interleukins
IL36A	interleukin	FIL1|FIL1(	2	Interleukins
IL37	interleukin	FIL1|FIL1(	2	Interleukins
IL36B	interleukin	FIL1|FIL1-	2	Interleukins
IL36G	interleukin	IL-1F9|IL-1	2	Interleukins
IL1RN	interleukin	DIRA|ICIL	2	Interleukins
IL2	interleukin	IL-2|TCGF	4	Interleukins
IL20	interleukin	IL-20|IL10I	1	Interleukins
IL21	interleukin	CVID11|IL	4	Interleukins

IL22	interleukin	IL-21|IL-22	12	Interleukins
IL23A	interleukin	IL-23|IL-23	12	Interleukins
IL24	interleukin	C49A|FISP	1	Interleukins
IL25	interleukin	IL17E	14	Interleukins
IL26	interleukin	AK155|IL-2	12	Interleukins
IL27	interleukin	IL-27|IL-27	16	Interleukins
IFNL2	interferon la	IL-28A|IL2	19	Interleukins
IFNL3	interferon la	IFN-lambda	19	Interleukins
IFNL1	interferon la	IL-29|IL29	19	Interleukins
IL3	interleukin	IL-3|MCGF	5	Interleukins
IL31	interleukin	IL-31	12	Interleukins
IL32	interleukin	IL-32alpha|	16	Interleukins
IL33	interleukin	C9orf26|DV	9	Interleukins
IL34	interleukin	C16orf77|IL	16	Interleukins
IL4	interleukin	BCGF-1|BC	5	Interleukins
IL5	interleukin	EDF|IL-5|T	5	Interleukins
IL6	interleukin	BSF-2|BSF	7	Interleukins
IL6ST	interleukin	CD130|CD	5	Interleukins
IL7	interleukin	IL-7	8	Interleukins
CXCL8	C-X-C mot	GCP-1|GCP	4	Interleukins
IL9	interleukin	HP40|IL-9|I	5	Interleukins
TXLNA	taxilin alph	IL14|TXLN	1	Interleukins
IL10RA	interleukin	CD210|CD2	11	Interleukins_Receptor
IL10RB	interleukin	CDW210B|	21	Interleukins_Receptor
IL11RA	interleukin	CRSDA	9	Interleukins_Receptor
IL12RB1	interleukin	CD212|IL-1	19	Interleukins_Receptor
IL12RB2	interleukin	-	1	Interleukins_Receptor
IL13RA1	interleukin	CD213A1|C	X	Interleukins_Receptor
IL13RA2	interleukin	CD213A2|C	X	Interleukins_Receptor
IL15RA	interleukin	CD215	10	Interleukins_Receptor
IL2RB	interleukin	CD122|IL1	22	Interleukins_Receptor
IL17RA	interleukin	CANDF5|C	22	Interleukins_Receptor
IL17RB	interleukin	CRL4|EVI2	3	Interleukins_Receptor
IL17RC	interleukin	CANDF9|IL	3	Interleukins_Receptor
IL17RD	interleukin	HH18|IL-1	3	Interleukins_Receptor
IL17RE	interleukin	-	3	Interleukins_Receptor
IL18R1	interleukin	CD218a|CD	2	Interleukins_Receptor
IL18RAP	interleukin	ACPL|CD2	2	Interleukins_Receptor
IL1R1	interleukin	CD121A|D	2	Interleukins_Receptor
IL1R2	interleukin	CD121b|CD	2	Interleukins_Receptor
IL1RAP	interleukin	C3orf13|IL-	3	Interleukins_Receptor
IL1RL1	interleukin	DER4|FIT-	2	Interleukins_Receptor
IL1RL2	interleukin	IL-1Rrp2|IL	2	Interleukins_Receptor
IL20RA	interleukin	CRF2-8|IL-	6	Interleukins_Receptor
IL20RB	interleukin	DIRS1|FNI	3	Interleukins_Receptor
IL21R	interleukin	CD360|IMI	16	Interleukins_Receptor
IL22RA1	interleukin	CRF2-9|IL2	1	Interleukins_Receptor
IL22RA2	interleukin	CRF2-10|C	6	Interleukins_Receptor
IL23R	interleukin	-	1	Interleukins_Receptor
IL27RA	interleukin	CRL1|IL-27	19	Interleukins_Receptor
IFNLR1	interferon la	CRF2/12|IF	1	Interleukins_Receptor
IL2RA	interleukin	CD25|IDDM	10	Interleukins_Receptor
IL2RB	interleukin	CD122|IL1	22	Interleukins_Receptor
IL2RG	interleukin	CD132|CID	X	Interleukins_Receptor
IL31RA	interleukin	CRL|CRL3	5	Interleukins_Receptor
IL3RA	interleukin	CD123|IL3	X|Y	Interleukins_Receptor
IL4R	interleukin	CD124|IL-4	16	Interleukins_Receptor
IL5RA	interleukin	CD125|CD	3	Interleukins_Receptor
IL6R	interleukin	CD126|IL-6	1	Interleukins_Receptor
IL7R	interleukin	CD127|CD	5	Interleukins_Receptor

CXCR1	C-X-C mot	C-C|C-C-C	2	Interleukins_Receptor
CXCR2	C-X-C mot	CD182|CD	2	Interleukins_Receptor
IL9R	interleukin	CD129|IL-9	X|Y	Interleukins_Receptor
ST2	-	-	11	Interleukins_Receptor
HLA-A	major histo	HLAA	6	NaturalKiller_Cell_Cytotoxicity
HLA-B	major histo	AS|B-4901|	6	NaturalKiller_Cell_Cytotoxicity
HLA-C	major histo	D6S204|HL	6	NaturalKiller_Cell_Cytotoxicity
HLA-E	major histo	HLA-6.2|Q	6	NaturalKiller_Cell_Cytotoxicity
HLA-G	major histo	MHC-G	6	NaturalKiller_Cell_Cytotoxicity
KIR3DL1	killer cell ir	CD158E1|K	19	NaturalKiller_Cell_Cytotoxicity
KIR3DL2	killer cell ir	3DL2|CD1	19	NaturalKiller_Cell_Cytotoxicity
KIR2DL1	killer cell ir	CD158A|K	19	NaturalKiller_Cell_Cytotoxicity
KIR2DL2	killer cell ir	CD158B1|C	19	NaturalKiller_Cell_Cytotoxicity
KIR2DL3	killer cell ir	CD158B2|C	19	NaturalKiller_Cell_Cytotoxicity
KIR2DL4	killer cell ir	CD158D|G	19	NaturalKiller_Cell_Cytotoxicity
KIR2DL5A	killer cell ir	CD158F|KI	19	NaturalKiller_Cell_Cytotoxicity
KLRC1	killer cell le	CD159A|N	12	NaturalKiller_Cell_Cytotoxicity
KLRC2	killer cell le	CD159c|NK	12	NaturalKiller_Cell_Cytotoxicity
KLRC3	killer cell le	NKG2-E|N	12	NaturalKiller_Cell_Cytotoxicity
KLRD1	killer cell le	CD94	12	NaturalKiller_Cell_Cytotoxicity
PTPN6	protein tyro	HCP|HCPH	12	NaturalKiller_Cell_Cytotoxicity
PTPN11	protein tyro	BPTP3|CFC	12	NaturalKiller_Cell_Cytotoxicity
ICAM1	intercellula	BB2|CD54|	19	NaturalKiller_Cell_Cytotoxicity
ICAM2	intercellula	CD102	17	NaturalKiller_Cell_Cytotoxicity
ITGAL	integrin sub	CD11A|LF	16	NaturalKiller_Cell_Cytotoxicity
ITGB2	integrin sub	CD18|LAD	21	NaturalKiller_Cell_Cytotoxicity
PTK2B	protein tyro	CADTK|CA	8	NaturalKiller_Cell_Cytotoxicity
VAV3	vav guanine	-	1	NaturalKiller_Cell_Cytotoxicity
VAV1	vav guanine	VAV	19	NaturalKiller_Cell_Cytotoxicity
VAV2	vav guanine	VAV-2	9	NaturalKiller_Cell_Cytotoxicity
RAC1	Rac family	MIG5|MRD	7	NaturalKiller_Cell_Cytotoxicity
RAC2	Rac family	EN-7|Gx|H	22	NaturalKiller_Cell_Cytotoxicity
RAC3	Rac family	-	17	NaturalKiller_Cell_Cytotoxicity
PAK1	p21 (RAC1	IDDMSSD|	11	NaturalKiller_Cell_Cytotoxicity
MAP2K1	mitogen-ac	CFC3|MAP	15	NaturalKiller_Cell_Cytotoxicity
MAP2K2	mitogen-ac	CFC4|MAP	19	NaturalKiller_Cell_Cytotoxicity
MAPK1	mitogen-ac	ERK|ERK-	22	NaturalKiller_Cell_Cytotoxicity
MAPK3	mitogen-ac	ERK-1|ERK	16	NaturalKiller_Cell_Cytotoxicity
TNF	tumor necro	DIF|TNF-a	6	NaturalKiller_Cell_Cytotoxicity
CSF2	colony stim	CSF|GMCS	5	NaturalKiller_Cell_Cytotoxicity
IFNG	interferon g	IFG|IFI	12	NaturalKiller_Cell_Cytotoxicity
KIR2DS1	killer cell ir	CD158H|C	19	NaturalKiller_Cell_Cytotoxicity
KIR2DS3	killer cell ir	NKAT7	19	NaturalKiller_Cell_Cytotoxicity
KIR2DS4	killer cell ir	CD158I|KI	19	NaturalKiller_Cell_Cytotoxicity
KIR2DS5	killer cell ir	CD158G|N	19	NaturalKiller_Cell_Cytotoxicity
NCR2	natural cyto	CD336|LY	6	NaturalKiller_Cell_Cytotoxicity
TYROBP	transmemb	DAP12|KA	19	NaturalKiller_Cell_Cytotoxicity
LCK	LCK proto-	IMD22|LSK	1	NaturalKiller_Cell_Cytotoxicity
FCGR3A	Fc fragmen	CD16|CD1	1	NaturalKiller_Cell_Cytotoxicity
FCGR3B	Fc fragmen	CD16|CD1	1	NaturalKiller_Cell_Cytotoxicity
NCR1	natural cyto	CD335|LY	19	NaturalKiller_Cell_Cytotoxicity
NCR3	natural cyto	1C7|CD337	6	NaturalKiller_Cell_Cytotoxicity
FCER1G	Fc fragmen	FCRG	1	NaturalKiller_Cell_Cytotoxicity
CD247	CD247 mol	CD3-ZETA	1	NaturalKiller_Cell_Cytotoxicity
ZAP70	zeta chain c	ADMIO2|II	2	NaturalKiller_Cell_Cytotoxicity
SYK	spleen asso	p72-Syk	9	NaturalKiller_Cell_Cytotoxicity
LCP2	lymphocyte	SLP-76|SL	5	NaturalKiller_Cell_Cytotoxicity
LAT	linker for a	IMD52|LA	16	NaturalKiller_Cell_Cytotoxicity
PLCG1	phospholipa	NCKAP3|P	20	NaturalKiller_Cell_Cytotoxicity
PLCG2	phospholipa	APLAID|F	16	NaturalKiller_Cell_Cytotoxicity

SH3BP2	SH3 domai	3BP-2|3BP	4	NaturalKiller_Cell_Cytotoxicity
PIK3CA	phosphatid	CLAPO|CL	3	NaturalKiller_Cell_Cytotoxicity
PIK3CB	phosphatid	P110BETA	3	NaturalKiller_Cell_Cytotoxicity
PIK3CD	phosphatid	APDS|IMD	1	NaturalKiller_Cell_Cytotoxicity
PIK3CG	phosphatid	PI3CG|PI3	7	NaturalKiller_Cell_Cytotoxicity
PIK3R5	phosphoino	F730038I1	17	NaturalKiller_Cell_Cytotoxicity
PIK3R1	phosphoino	AGM7|GR	5	NaturalKiller_Cell_Cytotoxicity
PIK3R2	phosphoino	MPPH|MP	19	NaturalKiller_Cell_Cytotoxicity
PIK3R3	phosphoino	p55|p55-G	1	NaturalKiller_Cell_Cytotoxicity
FYN	FYN proto-	SLK|SYN|	6	NaturalKiller_Cell_Cytotoxicity
SHC2	SHC adapt	SCK|SHCB	19	NaturalKiller_Cell_Cytotoxicity
SHC4	SHC adapt	RaLP|SHC	15	NaturalKiller_Cell_Cytotoxicity
SHC3	SHC adapt	N-Shc|NSH	9	NaturalKiller_Cell_Cytotoxicity
SHC1	SHC adapt	SHC|SHCA	1	NaturalKiller_Cell_Cytotoxicity
GRB2	growth fact	ASH|EGFR	17	NaturalKiller_Cell_Cytotoxicity
SOS1	SOS Ras/R	GF1|GGF1|	2	NaturalKiller_Cell_Cytotoxicity
SOS2	SOS Ras/R	NS9|SOS-2	14	NaturalKiller_Cell_Cytotoxicity
HRAS	HRas proto	C-BAS/HA	11	NaturalKiller_Cell_Cytotoxicity
KRAS	KRAS prot	'C-K-RAS|	12	NaturalKiller_Cell_Cytotoxicity
NRAS	NRAS prot	ALPS4|CM	1	NaturalKiller_Cell_Cytotoxicity
ARAF	A-Raf prot	A-RAF|AR	X	NaturalKiller_Cell_Cytotoxicity
BRAF	B-Raf prot	B-RAF1|B-	7	NaturalKiller_Cell_Cytotoxicity
RAF1	Raf-1 proto	CMD1NN|	3	NaturalKiller_Cell_Cytotoxicity
MICA	MHC class	MIC-A|PE	6	NaturalKiller_Cell_Cytotoxicity
MICB	MHC class	PERB11.2	6	NaturalKiller_Cell_Cytotoxicity
ULBP3	UL16 bindi	N2DL-3|N	6	NaturalKiller_Cell_Cytotoxicity
ULBP2	UL16 bindi	ALCAN-al	6	NaturalKiller_Cell_Cytotoxicity
ULBP1	UL16 bindi	N2DL-1|N	6	NaturalKiller_Cell_Cytotoxicity
KLRK1	killer cell l	CD314|D1	12	NaturalKiller_Cell_Cytotoxicity
HCST	hematopoie	DAP10|KA	19	NaturalKiller_Cell_Cytotoxicity
CD48	CD48 mole	BCM1|BL	1	NaturalKiller_Cell_Cytotoxicity
CD244	CD244 mol	2B4|NAIL|	1	NaturalKiller_Cell_Cytotoxicity
PPP3CA	protein pho	ACCIID|C	4	NaturalKiller_Cell_Cytotoxicity
PPP3CB	protein pho	CALNA2|C	10	NaturalKiller_Cell_Cytotoxicity
PPP3CC	protein pho	CALNA3|C	8	NaturalKiller_Cell_Cytotoxicity
CHP1	calcineurin	CHP|SLC9	15	NaturalKiller_Cell_Cytotoxicity
PPP3R1	protein pho	CALNB1|C	2	NaturalKiller_Cell_Cytotoxicity
PPP3R2	protein pho	PPP3RL	9	NaturalKiller_Cell_Cytotoxicity
CHP2	calcineurin	-	16	NaturalKiller_Cell_Cytotoxicity
NFAT5	nuclear fact	NF-AT5|N	16	NaturalKiller_Cell_Cytotoxicity
NFATC1	nuclear fact	NF-ATC|N	18	NaturalKiller_Cell_Cytotoxicity
NFATC2	nuclear fact	NFAT1|NF	20	NaturalKiller_Cell_Cytotoxicity
NFATC3	nuclear fact	NF-AT4c|N	16	NaturalKiller_Cell_Cytotoxicity
NFATC4	nuclear fact	NF-AT3|N	14	NaturalKiller_Cell_Cytotoxicity
PRKCA	protein kin	AAG6|PKC	17	NaturalKiller_Cell_Cytotoxicity
PRKCB	protein kin	PKC-beta|P	16	NaturalKiller_Cell_Cytotoxicity
PRKCG	protein kin	PKC-gamm	19	NaturalKiller_Cell_Cytotoxicity
SH2D1B	SH2 domai	EAT2	1	NaturalKiller_Cell_Cytotoxicity
SH2D1A	SH2 domai	DSHP|EBV	X	NaturalKiller_Cell_Cytotoxicity
IFNGR1	interferon g	CD119|IFN	6	NaturalKiller_Cell_Cytotoxicity
IFNGR2	interferon g	AF-1|IFGR	21	NaturalKiller_Cell_Cytotoxicity
IFNA1	interferon a	IFL|IFN|IF	9	NaturalKiller_Cell_Cytotoxicity
IFNA2	interferon a	IFN-alpha-	9	NaturalKiller_Cell_Cytotoxicity
IFNA4	interferon a	IFN-alpha4	9	NaturalKiller_Cell_Cytotoxicity
IFNA5	interferon a	IFN-alpha-	9	NaturalKiller_Cell_Cytotoxicity
IFNA6	interferon a	IFN-alphaK	9	NaturalKiller_Cell_Cytotoxicity
IFNA7	interferon a	IFN-alphaJ|	9	NaturalKiller_Cell_Cytotoxicity
IFNA8	interferon a	IFN-alphaB	9	NaturalKiller_Cell_Cytotoxicity
IFNA10	interferon a	IFN-alphaC	9	NaturalKiller_Cell_Cytotoxicity
IFNA13	interferon a	-	9	NaturalKiller_Cell_Cytotoxicity

IFNA14	interferon a	IFN-alphaH	9	NaturalKiller_Cell_Cytotoxicity
IFNA16	interferon a	IFN-alpha-	9	NaturalKiller_Cell_Cytotoxicity
IFNA17	interferon a	IFN-alphaI|	9	NaturalKiller_Cell_Cytotoxicity
IFNA21	interferon a	IFN-alphaI|	9	NaturalKiller_Cell_Cytotoxicity
IFNB1	interferon b	IFB|IFF|IFN	9	NaturalKiller_Cell_Cytotoxicity
IFNAR1	interferon a	AVP|IFN-a	21	NaturalKiller_Cell_Cytotoxicity
IFNAR2	interferon a	IFN-R|IFN-	21	NaturalKiller_Cell_Cytotoxicity
TNFSF10	TNF superf	APO2L|Ap	3	NaturalKiller_Cell_Cytotoxicity
TNFRSF10	TNF recept	CD264|DC	8	NaturalKiller_Cell_Cytotoxicity
TNFRSF10	TNF recept	CD263|DC	8	NaturalKiller_Cell_Cytotoxicity
TNFRSF10	TNF recept	CD262|DR	8	NaturalKiller_Cell_Cytotoxicity
TNFRSF10	TNF recept	APO2|CD2	8	NaturalKiller_Cell_Cytotoxicity
FASLG	Fas ligand	ALPS1B|A	1	NaturalKiller_Cell_Cytotoxicity
FAS	Fas cell sur	ALPS1A|A	10	NaturalKiller_Cell_Cytotoxicity
GZMB	granzyme B	C11|CCPI|C	14	NaturalKiller_Cell_Cytotoxicity
PRF1	perforin 1	HPLH2|P1|	10	NaturalKiller_Cell_Cytotoxicity
CASP3	caspase 3	CPP32|CPP	4	NaturalKiller_Cell_Cytotoxicity
BID	BH3 intera	FP497	22	NaturalKiller_Cell_Cytotoxicity
CD3D	CD3d mole	CD3-DELT	11	TCRsignalingPathway
CD3E	CD3e mole	IMD18|T3E	11	TCRsignalingPathway
CD3G	CD3g mole	CD3-GAM	11	TCRsignalingPathway
CD247	CD247 mol	CD3-ZETA	1	TCRsignalingPathway
CD4	CD4 molec	CD4mut	12	TCRsignalingPathway
CD8A	CD8a mole	CD8|Leu2|p	2	TCRsignalingPathway
CD8B	CD8b mole	CD8B1|LE	2	TCRsignalingPathway
PTPRC	protein tyro	B220|CD45	1	TCRsignalingPathway
LCK	LCK proto-	IMD22|LSK	1	TCRsignalingPathway
FYN	FYN proto-	SLK|SYN|p	6	TCRsignalingPathway
ZAP70	zeta chain c	ADMIO2|II	2	TCRsignalingPathway
LCP2	lymphocyte	SLP-76|SL	5	TCRsignalingPathway
LAT	linker for a	IMD52|LA	16	TCRsignalingPathway
ITK	IL2 inducib	EMT|LPFS	5	TCRsignalingPathway
TEC	tec protein	PSCTK4	4	TCRsignalingPathway
NCK1	NCK adapt	NCK|NCKa	3	TCRsignalingPathway
NCK2	NCK adapt	GRB4|NCK	2	TCRsignalingPathway
VAV3	vav guanine	-	1	TCRsignalingPathway
VAV1	vav guanine	VAV	19	TCRsignalingPathway
VAV2	vav guanine	VAV-2	9	TCRsignalingPathway
GRAP2	GRB2 relat	GADS|GRA	22	TCRsignalingPathway
GRB2	growth fact	ASH|EGFR	17	TCRsignalingPathway
PAK1	p21 (RAC1	IDDMSSD|	11	TCRsignalingPathway
PAK2	p21 (RAC1	PAK65|PA	3	TCRsignalingPathway
PAK3	p21 (RAC1	ARA|MRX	X	TCRsignalingPathway
PAK4	p21 (RAC1	-	19	TCRsignalingPathway
PAK6	p21 (RAC1	PAK5	15	TCRsignalingPathway
PAK5	p21 (RAC1	PAK7	20	TCRsignalingPathway
RHOA	ras homolog	ARH12|AR	3	TCRsignalingPathway
CDC42	cell division	CDC42Hs|C	1	TCRsignalingPathway
PPP3CA	protein pho	ACCIID|CA	4	TCRsignalingPathway
PPP3CB	protein pho	CALNA2|C	10	TCRsignalingPathway
PPP3CC	protein pho	CALNA3|C	8	TCRsignalingPathway
CHP1	calcineurin	CHP|SLC9	15	TCRsignalingPathway
PPP3R1	protein pho	CALNB1|C	2	TCRsignalingPathway
PPP3R2	protein pho	PPP3RL	9	TCRsignalingPathway
CHP2	calcineurin	-	16	TCRsignalingPathway
NFAT5	nuclear fact	NF-AT5|NI	16	TCRsignalingPathway
NFATC1	nuclear fact	NF-ATC|N	18	TCRsignalingPathway
NFATC2	nuclear fact	NFAT1|NF	20	TCRsignalingPathway
NFATC3	nuclear fact	NF-AT4c|N	16	TCRsignalingPathway
NFATC4	nuclear fact	NF-AT3|NI	14	TCRsignalingPathway

SOS1	SOS Ras/R	GF1|GGF1|	2	TCRsignalingPathway
SOS2	SOS Ras/R	NS9|SOS-2	14	TCRsignalingPathway
HRAS	HRas proto	C-BAS/HA	11	TCRsignalingPathway
KRAS	KRAS prot	'C-K-RAS|	12	TCRsignalingPathway
NRAS	NRAS prot	ALPS4|CM	1	TCRsignalingPathway
FOS	Fos proto-o	AP-1|C-FO	14	TCRsignalingPathway
JUN	Jun proto-o	AP-1|AP1|	1	TCRsignalingPathway
CARD11	caspase rec	BENTA|BI	7	TCRsignalingPathway
BCL10	BCL10 imr	CARMEN|	1	TCRsignalingPathway
MALT1	MALT1 pa	IMD12|ML	18	TCRsignalingPathway
CHUK	component	IKBKA|IK	10	TCRsignalingPathway
IKBKB	inhibitor of	IKK-beta|I	8	TCRsignalingPathway
IKBKG	inhibitor of	AMCBX1|I	X	TCRsignalingPathway
NFKB1	nuclear fact	CVID12|E	4	TCRsignalingPathway
RELA	RELA prot	CMCU|NF	11	TCRsignalingPathway
NFKBIA	NFKB inhi	EDAID2|I	14	TCRsignalingPathway
NFKBIB	NFKB inhi	IKBB|TRI	19	TCRsignalingPathway
NFKBIE	NFKB inhi	IKBE	6	TCRsignalingPathway
CD28	CD28 mole	Tp44	2	TCRsignalingPathway
ICOS	inducible T	AILIM|CD	2	TCRsignalingPathway
CD40LG	CD40 ligan	CD154|CD	X	TCRsignalingPathway
PIK3R5	phosphoino	F730038I1	17	TCRsignalingPathway
PIK3R1	phosphoino	AGM7|GR	5	TCRsignalingPathway
PIK3R2	phosphoino	MPPH|MP	19	TCRsignalingPathway
PIK3R3	phosphoino	p55|p55-G	1	TCRsignalingPathway
PIK3CA	phosphatid	CLAPO|CL	3	TCRsignalingPathway
PIK3CB	phosphatid	P110BETA	3	TCRsignalingPathway
PIK3CD	phosphatid	APDS|IMD	1	TCRsignalingPathway
PIK3CG	phosphatid	PI3CG|PI3	7	TCRsignalingPathway
AKT3	AKT serine	MPPH|MP	1	TCRsignalingPathway
AKT1	AKT serine	AKT|CWS	14	TCRsignalingPathway
AKT2	AKT serine	HIHGHH|P	19	TCRsignalingPathway
MAP3K8	mitogen-ac	AURA2|C	10	TCRsignalingPathway
MAP3K14	mitogen-ac	FTDCR1B|	17	TCRsignalingPathway
PDCD1	programme	CD279|PD-	2	TCRsignalingPathway
CTLA4	cytotoxic T	ALPS5|CD	2	TCRsignalingPathway
PTPN6	protein tyro	HCP|HCP	12	TCRsignalingPathway
CBLC	Cbl proto-o	CBL-3|CB	19	TCRsignalingPathway
CBL	Cbl proto-o	C-CBL|CB	11	TCRsignalingPathway
CBLB	Cbl proto-o	Cbl-b|Nbla	3	TCRsignalingPathway
IL2	interleukin	IL-2|TCGF	4	TCRsignalingPathway
IL4	interleukin	BCGF-1|B	5	TCRsignalingPathway
IL5	interleukin	EDF|IL-5|T	5	TCRsignalingPathway
IL10	interleukin	CSIF|GVH	1	TCRsignalingPathway
IFNG	interferon g	IFG|IFI	12	TCRsignalingPathway
CSF2	colony stim	CSF|GMC	5	TCRsignalingPathway
TNF	tumor necr	DIF|TNF-a	6	TCRsignalingPathway
CDK4	cyclin depe	CMM3|PS	12	TCRsignalingPathway
RASGRP1	RAS guany	CALDAG-	15	TCRsignalingPathway
PDK1	pyruvate de	-	2	TCRsignalingPathway
PLCG1	phospholip	NCKAP3|P	20	TCRsignalingPathway
PRKCQ	protein kin	PRKCT|nP	10	TCRsignalingPathway
TRAC	T cell recep	IMD7|TCR	14	TCRsignalingPathway
TRAJ1	T cell recep	-	14	TCRsignalingPathway
TRAJ2	T cell recep	-	14	TCRsignalingPathway
TRAJ3	T cell recep	-	14	TCRsignalingPathway
TRAJ4	T cell recep	-	14	TCRsignalingPathway
TRAJ5	T cell recep	-	14	TCRsignalingPathway
TRAJ6	T cell recep	-	14	TCRsignalingPathway
TRAJ7	T cell recep	-	14	TCRsignalingPathway

TRAJ8	T cell recep	-	14	TCRsignalingPathway
TRAJ9	T cell recep	-	14	TCRsignalingPathway
TRAJ10	T cell recep	-	14	TCRsignalingPathway
TRAJ11	T cell recep	-	14	TCRsignalingPathway
TRAJ12	T cell recep	-	14	TCRsignalingPathway
TRAJ13	T cell recep	-	14	TCRsignalingPathway
TRAJ14	T cell recep	-	14	TCRsignalingPathway
TRAJ15	T cell recep	-	14	TCRsignalingPathway
TRAJ16	T cell recep	-	14	TCRsignalingPathway
TRAJ17	T cell recep	-	14	TCRsignalingPathway
TRAJ18	T cell recep	-	14	TCRsignalingPathway
TRAJ19	T cell recep	-	14	TCRsignalingPathway
TRAJ20	T cell recep	-	14	TCRsignalingPathway
TRAJ21	T cell recep	-	14	TCRsignalingPathway
TRAJ22	T cell recep	-	14	TCRsignalingPathway
TRAJ23	T cell recep	-	14	TCRsignalingPathway
TRAJ24	T cell recep	-	14	TCRsignalingPathway
TRAJ25	T cell recep	-	14	TCRsignalingPathway
TRAJ26	T cell recep	-	14	TCRsignalingPathway
TRAJ27	T cell recep	-	14	TCRsignalingPathway
TRAJ28	T cell recep	-	14	TCRsignalingPathway
TRAJ29	T cell recep	-	14	TCRsignalingPathway
TRAJ30	T cell recep	-	14	TCRsignalingPathway
TRAJ31	T cell recep	-	14	TCRsignalingPathway
TRAJ32	T cell recep	-	14	TCRsignalingPathway
TRAJ33	T cell recep	-	14	TCRsignalingPathway
TRAJ34	T cell recep	-	14	TCRsignalingPathway
TRAJ35	T cell recep	-	14	TCRsignalingPathway
TRAJ36	T cell recep	-	14	TCRsignalingPathway
TRAJ37	T cell recep	-	14	TCRsignalingPathway
TRAJ38	T cell recep	-	14	TCRsignalingPathway
TRAJ39	T cell recep	-	14	TCRsignalingPathway
TRAJ40	T cell recep	-	14	TCRsignalingPathway
TRAJ41	T cell recep	-	14	TCRsignalingPathway
TRAJ42	T cell recep	-	14	TCRsignalingPathway
TRAJ43	T cell recep	-	14	TCRsignalingPathway
TRAJ44	T cell recep	-	14	TCRsignalingPathway
TRAJ45	T cell recep	-	14	TCRsignalingPathway
TRAJ46	T cell recep	-	14	TCRsignalingPathway
TRAJ47	T cell recep	-	14	TCRsignalingPathway
TRAJ48	T cell recep	-	14	TCRsignalingPathway
TRAJ49	T cell recep	-	14	TCRsignalingPathway
TRAJ50	T cell recep	-	14	TCRsignalingPathway
TRAJ52	T cell recep	-	14	TCRsignalingPathway
TRAJ53	T cell recep	-	14	TCRsignalingPathway
TRAJ54	T cell recep	-	14	TCRsignalingPathway
TRAJ56	T cell recep	-	14	TCRsignalingPathway
TRAJ57	T cell recep	-	14	TCRsignalingPathway
TRAJ58	T cell recep	-	14	TCRsignalingPathway
TRAJ59	T cell recep	-	14	TCRsignalingPathway
TRAJ61	T cell recep	-	14	TCRsignalingPathway
TRAV1-1	T cell recep	TCRAV1S	14	TCRsignalingPathway
TRAV1-2	T cell recep	TCRAV1S	14	TCRsignalingPathway
TRAV2	T cell recep	TCRAV11S	14	TCRsignalingPathway
TRAV3	T cell recep	TCRAV16S	14	TCRsignalingPathway
TRAV4	T cell recep	TCRAV20S	14	TCRsignalingPathway
TRAV5	T cell recep	TCRAV15S	14	TCRsignalingPathway
TRAV7	T cell recep	TCRAV7S	14	TCRsignalingPathway
TRAV8-1	T cell recep	TCRAV1S	14	TCRsignalingPathway
TRAV8-2	T cell recep	TCRAV1S	14	TCRsignalingPathway

TRAV8-3	T cell recep	TCRAV1S4	14	TCRsignalingPathway
TRAV8-4	T cell recep	TCRAV1S	14	TCRsignalingPathway
TRAV8-6	T cell recep	TCRAV1S	14	TCRsignalingPathway
TRAV8-7	T cell recep	TCRAV8S	14	TCRsignalingPathway
TRAV9-1	T cell recep	TCRAV9S	14	TCRsignalingPathway
TRAV9-2	T cell recep	TCRAV22S	14	TCRsignalingPathway
TRAV10	T cell recep	TCRAV10S	14	TCRsignalingPathway
TRAV12-1	T cell recep	TCRAV12S	14	TCRsignalingPathway
TRAV12-2	T cell recep	TCRAV12S	14	TCRsignalingPathway
TRAV12-3	T cell recep	TCRAV12S	14	TCRsignalingPathway
TRAV13-1	T cell recep	TCRAV13S	14	TCRsignalingPathway
TRAV13-2	T cell recep	TCRAV13S	14	TCRsignalingPathway
TRAV14D	T cell recep	TCRAV6S	14	TCRsignalingPathway
TRAV16	T cell recep	TCRAV16S	14	TCRsignalingPathway
TRAV17	T cell recep	TCRAV17S	14	TCRsignalingPathway
TRAV18	T cell recep	TCRAV18S	14	TCRsignalingPathway
TRAV19	T cell recep	TCRAV12S	14	TCRsignalingPathway
TRAV20	T cell recep	TCRAV20S	14	TCRsignalingPathway
TRAV21	T cell recep	TCRAV21S	14	TCRsignalingPathway
TRAV22	T cell recep	TCRAV13S	14	TCRsignalingPathway
TRAV23D	T cell recep	TCRAV17S	14	TCRsignalingPathway
TRAV24	T cell recep	TCRAV18S	14	TCRsignalingPathway
TRAV25	T cell recep	TCRAV25S	14	TCRsignalingPathway
TRAV26-1	T cell recep	TCRAV26S	14	TCRsignalingPathway
TRAV26-2	T cell recep	TCRAV26S	14	TCRsignalingPathway
TRAV27	T cell recep	TCRAV10S	14	TCRsignalingPathway
TRAV29D	T cell recep	TCRA|TCR	14	TCRsignalingPathway
TRAV30	T cell recep	TCRAV29S	14	TCRsignalingPathway
TRAV34	T cell recep	TCRAV26S	14	TCRsignalingPathway
TRAV35	T cell recep	TCRAV25S	14	TCRsignalingPathway
TRAV36D	T cell recep	TCRAV28S	14	TCRsignalingPathway
TRAV38-1	T cell recep	TCRAV14S	14	TCRsignalingPathway
TRAV38-2	T cell recep	TCRAV14S	14	TCRsignalingPathway
TRAV39	T cell recep	TCRAV27S	14	TCRsignalingPathway
TRAV40	T cell recep	TCRAV31S	14	TCRsignalingPathway
TRAV41	T cell recep	TCRAV19S	14	TCRsignalingPathway
TRBC1	T cell recep	BV05S1J2.	7	TCRsignalingPathway
TRBC2	T cell recep	TCRBC2	7	TCRsignalingPathway
TRBD1	T cell recep	TCRBD1	7	TCRsignalingPathway
TRBD2	T cell recep	TCRBD2	7	TCRsignalingPathway
TRBJ1-1	T cell recep	TCRBJ1S1	7	TCRsignalingPathway
TRBJ1-2	T cell recep	TCRBJ1S2	7	TCRsignalingPathway
TRBJ1-3	T cell recep	TCRBJ1S3	7	TCRsignalingPathway
TRBJ1-4	T cell recep	TCRBJ1S4	7	TCRsignalingPathway
TRBJ1-5	T cell recep	TCRBJ1S5	7	TCRsignalingPathway
TRBJ1-6	T cell recep	TCRBJ1S6	7	TCRsignalingPathway
TRBJ2-1	T cell recep	TCRBJ2S1	7	TCRsignalingPathway
TRBJ2-2	T cell recep	TCRBJ2S2	7	TCRsignalingPathway
TRBJ2-3	T cell recep	TCRBJ2S3	7	TCRsignalingPathway
TRBJ2-4	T cell recep	TCRBJ2S4	7	TCRsignalingPathway
TRBJ2-5	T cell recep	TCRBJ2S5	7	TCRsignalingPathway
TRBJ2-6	T cell recep	TCRBJ2S6	7	TCRsignalingPathway
TRBJ2-7	T cell recep	TCRBJ2S7	7	TCRsignalingPathway
TRBV2	T cell recep	TCRBV22S	7	TCRsignalingPathway
TRBV3-1	T cell recep	TCRBV3S	7	TCRsignalingPathway
TRBV4-1	T cell recep	BV07S1J2.	7	TCRsignalingPathway
TRBV4-2	T cell recep	TCRBV4S	7	TCRsignalingPathway
TRBV4-3	T cell recep	TCRBV4S	7	TCRsignalingPathway
TRBV5-1	T cell recep	TCRBV5S	7	TCRsignalingPathway
TRBV5-4	T cell recep	TCRBV5S4	7	TCRsignalingPathway

TRBV5-5	T cell recep	TCRBV5S3	7	TCRsignalingPathway
TRBV5-6	T cell recep	TCRBV5S2	7	TCRsignalingPathway
TRBV5-7	T cell recep	TCRBV5S1	7	TCRsignalingPathway
TRBV5-8	T cell recep	TCRBV5S4	7	TCRsignalingPathway
TRBV6-1	T cell recep	TCRBV13S	7	TCRsignalingPathway
TRBV6-2	T cell recep	TCRBV13S	7	TCRsignalingPathway
TRBV6-3	T cell recep	TCRBV13S	7	TCRsignalingPathway
TRBV6-4	T cell recep	TCRBV13S	7	TCRsignalingPathway
TRBV6-5	T cell recep	TCRBV13S	7	TCRsignalingPathway
TRBV6-6	T cell recep	TCRBV13S	7	TCRsignalingPathway
TRBV6-7	T cell recep	TCRBV13S	7	TCRsignalingPathway
TRBV6-8	T cell recep	TCRBV13S	7	TCRsignalingPathway
TRBV6-9	T cell recep	TCRBV13S	7	TCRsignalingPathway
TRBV7-2	T cell recep	TCRBV6S5	7	TCRsignalingPathway
TRBV7-3	T cell recep	TCRBV6S1	7	TCRsignalingPathway
TRBV7-4	T cell recep	TCRBV6S8	7	TCRsignalingPathway
TRBV7-6	T cell recep	TCRBV6S3	7	TCRsignalingPathway
TRBV7-7	T cell recep	TCRBV6S6	7	TCRsignalingPathway
TRBV7-8	T cell recep	TCRBV6S2	7	TCRsignalingPathway
TRBV7-9	T cell recep	TCRB|TCR	7	TCRsignalingPathway
TRBV9	T cell recep	TCRBV1S1	7	TCRsignalingPathway
TRBV10-1	T cell recep	TCRBV10S	7	TCRsignalingPathway
TRBV10-2	T cell recep	TCRBV10S	7	TCRsignalingPathway
TRBV10-3	T cell recep	TCRBV10S	7	TCRsignalingPathway
TRBV11-1	T cell recep	TCRBV11S	7	TCRsignalingPathway
TRBV11-2	T cell recep	TCRBV11S	7	TCRsignalingPathway
TRBV11-3	T cell recep	TCRBV11S	7	TCRsignalingPathway
TRBV12-3	T cell recep	TCRBV12S	7	TCRsignalingPathway
TRBV12-4	T cell recep	TCRBV12S	7	TCRsignalingPathway
TRBV12-5	T cell recep	TCRBV12S	7	TCRsignalingPathway
TRBV13	T cell recep	TCRBV13S	7	TCRsignalingPathway
TRBV14	T cell recep	TCRBV14S	7	TCRsignalingPathway
TRBV15	T cell recep	TCRBV15S	7	TCRsignalingPathway
TRBV16	T cell recep	BV25S1J1.	7	TCRsignalingPathway
TRBV17	T cell recep	TCRBV17S	7	TCRsignalingPathway
TRBV18	T cell recep	TCRBV18S	7	TCRsignalingPathway
TRBV19	T cell recep	TCRBV17S	7	TCRsignalingPathway
TRBV20-1	T cell recep	TCRBV20S	7	TCRsignalingPathway
TRBV24-1	T cell recep	TCRBV15S	7	TCRsignalingPathway
TRBV25-1	T cell recep	TCRBV11S	7	TCRsignalingPathway
TRBV27	T cell recep	TCRBV14S	7	TCRsignalingPathway
TRBV28	T cell recep	TCRBV28S	7	TCRsignalingPathway
TRBV29-1	T cell recep	TCRBV29S	7	TCRsignalingPathway
TRBV30	T cell recep	TCRBV20S	7	TCRsignalingPathway
TRDC	T cell recep	TCRD	14	TCRsignalingPathway
TRDD1	T cell recep	-	14	TCRsignalingPathway
TRDD2	T cell recep	-	14	TCRsignalingPathway
TRDD3	T cell recep	TCRD	14	TCRsignalingPathway
TRDJ1	T cell recep	TCRD	14	TCRsignalingPathway
TRDJ2	T cell recep	-	14	TCRsignalingPathway
TRDJ3	T cell recep	-	14	TCRsignalingPathway
TRDJ4	T cell recep	-	14	TCRsignalingPathway
TRDV1	T cell recep	hDV101S1	14	TCRsignalingPathway
TRDV2	T cell recep	hDV102S1	14	TCRsignalingPathway
TRDV3	T cell recep	hDV103S1	14	TCRsignalingPathway
TRGV9	T cell recep	TCRGV9|T	7	TCRsignalingPathway
TRGV8	T cell recep	TCRGV8|V	7	TCRsignalingPathway
TRGV5	T cell recep	TCRGV5|V	7	TCRsignalingPathway
TRGV4	T cell recep	TCRGV4|V	7	TCRsignalingPathway
TRGV3	T cell recep	TCRGV3|V	7	TCRsignalingPathway

TRGV2	T cell recep	TCRGV2|V	7	TCRsignalingPathway
TRGJP2	T cell recep	JP2|TCRGJ	7	TCRsignalingPathway
TRGJP1	T cell recep	JP1|TCRGJ	7	TCRsignalingPathway
TRGJP	T cell recep	JP|TCRGJF	7	TCRsignalingPathway
TRGJ2	T cell recep	J2|TCRGJ2	7	TCRsignalingPathway
TRGJ1	T cell recep	J1|TCRGJ1	7	TCRsignalingPathway
TRGC2	T cell recep	TCRGC2|T	7	TCRsignalingPathway
TRGC1	T cell recep	C1|TCRG|T	7	TCRsignalingPathway
TRAV6	T cell recep	TCRAV5S	14	TCRsignalingPathway
BMP1	bone morph	OI13|PCOL	8	TGFb_Family_Member
BMP10	bone morph	-	2	TGFb_Family_Member
BMP15	bone morph	GDF9B|OD	X	TGFb_Family_Member
BMP2	bone morph	BDA2|BMI	20	TGFb_Family_Member
BMP3	bone morph	BMP-3A	4	TGFb_Family_Member
BMP4	bone morph	BMP2B|BN	14	TGFb_Family_Member
BMP5	bone morph	-	6	TGFb_Family_Member
BMP6	bone morph	VGR|VGR	6	TGFb_Family_Member
BMP7	bone morph	OP-1	20	TGFb_Family_Member
BMP8A	bone morph	OP-2	1	TGFb_Family_Member
BMP8B	bone morph	BMP8|OP2	1	TGFb_Family_Member
GDF1	growth diff	CERS1|CH	19	TGFb_Family_Member
GDF10	growth diff	BIP|BMP-3	10	TGFb_Family_Member
GDF11	growth diff	BMP-11|BN	12	TGFb_Family_Member
GDF15	growth diff	GDF-15|MI	19	TGFb_Family_Member
GDF2	growth diff	BMP-9|BM	10	TGFb_Family_Member
GDF3	growth diff	KFS3|MCC	12	TGFb_Family_Member
GDF5	growth diff	BDA1C|BN	20	TGFb_Family_Member
GDF6	growth diff	BMP-13|BN	8	TGFb_Family_Member
GDF7	growth diff	BMP12	2	TGFb_Family_Member
GDF9	growth diff	POF14	5	TGFb_Family_Member
GDNF	glial cell de	ATF|ATF1|	5	TGFb_Family_Member
INHA	inhibin sub	-	2	TGFb_Family_Member
INHBA	inhibin sub	EDF|FRP	7	TGFb_Family_Member
INHBB	inhibin sub	-	2	TGFb_Family_Member
INHBC	inhibin sub	IHBC	12	TGFb_Family_Member
INHBE	inhibin sub	-	12	TGFb_Family_Member
LEFTY1	left-right de	LEFTB|LE	1	TGFb_Family_Member
LEFTY2	left-right de	EBAF|LEF	1	TGFb_Family_Member
NODAL	nodal grow	HTX5	10	TGFb_Family_Member
TGFB1	transformin	CED|DPD1	19	TGFb_Family_Member
TGFB2	transformin	G-TSF|LDS	1	TGFb_Family_Member
TGFB3	transformin	ARVD|ARY	14	TGFb_Family_Member
ACVR1B	activin A re	ACTRIB|A	12	TGFb_Family_Member_Receptor
ACVR1C	activin A re	ACVRLK7	2	TGFb_Family_Member_Receptor
ACVR2A	activin A re	ACTRII|AC	2	TGFb_Family_Member_Receptor
ACVR2B	activin A re	ACTRIIB|A	3	TGFb_Family_Member_Receptor
ACVRL1	activin A re	ACVRLK1	12	TGFb_Family_Member_Receptor
AMHR2	anti-Muller	AMHR|MIS	12	TGFb_Family_Member_Receptor
BMPR1A	bone morph	10q23del|A	10	TGFb_Family_Member_Receptor
BMPR1B	bone morph	ALK-6|ALI	4	TGFb_Family_Member_Receptor
BMPR2	bone morph	BMPR-II|B	2	TGFb_Family_Member_Receptor
TGFBR1	transformin	AAT5|ACV	9	TGFb_Family_Member_Receptor
TGFBR2	transformin	AAT3|FAA	3	TGFb_Family_Member_Receptor
TGFBR3	transformin	BGCAN|be	1	TGFb_Family_Member_Receptor
TNFRSF11	TNF recept	OCIF|OPG|	8	TNF_Family_Members
TNFSF10	TNF superf	APO2L|Ap	3	TNF_Family_Members
TNFSF11	TNF superf	CD254|OD	13	TNF_Family_Members
TNFSF12	TNF superf	APO3L|DR	17	TNF_Family_Members
TNFSF13	TNF superf	APRIL|CD	17	TNF_Family_Members
TNFSF13B	TNF superf	BAFF|BLY	13	TNF_Family_Members

TNFSF14	TNF superf	CD258|HV	19	TNF_Family_Members
TNFSF15	TNF superf	TL1|TL1A|	9	TNF_Family_Members
TNFSF18	TNF superf	AITRL|GIT	1	TNF_Family_Members
TNFSF4	TNF superf	CD134L|C	1	TNF_Family_Members
TNFSF8	TNF superf	CD153|CD	9	TNF_Family_Members
TNFSF9	TNF superf	4-1BB-L|C	19	TNF_Family_Members
TNFRSF10	TNF recept	CD262|DR	8	TNF_Family_Members_Receptors
TNFRSF10	TNF recept	CD263|DC	8	TNF_Family_Members_Receptors
TNFRSF10	TNF recept	CD264|DC	8	TNF_Family_Members_Receptors
TNFRSF11	TNF recept	CD265|FE	18	TNF_Family_Members_Receptors
TNFRSF12	TNF recept	CD266|FN	16	TNF_Family_Members_Receptors
TNFRSF13	TNF recept	CD267|CV	17	TNF_Family_Members_Receptors
TNFRSF13	TNF recept	BAFF-R|B	22	TNF_Family_Members_Receptors
TNFRSF14	TNF recept	ATAR|CD	1	TNF_Family_Members_Receptors
TNFRSF17	TNF recept	BCM|BCM	16	TNF_Family_Members_Receptors
TNFRSF18	TNF recept	AITR|CD3	1	TNF_Family_Members_Receptors
TNFRSF19	TNF recept	TAJ|TAJ-al	13	TNF_Family_Members_Receptors
TNFRSF1	TNF recept	CD120a|FP	12	TNF_Family_Members_Receptors
TNFRSF1	TNF recept	CD120b|T	1	TNF_Family_Members_Receptors
TNFRSF21	TNF recept	BM-018|C	6	TNF_Family_Members_Receptors
TNFRSF25	TNF recept	APO-3|DD	1	TNF_Family_Members_Receptors
TNFRSF4	TNF recept	ACT35|CD	1	TNF_Family_Members_Receptors
TNFRSF6	TNF recept	DCR3|DJ5	20	TNF_Family_Members_Receptors
TNFRSF8	TNF recept	CD30|D1S	1	TNF_Family_Members_Receptors
TNFRSF9	TNF recept	4-1BB|CD1	1	TNF_Family_Members_Receptors

Supplementary 3 Co-expressed mRNA

symbol	correlation	pvalue	ENTREZID
NOX4	1	0	50507
VCAN	0.884007	8.89E-142	1462
INHBA	0.883179	3.65E-141	3624
COL8A1	0.881659	4.75E-140	1295
COL10A1	0.880247	4.99E-139	1300
THBS2	0.879597	1.46E-138	7058
PRRX1	0.876591	1.92E-136	5396
KCND2	0.871782	3.64E-133	3751
CTHRC1	0.861228	2.05E-126	115908
ANTXR1	0.857947	1.99E-124	84168
SULF1	0.855768	3.89E-123	23213
ST6GALNAC5	0.850349	5.16E-120	81849
RAB31	0.845575	2.30E-117	11031
POSTN	0.84305	5.33E-116	10631
SPARC	0.841459	3.76E-115	6678
FAP	0.840486	1.23E-114	2191
ASPN	0.835146	7.07E-112	54829
LUM	0.834908	9.33E-112	4060
COL5A2	0.831806	3.36E-110	1290
SPOCK1	0.831141	7.17E-110	6695
NTM	0.826443	1.39E-107	50863
SFRP4	0.825098	6.09E-107	6424
COL11A1	0.822712	8.13E-106	1301
LOX	0.816923	3.74E-103	4015
WISP1	0.814078	7.02E-102	NA
PPAPDC1A	0.81338	1.43E-101	NA
BGN	0.812652	3.00E-101	633
COL8A2	0.801114	2.43E-96	1296
AEBP1	0.800257	5.47E-96	165
FBN1	0.799936	7.39E-96	2200
TENM4	0.799576	1.04E-95	26011
ECM2	0.794142	1.58E-93	1842
ITGA11	0.792822	5.23E-93	22801
COL1A2	0.791353	1.96E-92	1278
OLFML2B	0.79039	4.65E-92	25903
COL3A1	0.782437	4.82E-89	1281
CDH11	0.781853	7.94E-89	1009
ADAMTS12	0.779808	4.49E-88	81792
LRRC15	0.779308	6.84E-88	131578
OLR1	0.77717	4.09E-87	4973
PLXDC2	0.774929	2.61E-86	84898
FAM26E	0.773329	9.65E-86	NA
ISM1	0.769543	2.05E-84	140862
BICC1	0.768868	3.51E-84	80114
ADAM12	0.76848	4.79E-84	8038
P4HA3	0.765055	7.12E-83	283208
FGF1	0.763607	2.20E-82	2246
NALCN	0.763571	2.26E-82	259232
CDH2	0.762114	6.98E-82	1000
KCNE4	0.761923	8.09E-82	23704
COL1A1	0.761643	1.00E-81	1277
COL5A1	0.758711	9.39E-81	1289
CMTM3	0.758597	1.02E-80	123920
FNDC1	0.757832	1.83E-80	84624
COL12A1	0.755608	9.69E-80	1303
DOK5	0.75509	1.43E-79	55816
GPR176	0.754651	1.98E-79	11245
TGFB3	0.754148	2.87E-79	7043

FSTL1	0.753652	4.14E-79	11167
PDGFRB	0.75363	4.21E-79	5159
PDGFC	0.749954	6.22E-78	56034
COL6A3	0.749878	6.58E-78	1293
HTRA3	0.749552	8.33E-78	94031
CHSY3	0.748085	2.40E-77	337876
FAM19A5	0.745052	2.10E-76	NA
MXRA8	0.744459	3.20E-76	54587
MXRA5	0.744456	3.21E-76	25878
GPC6	0.742341	1.42E-75	10082
ITGBL1	0.740812	4.14E-75	9358
BCAT1	0.740239	6.16E-75	586
DIO2	0.739875	7.93E-75	1734
TNFAIP6	0.739812	8.28E-75	7130
HECW1	0.73932	1.16E-74	23072
NUAK1	0.738933	1.52E-74	9891
HTRA1	0.73866	1.84E-74	5654
PRICKLE1	0.738573	1.95E-74	144165
GFPT2	0.736133	1.04E-73	9945
FIBIN	0.734362	3.44E-73	387758
CERCAM	0.733901	4.70E-73	51148
VGLL3	0.73237	1.32E-72	389136
LTBP2	0.732342	1.34E-72	4053
DSE	0.731174	2.92E-72	29940
KCNJ15	0.730791	3.77E-72	3772
PXDN	0.729544	8.62E-72	7837
PPEF1	0.728251	2.02E-71	5475
THY1	0.726764	5.36E-71	7070
GLT8D2	0.726573	6.07E-71	83468
C5orf46	0.726047	8.55E-71	389336
LZTS1	0.72551	1.21E-70	11178
GPR68	0.7248	1.92E-70	8111
ZNF469	0.724453	2.40E-70	84627
PPFIA2	0.723362	4.85E-70	8499
CTSK	0.722376	9.14E-70	1513
PKD2	0.722148	1.06E-69	5311
COL15A1	0.7221	1.09E-69	1306
LPAR4	0.720906	2.34E-69	2846
TNFSF4	0.720714	2.64E-69	7292
CSGALNACT2	0.718476	1.09E-68	55454
MRC2	0.718258	1.25E-68	9902
ADAMTS2	0.7181	1.38E-68	9509
CDK14	0.71603	5.03E-68	5218
ITGAV	0.71512	8.86E-68	3685
SGIP1	0.713602	2.27E-67	84251
PABPC4L	0.713393	2.58E-67	132430
ATP10A	0.712534	4.37E-67	57194
GGT5	0.712433	4.65E-67	2687
HOPX	0.712357	4.87E-67	84525
IQCA1	0.711675	7.40E-67	79781
WNT2	0.710033	2.01E-66	7472
PDGFRL	0.709197	3.34E-66	5157
NRP1	0.708415	5.35E-66	8829
MMP14	0.707837	7.58E-66	4323
ALPK2	0.70745	9.57E-66	115701
MMP16	0.701938	2.52E-64	4325
FN1	0.701689	2.92E-64	2335
CYP1B1	0.700994	4.38E-64	1545
HMCN1	0.700541	5.71E-64	83872
GJA1	0.699903	8.28E-64	2697

MRAS	0.69892	1.47E-63	22808
ISLR	0.697988	2.51E-63	3671
RAI14	0.697175	4.01E-63	26064
ZNF521	0.697088	4.22E-63	25925
ENTPD1	0.696881	4.75E-63	953
ADAMTS16	0.696704	5.26E-63	170690
ADAMTS5	0.695948	8.11E-63	11096
GNB4	0.695217	1.23E-62	59345
FRMD6	0.694267	2.12E-62	122786
GPX8	0.693601	3.09E-62	493869
RCN3	0.693216	3.84E-62	57333
COL24A1	0.69271	5.11E-62	255631
MSC	0.690444	1.83E-61	9242
GAS1	0.689889	2.49E-61	2619
SCARF2	0.68896	4.18E-61	91179
ARSI	0.688117	6.68E-61	340075
TIMP2	0.685413	2.96E-60	7077
SERPINF1	0.684398	5.17E-60	5176
CYP7B1	0.683623	7.88E-60	9420
SYNDIG1	0.683266	9.57E-60	79953
SDC2	0.682673	1.32E-59	6383
CLEC5A	0.681779	2.14E-59	23601
ANGPTL2	0.681083	3.12E-59	23452
SLITRK4	0.680984	3.29E-59	139065
NID2	0.680663	3.91E-59	22795
CHST1	0.67959	6.96E-59	8534
LHFP	0.678975	9.66E-59	NA
NOTCH3	0.678625	1.17E-58	4854
GPR1	0.67813	1.52E-58	2825
PODNL1	0.678061	1.57E-58	79883
CCDC8	0.677591	2.02E-58	83987
MSR1	0.676871	2.96E-58	4481
COL16A1	0.676192	4.24E-58	1307
VEGFC	0.674719	9.22E-58	7424
RUNX2	0.674049	1.31E-57	860
ZNF532	0.673962	1.37E-57	55205
SERPING1	0.67363	1.63E-57	710
GDF6	0.672182	3.47E-57	392255
LPPR4	0.671697	4.47E-57	NA
MMP2	0.671535	4.86E-57	4313
ERMN	0.670447	8.54E-57	57471
FAM20A	0.670335	9.04E-57	54757
SNAI2	0.669787	1.20E-56	6591
LMCD1	0.66922	1.61E-56	29995
DKK3	0.666358	6.94E-56	27122
EVC	0.665982	8.40E-56	2121
COMP	0.665332	1.17E-55	1311
SFRP2	0.663867	2.45E-55	6423
PDPN	0.663092	3.61E-55	10630
CTGF	0.662941	3.90E-55	NA
MOV10L1	0.662886	4.01E-55	54456
KAL1	0.662213	5.62E-55	NA
PCDH17	0.661103	9.77E-55	27253
QKI	0.660923	1.07E-54	9444
GPNMB	0.660661	1.22E-54	10457
PRR16	0.659349	2.34E-54	51334
PCOLCE	0.658729	3.17E-54	5118
PCDHGA12	0.658687	3.24E-54	26025
GLIS2	0.657526	5.74E-54	84662
MEDAG	0.656664	8.76E-54	84935

IGFBP7	0.656526	9.37E-54	3490
FNDC4	0.65452	2.49E-53	64838
LAMA4	0.654386	2.66E-53	3910
HEG1	0.654374	2.68E-53	57493
HTRA4	0.653135	4.88E-53	203100
GREM1	0.652704	6.01E-53	26585
PLXNC1	0.652673	6.10E-53	10154
CALCRL	0.652661	6.13E-53	10203
SPOCD1	0.651923	8.75E-53	90853
CLEC11A	0.65144	1.10E-52	6320
CHST11	0.650907	1.43E-52	50515
MYH10	0.650063	2.13E-52	4628
TCF4	0.649816	2.40E-52	6925
ELK3	0.649289	3.09E-52	2004
LOXL1	0.647485	7.27E-52	4016
C1S	0.647086	8.78E-52	716
GPR116	0.645966	1.49E-51	NA
P3H1	0.645376	1.96E-51	64175
SLC1A3	0.644959	2.39E-51	6507
FKBP7	0.644056	3.64E-51	51661
CD84	0.644002	3.74E-51	8832
HHIPL1	0.643966	3.80E-51	84439
CCIN	0.643732	4.24E-51	881
AQP9	0.642235	8.51E-51	366
COL18A1	0.642042	9.31E-51	80781
VIM	0.641997	9.50E-51	7431
PRKD1	0.641952	9.70E-51	5587
CPXM1	0.641305	1.31E-50	56265
BEND6	0.641118	1.43E-50	221336
LOXL2	0.640651	1.77E-50	4017
SLC11A1	0.639363	3.20E-50	6556
C1QTNF6	0.638473	4.82E-50	114904
EVC2	0.638331	5.14E-50	132884
EBF2	0.638319	5.17E-50	64641
MCC	0.637742	6.72E-50	4163
EFEMP2	0.637621	7.10E-50	30008
SGCD	0.637204	8.59E-50	6444
PLXNA4	0.636058	1.45E-49	91584
CALU	0.635586	1.79E-49	813
HS3ST3A1	0.635298	2.04E-49	9955
SLC6A1	0.634898	2.44E-49	6529
AFAP1L1	0.632298	7.86E-49	134265
MMP19	0.631705	1.02E-48	4327
FCGR3A	0.629009	3.39E-48	2214
CPZ	0.628521	4.21E-48	8532
LTBP1	0.628015	5.26E-48	4052
MAFB	0.626918	8.52E-48	9935
KIRREL	0.626818	8.90E-48	NA
FUT11	0.626511	1.02E-47	170384
C10orf10	0.626096	1.22E-47	NA
PTGIR	0.625481	1.60E-47	5739
THBS1	0.625234	1.78E-47	7057
MFGE8	0.625172	1.83E-47	4240
BCL6B	0.625063	1.92E-47	255877
TLR2	0.624612	2.33E-47	7097
FILIP1L	0.624317	2.65E-47	11259
NREP	0.624251	2.73E-47	9315
UBE2E2	0.624111	2.90E-47	7325
TWIST1	0.623794	3.32E-47	7291
LRRC32	0.623442	3.87E-47	2615

DACT1	0.623417	3.91E-47	51339
TMEM45A	0.623114	4.46E-47	55076
IL1R1	0.622251	6.48E-47	3554
BASP1	0.621893	7.56E-47	10409
ELTD1	0.621673	8.31E-47	NA
CLIC4	0.621508	8.92E-47	25932
PLXDC1	0.621503	8.94E-47	57125
MN1	0.620822	1.20E-46	4330
PCDH12	0.620759	1.23E-46	51294
OSMR	0.620552	1.34E-46	9180
LBH	0.620341	1.47E-46	81606
CHST3	0.620325	1.48E-46	9469
MITF	0.619268	2.33E-46	4286
MOXD1	0.618846	2.79E-46	26002
F2R	0.618572	3.13E-46	2149
PPP1R18	0.617888	4.19E-46	170954
CORIN	0.617177	5.66E-46	10699
TENM3	0.616322	8.12E-46	55714
ADAMTS7	0.616073	9.02E-46	11173
COL4A1	0.615848	9.92E-46	1282
TM6SF1	0.61536	1.22E-45	53346
FCGR1A	0.615318	1.24E-45	2209
KIF26B	0.614937	1.45E-45	55083
MEX3B	0.614707	1.60E-45	84206
ADAMTS4	0.613472	2.69E-45	9507
EPYC	0.612803	3.55E-45	1833
COL6A2	0.612683	3.73E-45	1292
CEP170	0.612283	4.41E-45	9859
ITGAX	0.611854	5.27E-45	3687
4-9月	0.611577	5.91E-45	NA
OSCAR	0.610393	9.66E-45	126014
TREM1	0.610263	1.02E-44	54210
CILP2	0.610181	1.05E-44	148113
PALM2-AKAP2	0.609421	1.44E-44	NA
C5AR1	0.608023	2.56E-44	728
CCDC102B	0.607816	2.79E-44	79839
GSC	0.606789	4.24E-44	145258
MATN3	0.60604	5.75E-44	4148
MFAP2	0.604255	1.19E-43	4237
ADAMTS10	0.602571	2.34E-43	81794
PIEZO2	0.601939	3.01E-43	63895
VASH1	0.601602	3.45E-43	22846
LCA5	0.601494	3.60E-43	167691
CCDC88A	0.601386	3.76E-43	55704
SERPINH1	0.599	9.73E-43	871
RBMS1	0.598631	1.13E-42	5937
C1R	0.598225	1.32E-42	715
TGFBR1	0.596332	2.79E-42	7046
TREM2	0.596037	3.13E-42	54209
HAVCR2	0.595357	4.09E-42	84868
CTSL	0.595088	4.55E-42	1514
HTR2A	0.594584	5.54E-42	3356
PILRA	0.59364	8.01E-42	29992
COPZ2	0.593594	8.15E-42	51226
CNIH3	0.593077	9.96E-42	149111
COL6A1	0.592902	1.07E-41	1291
TMEM119	0.592636	1.18E-41	338773
COL5A3	0.592177	1.41E-41	50509
SYDE1	0.591903	1.57E-41	85360
TRPS1	0.591267	2.01E-41	7227

ITGAM	0.591133	2.12E-41	3684
COL4A2	0.590214	3.01E-41	1284
S1PR3	0.589936	3.36E-41	1903
NPR3	0.589448	4.05E-41	4883
SIGLEC9	0.589388	4.14E-41	27180
COL22A1	0.589229	4.40E-41	169044
APCDD1L	0.589157	4.52E-41	164284
FPR3	0.589145	4.55E-41	2359
TM4SF19	0.588904	4.99E-41	116211
SPON2	0.588769	5.25E-41	10417
ARHGAP31	0.588152	6.65E-41	57514
ALDH1A3	0.588041	6.93E-41	220
GLI2	0.587493	8.55E-41	2736
CLEC7A	0.587298	9.21E-41	64581
FOXS1	0.586628	1.19E-40	2307
TCTEX1D1	0.586475	1.26E-40	200132
APOC1	0.586132	1.43E-40	341
FAM63B	0.585872	1.58E-40	NA
LILRB4	0.585713	1.68E-40	11006
ZNF365	0.585653	1.72E-40	22891
SH3PXD2B	0.585525	1.81E-40	285590
CCDC81	0.585525	1.81E-40	60494
TMEM26	0.583503	3.87E-40	219623
NETO1	0.582985	4.70E-40	81832
FCGR2A	0.582965	4.74E-40	2212
TPST1	0.582605	5.42E-40	8460
PDCD1LG2	0.58194	6.95E-40	80380
IKBIP	0.581691	7.63E-40	121457
GPR85	0.581524	8.12E-40	54329
ZEB2	0.58151	8.17E-40	9839
DGKI	0.581365	8.62E-40	9162
CRISPLD2	0.580976	9.97E-40	83716
FKBP14	0.579916	1.48E-39	55033
PRAM1	0.578962	2.11E-39	84106
FAM126A	0.578866	2.18E-39	84668
C1QTNF3	0.578522	2.48E-39	114899
ZFHX4	0.578243	2.75E-39	79776
HECW2	0.577729	3.32E-39	57520
SIRPB2	0.577626	3.45E-39	284759
ZCCHC5	0.577244	3.97E-39	NA
RFTN1	0.576081	6.08E-39	23180
ZNF281	0.575467	7.62E-39	23528
ST3GAL6	0.575414	7.77E-39	10402
C14orf37	0.575343	7.97E-39	NA
MNDA	0.57533	8.01E-39	4332
IL1RAP	0.57527	8.18E-39	3556
ARL10	0.575128	8.62E-39	285598
LAYN	0.575042	8.90E-39	143903
CAMK1G	0.574801	9.72E-39	57172
TNFSF13B	0.574379	1.13E-38	10673
RUNX1	0.574235	1.19E-38	861
CYR61	0.574144	1.23E-38	NA
ARL4C	0.57403	1.29E-38	10123
ALOX5AP	0.573384	1.63E-38	241
MYO5A	0.57307	1.82E-38	4644
ITPRIP	0.572962	1.90E-38	85450
EMR2	0.572463	2.27E-38	NA
KDELC1	0.572128	2.56E-38	NA
NNMT	0.571772	2.92E-38	4837
LATS2	0.571563	3.15E-38	26524

RFTN2	0.570682	4.32E-38	130132
FMO2	0.570401	4.78E-38	2327
DZIP1L	0.570327	4.91E-38	199221
PHTF2	0.56999	5.54E-38	57157
CHN1	0.569553	6.48E-38	1123
FAT3	0.569366	6.93E-38	120114
UBTD1	0.569089	7.65E-38	80019
CD93	0.568365	9.91E-38	22918
TIMP3	0.568277	1.02E-37	7078
EFEMP1	0.568187	1.06E-37	2202
MEIS3	0.567346	1.42E-37	56917
P2RX7	0.567265	1.47E-37	5027
SCUBE3	0.56667	1.81E-37	222663
FCGR1B	0.566406	1.99E-37	2210
HRH2	0.566298	2.07E-37	3274
NLRP12	0.566198	2.14E-37	91662
HEYL	0.565339	2.90E-37	26508
FAM20C	0.565138	3.12E-37	56975
FGR	0.565011	3.26E-37	2268
GPR141	0.564194	4.34E-37	353345
PTPN14	0.564058	4.56E-37	5784
CCDC80	0.563792	5.00E-37	151887
CTSB	0.563407	5.73E-37	1508
CFH	0.563095	6.39E-37	3075
VCAM1	0.562949	6.72E-37	7412
ERG	0.562136	8.94E-37	2078
ADAMTS3	0.561782	1.01E-36	9508
AVPR1A	0.561462	1.13E-36	552
CHSY1	0.561398	1.16E-36	22856
VASN	0.5612	1.24E-36	114990
LRRC8C	0.561073	1.30E-36	84230
PCDH7	0.560985	1.34E-36	5099
GLI1	0.56059	1.53E-36	2735
ZFPM2	0.560144	1.79E-36	23414
HTR2B	0.560029	1.86E-36	3357
NTNG2	0.559613	2.15E-36	84628
PLEKHO2	0.559043	2.62E-36	80301
SRGAP2C	0.558545	3.11E-36	653464
GUCA1A	0.558504	3.15E-36	2978
MSN	0.558327	3.35E-36	4478
PDGFB	0.558229	3.47E-36	5155
SLC38A6	0.557937	3.84E-36	145389
CYBB	0.557628	4.27E-36	1536
DPYSL3	0.556628	6.02E-36	1809
KLHL4	0.556627	6.02E-36	56062
FAM101B	0.555894	7.74E-36	NA
RNF175	0.555239	9.68E-36	285533
DOK6	0.554847	1.11E-35	220164
LIMS1	0.55482	1.12E-35	3987
VSTM4	0.55439	1.29E-35	196740
CHRD	0.554222	1.37E-35	8646
NLRC4	0.553898	1.53E-35	58484
DLC1	0.553149	1.97E-35	10395
CRISPLD1	0.55302	2.06E-35	83690
NRP2	0.552952	2.11E-35	8828
C8orf34	0.551278	3.71E-35	116328
OLFML1	0.55092	4.18E-35	283298
AKT3	0.550482	4.84E-35	10000
FMNL3	0.55021	5.31E-35	91010
SRGAP2B	0.550121	5.47E-35	647135

SLAMF8	0.548884	8.27E-35	56833
TICAM2	0.548767	8.60E-35	353376
DOCK4	0.54862	9.04E-35	9732
C1orf162	0.548364	9.84E-35	128346
RAB3IL1	0.547967	1.12E-34	5866
PODN	0.547922	1.14E-34	127435
GPX7	0.547895	1.15E-34	2882
MMP11	0.546937	1.58E-34	4320
SGTB	0.546563	1.79E-34	54557
LILRB3	0.546266	1.98E-34	11025
CCDC36	0.54617	2.04E-34	NA
TIE1	0.546075	2.10E-34	7075
STC1	0.546032	2.14E-34	6781
CDO1	0.545898	2.23E-34	1036
NRK	0.544425	3.63E-34	203447
ADAMTS6	0.544222	3.88E-34	11174
WIPF1	0.543805	4.45E-34	7456
BTBD19	0.543609	4.74E-34	149478
METTL11B	0.543598	4.76E-34	149281
FLT1	0.543504	4.91E-34	2321
DIRC1	0.542585	6.64E-34	116093
RAB7B	0.542116	7.74E-34	338382
LAPTM5	0.541871	8.38E-34	7805
SOX11	0.541655	8.99E-34	6664
DCSTAMP	0.540251	1.42E-33	81501
GPR124	0.540217	1.44E-33	NA
CD86	0.539859	1.61E-33	942
CCR1	0.539821	1.63E-33	1230
RAB34	0.539779	1.65E-33	83871
MILR1	0.539167	2.02E-33	284021
PAPPA	0.538805	2.27E-33	5069
RNASE2	0.538238	2.72E-33	6036
GJA5	0.538206	2.75E-33	2702
ARMC9	0.538198	2.76E-33	80210
CDH13	0.537962	2.98E-33	1012
BCL2A1	0.53789	3.05E-33	597
ITGB1	0.537457	3.50E-33	3688
FBLN2	0.537346	3.63E-33	2199
EGR2	0.53705	3.99E-33	1959
TFEC	0.536775	4.36E-33	22797
RHOQ	0.536346	5.00E-33	23433
TWSG1	0.536221	5.21E-33	57045
MS4A14	0.536172	5.29E-33	84689
LAMB2	0.535715	6.12E-33	3913
TPRG1	0.535388	6.79E-33	285386
LY96	0.535107	7.43E-33	23643
TDRD6	0.535104	7.44E-33	221400
NOTCH4	0.535097	7.46E-33	4855
RAB8B	0.534465	9.12E-33	51762
CDKL5	0.534292	9.64E-33	6792
NCF2	0.534123	1.02E-32	4688
ATXN1	0.533532	1.23E-32	6310
FGF7	0.533363	1.29E-32	2252
LDB2	0.533306	1.32E-32	9079
CAMSAP2	0.533072	1.42E-32	23271
C3AR1	0.532944	1.48E-32	719
TMEM86A	0.532786	1.55E-32	144110
MDFIC	0.532554	1.67E-32	29969
AMZ1	0.532362	1.78E-32	155185
GPR84	0.532217	1.86E-32	53831

BNC2	0.532217	1.86E-32	54796
C3orf80	0.531812	2.11E-32	401097
CD248	0.531583	2.27E-32	57124
FCER1G	0.531559	2.29E-32	2207
LAIR1	0.531053	2.68E-32	3903
NAV1	0.530941	2.78E-32	89796
FAM19A1	0.530819	2.89E-32	NA
SLC2A3	0.530802	2.91E-32	6515
ANGPT2	0.530541	3.15E-32	285
KLF7	0.530338	3.36E-32	8609
DNAJC5B	0.530029	3.70E-32	85479
HAMP	0.529917	3.84E-32	57817
JAKMIP2	0.529843	3.93E-32	9832
ARHGAP29	0.529627	4.20E-32	9411
HS3ST2	0.5295	4.37E-32	9956
EMILIN1	0.529263	4.71E-32	11117
SPHK1	0.529191	4.82E-32	8877
PECAM1	0.528902	5.27E-32	5175
HK3	0.528767	5.50E-32	3101
RARB	0.528619	5.76E-32	5915
SLFN5	0.528452	6.07E-32	162394
SPSB1	0.528087	6.80E-32	80176
P3H3	0.528044	6.89E-32	10536
IGFBP5	0.527977	7.03E-32	3488
ANKRD6	0.52776	7.53E-32	22881
PTGER3	0.527182	9.01E-32	5733
FLRT2	0.526381	1.15E-31	23768
ZSWIM4	0.526374	1.16E-31	65249
CCR8	0.526374	1.16E-31	1237
LOXL3	0.526093	1.26E-31	84695
ARHGEF15	0.525655	1.45E-31	22899
CLEC4E	0.524931	1.81E-31	26253
SLA	0.524816	1.87E-31	6503
DSEL	0.524075	2.35E-31	92126
A4GALT	0.523796	2.56E-31	53947
AAED1	0.523246	3.04E-31	NA
PTHLH	0.522437	3.89E-31	5744
LPHN2	0.522223	4.15E-31	NA
HIVEP2	0.522191	4.19E-31	3097
SNX18	0.522158	4.24E-31	112574
HOOK3	0.522149	4.25E-31	84376
RGS16	0.522103	4.31E-31	6004
CD80	0.521579	5.05E-31	941
NAV3	0.521389	5.36E-31	89795
ZNF843	0.52137	5.39E-31	283933
NCKAP5L	0.521355	5.41E-31	57701
TRPC1	0.521139	5.78E-31	7220
PTGIS	0.520858	6.30E-31	5740
TPM4	0.520817	6.37E-31	7171
ANXA5	0.520686	6.63E-31	308
EHD3	0.518547	1.27E-30	30845
FAM198B	0.518436	1.31E-30	NA
WWC2	0.517491	1.74E-30	80014
FGF14	0.517426	1.78E-30	2259
ITGB5	0.517299	1.85E-30	3693
DENND5A	0.517193	1.91E-30	23258
ADPRH	0.516723	2.20E-30	141
PALMD	0.516533	2.33E-30	54873
NCAM2	0.516317	2.48E-30	4685
CSDC2	0.515558	3.11E-30	27254

TNFRSF9	0.515492	3.18E-30	3604
TIMP1	0.515261	3.40E-30	7076
TMEM204	0.515136	3.53E-30	79652
APOE	0.514985	3.69E-30	348
CCDC3	0.514861	3.83E-30	83643
IFFO1	0.513859	5.17E-30	25900
CLEC4D	0.513213	6.26E-30	338339
KMO	0.512871	6.93E-30	8564
BMPR2	0.512596	7.51E-30	659
SSH1	0.512211	8.42E-30	54434
OSBPL8	0.512157	8.56E-30	114882
SUCNR1	0.512111	8.67E-30	56670
RORB	0.511721	9.73E-30	6096
PTPRM	0.511564	1.02E-29	5797
SRGAP2	0.511328	1.09E-29	23380
SLFN12	0.51123	1.12E-29	55106
LAMC1	0.510556	1.37E-29	3915
SULF2	0.510121	1.56E-29	55959
DAZL	0.509666	1.78E-29	1618
SLC46A2	0.509468	1.89E-29	57864
CYP27C1	0.509408	1.92E-29	339761
CALHM2	0.509136	2.08E-29	51063
PLA2G7	0.509063	2.13E-29	7941
FOXC2	0.509026	2.15E-29	2303
MYOF	0.508989	2.17E-29	26509
SLC24A3	0.508894	2.23E-29	57419
RNF180	0.508753	2.33E-29	285671
C3	0.508395	2.58E-29	718
SIRPB1	0.507978	2.92E-29	10326
KLF17	0.507936	2.95E-29	128209
PLAT	0.507925	2.96E-29	5327
KLRD1	0.507755	3.11E-29	3824
LIPN	0.507725	3.14E-29	643418
ARHGEF17	0.50745	3.40E-29	9828
BHLHE22	0.50719	3.67E-29	27319
PIP4K2A	0.507087	3.78E-29	5305
MFRP	0.506769	4.15E-29	83552
GPR137B	0.506577	4.39E-29	7107
C11orf88	0.506522	4.46E-29	NA
KCNJ8	0.506304	4.75E-29	3764
LILRA6	0.506255	4.82E-29	79168
BMP1	0.506037	5.13E-29	649
LGALS1	0.505812	5.47E-29	3956
DRAM1	0.505585	5.85E-29	55332
PEA15	0.505511	5.97E-29	8682
ELN	0.504808	7.32E-29	2006
DZIP1	0.50434	8.37E-29	22873
PXDNL	0.504287	8.50E-29	137902
HAPLN3	0.503638	1.02E-28	145864
WWTR1	0.503476	1.07E-28	25937
CTIF	0.503467	1.08E-28	9811
COLEC12	0.503039	1.22E-28	81035
EFS	0.502978	1.24E-28	10278
FHL3	0.502016	1.63E-28	2275
THSD7A	0.501827	1.72E-28	221981
GRP	0.501725	1.77E-28	2922
FLT4	0.501277	2.01E-28	2324
BACE1	0.500613	2.43E-28	23621
NLGN2	0.50054	2.49E-28	57555
CCL2	0.499955	2.93E-28	6347

SNED1	0.499883	2.99E-28	25992
DCN	0.499651	3.20E-28	1634
GUCY1B3	0.499632	3.22E-28 NA	
RNF144A	0.499202	3.63E-28	9781
SNX10	0.498711	4.17E-28	29887
GEM	0.498337	4.64E-28	2669
LILRB2	0.498145	4.90E-28	10288
OMD	0.497896	5.25E-28	4958
KERA	0.497755	5.47E-28	11081
GPR4	0.497542	5.81E-28	2828
RUNX1T1	0.497399	6.05E-28	862
ZNF667	0.497376	6.08E-28	63934
ENG	0.497311	6.20E-28	2022
HSD11B1	0.497166	6.45E-28	3290
SSC5D	0.497044	6.68E-28	284297
PURG	0.497026	6.71E-28	29942
ITGB2	0.49693	6.90E-28	3689
GLI3	0.496177	8.52E-28	2737
LAMP5	0.495937	9.11E-28	24141
FZD4	0.495764	9.57E-28	8322
CD300C	0.49532	1.08E-27	10871
PTPN12	0.495248	1.11E-27	5782
MMP9	0.49496	1.20E-27	4318
LCP2	0.494682	1.29E-27	3937
MPDZ	0.494476	1.37E-27	8777
ABCA1	0.494471	1.37E-27	19
EGFLAM	0.49432	1.43E-27	133584
MAP3K12	0.494227	1.47E-27	7786
CNPY4	0.494102	1.52E-27	245812
MAP4K4	0.493589	1.76E-27	9448
GJA4	0.493232	1.94E-27	2701
B3GNT9	0.492986	2.08E-27	84752
TGFB1	0.492612	2.30E-27	7040
KLHL5	0.492337	2.48E-27	51088
DCBLD1	0.492236	2.55E-27	285761
GPR65	0.492074	2.67E-27	8477
ALDH1L2	0.492012	2.72E-27	160428
APBA2	0.491852	2.84E-27	321
APELA	0.491439	3.18E-27	100506013
GLT1D1	0.491386	3.23E-27	144423
SFMBT2	0.490903	3.69E-27	57713
PHLDB2	0.490875	3.72E-27	90102
ALPL	0.490162	4.53E-27	249
CDH6	0.490111	4.59E-27	1004
SH3PXD2A	0.489464	5.48E-27	9644
ETS1	0.489273	5.78E-27	2113
MAP1B	0.489123	6.02E-27	4131
HEY2	0.489065	6.12E-27	23493
GM2A	0.488899	6.40E-27	2760
CCL7	0.488476	7.19E-27	6354
TBCEL	0.488376	7.39E-27	219899
PNMAL2	0.487801	8.64E-27 NA	
SLC12A4	0.487748	8.77E-27	6560
GUCY1A3	0.487739	8.79E-27 NA	
THBS3	0.487645	9.02E-27	7059
EOGT	0.48749	9.41E-27	285203
ATP8B2	0.48726	1.00E-26	57198
MACF1	0.486788	1.14E-26	23499
GRM2	0.486523	1.22E-26	2912
AXL	0.486441	1.25E-26	558

RXFP1	0.486269	1.31E-26	59350
FAM160B1	0.485724	1.52E-26	57700
SOCS5	0.485618	1.56E-26	9655
KDR	0.485299	1.71E-26	3791
NXN	0.484896	1.90E-26	64359
G0S2	0.484705	2.00E-26	50486
CRYGN	0.484574	2.07E-26	155051
RASSF8	0.484467	2.13E-26	11228
SOD2	0.484366	2.19E-26	6648
RUSC2	0.483966	2.44E-26	9853
HIP1	0.48263	3.50E-26	3092
SSPN	0.482525	3.60E-26	8082
KIF3C	0.482245	3.88E-26	3797
LPPR2	0.481977	4.17E-26	NA
NOTCH2	0.48184	4.32E-26	4853
SLIT2	0.481694	4.50E-26	9353
ADAMTS14	0.481525	4.70E-26	140766
FZD1	0.481208	5.12E-26	8321
TRPV2	0.481126	5.23E-26	51393
SEMA6B	0.480989	5.43E-26	10501
EPHA3	0.480442	6.28E-26	2042
BCL6	0.480272	6.57E-26	604
FAM180A	0.480141	6.80E-26	389558
TNFRSF8	0.479911	7.23E-26	943
ESR1	0.479599	7.86E-26	2099
SDS	0.479439	8.20E-26	10993
ACVR1	0.479103	8.96E-26	90
ENTHD1	0.479038	9.12E-26	150350
PXDC1	0.478851	9.58E-26	221749
PTAFR	0.478521	1.05E-25	5724
FPR1	0.478516	1.05E-25	2357
PDE1A	0.478243	1.13E-25	5136
NFAM1	0.478178	1.14E-25	150372
S1PR5	0.478135	1.16E-25	53637
PANX1	0.478103	1.17E-25	24145
MAP3K3	0.477422	1.40E-25	4215
ICAM1	0.477106	1.52E-25	3383
LILRA2	0.476886	1.61E-25	11027
SNAI1	0.476346	1.85E-25	6615
FCGR2B	0.47623	1.91E-25	2213
SLC15A4	0.476078	1.99E-25	121260
KIAA1462	0.475795	2.14E-25	NA
EMILIN2	0.475375	2.39E-25	84034
SNX29	0.475246	2.48E-25	92017
CSF3R	0.475206	2.50E-25	1441
FGD5	0.475093	2.58E-25	152273
SIGLEC5	0.47501	2.63E-25	8778
RECQL	0.474687	2.87E-25	5965
FBXL7	0.474212	3.24E-25	23194
GLRB	0.474152	3.29E-25	2743
CLEC4A	0.474139	3.31E-25	50856
CHI3L1	0.474053	3.38E-25	1116
HSPA12B	0.473769	3.64E-25	116835
GNAI2	0.473659	3.75E-25	2771
APLNR	0.473653	3.75E-25	187
FCHSD2	0.47358	3.82E-25	9873
ZNF25	0.473381	4.03E-25	219749
ARID5B	0.473245	4.17E-25	84159
KCNJ5	0.473242	4.18E-25	3762
TNC	0.472741	4.76E-25	3371

ARMCX2		0.472723	4.78E-25	9823
	1-3月	0.472442	5.14E-25	NA
SHISA4		0.471989	5.78E-25	149345
KIAA1755		0.471462	6.62E-25	85449
SPP1		0.471261	6.98E-25	6696
ABCC9		0.471242	7.01E-25	10060
ESAM		0.470973	7.52E-25	90952
SPTA1		0.470963	7.54E-25	6708
VGLL4		0.470377	8.76E-25	9686
KIFC3		0.469725	1.04E-24	3801
WDPCP		0.469552	1.08E-24	51057
ARSB		0.469075	1.22E-24	411
FBLN7		0.469067	1.23E-24	129804
TLR1		0.468686	1.35E-24	7096
SRGN		0.468489	1.42E-24	5552
CCDC71L		0.468146	1.55E-24	168455
RFX8		0.468145	1.55E-24	731220
MAP3K7CL		0.468132	1.56E-24	56911
GPR161		0.46812	1.56E-24	23432
FSTL3		0.467975	1.62E-24	10272
C8orf48		0.467203	1.98E-24	157773
HEY1		0.467078	2.04E-24	23462
PACS1		0.466638	2.28E-24	55690
TSHZ3		0.466442	2.40E-24	57616
IDS		0.466414	2.42E-24	3423
SORCS2		0.465416	3.11E-24	57537
USP44		0.46534	3.17E-24	84101
C10orf55		0.465307	3.20E-24	414236
RASAL2		0.465245	3.25E-24	9462
DYRK3		0.465175	3.31E-24	8444
GBP5		0.464582	3.84E-24	115362
PLS3		0.464452	3.97E-24	5358
STAT2		0.464389	4.04E-24	6773
STON1		0.464321	4.11E-24	11037
ATP10D		0.464217	4.22E-24	57205
SLC15A3		0.464073	4.37E-24	51296
PLEKHG2		0.464004	4.45E-24	64857
LRRC25		0.463837	4.64E-24	126364
SYT11		0.463615	4.91E-24	23208
ZPLD1		0.463436	5.13E-24	131368
PABPC5		0.463268	5.35E-24	140886
THEMIS2		0.463057	5.65E-24	9473
NLRP3		0.462942	5.81E-24	114548
SFXN3		0.462725	6.14E-24	81855
CYTH3		0.462676	6.21E-24	9265
LOXHD1		0.4626	6.33E-24	125336
IRAK3		0.462587	6.35E-24	11213
GJB2		0.462224	6.96E-24	2706
TSHZ2		0.46214	7.11E-24	128553
CHST15		0.461985	7.39E-24	51363
PLAU		0.461683	7.97E-24	5328
LIX1L		0.460943	9.59E-24	128077
CD83		0.460829	9.87E-24	9308
ZNF208		0.460063	1.19E-23	7757
HDAC7		0.459973	1.22E-23	51564
PRRX2		0.45977	1.28E-23	51450
CXorf36		0.459745	1.29E-23	NA
IL4I1		0.459573	1.35E-23	259307
SERPINE1		0.459523	1.37E-23	5054
RASGRF2		0.459197	1.48E-23	5924

FAM168A	0.459126	1.51E-23	23201
FMO3	0.458866	1.61E-23	2328
ACP5	0.458377	1.82E-23	54
SPI1	0.458299	1.85E-23	6688
IGFBP3	0.457939	2.02E-23	3486
GALNT15	0.457523	2.24E-23	117248
SLC10A6	0.457454	2.28E-23	345274
YPEL4	0.457363	2.33E-23	219539
PDE1B	0.457106	2.48E-23	5153
FST	0.456762	2.70E-23	10468
SLFN11	0.456749	2.71E-23	91607
SEMA7A	0.456691	2.75E-23	8482
ADRBK2	0.456471	2.91E-23	NA
NPL	0.456419	2.94E-23	80896
MCTP1	0.456405	2.95E-23	79772
CD276	0.456308	3.02E-23	80381
SPRED3	0.45621	3.10E-23	399473
MCHR1	0.45605	3.22E-23	2847
CRIP2	0.455981	3.28E-23	1397
VMO1	0.455665	3.54E-23	284013
RHOJ	0.455407	3.77E-23	57381
MARCO	0.455334	3.84E-23	8685
ARL13B	0.455049	4.12E-23	200894
AMIGO2	0.454991	4.18E-23	347902
BICD1	0.4549	4.27E-23	636
ZNF267	0.454368	4.87E-23	10308
TRO	0.454358	4.88E-23	7216
HSPG2	0.454051	5.26E-23	3339
BBS9	0.45402	5.30E-23	27241
FAM65A	0.453786	5.61E-23	NA
HIC1	0.453592	5.88E-23	3090
CLEC1A	0.453484	6.04E-23	51267
BMP8A	0.453464	6.07E-23	353500
CDH5	0.453384	6.19E-23	1003
WNK3	0.453381	6.19E-23	65267
IGSF6	0.453127	6.59E-23	10261
UACA	0.453116	6.60E-23	55075
FAM228A	0.452378	7.90E-23	653140
GIT2	0.452309	8.03E-23	9815
RORA	0.452077	8.50E-23	6095
AMPH	0.451892	8.89E-23	273
ZFP92	0.451777	9.14E-23	139735
EVI2A	0.451581	9.59E-23	2123
PLXND1	0.451429	9.94E-23	23129
SOX7	0.450711	1.18E-22	83595
FOXP3	0.450441	1.26E-22	50943
MURC	0.450361	1.29E-22	NA
SAMD4A	0.450123	1.36E-22	23034
CARD8	0.449909	1.44E-22	22900
PCDHGA4	0.449872	1.45E-22	56111
CDYL2	0.449756	1.49E-22	124359
BICD2	0.449569	1.56E-22	23299
PNRC1	0.449469	1.60E-22	10957
RBFOX2	0.449325	1.65E-22	23543
HTR1B	0.449323	1.65E-22	3351
IFI16	0.448998	1.79E-22	3428
RASGRP3	0.448938	1.81E-22	25780
ANO6	0.448427	2.05E-22	196527
RASSF4	0.448092	2.22E-22	83937
GPRC5B	0.448065	2.23E-22	51704

DLG4	0.447778	2.39E-22	1742
THBD	0.447382	2.63E-22	7056
FGD6	0.446953	2.91E-22	55785
MCEMP1	0.446841	2.99E-22	199675
MFAP5	0.446608	3.16E-22	8076
SIRPA	0.446239	3.45E-22	140885
ANXA1	0.446223	3.47E-22	301
THSD1	0.44618	3.50E-22	55901
HGF	0.445682	3.94E-22	3082
ZNF99	0.445524	4.09E-22	7652
KATNAL1	0.445431	4.19E-22	84056
STARD8	0.445346	4.27E-22	9754
PRPH2	0.445288	4.33E-22	5961
PDGFRA	0.445232	4.39E-22	5156
KCNE1	0.445111	4.51E-22	3753
HSD17B14	0.44489	4.76E-22	51171
TSPAN4	0.444789	4.87E-22	7106
HIF1A	0.444645	5.04E-22	3091
SIGLEC7	0.444514	5.20E-22	27036
ABL2	0.444392	5.35E-22	27
FAM124B	0.444243	5.54E-22	79843
MYO1F	0.444211	5.59E-22	4542
CATSPER1	0.443958	5.93E-22	117144
PBX3	0.443851	6.08E-22	5090
CLEC6A	0.443485	6.63E-22	93978
SIGLEC14	0.443364	6.82E-22	100049587
XIRP1	0.443339	6.86E-22	165904
B4GALNT1	0.443011	7.41E-22	2583
ADAP2	0.44251	8.34E-22	55803
SYNE1	0.442273	8.82E-22	23345
C5AR2	0.442232	8.91E-22	27202
ITPRIPL2	0.442065	9.26E-22	162073
ZNF454	0.441867	9.70E-22	285676
APBB2	0.441462	1.07E-21	323
SCPEP1	0.441458	1.07E-21	59342
SDK1	0.441431	1.07E-21	221935
RECK	0.441266	1.12E-21	8434
LILRB1	0.441152	1.15E-21	10859
TFPI	0.441133	1.15E-21	7035
SESTD1	0.441132	1.15E-21	91404
PHLDB1	0.440583	1.31E-21	23187
TMEM43	0.440323	1.39E-21	79188
C1QTNF1	0.439888	1.54E-21	114897
ANKRD50	0.439843	1.56E-21	57182
OSTM1	0.439676	1.62E-21	28962
ITGA1	0.439668	1.62E-21	3672
DDR2	0.439603	1.65E-21	4921
TMEM47	0.439447	1.71E-21	83604
FNDC3B	0.439358	1.74E-21	64778
PGM2L1	0.439272	1.78E-21	283209
EHD2	0.439237	1.79E-21	30846
C1orf216	0.439036	1.88E-21	127703
CBLB	0.438932	1.92E-21	868
SYNPO2L	0.438909	1.93E-21	79933
FAM151B	0.438844	1.96E-21	167555
TLR6	0.438628	2.06E-21	10333
DEGS1	0.438586	2.08E-21	8560
PREX2	0.438499	2.13E-21	80243
NID1	0.438242	2.26E-21	4811
ASAP1	0.437961	2.41E-21	50807

PI4K2A	0.437961	2.41E-21	55361
SELE	0.437755	2.53E-21	6401
S1PR2	0.437681	2.57E-21	9294
SIRPD	0.43751	2.67E-21	128646
RBMS3	0.436634	3.27E-21	27303
EPGN	0.436569	3.32E-21	255324
KIAA0930	0.436456	3.41E-21	23313
SLC39A13	0.436289	3.55E-21	91252
PHLDA3	0.436	3.79E-21	23612
SLC39A6	0.435847	3.92E-21	25800
SNTB2	0.435814	3.95E-21	6645
GNA12	0.435603	4.15E-21	2768
CMYA5	0.435285	4.46E-21	202333
TYROBP	0.434965	4.80E-21	7305
DSTYK	0.434963	4.81E-21	25778
FAM127C	0.43463	5.19E-21	NA
TRPV4	0.434621	5.20E-21	59341
FAM110B	0.434589	5.24E-21	90362
KCNK13	0.434499	5.35E-21	56659
TMX3	0.434175	5.76E-21	54495
MSANTD3-TMEFF1	0.433967	6.04E-21	100526694
CARD6	0.433937	6.08E-21	84674
DCHS1	0.433794	6.28E-21	8642
FRMD4A	0.433728	6.38E-21	55691
FGFR1	0.433538	6.66E-21	2260
CSRNP2	0.433476	6.75E-21	81566
CD34	0.433401	6.87E-21	947
AMPD3	0.433341	6.96E-21	272
CETP	0.433216	7.17E-21	1071
TEAD1	0.432945	7.62E-21	7003
C1orf54	0.432724	8.02E-21	79630
GSDMC	0.432715	8.03E-21	56169
EVL	0.432505	8.42E-21	51466
BPGM	0.432404	8.62E-21	669
PCNX	0.432297	8.83E-21	NA
LAMB1	0.431955	9.54E-21	3912
PAM	0.431952	9.55E-21	5066
RGS17	0.431834	9.81E-21	26575
PRR34	0.431779	9.93E-21	55267
TNS3	0.431634	1.03E-20	64759
ZNF436	0.431633	1.03E-20	80818
CD300E	0.431504	1.06E-20	342510
MGP	0.431416	1.08E-20	4256
ANGPT4	0.4314	1.08E-20	51378
TBC1D19	0.431224	1.13E-20	55296
MAP4K5	0.430179	1.43E-20	11183
RGS1	0.430092	1.45E-20	5996
MSANTD3	0.430052	1.47E-20	91283
RIC1	0.430039	1.47E-20	57589
KLHL38	0.429841	1.54E-20	340359
ADAMTS9	0.429749	1.57E-20	56999
PRKG1	0.429049	1.84E-20	5592
ADAM17	0.42881	1.94E-20	6868
BOC	0.428756	1.96E-20	91653
PRND	0.428725	1.98E-20	23627
ACSL1	0.428707	1.99E-20	2180
DYSF	0.428581	2.04E-20	8291
PIK3CA	0.42832	2.17E-20	5290
TCEAL3	0.428117	2.27E-20	85012
ENOX1	0.427908	2.38E-20	55068

GP5	0.427789	2.44E-20	2814
NKIRAS1	0.427188	2.79E-20	28512
OPN1SW	0.427094	2.85E-20	611
HS3ST3B1	0.427033	2.89E-20	9953
CHST14	0.426852	3.01E-20	113189
HOXC8	0.426019	3.62E-20	3224
SH3BP5	0.426002	3.64E-20	9467
NME8	0.425717	3.87E-20	51314
ELOVL4	0.425614	3.96E-20	6785
CDK17	0.425007	4.53E-20	5128
STX2	0.425005	4.54E-20	2054
TRIM6	0.425004	4.54E-20	117854
HECTD2	0.424911	4.63E-20	143279
CYP46A1	0.424731	4.82E-20	10858
PTRF	0.424632	4.93E-20	NA
CASC10	0.424608	4.95E-20	NA
HIGD1B	0.424576	4.99E-20	51751
TCF23	0.424018	5.64E-20	150921
NUDT10	0.42375	5.99E-20	170685
ENAH	0.423727	6.02E-20	55740
SOCS3	0.423725	6.02E-20	9021
TRPC3	0.42359	6.20E-20	7222
CYYR1	0.423133	6.86E-20	116159
MMD	0.422962	7.12E-20	23531
CCDC184	0.422943	7.15E-20	387856
AF165138.7	0.422849	7.30E-20	NA
DOCK10	0.422722	7.51E-20	55619
RDX	0.422427	8.01E-20	5962
TPBG	0.422359	8.13E-20	7162
STX11	0.42223	8.37E-20	8676
ATL3	0.42215	8.52E-20	25923
PRAF2	0.422002	8.80E-20	11230
TBXA2R	0.421999	8.80E-20	6915
ITGA5	0.421978	8.85E-20	3678
TWIST2	0.421973	8.85E-20	117581
EBF3	0.421884	9.03E-20	253738
FMNL1	0.421787	9.22E-20	752
ZFP36L1	0.421544	9.73E-20	677
RANBP3L	0.421291	1.03E-19	202151
TSKS	0.421183	1.05E-19	60385
C7orf60	0.42112	1.07E-19	NA
TRIM23	0.42112	1.07E-19	373
TECTA	0.421079	1.08E-19	7007
NPC1	0.420949	1.11E-19	4864
NIN	0.420894	1.12E-19	51199
ZDHHC15	0.420797	1.15E-19	158866
ALOX15B	0.420721	1.16E-19	247
WDR47	0.420705	1.17E-19	22911
DUSP7	0.420613	1.19E-19	1849
FYN	0.420531	1.21E-19	2534
ITGB8	0.42036	1.26E-19	3696
S100A2	0.420307	1.28E-19	6273
MRC1	0.420103	1.33E-19	4360
CSMD2	0.419874	1.40E-19	114784
ADAM19	0.419572	1.50E-19	8728
MMP1	0.419485	1.53E-19	4312
TMCC1	0.419414	1.55E-19	23023
CEP112	0.41927	1.60E-19	201134
FAM167B	0.419078	1.67E-19	84734
HOXC9	0.419012	1.69E-19	3225

TIAM2	0.419	1.69E-19	26230
TTBK2	0.418906	1.73E-19	146057
RNF24	0.418674	1.82E-19	11237
TGFB2	0.418533	1.88E-19	7042
LRRC17	0.418489	1.89E-19	10234
MARVELD1	0.41841	1.93E-19	83742
ST8SIA2	0.418183	2.02E-19	8128
IFI30	0.418095	2.06E-19	10437
RGS4	0.417951	2.13E-19	5999
SPG20	0.417934	2.14E-19	NA
GNS	0.417774	2.21E-19	2799
AIDA	0.417727	2.23E-19	64853
CLEC14A	0.417679	2.26E-19	161198
PPM1F	0.417601	2.30E-19	9647
SEC14L1	0.417597	2.30E-19	6397
FAM198A	0.417442	2.38E-19	NA
FTO	0.41714	2.54E-19	79068
MRAP	0.417125	2.54E-19	56246
IGFL3	0.417122	2.55E-19	388555
ZNF438	0.417021	2.60E-19	220929
C4A	0.416867	2.69E-19	720
CLIP1	0.416561	2.87E-19	6249
SEC31A	0.416339	3.01E-19	22872
RBMS2	0.416325	3.02E-19	5939
BEST1	0.416304	3.04E-19	7439
UBE2H	0.416301	3.04E-19	7328
FCAR	0.41628	3.05E-19	2204
PRR29	0.416185	3.12E-19	92340
PIAS3	0.416024	3.23E-19	10401
SAMSN1	0.416012	3.23E-19	64092
FAM196B	0.415701	3.46E-19	NA
IGSF21	0.415638	3.50E-19	84966
FIGN	0.415235	3.82E-19	55137
TSPAN9	0.415227	3.83E-19	10867
FGF5	0.415191	3.86E-19	2250
TMTC3	0.415028	3.99E-19	160418
FAM43B	0.414601	4.38E-19	163933
TRIM61	0.414479	4.49E-19	391712
REEP3	0.414461	4.51E-19	221035
PLEK	0.414425	4.55E-19	5341
OSBPL10	0.414144	4.83E-19	114884
PLA1A	0.413934	5.05E-19	51365
CFAP58	0.413814	5.18E-19	159686
CERS5	0.413554	5.48E-19	91012
CD53	0.413529	5.51E-19	963
SWAP70	0.413196	5.91E-19	23075
POLK	0.413169	5.94E-19	51426
NUMBL	0.413138	5.98E-19	9253
FPR2	0.413095	6.04E-19	2358
C12orf54	0.413028	6.13E-19	121273
CCL3	0.412839	6.38E-19	6348
LYST	0.41271	6.55E-19	1130
ADARB1	0.412592	6.72E-19	104
GAB3	0.412574	6.75E-19	139716
SH2D3C	0.412408	6.99E-19	10044
WTIP	0.412249	7.23E-19	126374
PDZD2	0.412068	7.51E-19	23037
SETD7	0.412049	7.54E-19	80854
ARHGEF6	0.411997	7.63E-19	9459
GAL3ST4	0.411873	7.83E-19	79690

CREB1	0.411863	7.85E-19	1385
CSF1	0.411746	8.04E-19	1435
C11orf96	0.411609	8.28E-19	387763
PICALM	0.411366	8.72E-19	8301
FCRLB	0.411216	9.00E-19	127943
PRKAR1A	0.410846	9.73E-19	5573
PCDHGA9	0.410822	9.78E-19	56107
EMP3	0.410769	9.89E-19	2014
ANXA6	0.410657	1.01E-18	309
RBM43	0.41059	1.03E-18	375287
ZC2HC1A	0.410312	1.09E-18	51101
MYOZ3	0.410208	1.11E-18	91977
LSAMP	0.41012	1.13E-18	4045
TNFSF14	0.409573	1.27E-18	8740
TLR8	0.409533	1.28E-18	51311
CD226	0.409204	1.38E-18	10666
MYCT1	0.409041	1.42E-18	80177
CCL4	0.409028	1.43E-18	6351
BACH1	0.409007	1.43E-18	571
FYB	0.409003	1.44E-18	NA
RBPJ	0.408867	1.48E-18	3516
SAMHD1	0.408509	1.59E-18	25939
RIMKLB	0.408488	1.60E-18	57494
MMRN2	0.408234	1.69E-18	79812
ROR2	0.408154	1.72E-18	4920
WDR91	0.408058	1.75E-18	29062
ARHGAP1	0.407986	1.78E-18	392
NFYB	0.407926	1.80E-18	4801
MAP7D1	0.407657	1.90E-18	55700
EFR3A	0.407565	1.94E-18	23167
BNC1	0.407121	2.13E-18	646
ECSCR	0.407058	2.16E-18	641700
INPP4B	0.407012	2.18E-18	8821
FGF10	0.406969	2.20E-18	2255
C1orf198	0.406729	2.31E-18	84886
TAOK1	0.406726	2.31E-18	57551
ABHD2	0.406688	2.33E-18	11057
BPI	0.406664	2.34E-18	671
PARVG	0.406569	2.39E-18	64098
NLGN4Y	0.406501	2.42E-18	22829
ATP8B4	0.406401	2.47E-18	79895
PRKCDBP	0.40638	2.48E-18	NA
TNKS2	0.406261	2.55E-18	80351
CD300LF	0.406257	2.55E-18	146722
IL1RN	0.40578	2.82E-18	3557
ZFP1	0.405753	2.83E-18	162239
ROBO4	0.405734	2.84E-18	54538
HOMER3	0.405696	2.87E-18	9454
COL14A1	0.405353	3.08E-18	7373
GNG11	0.405141	3.22E-18	2791
MR1	0.405117	3.23E-18	3140
SPIN1	0.404721	3.51E-18	10927
SIGLEC10	0.404601	3.60E-18	89790
GREB1	0.404523	3.65E-18	9687
MMP13	0.403997	4.07E-18	4322
TXNDC15	0.403977	4.09E-18	79770
RUFY2	0.403956	4.11E-18	55680
EGFL6	0.40391	4.15E-18	25975
CD163	0.403741	4.30E-18	9332
GAS7	0.403486	4.53E-18	8522

EXTL2	0.403336	4.67E-18	2135
PIK3R6	0.403216	4.79E-18	146850
CREM	0.403104	4.90E-18	1390
NPR2	0.403089	4.91E-18	4882
CYTL1	0.402894	5.12E-18	54360
ABCA6	0.402697	5.33E-18	23460
IFNAR2	0.402387	5.68E-18	3455
ZBTB47	0.402277	5.81E-18	92999
BBX	0.40226	5.83E-18	56987
EIF5A2	0.402192	5.91E-18	56648
S1PR1	0.402124	5.99E-18	1901
CNN3	0.401997	6.15E-18	1266
CSRP2	0.401957	6.20E-18	1466
SOX4	0.401876	6.31E-18	6659
ELOVL5	0.401722	6.51E-18	60481
EVA1B	0.401667	6.58E-18	55194
GLIS1	0.401662	6.59E-18	148979
RARRES2	0.401551	6.74E-18	5919
STYX	0.401084	7.42E-18	6815
ZNF677	0.401075	7.43E-18	342926
CA5B	0.400815	7.84E-18	11238
MICU3	0.400687	8.04E-18	286097
GATA3	0.400636	8.13E-18	2625
CSGALNACT1	0.400244	8.80E-18	55790
CAV2	0.400136	9.00E-18	858
PALM2	0.400055	9.15E-18	NA
ZMAT3	0.39996	9.33E-18	64393
PDLIM5	0.399936	9.37E-18	10611
DNAJC24	0.399496	1.03E-17	120526
MS4A7	0.399373	1.05E-17	58475
HOXC5	0.399364	1.05E-17	3222
RNF146	0.399313	1.06E-17	81847
RPS6KA2	0.399159	1.10E-17	6196
PAPSS1	0.399147	1.10E-17	9061
ABI2	0.398942	1.15E-17	10152
DCUN1D3	0.398912	1.15E-17	123879
ZHX2	0.398883	1.16E-17	22882
ZEB1	0.398631	1.22E-17	6935
LTBP3	0.398479	1.26E-17	4054
BCL9L	0.398476	1.26E-17	283149
CD300A	0.398307	1.31E-17	11314
TBC1D2B	0.398288	1.31E-17	23102
WWC3	0.398056	1.37E-17	55841
DNAL1	0.39802	1.38E-17	83544
CEACAM4	0.397947	1.40E-17	1089
ANKRD44	0.397758	1.46E-17	91526
SEC14L2	0.397625	1.50E-17	23541
PTPLAD2	0.397607	1.50E-17	NA
GXYLT2	0.397549	1.52E-17	727936
PIK3R5	0.397337	1.59E-17	23533
TNFRSF4	0.397324	1.59E-17	7293
MAGEH1	0.397275	1.61E-17	28986
FBLN5	0.397245	1.62E-17	10516
NHSL2	0.397067	1.68E-17	340527
CNRIP1	0.39698	1.71E-17	25927
SLC38A2	0.396979	1.71E-17	54407
MBNL2	0.396536	1.87E-17	10150
EMCN	0.396252	1.98E-17	51705
CLIP4	0.39613	2.03E-17	79745
GADD45B	0.396024	2.07E-17	4616

DOK3	0.395921	2.11E-17	79930
LRP1	0.395879	2.13E-17	4035
TRAPPC3L	0.395776	2.18E-17	100128327
ZCCHC18	0.395676	2.22E-17	644353
KIAA1217	0.395568	2.27E-17	56243
IGF1	0.395545	2.28E-17	3479
IRS1	0.395396	2.35E-17	3667
FAM49A	0.394929	2.58E-17	NA
TRAM1	0.394925	2.58E-17	23471
PIK3IP1	0.39489	2.60E-17	113791
FYTTD1	0.394876	2.61E-17	84248
LRRK2	0.394756	2.67E-17	120892
SKI	0.394702	2.70E-17	6497
GULP1	0.394241	2.96E-17	51454
PARP8	0.394105	3.04E-17	79668
PLOD1	0.393842	3.21E-17	5351
ZNF512B	0.393841	3.21E-17	57473
PRCP	0.393537	3.41E-17	5547
FAM114A2	0.393512	3.42E-17	10827
PTCH2	0.393277	3.59E-17	8643
ARID4B	0.39326	3.60E-17	51742
F2RL3	0.393225	3.62E-17	9002
KLHL28	0.393037	3.76E-17	54813
MYO9B	0.392999	3.79E-17	4650
ATF7IP	0.392953	3.83E-17	55729
TANC2	0.39292	3.85E-17	26115
PSAP	0.392838	3.92E-17	5660
MIB1	0.39282	3.93E-17	57534
CCDC50	0.392759	3.98E-17	152137
COL25A1	0.392758	3.98E-17	84570
NUDT3	0.39244	4.24E-17	11165
FAM229B	0.392343	4.32E-17	619208
ACTN1	0.39221	4.44E-17	87
LRP12	0.392166	4.47E-17	29967
PAPOLG	0.392119	4.52E-17	64895
DNAJB4	0.392114	4.52E-17	11080
INMT	0.391821	4.79E-17	11185
B3GALNT1	0.391791	4.82E-17	8706
KCTD20	0.391726	4.88E-17	222658
CCR5	0.39165	4.96E-17	1234
TRIM46	0.391617	4.99E-17	80128
PHTF1	0.391396	5.21E-17	10745
WDR86	0.39127	5.34E-17	349136
ITIH3	0.391091	5.54E-17	3699
IL5	0.391046	5.59E-17	3567
KCTD11	0.390863	5.79E-17	147040
DYNC2H1	0.390693	5.99E-17	79659
DCLK3	0.390599	6.10E-17	85443
ARRDC3	0.390209	6.59E-17	57561
SLC22A16	0.390134	6.69E-17	85413
NAGK	0.390104	6.73E-17	55577
CABYR	0.389982	6.89E-17	26256
PPFIBP1	0.389907	7.00E-17	8496
CFAP54	0.389858	7.06E-17	144535
AMOTL2	0.389826	7.11E-17	51421
RIT1	0.389731	7.24E-17	6016
HPS5	0.389547	7.51E-17	11234
HIPK3	0.389337	7.82E-17	10114
SLC4A7	0.3892	8.04E-17	9497
TEK	0.389127	8.16E-17	7010

DENND2C	0.389109	8.18E-17	163259
GPR78	0.388995	8.37E-17	27201
MAML2	0.388797	8.70E-17	84441
AKAP12	0.388682	8.90E-17	9590
EIF2AK2	0.38865	8.95E-17	5610
SENP7	0.388526	9.18E-17	57337
RASSF2	0.388447	9.32E-17	9770
RIN2	0.388428	9.35E-17	54453
PLA2G15	0.38842	9.37E-17	23659
NGF	0.388405	9.40E-17	4803
ZNF697	0.388399	9.41E-17	90874
ARHGAP28	0.388074	1.00E-16	79822
7-9月	0.387917	1.03E-16	NA
TMEM132A	0.387842	1.05E-16	54972
C11orf87	0.387555	1.11E-16	399947
GADL1	0.387417	1.14E-16	339896
UVRAG	0.387358	1.15E-16	7405
EXOC5	0.387264	1.17E-16	10640
PHYHIP	0.387243	1.18E-16	9796
ADAM23	0.387222	1.18E-16	8745
IGFL2	0.386663	1.32E-16	147920
SERPINB9	0.386605	1.34E-16	5272
GRID1	0.386269	1.43E-16	2894
SLC41A1	0.38624	1.43E-16	254428
VAT1	0.385869	1.54E-16	10493
ANGPT1	0.385849	1.55E-16	284
DNM3	0.385829	1.55E-16	26052
PLD3	0.385801	1.56E-16	23646
BNIP2	0.385793	1.56E-16	663
PPM1M	0.385633	1.61E-16	132160
PMEPA1	0.385443	1.67E-16	56937
PODXL	0.385306	1.72E-16	5420
INPP5F	0.385304	1.72E-16	22876
ADCY4	0.385169	1.77E-16	196883
TRIM8	0.385151	1.77E-16	81603
SPINK7	0.385084	1.79E-16	84651
IKZF2	0.38482	1.89E-16	22807
MBD5	0.384795	1.90E-16	55777
VWF	0.384643	1.95E-16	7450
ZBTB41	0.384567	1.98E-16	360023
SACS	0.384562	1.99E-16	26278
KRT79	0.384239	2.11E-16	338785
LRCH3	0.383953	2.23E-16	84859
YAF2	0.383803	2.30E-16	10138
CLMP	0.383802	2.30E-16	79827
ZBTB46	0.383729	2.33E-16	140685
FCN3	0.3837	2.35E-16	8547
ATM	0.383677	2.36E-16	472
REL	0.383636	2.37E-16	5966
KIAA1033	0.383588	2.40E-16	NA
IGFBP4	0.383567	2.41E-16	3487
REM1	0.38355	2.41E-16	28954
FRY	0.383443	2.46E-16	10129
HLX	0.383325	2.52E-16	3142
PHF21A	0.383312	2.53E-16	51317
EBF1	0.383266	2.55E-16	1879
IMPG2	0.383222	2.57E-16	50939
ROCK1	0.383219	2.57E-16	6093
ADIPOQ	0.383115	2.63E-16	9370
DIP2C	0.38302	2.67E-16	22982

AHR	0.38295	2.71E-16	196
PRKCH	0.382901	2.74E-16	5583
PHF20L1	0.38285	2.76E-16	51105
CBL	0.382792	2.79E-16	867
DDHD1	0.382736	2.82E-16	80821
LST1	0.382658	2.87E-16	7940
RGS7BP	0.382419	3.00E-16	401190
NR2F2	0.382377	3.03E-16	7026
PCNXL4	0.382265	3.09E-16	NA
OGFRL1	0.38204	3.23E-16	79627
VKORC1	0.381832	3.36E-16	79001
ZNF720	0.381825	3.36E-16	124411
ENTPD7	0.381754	3.41E-16	57089
TMPRSS11D	0.381569	3.53E-16	9407
WDFY3	0.381435	3.62E-16	23001
RCBTB2	0.381302	3.72E-16	1102
RAMP2	0.38123	3.77E-16	10266
ACAD11	0.381205	3.79E-16	84129
ELK4	0.3811	3.86E-16	2005
SELPLG	0.381003	3.94E-16	6404
ARHGAP24	0.38073	4.15E-16	83478
HCK	0.380633	4.22E-16	3055
RILPL2	0.380587	4.26E-16	196383
FLI1	0.380516	4.32E-16	2313
DKK2	0.380397	4.42E-16	27123
CILP	0.380368	4.44E-16	8483
MCFD2	0.380357	4.45E-16	90411
SLC35B4	0.380331	4.47E-16	84912
PTGFR	0.380247	4.55E-16	5737
GRK4	0.380188	4.60E-16	2868
SCHIP1	0.380132	4.65E-16	29970
STOM	0.380049	4.72E-16	2040
JMJD1C	0.379845	4.91E-16	221037
C20orf194	0.379809	4.94E-16	25943
TMEM110	0.379801	4.95E-16	NA
MTMR9	0.379463	5.28E-16	66036
ZNF366	0.379435	5.31E-16	167465
CYLD	0.379405	5.34E-16	1540
SDCCAG8	0.379373	5.37E-16	10806
SEMA5B	0.379356	5.39E-16	54437
WBP1L	0.379332	5.41E-16	54838
PINLYP	0.379306	5.44E-16	390940
VPS13B	0.379267	5.48E-16	157680
RHOBTB1	0.379123	5.63E-16	9886
CCNJ	0.379001	5.76E-16	54619
PPT1	0.378537	6.29E-16	5538
IKZF4	0.37836	6.51E-16	64375
C16orf52	0.378296	6.59E-16	NA
EFNB3	0.378157	6.76E-16	1949
SASH1	0.378003	6.96E-16	23328
CACNA2D1	0.377791	7.25E-16	781
ZNF641	0.377703	7.37E-16	121274
ST8SIA4	0.377388	7.82E-16	7903
PEAK1	0.377365	7.85E-16	79834
P2RY6	0.377273	7.99E-16	5031
ZNF333	0.37726	8.01E-16	84449
SLC2A6	0.377242	8.04E-16	11182
RNF217	0.377077	8.29E-16	154214
SYT16	0.3769	8.57E-16	83851
MAP4	0.376875	8.61E-16	4134

LAMA2	0.376671	8.95E-16	3908
KYNU	0.376625	9.03E-16	8942
PTPRG	0.376354	9.50E-16	5793
IL21R	0.376299	9.60E-16	50615
ADAM8	0.376252	9.69E-16	101
SLC30A7	0.376244	9.70E-16	148867
KCNK2	0.37623	9.73E-16	3776
NFKB2	0.376166	9.84E-16	4791
PQLC3	0.37581	1.05E-15	NA
GPR97	0.375453	1.13E-15	NA
ZNF362	0.375429	1.13E-15	149076
IL2RA	0.375421	1.13E-15	3559
CD33	0.37538	1.14E-15	945
SCEL	0.375375	1.14E-15	8796
RAB23	0.375333	1.15E-15	51715
SLFN12L	0.375196	1.18E-15	100506736
MKL1	0.375134	1.19E-15	NA
CYTH4	0.375132	1.19E-15	27128
SAR1A	0.37503	1.22E-15	56681
CD300LB	0.374851	1.26E-15	124599
ITGA4	0.374481	1.35E-15	3676
TRIM22	0.374479	1.35E-15	10346
MFAP3	0.374436	1.36E-15	4238
ZNF135	0.374345	1.38E-15	7694
STEAP2	0.374122	1.44E-15	261729
S100PBP	0.373887	1.51E-15	64766
ALS2CR12	0.373808	1.53E-15	NA
RILPL1	0.373713	1.56E-15	353116
NABP1	0.373565	1.60E-15	64859
ACAN	0.373461	1.63E-15	176
TET1	0.373421	1.64E-15	80312
GIPC3	0.373383	1.66E-15	126326
NLGN4X	0.37338	1.66E-15	57502
KLRG1	0.373292	1.68E-15	10219
GPR132	0.373258	1.70E-15	29933
TRPC6	0.373202	1.71E-15	7225
AGPAT4	0.373084	1.75E-15	56895
CCDC176	0.373008	1.78E-15	NA
FMOD	0.372936	1.80E-15	2331
ZBED2	0.372817	1.84E-15	79413
MOB1B	0.372594	1.92E-15	92597
SAMD8	0.372466	1.96E-15	142891
WDFY2	0.371997	2.14E-15	115825
TBX15	0.371978	2.15E-15	6913
ZBTB8A	0.371976	2.15E-15	653121
REST	0.371889	2.19E-15	5978
ZNF732	0.371864	2.20E-15	654254
PLEKHG1	0.371757	2.24E-15	57480
KPNA5	0.371515	2.34E-15	3841
SLC45A1	0.371286	2.44E-15	50651
ARHGAP23	0.371223	2.47E-15	57636
CCSER2	0.371223	2.47E-15	54462
CEP135	0.371174	2.49E-15	9662
IL6ST	0.371145	2.51E-15	3572
BTF3L4	0.371091	2.53E-15	91408
SLC6A6	0.371065	2.55E-15	6533
CCL18	0.371054	2.55E-15	6362
CREBRF	0.370687	2.73E-15	153222
LY86	0.37044	2.86E-15	9450
ZNF750	0.370419	2.87E-15	79755

ZBTB1	0.37038	2.89E-15	22890
ATF7	0.370245	2.96E-15	11016
ST3GAL2	0.370184	2.99E-15	6483
RNF222	0.370075	3.06E-15	643904
CEP162	0.369934	3.14E-15	22832
ECM1	0.369815	3.20E-15	1893
CREB5	0.369789	3.22E-15	9586
ARHGEF3	0.369723	3.26E-15	50650
RNF169	0.369616	3.32E-15	254225
RAP2B	0.369568	3.35E-15	5912
BNIP3L	0.369504	3.39E-15	665
MEGF6	0.369492	3.40E-15	1953
STXBP4	0.369473	3.41E-15	252983
ZCCHC24	0.369466	3.42E-15	219654
DUSP10	0.369447	3.43E-15	11221
SH2B3	0.369423	3.44E-15	10019
PLK3	0.369286	3.53E-15	1263
MPP4	0.368803	3.86E-15	58538
ZC3H10	0.368714	3.92E-15	84872
AMOTL1	0.368666	3.96E-15	154810
OTUD4	0.368572	4.03E-15	54726
BBIP1	0.368501	4.08E-15	92482
EXOC3L2	0.368484	4.09E-15	90332
RASA1	0.368309	4.22E-15	5921
NOVA2	0.368228	4.29E-15	4858
ISLR2	0.368213	4.30E-15	57611
GPR182	0.368192	4.32E-15	11318
LRRC70	0.368138	4.36E-15	100130733
SPRY1	0.367681	4.74E-15	10252
GPATCH2L	0.367619	4.79E-15	55668
FAM155A	0.367446	4.94E-15	728215
UBE2W	0.367292	5.09E-15	55284
ELL2	0.367206	5.17E-15	22936
PIK3AP1	0.367024	5.34E-15	118788
NEK1	0.366917	5.44E-15	4750
LRRC38	0.366863	5.50E-15	126755
LAMC2	0.366768	5.59E-15	3918
LEPROT	0.366742	5.62E-15	54741
ALCAM	0.366641	5.73E-15	214
OXTR	0.366628	5.74E-15	5021
MFSD1	0.366465	5.91E-15	64747
ACSL4	0.36629	6.10E-15	2182
GNB5	0.366267	6.13E-15	10681
AGO3	0.366116	6.30E-15	192669
PRDM6	0.366077	6.34E-15	93166
IRAK2	0.365944	6.50E-15	3656
PLEKHA3	0.365882	6.57E-15	65977
GABARAPL1	0.365862	6.60E-15	23710
LBP	0.365844	6.62E-15	3929
COL13A1	0.365807	6.66E-15	1305
COLGALT1	0.36576	6.72E-15	79709
FAM178A	0.365728	6.76E-15	NA
PCDH9	0.365605	6.91E-15	5101
HDGFRP3	0.365557	6.97E-15	NA
FKBP15	0.365532	7.00E-15	23307
IFIT5	0.36553	7.00E-15	24138
RIPK2	0.365165	7.48E-15	8767
AC037459.4	0.365151	7.50E-15	NA
TGFBR2	0.36512	7.54E-15	7048
SUFU	0.365091	7.58E-15	51684

PPAPDC3	0.364923	7.82E-15	NA
SNX31	0.364911	7.83E-15	169166
RC3H2	0.364888	7.87E-15	54542
C5orf51	0.364691	8.15E-15	285636
UBXN7	0.364587	8.31E-15	26043
VAMP4	0.364564	8.34E-15	8674
FAM219A	0.364531	8.39E-15	203259
MAP3K7	0.364476	8.47E-15	6885
PYGO1	0.364432	8.54E-15	26108
BBS10	0.364421	8.56E-15	79738
ZNF484	0.364344	8.68E-15	83744
TRAM2	0.364233	8.85E-15	9697
RAB13	0.364097	9.07E-15	5872
COL6A6	0.364039	9.17E-15	131873
CLTCL1	0.363998	9.24E-15	8218
SP110	0.363958	9.30E-15	3431
TMEM30A	0.363945	9.33E-15	55754
PREX1	0.363824	9.53E-15	57580
WNK1	0.363505	1.01E-14	65125
C2orf74	0.363483	1.01E-14	339804
ZFHX3	0.363438	1.02E-14	463
NR3C1	0.363359	1.04E-14	2908
ZNF382	0.363303	1.05E-14	84911
SGMS2	0.363294	1.05E-14	166929
C21orf91	0.363241	1.06E-14	54149
C15orf53	0.363181	1.07E-14	NA
JAM3	0.363052	1.10E-14	83700
PLAUR	0.362948	1.12E-14	5329
STX7	0.362925	1.12E-14	8417
MMP21	0.362887	1.13E-14	118856
RAB12	0.362861	1.13E-14	201475
MYADM	0.362857	1.13E-14	91663
AP1S2	0.362777	1.15E-14	8905
LATS1	0.362759	1.15E-14	9113
PURA	0.362736	1.16E-14	5813
TSPYL5	0.362666	1.17E-14	85453
ZNF626	0.362648	1.18E-14	199777
CNN2	0.362554	1.20E-14	1265
STK3	0.362538	1.20E-14	6788
GGCX	0.36253	1.20E-14	2677
KANSL1L	0.362492	1.21E-14	151050
CXorf21	0.362343	1.24E-14	NA
DISC1	0.362323	1.25E-14	27185
MSANTD4	0.36226	1.26E-14	84437
ETV1	0.362251	1.26E-14	2115
PRDM2	0.362136	1.29E-14	7799
IQSEC1	0.362096	1.30E-14	9922
TRIM5	0.362061	1.31E-14	85363
FAT4	0.362024	1.32E-14	79633
RNF115	0.36198	1.33E-14	27246
PFKFB3	0.361964	1.33E-14	5209
PITPNC1	0.361776	1.38E-14	26207
TSPYL4	0.361721	1.39E-14	23270
TTC7B	0.361683	1.40E-14	145567
ARIH1	0.361668	1.40E-14	25820
NBPF19	0.361557	1.43E-14	101060226
NCKAP1L	0.361481	1.45E-14	3071
NBPF26	0.361452	1.46E-14	101060684
ANXA8	0.361338	1.49E-14	653145
GIMAP4	0.361261	1.51E-14	55303

MAP3K8	0.36106	1.56E-14	1326
ZNF322	0.360922	1.60E-14	79692
PHF14	0.360874	1.62E-14	9678
ELFN1	0.360871	1.62E-14	392617
TMEM263	0.36087	1.62E-14	90488
CLDN11	0.360822	1.63E-14	5010
RASGRP1	0.360791	1.64E-14	10125
KIAA1586	0.360767	1.65E-14	57691
PRKD3	0.360705	1.67E-14	23683
11-9月	0.360691	1.67E-14	NA
RAB29	0.360659	1.68E-14	8934
TRIO	0.360542	1.72E-14	7204
MXRA7	0.36046	1.74E-14	439921
MCAM	0.360442	1.75E-14	4162
PTPN22	0.360373	1.77E-14	26191
DHX36	0.36037	1.77E-14	170506
OTULIN	0.360368	1.77E-14	90268
TNFAIP3	0.360288	1.79E-14	7128
PALLD	0.360274	1.80E-14	23022
RUFY3	0.360133	1.84E-14	22902
RHCG	0.36004	1.88E-14	51458
FAM83A	0.360013	1.88E-14	84985
SLC12A8	0.35997	1.90E-14	84561
CDC42EP3	0.359825	1.95E-14	10602
JAK3	0.359802	1.96E-14	3718
UNC5B	0.359785	1.96E-14	219699
CDC73	0.359761	1.97E-14	79577
SCIMP	0.359698	1.99E-14	388325
PCDHGC3	0.359648	2.01E-14	5098
MYOZ2	0.359554	2.04E-14	51778
RAMP3	0.359537	2.05E-14	10268
LEPR	0.359371	2.11E-14	3953
DLEU7	0.359371	2.11E-14	220107
TNFSF18	0.359341	2.12E-14	8995
ZFP90	0.359304	2.14E-14	146198
PELO	0.359296	2.14E-14	53918
SYNC	0.359256	2.15E-14	81493
MYCBP2	0.359204	2.18E-14	23077
PCMTD1	0.359154	2.19E-14	115294
ROBO1	0.359104	2.21E-14	6091
CLASP1	0.35907	2.23E-14	23332
SP100	0.358988	2.26E-14	6672
ARL6	0.358972	2.27E-14	84100
IFT81	0.358917	2.29E-14	28981
PLEKHA1	0.358765	2.35E-14	59338
GSAP	0.358695	2.38E-14	54103
C10orf131	0.358668	2.39E-14	NA
KIAA1024	0.35852	2.45E-14	NA
AJAP1	0.35851	2.46E-14	55966
MYBPH	0.358506	2.46E-14	4608
TNN	0.35848	2.47E-14	63923
STK32B	0.358371	2.52E-14	55351
SERPINB2	0.358262	2.57E-14	5055
C2CD2	0.35814	2.63E-14	25966
CLEC2B	0.358123	2.63E-14	9976
ASXL2	0.358006	2.69E-14	55252
STEAP4	0.357743	2.82E-14	79689
SMURF2	0.357595	2.89E-14	64750
UBE2D1	0.357548	2.91E-14	7321
MDGA1	0.357377	3.00E-14	266727

TNFAIP2	0.357365	3.01E-14	7127
PSTPIP1	0.357354	3.02E-14	9051
KIAA1551	0.357349	3.02E-14	NA
GPR75-ASB3	0.357153	3.12E-14	100302652
OAS2	0.357109	3.15E-14	4939
EVI5	0.357102	3.15E-14	7813
SMCO3	0.357081	3.16E-14	440087
ZFAND5	0.357053	3.18E-14	7763
GPR183	0.357018	3.20E-14	1880
CFLAR	0.357001	3.21E-14	8837
ZNF154	0.356948	3.24E-14	7710
GLIS3	0.356836	3.30E-14	169792
TTYH2	0.356831	3.31E-14	94015
CDC42SE1	0.356819	3.31E-14	56882
CTSO	0.356746	3.36E-14	1519
MPRIP	0.356719	3.37E-14	23164
CLSTN3	0.356685	3.39E-14	9746
RAB11FIP2	0.356599	3.44E-14	22841
OSBPL9	0.356548	3.47E-14	114883
LGI2	0.356406	3.56E-14	55203
SV2B	0.35638	3.58E-14	9899
KIAA0825	0.356379	3.58E-14	285600
NME7	0.356301	3.63E-14	29922
FZD8	0.356277	3.64E-14	8325
ANKRD42	0.356227	3.68E-14	338699
CTLA4	0.356154	3.72E-14	1493
FABP4	0.355879	3.91E-14	2167
USHBP1	0.355789	3.97E-14	83878
GPR133	0.355739	4.00E-14	NA
ZKSCAN8	0.355659	4.06E-14	7745
ZNF676	0.355659	4.06E-14	163223
BLOC1S6	0.355562	4.13E-14	26258
IGDCC4	0.355168	4.42E-14	57722
C17orf107	0.355082	4.49E-14	100130311
MEF2A	0.355037	4.53E-14	4205
APOC2	0.355031	4.53E-14	344
CDR2L	0.354936	4.61E-14	30850
ERCC4	0.354852	4.67E-14	2072
RANBP9	0.354794	4.72E-14	10048
MALT1	0.354756	4.75E-14	10892
ANGPTL4	0.354634	4.86E-14	51129
AFAP1	0.354417	5.04E-14	60312
CXCL9	0.354412	5.05E-14	4283
TTC28	0.354342	5.11E-14	23331
SMARCA1	0.35431	5.14E-14	6594
TAF3	0.3543	5.15E-14	83860
FBLN1	0.354194	5.24E-14	2192
CYP19A1	0.354141	5.29E-14	1588
NEK7	0.353986	5.44E-14	140609
CDC27	0.35397	5.45E-14	996
IGFL1	0.353929	5.49E-14	374918
KLHL20	0.353856	5.56E-14	27252
SLC16A4	0.353698	5.71E-14	9122
AKAP2	0.353696	5.72E-14	NA
EPS15	0.353595	5.82E-14	2060
CLNK	0.353572	5.84E-14	116449
ELF2	0.353524	5.89E-14	1998
GRHL1	0.353178	6.25E-14	29841
KCNT2	0.353152	6.28E-14	343450
STX6	0.353128	6.31E-14	10228

SEC24D	0.353121	6.32E-14	9871
YWHAG	0.35298	6.47E-14	7532
COL7A1	0.352759	6.72E-14	1294
KLF12	0.352759	6.72E-14	11278
WDR26	0.352755	6.73E-14	80232
FADS1	0.352719	6.77E-14	3992
CEP19	0.352694	6.80E-14	84984
STAU2	0.352658	6.84E-14	27067
ITGA2	0.352555	6.97E-14	3673
TROVE2	0.352553	6.97E-14	NA
KLF11	0.352523	7.01E-14	8462
KLHL7	0.352504	7.03E-14	55975
CEP85L	0.352453	7.09E-14	387119
RELT	0.352385	7.17E-14	84957
DHH	0.352243	7.35E-14	50846
KMT2A	0.352238	7.36E-14	4297
INPP4A	0.351935	7.76E-14	3631
CLIC2	0.351931	7.76E-14	1193
ECEL1	0.351896	7.81E-14	9427
YEATS2	0.351839	7.89E-14	55689
FBXL2	0.351817	7.91E-14	25827
TMEM163	0.351714	8.06E-14	81615
KIAA1210	0.351678	8.11E-14	57481
MDFI	0.351574	8.25E-14	4188
CYGB	0.351499	8.36E-14	114757
RAET1G	0.351487	8.38E-14	353091
APBB1IP	0.35143	8.46E-14	54518
CHPF	0.351311	8.64E-14	79586
TEX14	0.351287	8.67E-14	56155
SOX17	0.351131	8.91E-14	64321
CHST10	0.351047	9.04E-14	9486
FAM212A	0.351037	9.05E-14	NA
KRT80	0.351036	9.06E-14	144501
KIF3A	0.350809	9.42E-14	11127
RSF1	0.350784	9.46E-14	51773
ZDHHC17	0.350703	9.59E-14	23390
DOCK2	0.350637	9.70E-14	1794
PRELP	0.350523	9.89E-14	5549
KRT17	0.350481	9.96E-14	3872
PCDHGA3	0.350476	9.97E-14	56112
CBFB	0.350367	1.02E-13	865
LURAP1	0.350161	1.05E-13	541468
OAZ2	0.350114	1.06E-13	4947
CLCF1	0.350048	1.07E-13	23529
GTF2I	0.350041	1.07E-13	2969
MAP3K1	0.350023	1.08E-13	4214
ATG7	0.349885	1.10E-13	10533
FMNL2	0.349872	1.11E-13	114793
CCDC144A	0.349869	1.11E-13	9720
SUSD5	0.349859	1.11E-13	26032
IGIP	0.349789	1.12E-13	492311
PCED1B	0.349653	1.15E-13	91523
CDC37L1	0.349628	1.15E-13	55664
ZFYVE26	0.349624	1.15E-13	23503
NCCRP1	0.349561	1.17E-13	342897
MLC1	0.349406	1.20E-13	23209
SAMD14	0.349392	1.20E-13	201191
REV3L	0.34935	1.21E-13	5980
ALS2	0.349322	1.22E-13	57679
RAB42	0.34926	1.23E-13	115273

DENND6A	0.349218	1.24E-13	201627
ICOS	0.349194	1.24E-13	29851
FBXO30	0.349157	1.25E-13	84085
TUBB6	0.349057	1.27E-13	84617
YAP1	0.349027	1.28E-13	10413
DNASE2	0.348887	1.31E-13	1777
GREB1L	0.348854	1.32E-13	80000
MYH8	0.348744	1.34E-13	4626
ANKIB1	0.348744	1.34E-13	54467
ITPR1	0.348607	1.37E-13	3708
HSPA13	0.348483	1.40E-13	6782
CLEC1B	0.348481	1.40E-13	51266
COPS8	0.348459	1.41E-13	10920
FRS2	0.348439	1.41E-13	10818
INSIG2	0.348404	1.42E-13	51141
RPAP2	0.34837	1.43E-13	79871
NXPE3	0.348345	1.44E-13	91775
CASS4	0.348328	1.44E-13	57091
BMI1	0.348318	1.44E-13	648
PLCB1	0.34825	1.46E-13	23236
CAMSAP1	0.348191	1.47E-13	157922
FZD2	0.347962	1.53E-13	2535
TYW5	0.347909	1.55E-13	129450
B3GALTL	0.347856	1.56E-13	NA
SLN	0.347778	1.58E-13	6588
NAP1L3	0.347762	1.59E-13	4675
PIKFYVE	0.347729	1.59E-13	200576
CHORDC1	0.347724	1.60E-13	26973
RNGTT	0.347711	1.60E-13	8732
C4orf3	0.347637	1.62E-13	401152
AHDC1	0.347592	1.63E-13	27245
SHC1	0.347562	1.64E-13	6464
HIAT1	0.347472	1.67E-13	NA
P4HA1	0.347423	1.68E-13	5033
ZNF292	0.347381	1.69E-13	23036
RAB33B	0.347214	1.74E-13	83452
DPYD	0.347213	1.74E-13	1806
DOCK11	0.347092	1.78E-13	139818
SMIM13	0.347063	1.79E-13	221710
STEAP1B	0.347026	1.80E-13	256227
GPATCH2	0.347024	1.80E-13	55105
CYS1	0.346989	1.81E-13	192668
MS4A4A	0.346944	1.82E-13	51338
C12orf79	0.346909	1.83E-13	NA
ITSN1	0.346857	1.85E-13	6453
SNAPC1	0.346717	1.89E-13	6617
OLFM2	0.34665	1.92E-13	93145
CCDC82	0.346647	1.92E-13	79780
EHBP1	0.346568	1.94E-13	23301
TSPYL2	0.346557	1.95E-13	64061
HACE1	0.346552	1.95E-13	57531
RFX3	0.346516	1.96E-13	5991
WSB1	0.346472	1.97E-13	26118
METTL21B	0.346404	2.00E-13	NA
AASS	0.346196	2.07E-13	10157
SMURF1	0.345949	2.16E-13	57154
UBE2Q2	0.345945	2.16E-13	92912
KCTD7	0.34593	2.16E-13	154881
ATG12	0.345884	2.18E-13	9140
SOS1	0.345835	2.20E-13	6654

DFNA5	0.345834	2.20E-13	NA
DPEP2	0.345759	2.23E-13	64174
SEC22C	0.345523	2.32E-13	9117
PLVAP	0.345473	2.34E-13	83483
HSD17B6	0.345437	2.35E-13	8630
DDX58	0.345435	2.35E-13	23586
PCDHB7	0.345381	2.37E-13	56129
BMPR1B	0.345345	2.39E-13	658
ZNF449	0.345261	2.42E-13	203523
C16orf72	0.345209	2.44E-13	29035
C16orf71	0.345186	2.45E-13	146562
UGCG	0.345073	2.50E-13	7357
TMEM109	0.345033	2.52E-13	79073
BLOC1S2	0.345024	2.52E-13	282991
FBN2	0.344825	2.61E-13	2201
NBN	0.344816	2.61E-13	4683
WNT9B	0.344781	2.63E-13	7484
TK2	0.344772	2.63E-13	7084
TOR1AIP1	0.344767	2.63E-13	26092
DNAJB6	0.34473	2.65E-13	10049
PITPNB	0.344697	2.66E-13	23760
ATXN3	0.344688	2.67E-13	4287
TNRC6A	0.34449	2.76E-13	27327
C5orf24	0.344442	2.78E-13	134553
KIDINS220	0.344357	2.82E-13	57498
A2M	0.344337	2.83E-13	2
KIF17	0.344285	2.85E-13	57576
HELZ	0.344241	2.88E-13	9931
LILRA1	0.344148	2.92E-13	11024
C1orf101	0.344135	2.93E-13	NA
DNAJC18	0.344074	2.96E-13	202052
STK10	0.343929	3.03E-13	6793
NFKBIE	0.3439	3.04E-13	4794
PPP3CA	0.343874	3.06E-13	5530
SCAF11	0.343788	3.10E-13	9169
TMED7-TICAM2	0.343745	3.12E-13	100302736
WNT7A	0.343639	3.18E-13	7476
APOL3	0.343588	3.21E-13	80833
MYH9	0.343512	3.25E-13	4627
C4B	0.343397	3.31E-13	721
ST3GAL1	0.343307	3.36E-13	6482
CECR1	0.343262	3.39E-13	NA
TREML1	0.343074	3.50E-13	340205
MKL2	0.342846	3.63E-13	NA
LPXN	0.342718	3.71E-13	9404
ZNF699	0.342689	3.73E-13	374879
RGS19	0.342674	3.74E-13	10287
PRCD	0.34265	3.75E-13	768206
CSF1R	0.342595	3.79E-13	1436
ZBTB11	0.342527	3.83E-13	27107
MX2	0.3425	3.85E-13	4600
CEP120	0.342468	3.87E-13	153241
RNF19A	0.342436	3.89E-13	25897
NFIC	0.34229	3.99E-13	4782
GPATCH8	0.342275	4.00E-13	23131
ARL8B	0.34222	4.03E-13	55207
MYO1G	0.342201	4.05E-13	64005
SCARF1	0.341974	4.20E-13	8578
MEX3C	0.34191	4.25E-13	51320
HCFC1R1	0.341879	4.27E-13	54985

CEP97	0.341795	4.33E-13	79598
PARVA	0.341694	4.40E-13	55742
NEMF	0.341682	4.41E-13	9147
SFT2D2	0.341568	4.50E-13	375035
TGM2	0.341523	4.53E-13	7052
WDR19	0.341422	4.61E-13	57728
SHISA2	0.341382	4.64E-13	387914
AIF1	0.341372	4.64E-13	199
GLT8D1	0.341336	4.67E-13	55830
TMEM40	0.341321	4.68E-13	55287
CRTC3	0.341308	4.69E-13	64784
ETAA1	0.341249	4.74E-13	54465
NPAT	0.341241	4.75E-13	4863
NOD1	0.341151	4.82E-13	10392
ZBTB4	0.341083	4.87E-13	57659
GNPDA2	0.341058	4.89E-13	132789
MAST4	0.341015	4.93E-13	375449
M6PR	0.34098	4.96E-13	4074
ATF2	0.340969	4.97E-13	1386
LIMK1	0.340787	5.12E-13	3984
PLXNA1	0.340467	5.40E-13	5361
FBXO11	0.340435	5.43E-13	80204
LRRC8A	0.340413	5.45E-13	56262
TMEM52B	0.340361	5.49E-13	120939
P3H4	0.340154	5.68E-13	10609
FAM3C	0.340038	5.79E-13	10447
BPIFB4	0.339992	5.84E-13	149954
HCAR2	0.339901	5.93E-13	338442
USP15	0.339884	5.94E-13	9958
OSGIN2	0.339867	5.96E-13	734
TRAPPC6B	0.339854	5.97E-13	122553
MKX	0.339678	6.15E-13	283078
PPL	0.339666	6.16E-13	5493
SELP	0.339538	6.29E-13	6403
TRIM59	0.339392	6.45E-13	286827
HLA-DQA1	0.339331	6.51E-13	3117
KIAA0355	0.339205	6.65E-13	NA
OSBPL3	0.339101	6.76E-13	26031
MEMO1	0.339002	6.88E-13	7795
SLC25A30	0.338993	6.89E-13	253512
ARPP19	0.338971	6.91E-13	10776
PCDHGB7	0.338915	6.98E-13	56099
VRK2	0.33884	7.06E-13	7444
MOSPD1	0.338839	7.06E-13	56180
NAIP	0.338814	7.09E-13	4671
NBPF15	0.338787	7.12E-13	284565
MOSPD2	0.338774	7.14E-13	158747
RNF13	0.338724	7.20E-13	11342
ZNF286A	0.33859	7.36E-13	57335
CMKLR1	0.338491	7.48E-13	1240
FOXN2	0.338483	7.49E-13	3344
PTEN	0.338352	7.65E-13	5728
XXYLT1	0.338213	7.83E-13	152002
FAM220A	0.338199	7.85E-13	84792
AGO4	0.338092	7.99E-13	192670
CC2D2A	0.338081	8.00E-13	57545
PCDHB10	0.338064	8.02E-13	56126
KPNA1	0.338054	8.04E-13	3836
CMTM1	0.337998	8.11E-13	113540
NOS3	0.337956	8.17E-13	4846

ERGIC2	0.337947	8.18E-13	51290
FOXC1	0.337915	8.22E-13	2296
CCPG1	0.337875	8.28E-13	9236
LAMP2	0.337861	8.30E-13	3920
NFATC1	0.33774	8.46E-13	4772
JAZF1	0.337725	8.48E-13	221895
TNFSF12	0.337695	8.52E-13	8742
HPS3	0.337624	8.62E-13	84343
MANBA	0.33757	8.70E-13	4126
DAB2	0.337543	8.74E-13	1601
EID3	0.337529	8.76E-13	493861
CD99L2	0.337492	8.81E-13	83692
TMEM39A	0.337438	8.89E-13	55254
CD4	0.337393	8.96E-13	920
RASIP1	0.337275	9.13E-13	54922
MS4A4E	0.33721	9.23E-13	643680
CACUL1	0.337185	9.27E-13	143384
XAF1	0.337183	9.27E-13	54739
GYS2	0.337179	9.28E-13	2998
SLC12A1	0.337169	9.29E-13	6557
NBPF8	0.337148	9.32E-13	728841
USP31	0.337133	9.35E-13	57478
CCDC89	0.337084	9.42E-13	220388
SMCO2	0.337037	9.50E-13	341346
ZIK1	0.336778	9.91E-13	284307
MAN1A2	0.336768	9.92E-13	10905
BOD1L1	0.336758	9.94E-13	259282
SPRED1	0.336699	1.00E-12	161742
ZNF385A	0.336668	1.01E-12	25946
CFAP97	0.336656	1.01E-12	57587
TMEM200C	0.336635	1.01E-12	645369
ZNF280D	0.336538	1.03E-12	54816
PGBD3	0.336521	1.03E-12	267004
CLOCK	0.336519	1.03E-12	9575
VASH2	0.336475	1.04E-12	79805
SOCS4	0.336458	1.04E-12	122809
LAT2	0.336298	1.07E-12	7462
DENND4A	0.336239	1.08E-12	10260
TMEM136	0.336051	1.12E-12	NA
PHIP	0.336	1.12E-12	55023
QSER1	0.335989	1.13E-12	79832
MAP3K2	0.335971	1.13E-12	10746
C9orf47	0.335948	1.13E-12	286223
PARP14	0.335905	1.14E-12	54625
LY6D	0.335769	1.17E-12	8581
GRIA3	0.335542	1.21E-12	2892
FOXO1	0.335363	1.25E-12	2308
VEZF1	0.335335	1.25E-12	7716
CORO1C	0.33531	1.26E-12	23603
IBSP	0.335222	1.28E-12	3381
SRSF12	0.335152	1.29E-12	135295
DOCK9	0.335149	1.29E-12	23348
C12orf4	0.334961	1.33E-12	57102
AJUBA	0.334955	1.33E-12	84962
ZNF430	0.334826	1.36E-12	80264
OSM	0.33477	1.37E-12	5008
ERCC6	0.334754	1.38E-12	2074
ATRX	0.334679	1.39E-12	546
CD14	0.334599	1.41E-12	929
FHL5	0.334585	1.42E-12	9457

SLC9B2	0.334451	1.45E-12	133308
ARHGAP30	0.33428	1.49E-12	257106
CERS3	0.334173	1.51E-12	204219
C4orf32	0.334163	1.52E-12	NA
AP1M1	0.33414	1.52E-12	8907
EID1	0.334059	1.54E-12	23741
CASP4	0.33395	1.57E-12	837
MCF2	0.333944	1.57E-12	4168
C8orf58	0.333902	1.58E-12	541565
RNF225	0.333876	1.59E-12	646862
FAM91A1	0.333864	1.59E-12	157769
ZNF618	0.333848	1.60E-12	114991
MTR	0.333771	1.62E-12	4548
GLS	0.333732	1.63E-12	2744
CHD9	0.333676	1.64E-12	80205
SRPK2	0.333673	1.64E-12	6733
ZMYM4	0.333673	1.64E-12	9202
TNRC6C	0.333537	1.68E-12	57690
HSPB2	0.333409	1.71E-12	3316
MMP12	0.333382	1.72E-12	4321
MEFV	0.333367	1.72E-12	4210
USP32	0.333357	1.73E-12	84669
CCDC6	0.333318	1.74E-12	8030
PPP1R12A	0.333184	1.78E-12	4659
DUSP14	0.333147	1.79E-12	11072
SPATA9	0.333134	1.79E-12	83890
MECP2	0.333038	1.82E-12	4204
CPEB4	0.333026	1.82E-12	80315
SGCE	0.332963	1.84E-12	8910
ADAR	0.332961	1.84E-12	103
CMTM6	0.332882	1.87E-12	54918
CLASP2	0.332872	1.87E-12	23122
PHACTR3	0.332821	1.88E-12	116154
RICTOR	0.332774	1.90E-12	253260
SMIM10	0.332768	1.90E-12	644538
FBXW11	0.332767	1.90E-12	23291
POLI	0.332734	1.91E-12	11201
VCPIP1	0.33273	1.91E-12	80124
SAV1	0.332589	1.96E-12	60485
IL27RA	0.332373	2.02E-12	9466
TULP4	0.33234	2.04E-12	56995
CCP110	0.33228	2.05E-12	9738
TPP1	0.332225	2.07E-12	1200
HCFC2	0.332202	2.08E-12	29915
CFI	0.332162	2.09E-12	3426
IMPAD1	0.332148	2.10E-12	NA
ARHGAP15	0.332013	2.15E-12	55843
CTNS	0.331985	2.15E-12	1497
ZCCHC11	0.3319	2.18E-12	NA
PRL	0.331899	2.18E-12	5617
ZNHIT6	0.331878	2.19E-12	54680
STON1-GTF2A1L	0.331865	2.20E-12	286749
PRNP	0.331799	2.22E-12	5621
FAM171B	0.331778	2.23E-12	165215
CBLN3	0.331747	2.24E-12	643866
TNFSF11	0.331696	2.26E-12	8600
BTN2A2	0.331599	2.29E-12	10385
FKBP9	0.331555	2.31E-12	11328
LDOC1	0.331494	2.33E-12	23641
HERC4	0.331345	2.39E-12	26091

GTDC1	0.331336	2.39E-12	79712
NAB1	0.331273	2.42E-12	4664
TP63	0.331267	2.42E-12	8626
CGGBP1	0.331244	2.43E-12	8545
UHRF2	0.331237	2.43E-12	115426
HCLS1	0.331137	2.47E-12	3059
ZNF385D	0.331107	2.48E-12	79750
TMEM245	0.331035	2.51E-12	23731
CIDEA	0.330998	2.52E-12	1149
RPS6KB1	0.330839	2.59E-12	6198
APOL4	0.330771	2.62E-12	80832
GPR180	0.33072	2.64E-12	160897
SNURF	0.33067	2.66E-12	8926
AZI2	0.330655	2.67E-12	64343
THADA	0.33063	2.68E-12	63892
GPAM	0.330619	2.68E-12	57678
FAT2	0.330599	2.69E-12	2196
ZNF264	0.330542	2.72E-12	9422
NCOA3	0.330492	2.74E-12	8202
ESCO1	0.330426	2.77E-12	114799
TCEAL4	0.330415	2.77E-12	79921
S100A8	0.330324	2.81E-12	6279
GBGT1	0.330304	2.82E-12	26301
ALKBH8	0.330283	2.83E-12	91801
KAT6A	0.330255	2.84E-12	7994
SLC16A7	0.330223	2.86E-12	9194
CREB3L2	0.330214	2.86E-12	64764
RPGRIP1L	0.330206	2.87E-12	23322
BAZ2B	0.330004	2.96E-12	29994
SP4	0.329964	2.98E-12	6671
WNT5A	0.32994	2.99E-12	7474
DPY19L1	0.329929	2.99E-12	23333
UBLCP1	0.329775	3.07E-12	134510
MTMR2	0.329774	3.07E-12	8898
ZFR	0.329722	3.10E-12	51663
ZFYVE16	0.329636	3.14E-12	9765
THAP1	0.329508	3.20E-12	55145
ANKAR	0.329503	3.20E-12	150709
RASA3	0.329491	3.21E-12	22821
MORF4L1	0.329429	3.24E-12	10933
CSF2RA	0.329381	3.27E-12	1438
ADAM9	0.329313	3.30E-12	8754
SOX5	0.329145	3.39E-12	6660
TMEM106A	0.329142	3.39E-12	113277
CYB5R1	0.329042	3.45E-12	51706
KLHL24	0.329024	3.46E-12	54800
CR1L	0.329024	3.46E-12	1379
SYT14	0.328987	3.48E-12	255928
IFI44	0.328672	3.66E-12	10561
FRA10AC1	0.328621	3.69E-12	118924
VSIG4	0.328603	3.70E-12	11326
PHC1	0.3285	3.76E-12	1911
PRICKLE2	0.328439	3.80E-12	166336
KDM5A	0.328432	3.80E-12	5927
PARD6G	0.328408	3.81E-12	84552
ACTR3	0.328407	3.82E-12	10096
ARID1B	0.328286	3.89E-12	57492
TNR	0.328154	3.97E-12	7143
FKBP10	0.328114	4.00E-12	60681
RB1CC1	0.328062	4.03E-12	9821

SLC9C1	0.328041	4.04E-12	285335
CD68	0.32794	4.11E-12	968
ZNF483	0.327913	4.13E-12	158399
SPTY2D1	0.327846	4.17E-12	144108
FAM69A	0.327825	4.18E-12	NA
KIAA2026	0.327773	4.22E-12	158358
LRRC58	0.327746	4.24E-12	116064
ZNF570	0.327737	4.24E-12	148268
SCLT1	0.327724	4.25E-12	132320
SMG1	0.327646	4.30E-12	23049
GOLT1B	0.327638	4.31E-12	51026
LINGO2	0.327574	4.35E-12	158038
NF1	0.327572	4.35E-12	4763
HSPBAP1	0.327507	4.40E-12	79663
MEGF9	0.327461	4.43E-12	1955
HIVEP3	0.327437	4.45E-12	59269
DCBLD2	0.327408	4.47E-12	131566
SEC63	0.327368	4.50E-12	11231
NUFIP2	0.327345	4.51E-12	57532
TNFAIP8L2	0.327317	4.53E-12	79626
ZRANB1	0.327245	4.59E-12	54764
CPQ	0.3271	4.69E-12	10404
L3MBTL3	0.327085	4.70E-12	84456
C1RL	0.327084	4.70E-12	51279
ZNF143	0.32705	4.73E-12	7702
KCNK12	0.327001	4.77E-12	56660
SLC12A6	0.326957	4.80E-12	9990
SENP1	0.326912	4.83E-12	29843
DTWD1	0.326829	4.90E-12	56986
SNX16	0.326799	4.92E-12	64089
IGF1R	0.326702	5.00E-12	3480
SLCO1C1	0.326669	5.02E-12	53919
NOG	0.326603	5.07E-12	9241
PIK3C2A	0.326503	5.16E-12	5286
PHC3	0.326461	5.19E-12	80012
PRG4	0.326452	5.20E-12	10216
ENDOU	0.32639	5.25E-12	8909
GRM6	0.326361	5.27E-12	2916
CRLF2	0.326284	5.34E-12	64109
FAM208A	0.326211	5.40E-12	NA
ATP6AP1L	0.326206	5.40E-12	92270
ZNF781	0.326201	5.41E-12	163115
EPG5	0.326085	5.51E-12	57724
TBX19	0.326064	5.52E-12	9095
SYNCRIP	0.326026	5.56E-12	10492
NCKAP1	0.326023	5.56E-12	10787
APOBEC3H	0.326008	5.57E-12	164668
GBE1	0.325949	5.62E-12	2632
VPS41	0.325831	5.73E-12	27072
PLIN1	0.325792	5.77E-12	5346
SAMD3	0.325768	5.79E-12	154075
ZNF880	0.325758	5.80E-12	400713
FERMT2	0.325735	5.82E-12	10979
SLC7A6OS	0.325631	5.91E-12	84138
SOGA1	0.325544	5.99E-12	140710
MID2	0.325454	6.08E-12	11043
ZNF569	0.325452	6.08E-12	148266
CGB7	0.325357	6.17E-12	94027
IKZF5	0.325297	6.23E-12	64376
CD72	0.325295	6.23E-12	971

RGS20	0.325207	6.32E-12	8601
IL7R	0.325145	6.38E-12	3575
WAPAL	0.325089	6.44E-12	NA
STAT1	0.325083	6.44E-12	6772
TAF2	0.325058	6.47E-12	6873
MAP7D3	0.325012	6.52E-12	79649
CD47	0.324979	6.55E-12	961
STRADA	0.324948	6.58E-12	92335
ZNF148	0.32485	6.68E-12	7707
SNN	0.324811	6.72E-12	8303
GPSM3	0.324752	6.79E-12	63940
BTN3A3	0.324672	6.87E-12	10384
DR1	0.324647	6.90E-12	1810
AFF4	0.324499	7.06E-12	27125
MLLT11	0.324429	7.14E-12	10962
LIPA	0.324396	7.18E-12	3988
IL10RA	0.324327	7.25E-12	3587
ZNFX1	0.324287	7.30E-12	57169
EMR3	0.324286	7.30E-12	NA
SLC38A7	0.324281	7.30E-12	55238
DST	0.323974	7.66E-12	667
NR1D2	0.323965	7.67E-12	9975
SRGAP1	0.323885	7.77E-12	57522
RLF	0.323842	7.82E-12	6018
LGALS12	0.323827	7.84E-12	85329
ZNF546	0.323617	8.10E-12	339327
CLEC2D	0.323545	8.19E-12	29121
KIAA0196	0.323498	8.25E-12	NA
UTRN	0.323447	8.32E-12	7402
ZNF860	0.323233	8.60E-12	344787
PNMAL1	0.32322	8.62E-12	NA
C16orf87	0.323115	8.76E-12	388272
RAP2A	0.323028	8.88E-12	5911
USP47	0.322983	8.94E-12	55031
AHNAK2	0.322966	8.97E-12	113146
TUB	0.322774	9.24E-12	7275
RPS6KC1	0.322734	9.30E-12	26750
ADCY7	0.322714	9.33E-12	113
MAPK11	0.322661	9.40E-12	5600
DOK2	0.322612	9.47E-12	9046
RP5-1021I20.4	0.322609	9.48E-12	NA
HIVEP1	0.322574	9.53E-12	3096
ZNF624	0.322521	9.61E-12	57547
DBN1	0.322477	9.67E-12	1627
KCTD10	0.322462	9.70E-12	83892
MTSS1L	0.322418	9.76E-12	NA
LIPE	0.322341	9.88E-12	3991
ZNF555	0.322289	9.96E-12	148254
RFX7	0.322232	1.00E-11	64864
ARMCX1	0.322176	1.01E-11	51309
USP34	0.322051	1.03E-11	9736
PXK	0.322003	1.04E-11	54899
SHC4	0.322002	1.04E-11	399694
XRN1	0.321978	1.05E-11	54464
C7orf25	0.321969	1.05E-11	79020
CD59	0.321967	1.05E-11	966
TANK	0.321886	1.06E-11	10010
RP11-196G11.1	0.321773	1.08E-11	NA
STK17A	0.321764	1.08E-11	9263
SLC35G2	0.321635	1.10E-11	80723

ZNF70	0.321629	1.10E-11	7621
PDCL	0.321619	1.11E-11	5082
EEA1	0.321497	1.13E-11	8411
COLQ	0.321454	1.13E-11	8292
NEDD1	0.321444	1.14E-11	121441
PRDM1	0.321435	1.14E-11	639
RNF2	0.321272	1.17E-11	6045
PTPRC	0.321205	1.18E-11	5788
NRROS	0.321173	1.18E-11	375387
SMNDC1	0.321111	1.20E-11	10285
DCP2	0.321059	1.20E-11	167227
PTPN5	0.321058	1.21E-11	84867
RGL1	0.321013	1.21E-11	23179
ZNF354C	0.321007	1.21E-11	30832
ANO1	0.321002	1.22E-11	55107
GNAI3	0.32097	1.22E-11	2773
SDCBP	0.320939	1.23E-11	6386
PLEKHF1	0.320887	1.24E-11	79156
IFITM10	0.320844	1.25E-11	402778
PPP3CB	0.320816	1.25E-11	5532
DYNC1LI1	0.320803	1.25E-11	51143
CCDC30	0.320797	1.25E-11	728621
MTDH	0.320783	1.26E-11	92140
CD82	0.320776	1.26E-11	3732
WDR41	0.320747	1.26E-11	55255
PLEKHA8	0.320663	1.28E-11	84725
SLC30A6	0.320652	1.28E-11	55676
WDFY1	0.320619	1.29E-11	57590
CNTN1	0.320596	1.29E-11	1272
GLMP	0.320556	1.30E-11	112770
C9orf3	0.32054	1.31E-11	NA
DIP2B	0.320472	1.32E-11	57609
ESYT2	0.320454	1.32E-11	57488
CASP8AP2	0.320404	1.33E-11	9994
TRIM33	0.320395	1.33E-11	51592
NFATC3	0.320366	1.34E-11	4775
KIN	0.320168	1.38E-11	22944
RNF130	0.320056	1.41E-11	55819
STAM	0.319976	1.42E-11	8027
CKLF-CMTM1	0.319918	1.44E-11	100529251
HBP1	0.319916	1.44E-11	26959
XYLT1	0.319869	1.45E-11	64131
PNPLA8	0.319845	1.45E-11	50640
WDR44	0.319833	1.46E-11	54521
RNF122	0.319791	1.46E-11	79845
HS2ST1	0.319685	1.49E-11	9653
RBM12B	0.319681	1.49E-11	389677
ARID2	0.319663	1.49E-11	196528
CCL4L1	0.319629	1.50E-11	388372
PRSS27	0.319574	1.51E-11	83886
MAP4K3	0.319556	1.52E-11	8491
EPSTI1	0.319443	1.55E-11	94240
PTPDC1	0.319407	1.55E-11	138639
CDK2AP1	0.319401	1.56E-11	8099
TRAF1	0.319368	1.56E-11	7185
PHACTR1	0.319347	1.57E-11	221692
GTF2A1	0.319198	1.60E-11	2957
MED17	0.319191	1.61E-11	9440
USO1	0.319159	1.61E-11	8615
KDM2A	0.319123	1.62E-11	22992

RAB2B	0.319093	1.63E-11	84932
LYPD3	0.319024	1.65E-11	27076
TMEM55A	0.318976	1.66E-11	NA
ELMO2	0.318904	1.68E-11	63916
SRRD	0.318735	1.72E-11	402055
NHLRC2	0.318656	1.74E-11	374354
PDLIM4	0.318573	1.77E-11	8572
DPYS	0.318477	1.79E-11	1807
BPTF	0.318402	1.81E-11	2186
GRAP	0.318358	1.82E-11	10750
SVEP1	0.318256	1.85E-11	79987
MED13	0.31822	1.86E-11	9969
TUSC5	0.318182	1.87E-11	NA
C3orf58	0.318107	1.90E-11	NA
ZNF451	0.317971	1.94E-11	26036
DAAM1	0.317945	1.94E-11	23002
AZIN1	0.317935	1.95E-11	51582
TMEM67	0.317923	1.95E-11	91147
SMAD5	0.317909	1.95E-11	4090
PKP1	0.317893	1.96E-11	5317
SPAST	0.317827	1.98E-11	6683
CUL5	0.317816	1.98E-11	8065
F8	0.31779	1.99E-11	2157
LHFPL2	0.317742	2.00E-11	10184
CRIM1	0.317542	2.07E-11	51232
POGLUT1	0.317526	2.07E-11	56983
LILRA5	0.317478	2.09E-11	353514
GBP1	0.31747	2.09E-11	2633
WDR82	0.317461	2.09E-11	80335
LNPEP	0.31741	2.11E-11	4012
MORC3	0.317404	2.11E-11	23515
RNF216	0.317375	2.12E-11	54476
C4orf47	0.317301	2.14E-11	441054
TIGD7	0.317133	2.20E-11	91151
TNFAIP1	0.317118	2.20E-11	7126
ADAMTSL4	0.316974	2.25E-11	54507
STEAP1	0.316956	2.26E-11	26872
ATP6V1C1	0.316914	2.27E-11	528
ZYX	0.316827	2.30E-11	7791
NAALAD2	0.316807	2.31E-11	10003
SEC62	0.316753	2.33E-11	7095
C10orf128	0.316624	2.38E-11	NA
LARP6	0.316594	2.39E-11	55323
C2orf44	0.316488	2.43E-11	NA
EXTL3	0.316464	2.44E-11	2137
CLK4	0.316286	2.50E-11	57396
SLK	0.316225	2.52E-11	9748
IQCJ-SCHIP1	0.316213	2.53E-11	100505385
CTF1	0.316146	2.56E-11	1489
TLR5	0.316069	2.59E-11	7100
ABHD13	0.316016	2.61E-11	84945
SCAMP1	0.316005	2.61E-11	9522
C1orf100	0.31583	2.68E-11	200159
PCDHGC5	0.315817	2.69E-11	56097
SKIL	0.315803	2.69E-11	6498
AKAP13	0.315755	2.71E-11	11214
BTRC	0.315711	2.73E-11	8945
SLC39A10	0.315694	2.74E-11	57181
BZW1	0.315679	2.74E-11	9689
S100A9	0.315656	2.75E-11	6280

AKR1B1	0.315559	2.79E-11	231
TLL2	0.315449	2.84E-11	7093
ANKDD1A	0.315427	2.85E-11	348094
SYTL3	0.315413	2.86E-11	94120
KRT6B	0.315345	2.88E-11	3854
ADD2	0.31533	2.89E-11	119
WDR81	0.315289	2.91E-11	124997
ZNF37A	0.315236	2.93E-11	7587
KRT16	0.315209	2.94E-11	3868
RIF1	0.315192	2.95E-11	55183
SNRK	0.315192	2.95E-11	54861
NEFH	0.315162	2.97E-11	4744
ATAD1	0.315161	2.97E-11	84896
LRRC4C	0.315119	2.98E-11	57689
KIAA1429	0.314994	3.04E-11	NA
C15orf59	0.314911	3.08E-11	NA
ZNF185	0.314852	3.11E-11	7739
NFAT5	0.314851	3.11E-11	10725
8-9月	0.314836	3.11E-11	NA
HEXA	0.3148	3.13E-11	3073
ZBTB21	0.314788	3.14E-11	49854
GABPA	0.314694	3.18E-11	2551
DPY19L4	0.314681	3.19E-11	286148
FEZ1	0.314654	3.20E-11	9638
IL36G	0.314582	3.24E-11	56300
FOXJ2	0.314568	3.24E-11	55810
HERC5	0.314544	3.26E-11	51191
PCNP	0.314543	3.26E-11	57092
BCAP29	0.31449	3.28E-11	55973
SPATC1	0.314442	3.31E-11	375686
RNF213	0.314427	3.31E-11	57674
ACBD3	0.314382	3.34E-11	64746
ASCC1	0.314373	3.34E-11	51008
PCDHB12	0.314351	3.35E-11	56124
G3BP1	0.31434	3.36E-11	10146
TTC3	0.314294	3.38E-11	7267
FBXW2	0.314234	3.41E-11	26190
ZBTB33	0.314201	3.43E-11	10009
VEZT	0.314182	3.44E-11	55591
ATXN1L	0.314066	3.50E-11	342371
MBNL1	0.313908	3.58E-11	4154
GPRASP2	0.313787	3.65E-11	114928
RP11-38C17.1	0.313735	3.68E-11	NA
ACTN3	0.313697	3.70E-11	89
TSC22D3	0.313635	3.73E-11	1831
CCDC117	0.313558	3.78E-11	150275
IL36RN	0.313483	3.82E-11	26525
ICE1	0.31348	3.82E-11	23379
PPHLN1	0.313442	3.84E-11	51535
ZBTB2	0.313393	3.87E-11	57621
HELB	0.313354	3.89E-11	92797
SLC35E3	0.313295	3.93E-11	55508
GJB6	0.313282	3.94E-11	10804
ANKRD12	0.313181	4.00E-11	23253
DPY19L3	0.313153	4.01E-11	147991
CALD1	0.313046	4.08E-11	800
SERINC1	0.312964	4.13E-11	57515
TMEM221	0.312914	4.16E-11	100130519
GYPE	0.312912	4.16E-11	2996
SWT1	0.312872	4.18E-11	54823

NOTCH2NL	0.312762	4.25E-11	NA
TNPO1	0.312739	4.27E-11	3842
ZNF207	0.31264	4.33E-11	7756
GTF2H1	0.312626	4.34E-11	2965
RP11-244H3.4	0.312548	4.39E-11	NA
LHCGR	0.312518	4.41E-11	3973
YIPF5	0.312476	4.44E-11	81555
CRY1	0.31243	4.47E-11	1407
SLCO5A1	0.312384	4.50E-11	81796
ATP1A4	0.312359	4.52E-11	480
N4BP2	0.312324	4.54E-11	55728
FAM179B	0.31232	4.55E-11	NA
RAB30	0.31231	4.55E-11	27314
KLHL2	0.312248	4.59E-11	11275
PLEKHM3	0.31224	4.60E-11	389072
NSRP1	0.312117	4.68E-11	84081
KRT13	0.312104	4.69E-11	3860
RNF149	0.312099	4.70E-11	284996
SLC27A1	0.31198	4.78E-11	376497
TBCK	0.311974	4.79E-11	93627
CCDC90B	0.31197	4.79E-11	60492
PBRM1	0.311964	4.79E-11	55193
MPZL1	0.311938	4.81E-11	9019
PHACTR2	0.311883	4.85E-11	9749
ZSCAN20	0.311785	4.92E-11	7579
HCST	0.311757	4.94E-11	10870
SBSN	0.31163	5.04E-11	374897
IFT80	0.311614	5.05E-11	57560
YIPF4	0.311607	5.06E-11	84272
TGM1	0.311566	5.09E-11	7051
FXR1	0.31155	5.10E-11	8087
TIGIT	0.311518	5.12E-11	201633
PRRC1	0.311498	5.14E-11	133619
DOCK1	0.311497	5.14E-11	1793
MGA	0.311495	5.14E-11	23269
THAP2	0.311482	5.15E-11	83591
MGEA5	0.31144	5.18E-11	NA
ARHGAP10	0.31133	5.27E-11	79658
ZFP37	0.311328	5.27E-11	7539
SLC26A10	0.311262	5.32E-11	65012
5-3月	0.311226	5.35E-11	NA
RUNX3	0.311208	5.37E-11	864
RAPH1	0.311192	5.38E-11	65059
ADAM20	0.311118	5.44E-11	8748
STAC	0.311089	5.46E-11	6769
RBAK	0.311046	5.50E-11	57786
PRKAB2	0.311038	5.50E-11	5565
CWF19L2	0.310996	5.54E-11	143884
ZNF770	0.310985	5.55E-11	54989
ZMYND11	0.31096	5.57E-11	10771
TWISTNB	0.310862	5.65E-11	NA
MTPN	0.310794	5.71E-11	136319
CTSF	0.310769	5.73E-11	8722
PCNXL2	0.31069	5.79E-11	NA
SDAD1	0.310666	5.82E-11	55153
UBE2QL1	0.310632	5.84E-11	134111
PTPN11	0.31062	5.86E-11	5781
CYP27B1	0.310526	5.94E-11	1594
ATAD2B	0.310468	5.99E-11	54454
DCUN1D1	0.310374	6.07E-11	54165

TTC39C	0.310359	6.09E-11	125488
KRR1	0.310356	6.09E-11	11103
KIF27	0.310347	6.10E-11	55582
KRT6C	0.310305	6.14E-11	286887
KLHL3	0.310246	6.19E-11	26249
SIPA1	0.310141	6.29E-11	6494
CHML	0.31008	6.34E-11	1122
IL18BP	0.310059	6.36E-11	10068
CYP2U1	0.310054	6.37E-11	113612
UBASH3B	0.310033	6.39E-11	84959
DYNC1I2	0.30998	6.44E-11	1781
RABGAP1	0.309946	6.47E-11	23637
CD44	0.309915	6.50E-11	960
TNRC6B	0.309909	6.51E-11	23112
R3HCC1L	0.309909	6.51E-11	27291
TFE3	0.309811	6.60E-11	7030
FSD2	0.30981	6.60E-11	123722
EFCAB7	0.309781	6.63E-11	84455
BAG4	0.309698	6.71E-11	9530
KRTDAP	0.309594	6.82E-11	388533
UPF2	0.309593	6.82E-11	26019
KBTBD2	0.309567	6.84E-11	25948
PAK2	0.309552	6.86E-11	5062
MEIS1	0.309536	6.88E-11	4211
EVI2B	0.309528	6.88E-11	2124
SLCO1B7	0.309515	6.90E-11	338821
PTPN4	0.309486	6.93E-11	5775
SENP6	0.30948	6.93E-11	26054
INTU	0.309429	6.98E-11	27152
CNTN6	0.309428	6.99E-11	27255
YBX3	0.309427	6.99E-11	8531
HABP4	0.309412	7.00E-11	22927
FGF11	0.309398	7.02E-11	2256
CEBPB	0.309279	7.14E-11	1051
SBF2	0.309245	7.18E-11	81846
C16orf70	0.309092	7.34E-11	80262
RP11-219A15.1	0.309088	7.35E-11	NA
TBK1	0.309056	7.38E-11	29110
PML	0.30902	7.42E-11	5371
INO80D	0.308995	7.45E-11	54891
DDX6	0.308955	7.49E-11	1656
CCR4	0.308913	7.54E-11	1233
VPS13C	0.30891	7.54E-11	54832
TM2D1	0.308818	7.65E-11	83941
ARMC1	0.308791	7.68E-11	55156
BHLHE40	0.308747	7.73E-11	8553
MTAP	0.308718	7.76E-11	4507
ZSCAN2	0.308674	7.81E-11	54993
NCEH1	0.308592	7.90E-11	57552
IFI44L	0.308571	7.93E-11	10964
ACTA2	0.308563	7.94E-11	59
C11orf30	0.308536	7.97E-11	NA
ANKRD40	0.308519	7.99E-11	91369
ZNF431	0.308497	8.01E-11	170959
GATSL2	0.308496	8.02E-11	NA
RCBTB1	0.308416	8.11E-11	55213
VSIG10L	0.308394	8.14E-11	147645
CCDC91	0.308348	8.19E-11	55297
ATXN7L1	0.308337	8.21E-11	222255
AASDHPPT	0.30831	8.24E-11	60496

TMEM167A	0.308226	8.34E-11	153339
KCNAB2	0.308041	8.57E-11	8514
RASSF9	0.308029	8.59E-11	9182
SLC9B1	0.307985	8.64E-11	150159
SLC29A3	0.307981	8.65E-11	55315
ERC1	0.307921	8.72E-11	23085
MACC1	0.307902	8.75E-11	346389
ZNF609	0.307897	8.76E-11	23060
CEP57L1	0.307887	8.77E-11	285753
RBBP6	0.307878	8.78E-11	5930
TRAF6	0.307791	8.89E-11	7189
TMEM200B	0.307773	8.92E-11	399474
MAN1C1	0.307753	8.94E-11	57134
KRT6A	0.307746	8.95E-11	3853
EXTL1	0.307714	8.99E-11	2134
GABPB1	0.307584	9.17E-11	2553
DDHD2	0.30758	9.17E-11	23259
RAI1	0.307568	9.19E-11	10743
SYNGAP1	0.307511	9.27E-11	8831
FPGT-TNNI3K	0.307506	9.27E-11	100526835
TRIP11	0.307493	9.29E-11	9321
KPNA6	0.307489	9.30E-11	23633
TRIP12	0.307461	9.33E-11	9320
PPP3CC	0.307447	9.35E-11	5533
ATP6AP2	0.307446	9.35E-11	10159
IFFO2	0.307397	9.42E-11	126917
AP4E1	0.307361	9.47E-11	23431
FER	0.307355	9.48E-11	2241
5-9月	0.307258	9.62E-11	NA
S100A12	0.307199	9.70E-11	6283
ZNF433	0.307186	9.72E-11	163059
CEP128	0.307158	9.76E-11	145508
TREML4	0.307114	9.82E-11	285852
G2E3	0.307086	9.86E-11	55632
PRRC2C	0.306993	1.00E-10	23215
ARHGAP25	0.306975	1.00E-10	9938
ARHGEF12	0.306929	1.01E-10	23365
PCDHGB6	0.306901	1.01E-10	56100
RBMXL1	0.306875	1.02E-10	494115
TANGO6	0.306868	1.02E-10	79613
FAM208B	0.306836	1.02E-10	NA
INVS	0.306827	1.02E-10	27130
ORAI2	0.306651	1.05E-10	80228
TERF2IP	0.306647	1.05E-10	54386
LRP2BP	0.306611	1.06E-10	55805
FCGR3B	0.306598	1.06E-10	2215
2-9月	0.306545	1.07E-10	NA
APPBP2	0.306531	1.07E-10	10513
ADAM22	0.306481	1.08E-10	53616
FGF2	0.306479	1.08E-10	2247
FES	0.306239	1.12E-10	2242
MYO9A	0.306231	1.12E-10	4649
RNF111	0.306195	1.12E-10	54778
CHIC2	0.306186	1.12E-10	26511
PRSS35	0.306107	1.14E-10	167681
GNA15	0.306085	1.14E-10	2769
CTC-487M23.8	0.305992	1.16E-10	NA
FNIP1	0.305956	1.16E-10	96459
PCDH18	0.305912	1.17E-10	54510
LCP1	0.305891	1.17E-10	3936

ZNF146	0.305883	1.18E-10	7705
EPC2	0.30584	1.18E-10	26122
AC087350.1	0.305815	1.19E-10	NA
WASF1	0.305783	1.19E-10	8936
ODF2L	0.305755	1.20E-10	57489
ANKLE2	0.305742	1.20E-10	23141
GRASP	0.305648	1.22E-10	NA
MTMR6	0.305617	1.22E-10	9107
TMEM79	0.305601	1.23E-10	84283
B3GNT2	0.305589	1.23E-10	10678
ZNF426	0.305564	1.23E-10	79088
TRPC5	0.305541	1.24E-10	7224
ZNF507	0.305524	1.24E-10	22847
MYO1B	0.30552	1.24E-10	4430
TMEM65	0.305487	1.25E-10	157378
LAPTM4A	0.305374	1.27E-10	9741
CRCT1	0.305368	1.27E-10	54544
SCAF8	0.30531	1.28E-10	22828
FAM127A	0.305276	1.28E-10	NA
FAM78A	0.30523	1.29E-10	286336
SPOCK2	0.305222	1.29E-10	9806
FAM76B	0.305221	1.29E-10	143684
DDX21	0.305164	1.31E-10	9188
RRM2B	0.30514	1.31E-10	50484
SLAMF9	0.30507	1.32E-10	89886
KDELC2	0.305062	1.33E-10	NA
CEP350	0.304974	1.34E-10	9857
NIPBL	0.304912	1.35E-10	25836
NFATC4	0.304886	1.36E-10	4776
GRB10	0.304871	1.36E-10	2887
NEK11	0.304853	1.37E-10	79858
SH3RF3	0.30485	1.37E-10	344558
CNOT6	0.304814	1.37E-10	57472
PTBP2	0.304811	1.37E-10	58155
SHPRH	0.304778	1.38E-10	257218
SETX	0.304774	1.38E-10	23064
CTD-2370N5.3	0.304766	1.38E-10	NA
NBPF9	0.304763	1.38E-10	400818
ZNF638	0.304682	1.40E-10	27332
MTX3	0.304566	1.42E-10	345778
C11orf58	0.30453	1.43E-10	10944
CANX	0.304491	1.44E-10	821
METTL6	0.304453	1.45E-10	131965
MAPKBP1	0.304452	1.45E-10	23005
KLHL21	0.304445	1.45E-10	9903
PKD2L2	0.304441	1.45E-10	27039
COX4I2	0.304431	1.45E-10	84701
TBC1D8B	0.304425	1.45E-10	54885
USP6NL	0.3044	1.46E-10	9712
TUBA1A	0.304377	1.46E-10	7846
DLGAP4	0.304334	1.47E-10	22839
ATF6	0.304331	1.47E-10	22926
SREK1	0.304286	1.48E-10	140890
CNOT2	0.304238	1.49E-10	4848
FAM49B	0.304215	1.50E-10	NA
ARID4A	0.304199	1.50E-10	5926
RRAGD	0.304183	1.51E-10	58528
ZC3H11A	0.30403	1.54E-10	9877
JAKMIP1	0.304012	1.54E-10	152789
KANSL1	0.304004	1.55E-10	284058

GBP6	0.303981	1.55E-10	163351
KLK9	0.303979	1.55E-10	284366
C3orf17	0.303933	1.56E-10	NA
CRNN	0.303823	1.59E-10	49860
ZC3H6	0.303791	1.59E-10	376940
PPIL4	0.30377	1.60E-10	85313
ITPR2	0.303716	1.61E-10	3709
LRRFIP1	0.303703	1.61E-10	9208
MYO16	0.303614	1.63E-10	23026
FCRL6	0.303569	1.65E-10	343413
CXCR4	0.303474	1.67E-10	7852
KIAA1328	0.303449	1.67E-10	57536
NRIP3	0.303449	1.67E-10	56675
CCDC39	0.303415	1.68E-10	339829
MARK4	0.303335	1.70E-10	57787
DDX17	0.303329	1.70E-10	10521
ZNF654	0.303323	1.70E-10	55279
FAM13B	0.303265	1.72E-10	51306
PLEKHM2	0.303258	1.72E-10	23207
EPC1	0.303251	1.72E-10	80314
CEP68	0.30325	1.72E-10	23177
LRRC37B	0.303247	1.72E-10	114659
RAB22A	0.303183	1.74E-10	57403
BCLAF1	0.303145	1.75E-10	9774
CNOT4	0.303131	1.75E-10	4850
KDM5B	0.303105	1.76E-10	10765
GJB4	0.303089	1.76E-10	127534
RC3H1	0.30308	1.77E-10	149041
LCE3D	0.303047	1.77E-10	84648
RGS3	0.303039	1.78E-10	5998
VEGFB	0.303036	1.78E-10	7423
SARNP	0.303029	1.78E-10	84324
IVL	0.30302	1.78E-10	3713
ZFP28	0.30296	1.80E-10	140612
DNAH11	0.302935	1.80E-10	8701
RP11-286N22.8	0.302912	1.81E-10	NA
KCTD15	0.30288	1.82E-10	79047
MTFR1	0.302828	1.83E-10	9650
MEF2C	0.3028	1.84E-10	4208
ANKRD26	0.302771	1.85E-10	22852
RASSF5	0.302697	1.87E-10	83593
CLUAP1	0.302668	1.87E-10	23059
C19orf44	0.302643	1.88E-10	84167
IQSEC3	0.302592	1.89E-10	440073
BDP1	0.302578	1.90E-10	55814
APLF	0.302578	1.90E-10	200558
CHRM3	0.302556	1.90E-10	1131
GATAD2B	0.302472	1.93E-10	57459
SPRTN	0.302434	1.94E-10	83932
BTAF1	0.30238	1.95E-10	9044
YWHAH	0.302377	1.95E-10	7533
STAT5B	0.302327	1.97E-10	6777
KRT78	0.302262	1.99E-10	196374
DTX3L	0.302259	1.99E-10	151636
PDP1	0.302254	1.99E-10	54704
SPRR2B	0.302251	1.99E-10	6701
ATP6V1B2	0.302193	2.01E-10	526
SEC23IP	0.302152	2.02E-10	11196
SSH2	0.302146	2.02E-10	85464
CUL4B	0.302119	2.03E-10	8450

PTBP3	0.302043	2.05E-10	9991
MED13L	0.302027	2.06E-10	23389
GLG1	0.301935	2.08E-10	2734
KIAA1467	0.301933	2.08E-10	NA
UBA6	0.301917	2.09E-10	55236
NLRP1	0.301865	2.10E-10	22861
DCAF10	0.301856	2.11E-10	79269
SAMD4B	0.301835	2.11E-10	55095
RASGEF1B	0.301824	2.12E-10	153020
KLHL18	0.301813	2.12E-10	23276
ZNF286B	0.301721	2.15E-10	729288
KRT14	0.301705	2.15E-10	3861
PARP15	0.301662	2.17E-10	165631
HELQ	0.301617	2.18E-10	113510
SERPINB13	0.301608	2.18E-10	5275
CCDC102A	0.301596	2.19E-10	92922
PJA1	0.301585	2.19E-10	64219
ARRB2	0.30157	2.19E-10	409
SEMA4C	0.301569	2.19E-10	54910
ALG10B	0.301472	2.23E-10	144245
TBC1D23	0.301438	2.24E-10	55773
ARSK	0.301433	2.24E-10	153642
BRMS1L	0.301426	2.24E-10	84312
WTAP	0.301392	2.25E-10	9589
NIPSNAP3B	0.301377	2.26E-10	55335
NKX3-2	0.30137	2.26E-10	579
TM4SF1	0.301368	2.26E-10	4071
NUP153	0.301357	2.26E-10	9972
BHLHB9	0.30133	2.27E-10	80823
SLC4A1AP	0.301324	2.27E-10	22950
PALB2	0.301309	2.28E-10	79728
ZNF728	0.301248	2.30E-10	388523
DNAJB14	0.30117	2.32E-10	79982
RSU1	0.301098	2.35E-10	6251
LMLN	0.301051	2.36E-10	89782
BHLHE41	0.301	2.38E-10	79365
MERTK	0.300985	2.39E-10	10461
SIN3B	0.300972	2.39E-10	23309
LDLRAD4	0.300898	2.42E-10	753
GLB1L	0.300849	2.43E-10	79411
FAM122C	0.300772	2.46E-10	159091
MLLT10	0.300684	2.49E-10	8028
POU2F1	0.300665	2.50E-10	5451
ARL5A	0.300589	2.53E-10	26225
KRT5	0.30057	2.53E-10	3852
NT5C2	0.300517	2.55E-10	22978
LEF1	0.300511	2.55E-10	51176
USP37	0.30051	2.55E-10	57695
UBXN2B	0.300508	2.55E-10	137886
TMEM200A	0.300493	2.56E-10	114801
UBFD1	0.300375	2.60E-10	56061
ITSN2	0.300362	2.61E-10	50618
UHRF1BP1	0.300337	2.62E-10	54887
AEBP2	0.300297	2.63E-10	121536
SEL1L	0.300247	2.65E-10	6400
WAS	0.300188	2.67E-10	7454
SPRR2E	0.300116	2.70E-10	6704
MAP1A	0.300056	2.73E-10	4130
NRBP1	0.300052	2.73E-10	29959
C2orf49	0.299895	2.79E-10	79074

SLC5A3	0.299887	2.79E-10	6526
KIAA0430	0.299873	2.80E-10	NA
AGO2	0.299866	2.80E-10	27161
BHMT2	0.299865	2.80E-10	23743
CYSRT1	0.299788	2.83E-10	375791
PLCB2	0.299742	2.85E-10	5330
IPO7	0.299711	2.86E-10	10527
MANEA	0.2997	2.87E-10	79694
LYN	0.299698	2.87E-10	4067
PIM1	0.299589	2.91E-10	5292
TLDC1	0.299453	2.97E-10	NA
HERC1	0.299419	2.98E-10	8925
IFIT3	0.299414	2.99E-10	3437
EYA3	0.299393	3.00E-10	2140
EXOC2	0.299383	3.00E-10	55770
AP3S1	0.299363	3.01E-10	1176
DCUN1D4	0.299216	3.07E-10	23142
RAP2C	0.299072	3.14E-10	57826
JMY	0.299021	3.16E-10	133746
TNFSF12-TNFSF13	0.299021	3.16E-10	407977
SLC23A2	0.299005	3.17E-10	9962
FBXL13	0.29895	3.19E-10	222235
CFAP69	0.298915	3.21E-10	79846
C10orf88	0.298753	3.28E-10	80007
CYB5R3	0.298701	3.30E-10	1727
ZC3H7A	0.2987	3.31E-10	29066
LETM2	0.298657	3.33E-10	137994
NEXN	0.298625	3.34E-10	91624
MCF2L2	0.298562	3.37E-10	23101
JAK2	0.298472	3.41E-10	3717
FBXO33	0.298463	3.42E-10	254170
HRH1	0.298445	3.43E-10	3269
ASH1L	0.298416	3.44E-10	55870
ARHGAP5	0.298411	3.44E-10	394
GRIN2B	0.298407	3.45E-10	2904
SSR1	0.298387	3.46E-10	6745
GAPVD1	0.298382	3.46E-10	26130
ARID5A	0.298377	3.46E-10	10865
AKAP11	0.298351	3.47E-10	11215
ST8SIA1	0.298332	3.48E-10	6489
PDS5B	0.298331	3.48E-10	23047
VOPP1	0.298315	3.49E-10	81552
STAT4	0.298293	3.50E-10	6775
ADAT1	0.298288	3.50E-10	23536
B3GALNT2	0.298286	3.50E-10	148789
GON4L	0.298276	3.51E-10	54856
TANC1	0.298151	3.57E-10	85461
ZFP69B	0.298124	3.59E-10	65243
SMARCD3	0.298122	3.59E-10	6604
ZBTB6	0.298102	3.60E-10	10773
ARRDC2	0.298072	3.61E-10	27106
TBC1D5	0.298036	3.63E-10	9779
ARHGAP22	0.298019	3.64E-10	58504
TMEM194B	0.298007	3.65E-10	NA
ZNF254	0.297939	3.68E-10	9534
RASA2	0.297933	3.68E-10	5922
SPRR1B	0.297883	3.71E-10	6699
PAFAH1B2	0.297848	3.73E-10	5049
PCDHGA11	0.297788	3.76E-10	56105
METTL8	0.297765	3.77E-10	79828

LDB1	0.297746	3.78E-10	8861
PHC2	0.29773	3.79E-10	1912
TPTE2	0.297718	3.80E-10	93492
TMEM117	0.297696	3.81E-10	84216
IGF2R	0.297672	3.82E-10	3482
CSF2RB	0.297602	3.86E-10	1439
CCDC126	0.297598	3.86E-10	90693
RABEP1	0.297574	3.88E-10	9135
ZKSCAN5	0.297567	3.88E-10	23660
RSPRY1	0.297537	3.90E-10	89970
ZNF331	0.297536	3.90E-10	55422
L3HYPDH	0.297517	3.91E-10	112849
NHLRC4	0.297491	3.92E-10	283948
CDK6	0.297437	3.95E-10	1021
SPRR2D	0.297415	3.96E-10	6703
NPAS3	0.297395	3.98E-10	64067
LPP	0.297336	4.01E-10	4026
THBS4	0.297334	4.01E-10	7060
ZNF660	0.297333	4.01E-10	285349
RAB21	0.297238	4.06E-10	23011
NOC3L	0.297185	4.10E-10	64318
ARF4	0.297165	4.11E-10	378
CD302	0.297161	4.11E-10	9936
TMEM233	0.297154	4.11E-10	387890
ICAM2	0.297151	4.11E-10	3384
ZNF131	0.297108	4.14E-10	7690
MATR3	0.297105	4.14E-10	9782
ZC3H13	0.297087	4.15E-10	23091
KDSR	0.296937	4.24E-10	2531
GLTSCR1L	0.296921	4.25E-10	NA
CCM2L	0.296899	4.26E-10	140706
CREBL2	0.29689	4.27E-10	1389
SLC16A6	0.29687	4.28E-10	9120
HIPK1	0.296793	4.33E-10	204851
JAK1	0.296773	4.34E-10	3716
DMP1	0.296639	4.42E-10	1758
DYNC1LI2	0.296616	4.44E-10	1783
RAB41	0.29661	4.44E-10	347517
KPNA3	0.296592	4.45E-10	3839
DLG2	0.296542	4.48E-10	1740
MAP3K4	0.296527	4.49E-10	4216
RBM12	0.296525	4.49E-10	10137
ZNF410	0.29617	4.72E-10	57862
TMEM169	0.296167	4.73E-10	92691
ZNF548	0.296142	4.74E-10	147694
CPSF6	0.295962	4.86E-10	11052
TRIM13	0.295953	4.87E-10	10206
INTS6	0.295904	4.90E-10	26512
TSPAN10	0.295875	4.92E-10	83882
SPATA12	0.295837	4.95E-10	353324
INPP5B	0.295791	4.98E-10	3633
RS1	0.295772	5.00E-10	6247
WFDC5	0.295714	5.04E-10	149708
MAGEL2	0.295674	5.07E-10	54551
ZNF510	0.295661	5.07E-10	22869
NMD3	0.295657	5.08E-10	51068
CNOT6L	0.295588	5.13E-10	246175
SPRR2A	0.29557	5.14E-10	6700
YTHDF3	0.295494	5.19E-10	253943
C11orf45	0.29549	5.20E-10	219833

ZNF780A	0.295482	5.20E-10	284323
ZNF236	0.29546	5.22E-10	7776
RBP7	0.295454	5.22E-10	116362
ARL14EP	0.295422	5.25E-10	120534
XPR1	0.295415	5.25E-10	9213
TMEM110-MUSTN1	0.295357	5.30E-10	NA
ZCCHC14	0.295353	5.30E-10	23174
FBXO38	0.295302	5.34E-10	81545
SPATS2	0.295284	5.35E-10	65244
ERCC6L2	0.295253	5.37E-10	375748
FAM228B	0.29523	5.39E-10	375190
MFSD7	0.295198	5.41E-10	NA
FAM216B	0.295192	5.42E-10	144809
TMEM106B	0.295175	5.43E-10	54664
AGFG1	0.295154	5.45E-10	3267
ISPD	0.295151	5.45E-10	NA
TRDMT1	0.295094	5.49E-10	1787
TLN1	0.295081	5.50E-10	7094
ZNF713	0.295075	5.51E-10	349075
PIK3R4	0.295047	5.53E-10	30849
CERKL	0.295038	5.54E-10	375298
SHE	0.294999	5.57E-10	126669
ZNF644	0.29495	5.61E-10	84146
ARHGDIB	0.294942	5.61E-10	397
DHX57	0.294929	5.62E-10	90957
TRAF3IP3	0.294811	5.72E-10	80342
ZSCAN23	0.294767	5.75E-10	222696
ZZZ3	0.294746	5.77E-10	26009
C11orf63	0.294667	5.83E-10	NA
KIAA1549L	0.294652	5.84E-10	25758
UHMK1	0.294638	5.86E-10	127933
MYH1	0.294632	5.86E-10	4619
ATP6V1A	0.294613	5.88E-10	523
SIGLEC1	0.294593	5.89E-10	6614
ZYG11B	0.294519	5.95E-10	79699
CD200	0.294503	5.97E-10	4345
FZD6	0.294494	5.97E-10	8323
ATG4C	0.294492	5.98E-10	84938
SIK2	0.294432	6.03E-10	23235
SLC43A3	0.294376	6.07E-10	29015
CEP290	0.294372	6.08E-10	80184
KIAA1614	0.294363	6.09E-10	57710
BLZF1	0.294248	6.18E-10	8548
POLR2M	0.294233	6.20E-10	81488
TMOD2	0.294228	6.20E-10	29767
NAA25	0.294221	6.21E-10	80018
PEX5L	0.29422	6.21E-10	51555
ALOX5	0.294215	6.21E-10	240
MAPRE2	0.29421	6.22E-10	10982
CDC5L	0.29416	6.26E-10	988
KANK3	0.294134	6.28E-10	256949
ZBTB25	0.294088	6.32E-10	7597
PGM3	0.294076	6.33E-10	5238
CRTAP	0.293983	6.42E-10	10491
KPNA4	0.293958	6.44E-10	3840
PAK3	0.29395	6.45E-10	5063
FAM149B1	0.293933	6.46E-10	317662
TTF1	0.293925	6.47E-10	7270
TTLL1	0.29392	6.47E-10	25809
TTC9	0.293905	6.49E-10	23508

BIRC2	0.293874	6.51E-10	329
PDE4DIP	0.293856	6.53E-10	9659
HBS1L	0.293835	6.55E-10	10767
SH3BGRL	0.293787	6.59E-10	6451
ZNF225	0.293763	6.62E-10	7768
ZNF287	0.29374	6.64E-10	57336
CCDC74A	0.293685	6.69E-10	90557
SDC4	0.293511	6.85E-10	6385
C2CD3	0.2935	6.86E-10	26005
DCAF17	0.293381	6.98E-10	80067
MEAF6	0.293332	7.03E-10	64769
PLEKHA4	0.29333	7.03E-10	57664
PHF6	0.293319	7.04E-10	84295
BAZ1A	0.293303	7.05E-10	11177
ZBTB38	0.293276	7.08E-10	253461
ARL2BP	0.293245	7.11E-10	23568
RAB3GAP1	0.293244	7.11E-10	22930
STK4	0.293185	7.17E-10	6789
PURB	0.293179	7.18E-10	5814
STX17	0.293157	7.20E-10	55014
CDR2	0.293152	7.20E-10	1039
PTGFRN	0.293087	7.27E-10	5738
TMEM170B	0.293073	7.28E-10	100113407
WDR66	0.29307	7.28E-10	NA
WDFY4	0.293057	7.30E-10	57705
PIK3C3	0.293009	7.35E-10	5289
NDST3	0.293008	7.35E-10	9348
BMPR1A	0.292971	7.39E-10	657
MRVI1	0.292967	7.39E-10	NA
ZCCHC7	0.292966	7.39E-10	84186
APOC4-APOC2	0.292954	7.40E-10	100533990
KCNQ3	0.292924	7.43E-10	3786
GMFG	0.292884	7.48E-10	9535
ZRSR1	0.292869	7.49E-10	NA
AGRP	0.292779	7.59E-10	181
NPC2	0.292714	7.65E-10	10577
ATP11C	0.292709	7.66E-10	286410
SLC31A2	0.292591	7.79E-10	1318
FCF1	0.292571	7.81E-10	51077
TNS2	0.292556	7.82E-10	23371
USP49	0.292531	7.85E-10	25862
TRNT1	0.292499	7.89E-10	51095
FAM171A2	0.292476	7.91E-10	284069
HNRNPR	0.292442	7.95E-10	10236
CCAR1	0.292434	7.96E-10	55749
NBPF12	0.29243	7.96E-10	149013
GATSL3	0.292392	8.00E-10	NA
7-3月	0.292337	8.07E-10	NA
CLCA2	0.292281	8.13E-10	9635
MS4A6A	0.292278	8.13E-10	64231
DOCK8	0.292277	8.13E-10	81704
SON	0.292242	8.17E-10	6651
ZNF668	0.292237	8.18E-10	79759
NDST2	0.292164	8.26E-10	8509
MCM9	0.292163	8.26E-10	254394
IQUB	0.292102	8.33E-10	154865
GPC1	0.292094	8.34E-10	2817
FP325317.1	0.292079	8.36E-10	NA
TACSTD2	0.292057	8.38E-10	4070
CRTAC1	0.292021	8.43E-10	55118

CHD6	0.292014	8.43E-10	84181
SPATA7	0.291889	8.58E-10	55812
HSDL1	0.291881	8.59E-10	83693
RNF38	0.291831	8.65E-10	152006
CPNE3	0.291814	8.67E-10	8895
CNTLN	0.291812	8.67E-10	54875
GABRR2	0.291812	8.67E-10	2570
RAD51B	0.291808	8.68E-10	5890
FAM182B	0.291784	8.71E-10	728882
SIAH1	0.291652	8.87E-10	6477
TMX4	0.291611	8.92E-10	56255
HYAL2	0.291603	8.93E-10	8692
RASL11B	0.291603	8.93E-10	65997
LAMB4	0.291511	9.04E-10	22798
DESI2	0.291386	9.20E-10	51029
NCDN	0.291369	9.22E-10	23154
MKLN1	0.291324	9.28E-10	4289
PARP9	0.291291	9.32E-10	83666
PRRG1	0.291234	9.39E-10	5638
ZMYM2	0.291231	9.40E-10	7750
VPS8	0.291193	9.45E-10	23355
PCYOX1	0.291173	9.47E-10	51449
BTK	0.291151	9.50E-10	695
ZNF568	0.291136	9.52E-10	374900
LZTS2	0.291122	9.54E-10	84445
CREBBP	0.291063	9.62E-10	1387
TIA1	0.291035	9.65E-10	7072
PUS7L	0.290961	9.75E-10	83448
CD36	0.290946	9.77E-10	948
YLPM1	0.290921	9.81E-10	56252
WDR36	0.290915	9.81E-10	134430
C4orf45	0.290851	9.90E-10	152940
ST3GAL3	0.290842	9.91E-10	6487
OAS3	0.290689	1.01E-09	4940
ARNTL2	0.290681	1.01E-09	56938
C3orf36	0.29067	1.02E-09	80111
PSD3	0.29066	1.02E-09	23362
TRPC4	0.290653	1.02E-09	7223
WAC	0.290644	1.02E-09	51322
ADAM21	0.29056	1.03E-09	8747
MLH3	0.29048	1.04E-09	27030
MYH13	0.290437	1.05E-09	8735
DDX50	0.290394	1.05E-09	79009
DLG5	0.290347	1.06E-09	9231
LARP4	0.290264	1.07E-09	113251
FLT3LG	0.290261	1.07E-09	2323
ZNF175	0.290251	1.08E-09	7728
ATP2C1	0.290245	1.08E-09	27032
SEPN1	0.290228	1.08E-09	NA
RBM4	0.29019	1.08E-09	5936
ZNF566	0.290151	1.09E-09	84924
TFIP11	0.290132	1.09E-09	24144
ZNF407	0.290124	1.09E-09	55628
SP3	0.290111	1.10E-09	6670
PHYHD1	0.290033	1.11E-09	254295
SMAD2	0.290033	1.11E-09	4087
EVA1A	0.290001	1.11E-09	84141
TERF1	0.289996	1.11E-09	7013
NAP1L1	0.289955	1.12E-09	4673
RFX5	0.289939	1.12E-09	5993

CCDC66	0.28993	1.12E-09	285331
ARFGAP3	0.289922	1.13E-09	26286
SYNJ2	0.289904	1.13E-09	8871
TBPL1	0.289897	1.13E-09	9519
S100A7	0.289823	1.14E-09	6278
AOC2	0.28976	1.15E-09	314
AKNAD1	0.289737	1.15E-09	254268
NSD1	0.289646	1.17E-09	64324
PUM1	0.289622	1.17E-09	9698
MEOX2	0.289622	1.17E-09	4223
GTF2E1	0.289616	1.17E-09	2960
DEPTOR	0.289615	1.17E-09	64798
NRBF2	0.289586	1.18E-09	29982
SUPT20H	0.289549	1.18E-09	55578
ARHGAP21	0.289543	1.18E-09	57584
UBR5	0.289533	1.19E-09	51366
WEE2	0.289522	1.19E-09	494551
PPP1R9B	0.289508	1.19E-09	84687
PTGES	0.289502	1.19E-09	9536
RAET1E	0.289476	1.20E-09	135250
SF3B1	0.28944	1.20E-09	23451
PTK2	0.289389	1.21E-09	5747
TPR	0.289389	1.21E-09	7175
CHM	0.289222	1.24E-09	1121
NCALD	0.289086	1.26E-09	83988
BRWD3	0.289071	1.26E-09	254065
PLCL1	0.289061	1.27E-09	5334
VTI1A	0.289058	1.27E-09	143187
EDNRA	0.289048	1.27E-09	1909
SPCS3	0.289035	1.27E-09	60559
FAM227B	0.289019	1.27E-09	196951
ATXN2	0.288928	1.29E-09	6311
SLC35F3	0.288916	1.29E-09	148641
YPEL1	0.288912	1.29E-09	29799
EP300	0.288896	1.29E-09	2033
CTD-3088G3.8	0.288848	1.30E-09	NA
GTF3C3	0.288843	1.30E-09	9330
SRSF10	0.288838	1.30E-09	10772
RHBDF2	0.288757	1.32E-09	79651
SERPINB3	0.288734	1.32E-09	6317
CARF	0.28873	1.32E-09	79800
PTPRE	0.288696	1.33E-09	5791
ZNF33A	0.28864	1.34E-09	7581
MGAT1	0.288639	1.34E-09	4245
FAM73A	0.288629	1.34E-09	NA
MREG	0.288481	1.37E-09	55686
CHRNE	0.288479	1.37E-09	1145
TRAF3IP1	0.288457	1.37E-09	26146
PHF20	0.288418	1.38E-09	51230
KLC1	0.288406	1.38E-09	3831
RETN	0.288379	1.39E-09	56729
JARID2	0.288366	1.39E-09	3720
SLIT3	0.288363	1.39E-09	6586
C3orf18	0.288353	1.39E-09	51161
ACKR3	0.288345	1.40E-09	57007
FEM1B	0.288344	1.40E-09	10116
TRIM4	0.288344	1.40E-09	89122
ZC3HAV1	0.288327	1.40E-09	56829
SBK3	0.288288	1.41E-09	100130827
NEK9	0.288258	1.41E-09	91754

RP11-849F2.7	0.288216	1.42E-09	NA
NSMAF	0.288183	1.43E-09	8439
CEP164	0.288143	1.43E-09	22897
C5orf42	0.288013	1.46E-09	NA
PEAR1	0.287996	1.46E-09	375033
ZMYM5	0.287976	1.47E-09	9205
TYMP	0.287955	1.47E-09	1890
ZNF829	0.287942	1.47E-09	374899
PAIP1	0.287906	1.48E-09	10605
RYK	0.287886	1.49E-09	6259
C18orf54	0.287777	1.51E-09	162681
RNF215	0.287773	1.51E-09	200312
TUSC3	0.287768	1.51E-09	7991
NDEL1	0.287764	1.51E-09	81565
CCND1	0.287744	1.51E-09	595
BRAF	0.287735	1.52E-09	673
OGFOD1	0.287729	1.52E-09	55239
TMF1	0.287706	1.52E-09	7110
RSRC2	0.287688	1.53E-09	65117
NOL8	0.287687	1.53E-09	55035
RABGAP1L	0.287685	1.53E-09	9910
NCOR2	0.287684	1.53E-09	9612
MOB1A	0.28763	1.54E-09	55233
CHUK	0.287617	1.54E-09	1147
SMARCA5	0.287611	1.54E-09	8467
GSK3B	0.287516	1.56E-09	2932
MUC15	0.287515	1.56E-09	143662
C8orf44-SGK3	0.287485	1.57E-09	100533105
ANKRD34A	0.287457	1.57E-09	284615
ZNF471	0.287298	1.61E-09	57573
CTSH	0.287295	1.61E-09	1512
ZNF214	0.287294	1.61E-09	7761
ARCN1	0.287289	1.61E-09	372
PAPLN	0.287185	1.63E-09	89932
TP53INP1	0.287181	1.63E-09	94241
FAM175A	0.287141	1.64E-09	NA
SPRR2G	0.287106	1.65E-09	6706
TES	0.2871	1.65E-09	26136
SEL1L2	0.287093	1.65E-09	80343
C2orf42	0.287035	1.67E-09	54980
UBE3D	0.287019	1.67E-09	90025
CHD8	0.286992	1.68E-09	57680
CNTRL	0.286964	1.68E-09	11064
KDM3A	0.286899	1.70E-09	55818
KIAA0586	0.286822	1.72E-09	9786
CNEP1R1	0.286821	1.72E-09	255919
PUM2	0.286757	1.73E-09	23369
GPSM1	0.286709	1.74E-09	26086
CCNT1	0.286706	1.74E-09	904
UCN2	0.286701	1.75E-09	90226
BLOC1S5	0.286665	1.75E-09	63915
NOXRED1	0.286645	1.76E-09	122945
RBM41	0.286515	1.79E-09	55285
MDN1	0.286475	1.80E-09	23195
CLEC4C	0.28646	1.80E-09	170482
PCDHB4	0.286457	1.80E-09	56131
RRP15	0.2864	1.82E-09	51018
NFYA	0.286369	1.83E-09	4800
MAGI2	0.286322	1.84E-09	9863
SIPA1L1	0.286232	1.86E-09	26037

NPHP1	0.28622	1.86E-09	4867
C9orf91	0.286218	1.86E-09	NA
FAM175B	0.286173	1.87E-09	NA
HGSNAT	0.286117	1.89E-09	138050
FSCN1	0.286107	1.89E-09	6624
CPA4	0.286086	1.90E-09	51200
NINJ2	0.286045	1.91E-09	4815
DDX24	0.285934	1.94E-09	57062
ICK	0.285928	1.94E-09	NA
USP46	0.285876	1.95E-09	64854
HLA-DPB1	0.285826	1.96E-09	3115
ST5	0.285775	1.98E-09	NA
PIP4K2B	0.285729	1.99E-09	8396
DEK	0.285685	2.00E-09	7913
CELF1	0.285666	2.01E-09	10658
CD40	0.285652	2.01E-09	958
UBQLN2	0.285648	2.01E-09	29978
ANGEL2	0.285604	2.02E-09	90806
RPAP3	0.285553	2.04E-09	79657
FAM196A	0.285533	2.04E-09	NA
FJX1	0.285513	2.05E-09	24147
COMMD2	0.285485	2.06E-09	51122
ZNF17	0.28536	2.09E-09	7565
RBM25	0.285338	2.10E-09	58517
TTL	0.285312	2.11E-09	150465
ZBTB34	0.285302	2.11E-09	403341
PTCD2	0.285295	2.11E-09	79810
NCOA2	0.285193	2.14E-09	10499
ACOT9	0.285152	2.15E-09	23597
ITGB1BP1	0.285133	2.16E-09	9270
DBR1	0.285115	2.16E-09	51163
GDF11	0.285068	2.18E-09	10220
ZNF501	0.285061	2.18E-09	115560
PRPF4B	0.285051	2.18E-09	8899
ADO	0.284983	2.20E-09	84890
WBP4	0.284956	2.21E-09	11193
SEMA6C	0.284951	2.21E-09	10500
AGTPBP1	0.284928	2.22E-09	23287
LPL	0.284889	2.23E-09	4023
KLK13	0.284818	2.25E-09	26085
C6orf62	0.284788	2.26E-09	81688
ABRA	0.284779	2.26E-09	137735
TRERF1	0.284745	2.27E-09	55809
TNKS1BP1	0.28473	2.28E-09	85456
OXR1	0.284729	2.28E-09	55074
PLEKHO1	0.284701	2.29E-09	51177
USP51	0.284682	2.29E-09	158880
SBNO1	0.284568	2.33E-09	55206
PSMD5	0.2845	2.35E-09	5711
CHD2	0.284436	2.37E-09	1106
CXCL6	0.284421	2.37E-09	6372
ZNF423	0.284407	2.38E-09	23090
CENPC	0.284366	2.39E-09	1060
C16orf86	0.284332	2.40E-09	388284
TMEM256-PLSCR3	0.284268	2.42E-09	100529211
SMYD3	0.28425	2.43E-09	64754
CMTR1	0.28422	2.44E-09	23070
INTS4	0.284216	2.44E-09	92105
SESN3	0.284172	2.45E-09	143686
EPB42	0.284144	2.46E-09	2038

ZMYM6	0.284141	2.46E-09	9204
PMCH	0.284139	2.46E-09	5367
CASC4	0.284128	2.47E-09	NA
PARN	0.284127	2.47E-09	5073
PROK1	0.284124	2.47E-09	84432
DDX19A	0.284069	2.49E-09	55308
POU6F1	0.284067	2.49E-09	5463
ZBTB44	0.284066	2.49E-09	29068
KCTD12	0.284062	2.49E-09	115207
PROX1	0.28388	2.55E-09	5629
ZFAT	0.283866	2.56E-09	57623
MPP6	0.283859	2.56E-09	51678
SOAT1	0.283849	2.56E-09	6646
DYM	0.283828	2.57E-09	54808
TRMT13	0.283718	2.61E-09	54482
LZTFL1	0.283711	2.61E-09	54585
CTNNB1	0.283668	2.63E-09	1499
CPT1C	0.283604	2.65E-09	126129
PPP2R5E	0.283588	2.65E-09	5529
TNFSF8	0.283499	2.69E-09	944
ZNF2	0.283472	2.69E-09	7549
ARMCX3	0.28347	2.70E-09	51566
IDE	0.283438	2.71E-09	3416
BLVRA	0.283434	2.71E-09	644
GIGYF2	0.283432	2.71E-09	26058
PHF11	0.283405	2.72E-09	51131
STARD13	0.283393	2.72E-09	90627
CDH23	0.283328	2.75E-09	64072
TMEM184C	0.283318	2.75E-09	55751
IL17RA	0.283307	2.75E-09	23765
CWC22	0.283306	2.76E-09	57703
FUBP1	0.283287	2.76E-09	8880
TMTC1	0.283221	2.79E-09	83857
ALG10	0.28321	2.79E-09	84920
AP3M2	0.283156	2.81E-09	10947
FAM98A	0.283147	2.81E-09	25940
KRIT1	0.28312	2.82E-09	889
EDEM1	0.283108	2.83E-09	9695
UHRF1BP1L	0.283094	2.83E-09	23074
ANKRD17	0.283093	2.83E-09	26057
ZNF12	0.283044	2.85E-09	7559
PRPF38B	0.283035	2.86E-09	55119
SMARCA2	0.28301	2.87E-09	6595
FICD	0.282997	2.87E-09	11153
ANXA8L1	0.282986	2.88E-09	728113
KLHDC1	0.282927	2.90E-09	122773
ZNF319	0.282908	2.91E-09	57567
APOBEC3G	0.282898	2.91E-09	60489
TRIM44	0.282896	2.91E-09	54765
MAP9	0.282893	2.91E-09	79884
CLEC12A	0.282818	2.94E-09	160364
HIF1AN	0.282785	2.95E-09	55662
SLC4A11	0.282775	2.96E-09	83959
PZP	0.282735	2.97E-09	5858
SNX3	0.282672	3.00E-09	8724
CTSZ	0.282656	3.00E-09	1522
FBXO42	0.282623	3.02E-09	54455
SPAG9	0.282617	3.02E-09	9043
TMPPE	0.282611	3.02E-09	643853
SARDH	0.282526	3.06E-09	1757

TMED8	0.282492	3.07E-09	283578
LSP1	0.282489	3.07E-09	4046
ESM1	0.282472	3.08E-09	11082
SPRR2F	0.282469	3.08E-09	6705
SPTB	0.282433	3.10E-09	6710
C11orf73	0.282429	3.10E-09	NA
DDI2	0.282372	3.12E-09	84301
HERPUD2	0.282352	3.13E-09	64224
ARAP1	0.282348	3.13E-09	116985
GTF3C4	0.282345	3.13E-09	9329
VCPKMT	0.282333	3.14E-09	79609
ICE2	0.282268	3.16E-09	79664
FAM195B	0.282252	3.17E-09	NA
YPEL5	0.28217	3.21E-09	51646
TSC22D2	0.282167	3.21E-09	9819
CNP	0.282156	3.21E-09	1267
IL20RB	0.282131	3.22E-09	53833
WDR35	0.282113	3.23E-09	57539
SAMD9L	0.282104	3.23E-09	219285
GIMAP6	0.282061	3.25E-09	474344
NUDT4	0.281968	3.29E-09	11163
UBR1	0.281967	3.29E-09	197131
NUDCD1	0.281736	3.40E-09	84955
SLC35E1	0.281698	3.41E-09	79939
IPO11	0.281675	3.42E-09	51194
LRCH1	0.281635	3.44E-09	23143
CNTNAP1	0.281628	3.45E-09	8506
PLSCR1	0.281536	3.49E-09	5359
ARNT	0.281475	3.52E-09	405
USP35	0.281451	3.53E-09	57558
FAM78B	0.281428	3.54E-09	149297
RAD1	0.28139	3.56E-09	5810
TCAF1	0.281389	3.56E-09	9747
FBXW8	0.281313	3.59E-09	26259
TOP2B	0.281298	3.60E-09	7155
KLF8	0.281298	3.60E-09	11279
ZBED8	0.281285	3.61E-09	63920
LYL1	0.281233	3.63E-09	4066
CD109	0.281179	3.66E-09	135228
CHURC1	0.281166	3.66E-09	91612
RAB6A	0.281064	3.71E-09	5870
CHST12	0.281056	3.72E-09	55501
PDE4B	0.281045	3.72E-09	5142
PIGK	0.281043	3.72E-09	10026
CHIT1	0.281005	3.74E-09	1118
YPEL2	0.280991	3.75E-09	388403
LOXL4	0.280987	3.75E-09	84171
YWHAZ	0.280915	3.79E-09	7534
C9orf84	0.280865	3.81E-09	NA
AC090154.1	0.280838	3.83E-09	NA
C17orf51	0.280833	3.83E-09	NA
FAM172A	0.280821	3.83E-09	83989
OR1L8	0.280775	3.86E-09	138881
DNAJC10	0.280677	3.91E-09	54431
RAB3GAP2	0.280669	3.91E-09	25782
CDKL3	0.280666	3.91E-09	51265
EID2B	0.280666	3.91E-09	126272
RAB18	0.280656	3.92E-09	22931
HNRNPLL	0.28064	3.93E-09	92906
GPATCH11	0.280637	3.93E-09	253635

TMTC2	0.280629	3.93E-09	160335
ARRDC5	0.280609	3.94E-09	645432
PGBD1	0.280595	3.95E-09	84547
FBXL4	0.28059	3.95E-09	26235
RAB11FIP5	0.280494	4.00E-09	26056
ZBTB43	0.280439	4.03E-09	23099
FAM133B	0.28041	4.05E-09	257415
KLK5	0.280403	4.05E-09	25818
ZBTB20	0.280398	4.06E-09	26137
CSPP1	0.28037	4.07E-09	79848
FCN1	0.28034	4.09E-09	2219
CSTA	0.280317	4.10E-09	1475
CUL2	0.280279	4.12E-09	8453
SPN	0.28026	4.13E-09	6693
ACTR2	0.280258	4.13E-09	10097
SCRN1	0.28023	4.15E-09	9805
RTL1	0.280181	4.17E-09	388015
NUS1	0.280114	4.21E-09	116150
MTRF1L	0.280113	4.21E-09	54516
SEC24A	0.280093	4.22E-09	10802
CBX1	0.280001	4.27E-09	10951
IL1A	0.279942	4.31E-09	3552
ARL6IP5	0.279895	4.33E-09	10550
NGFR	0.279775	4.40E-09	4804
SERAC1	0.279764	4.41E-09	84947
SIKE1	0.279757	4.41E-09	80143
PSME4	0.279709	4.44E-09	23198
SMC6	0.279709	4.44E-09	79677
YTHDC1	0.279652	4.47E-09	91746
FADS2	0.279613	4.50E-09	9415
MYSM1	0.279608	4.50E-09	114803
PBX2	0.279564	4.53E-09	5089
TCAF2	0.279412	4.62E-09	285966
ANKRD10	0.279387	4.63E-09	55608
SLC38A9	0.279336	4.66E-09	153129
CDKN1B	0.279335	4.67E-09	1027
ZNF610	0.279315	4.68E-09	162963
KIAA0226	0.279314	4.68E-09	NA
PHF10	0.279287	4.69E-09	55274
CXCL16	0.27928	4.70E-09	58191
CYSLTR2	0.279104	4.81E-09	57105
CTSC	0.279031	4.86E-09	1075
BMS1	0.279011	4.87E-09	9790
ZNF621	0.278995	4.88E-09	285268
NECAP2	0.278961	4.90E-09	55707
PP2D1	0.278947	4.91E-09	151649
USP6	0.278936	4.92E-09	9098
CDH8	0.278919	4.93E-09	1006
LYPD2	0.2789	4.94E-09	137797
PMS2	0.278885	4.95E-09	5395
TTC25	0.278847	4.97E-09	83538
RAD54L2	0.278844	4.98E-09	23132
CAPG	0.278823	4.99E-09	822
TRIM38	0.278744	5.04E-09	10475
PXT1	0.278741	5.04E-09	222659
ZNF8	0.278721	5.06E-09	7554
TIMM23B	0.278686	5.08E-09	100652748
PTMS	0.278682	5.08E-09	5763
SCN1B	0.278605	5.13E-09	6324
CCDC109B	0.278601	5.14E-09	NA

PERP	0.278599	5.14E-09	64065
SRFBP1	0.278585	5.15E-09	153443
ZNF639	0.278569	5.16E-09	51193
DNAH10OS	0.278557	5.17E-09	642797
CHRNA3	0.278534	5.18E-09	1136
DIEXF	0.278528	5.19E-09	NA
DOCK7	0.278522	5.19E-09	85440
CLDND1	0.278466	5.23E-09	56650
IRG1	0.278458	5.23E-09	NA
GUCY1A2	0.2784	5.27E-09	2977
SCD	0.278377	5.29E-09	6319
ABLIM3	0.278372	5.29E-09	22885
FAM122A	0.278353	5.31E-09	116224
FGD2	0.278343	5.31E-09	221472
HCAR3	0.278342	5.31E-09	8843
ASB14	0.278332	5.32E-09	142686
TMPRSS11A	0.278284	5.36E-09	339967
PHF2	0.27827	5.36E-09	5253
MSI2	0.278168	5.44E-09	124540
ATP7A	0.27813	5.46E-09	538
DHX29	0.278094	5.49E-09	54505
C15orf41	0.278078	5.50E-09	NA
MYL4	0.278072	5.51E-09	4635
NBAS	0.277995	5.56E-09	51594
CCDC116	0.277974	5.58E-09	164592
SIRT1	0.277918	5.62E-09	23411
SLC9A6	0.277881	5.65E-09	10479
ZHX1	0.27787	5.65E-09	11244
PPP2R2B	0.277862	5.66E-09	5521
NDE1	0.277861	5.66E-09	54820
RTKN2	0.277855	5.66E-09	219790
DNAJB9	0.277839	5.68E-09	4189
C2orf69	0.277837	5.68E-09	205327
GPR107	0.277835	5.68E-09	57720
RP11-302B13.5	0.277831	5.68E-09	NA
GNGT2	0.27783	5.68E-09	2793
SUGT1	0.277806	5.70E-09	10910
EFHD1	0.277764	5.73E-09	80303
ABCE1	0.277756	5.74E-09	6059
EYS	0.277753	5.74E-09	346007
CLCN6	0.277728	5.76E-09	1185
BBS4	0.277725	5.76E-09	585
CLK1	0.277702	5.78E-09	1195
DHX40	0.277691	5.79E-09	79665
ZFP14	0.277669	5.80E-09	57677
CHPF2	0.277641	5.82E-09	54480
TMEM185A	0.277621	5.84E-09	84548
UBTD2	0.277616	5.84E-09	92181
SLC24A4	0.277568	5.88E-09	123041
PFKFB1	0.277566	5.88E-09	5207
MRE11A	0.277548	5.90E-09	NA
PIAS1	0.277497	5.94E-09	8554
PKD2L1	0.277394	6.02E-09	9033
FAM53C	0.277392	6.02E-09	51307
TJP1	0.27738	6.03E-09	7082
DDX18	0.277362	6.04E-09	8886
ZBED6	0.277346	6.05E-09	100381270
SSR3	0.277309	6.08E-09	6747
AOX1	0.277285	6.10E-09	316
NBPF14	0.277262	6.12E-09	25832

PLA2G4C	0.277258	6.12E-09	8605
ATP13A3	0.277207	6.16E-09	79572
SNX27	0.277132	6.23E-09	81609
DYNC1H1	0.277118	6.24E-09	1778
HAUS6	0.277103	6.25E-09	54801
PARD3B	0.277073	6.27E-09	117583
CRYBG3	0.277044	6.30E-09	131544
ZSCAN32	0.276998	6.33E-09	54925
ST7	0.276985	6.35E-09	7982
ZNF221	0.276895	6.42E-09	7638
PLBD2	0.276885	6.43E-09	196463
TRIM39	0.276881	6.43E-09	56658
ZNF260	0.276851	6.46E-09	339324
ZNF92	0.276782	6.52E-09	168374
SETD5	0.276674	6.61E-09	55209
GALNS	0.276663	6.62E-09	2588
ZNF160	0.276629	6.65E-09	90338
GNG10	0.276615	6.66E-09	2790
ATMIN	0.276592	6.68E-09	23300
RNF165	0.276592	6.68E-09	494470
ZBTB24	0.276586	6.68E-09	9841
SMEK2	0.276546	6.72E-09	NA
THUMPD1	0.276509	6.75E-09	55623
TBL1XR1	0.276504	6.76E-09	79718
FBXO28	0.276503	6.76E-09	23219
TVP23C	0.276498	6.76E-09	201158
TRIM32	0.276459	6.80E-09	22954
HDX	0.2764	6.85E-09	139324
PRKCI	0.276375	6.87E-09	5584
TRABD2B	0.276286	6.95E-09	388630
HP	0.276263	6.97E-09	3240
LRCH2	0.276252	6.98E-09	57631
ID4	0.27624	6.99E-09	3400
HAS2	0.276212	7.02E-09	3037
MED23	0.276209	7.02E-09	9439
PDE7A	0.276201	7.03E-09	5150
PWP1	0.276199	7.03E-09	11137
SPATA5	0.276123	7.10E-09	166378
EFCAB6	0.276038	7.18E-09	64800
RP5-1042K10.14	0.276007	7.21E-09	NA
PLEKHH2	0.275973	7.24E-09	130271
PHF3	0.275965	7.25E-09	23469
DNAJA1	0.275951	7.26E-09	3301
SMC3	0.275939	7.27E-09	9126
FAM109B	0.275928	7.28E-09	NA
RBM34	0.275887	7.32E-09	23029
SMG8	0.275874	7.33E-09	55181
NSUN4	0.275866	7.34E-09	387338
DMTF1	0.275852	7.35E-09	9988
SHOC2	0.275839	7.37E-09	8036
GGA2	0.27577	7.43E-09	23062
SUPT6H	0.275766	7.44E-09	6830
RBM27	0.275764	7.44E-09	54439
ZCRB1	0.275759	7.44E-09	85437
RBBP5	0.275745	7.46E-09	5929
ZNF136	0.275682	7.52E-09	7695
EFHC1	0.27566	7.54E-09	114327
ARL3	0.275629	7.57E-09	403
RNMT	0.275624	7.57E-09	8731
INTS2	0.275608	7.59E-09	57508

ERO1LB	0.275558	7.64E-09	NA
FAM204A	0.27552	7.68E-09	63877
SMO	0.275512	7.68E-09	6608
TMEM154	0.275504	7.69E-09	201799
SV2A	0.275485	7.71E-09	9900
ULK4	0.27544	7.76E-09	54986
MIS18BP1	0.275423	7.77E-09	55320
ZNF529	0.27541	7.79E-09	57711
MAPK8	0.275403	7.79E-09	5599
PDLIM7	0.275401	7.80E-09	9260
UBE2V1	0.275344	7.85E-09	7335
FMR1	0.275302	7.90E-09	2332
SCAI	0.27528	7.92E-09	286205
ZNF280C	0.275253	7.95E-09	55609
LSM14A	0.275244	7.96E-09	26065
GPR137C	0.27517	8.03E-09	283554
LACTB	0.275146	8.06E-09	114294
TRAM1L1	0.275101	8.10E-09	133022
MARK3	0.27509	8.12E-09	4140
SLC8A1	0.275036	8.17E-09	6546
EMC2	0.27497	8.24E-09	9694
UBQLN1	0.27496	8.25E-09	29979
RHBDD1	0.274951	8.26E-09	84236
COG5	0.274939	8.28E-09	10466
DUSP19	0.274934	8.28E-09	142679
TENM2	0.274904	8.31E-09	57451
CHD4	0.274829	8.39E-09	1108
MPV17	0.274818	8.41E-09	4358
DUSP11	0.274811	8.41E-09	8446
GDAP2	0.274783	8.44E-09	54834
TTC5	0.274775	8.45E-09	91875
USP48	0.274771	8.46E-09	84196
ZNF506	0.274739	8.49E-09	440515
UFL1	0.274616	8.63E-09	23376
SLC25A36	0.274559	8.69E-09	55186
pk	0.274522	8.73E-09	NA
PRRT3	0.274455	8.81E-09	285368
LMBR1	0.274442	8.82E-09	64327
HVCN1	0.274441	8.83E-09	84329
STARD9	0.274274	9.02E-09	57519
SPINK13	0.274239	9.06E-09	153218
GOPC	0.274204	9.10E-09	57120
UBE2Z	0.27418	9.13E-09	65264
RP11-248J23.6	0.274154	9.16E-09	NA
SLC24A2	0.274143	9.17E-09	25769
HLA-DQB1	0.274128	9.19E-09	3119
HOXC4	0.274111	9.21E-09	3221
POGK	0.274086	9.24E-09	57645
ATG2B	0.274068	9.26E-09	55102
FNDC3A	0.274037	9.30E-09	22862
PLA2G16	0.274029	9.31E-09	NA
RAPGEF3	0.273999	9.34E-09	10411
TAF1A	0.273953	9.40E-09	9015
PCGF6	0.273945	9.41E-09	84108
CREG1	0.27394	9.41E-09	8804
PEX12	0.273909	9.45E-09	5193
PLEKHA5	0.273885	9.48E-09	54477
PANK2	0.273805	9.58E-09	80025
RHD	0.273792	9.59E-09	6007
TMEM206	0.273725	9.68E-09	NA

USP25	0.27369	9.72E-09	29761
ZNF235	0.273689	9.72E-09	9310
SDC3	0.273679	9.74E-09	9672
XPC	0.273674	9.74E-09	7508
GIMAP5	0.273658	9.76E-09	55340
CEP44	0.273621	9.81E-09	80817
RBM14-RBM4	0.273581	9.86E-09	100526737
10-9月	0.273573	9.87E-09	NA
TCTN2	0.273472	1.00E-08	79867
FBXO10	0.273439	1.00E-08	26267
SLC25A32	0.27342	1.01E-08	81034
XRRA1	0.273381	1.01E-08	143570
DPP8	0.273319	1.02E-08	54878
NFIL3	0.273316	1.02E-08	4783
PRMT9	0.273289	1.02E-08	90826
CMTM7	0.273259	1.03E-08	112616
BDNF	0.273245	1.03E-08	627
TAX1BP1	0.273242	1.03E-08	8887
FHAD1	0.273145	1.04E-08	114827
C4orf50	0.273128	1.04E-08	389197
BAG3	0.273118	1.05E-08	9531
SUPT3H	0.273089	1.05E-08	8464
TOPORS	0.273037	1.06E-08	10210
TTC33	0.273032	1.06E-08	23548
GPR34	0.273009	1.06E-08	2857
SPICE1	0.273004	1.06E-08	152185
RSRC1	0.272998	1.06E-08	51319
ZDHHC20	0.272994	1.06E-08	253832
AGGF1	0.272903	1.08E-08	55109
PHF13	0.272873	1.08E-08	148479
ACTR8	0.272856	1.08E-08	93973
VPS35	0.272854	1.08E-08	55737
UBXN2A	0.272851	1.08E-08	165324
F2RL2	0.272848	1.08E-08	2151
ZXDB	0.272819	1.09E-08	158586
GOLGA2	0.272733	1.10E-08	2801
VCL	0.272723	1.10E-08	7414
ABI1	0.272685	1.11E-08	10006
SPINK9	0.272671	1.11E-08	643394
POU5F2	0.27267	1.11E-08	134187
UBXN4	0.272619	1.12E-08	23190
XKR5	0.272597	1.12E-08	389610
RAPGEF2	0.272587	1.12E-08	9693
CDC26	0.272582	1.12E-08	246184
ACSM6	0.272578	1.12E-08	142827
PRKCQ	0.272559	1.12E-08	5588
SP1	0.272512	1.13E-08	6667
TMEM184B	0.272489	1.13E-08	25829
SYNJ1	0.272484	1.13E-08	8867
C19orf66	0.272422	1.14E-08	NA
DCAF4L1	0.272381	1.15E-08	285429
PLEKHA2	0.272299	1.16E-08	59339
DUSP3	0.272258	1.17E-08	1845
MED1	0.272256	1.17E-08	5469
LLGL1	0.272208	1.18E-08	3996
RASGRP4	0.272085	1.19E-08	115727
ZNF250	0.272081	1.19E-08	58500
RNF214	0.272063	1.20E-08	257160
HECA	0.272012	1.21E-08	51696
KCNIP2	0.271993	1.21E-08	30819

KLK7	0.271964	1.21E-08	5650
ZNF543	0.271953	1.21E-08	125919
RSBN1L	0.271946	1.22E-08	222194
LRP6	0.271917	1.22E-08	4040
LPGAT1	0.271908	1.22E-08	9926
SH2D1A	0.271876	1.23E-08	4068
NUCKS1	0.271691	1.26E-08	64710
ELF1	0.271637	1.26E-08	1997
TASP1	0.271627	1.27E-08	55617
C2	0.271626	1.27E-08	717
ARL5B	0.271626	1.27E-08	221079
TUBB3	0.271607	1.27E-08	10381
CAND1	0.271582	1.27E-08	55832
CCSAP	0.271572	1.27E-08	126731
TRIB2	0.271565	1.28E-08	28951
MTMR1	0.271553	1.28E-08	8776
KMT2C	0.271553	1.28E-08	58508
FAM110D	0.271533	1.28E-08	79927
ALPK1	0.271522	1.28E-08	80216
SNX25	0.271512	1.28E-08	83891
AC016549.1	0.271481	1.29E-08	NA
C18orf25	0.271438	1.30E-08	147339
CPD	0.271375	1.31E-08	1362
CIC	0.271268	1.33E-08	23152
CCDC53	0.271262	1.33E-08	NA
HEATR9	0.271247	1.33E-08	256957
ATF1	0.271232	1.33E-08	466
C10orf12	0.271193	1.34E-08	NA
STRN	0.271166	1.34E-08	6801
CHST7	0.27116	1.34E-08	56548
SREK1IP1	0.271133	1.35E-08	285672
IRF2BPL	0.271102	1.35E-08	64207
SEC22A	0.271091	1.36E-08	26984
PTPRK	0.271079	1.36E-08	5796
CDC40	0.271071	1.36E-08	51362
ASPH	0.270995	1.37E-08	444
TCTN3	0.270984	1.37E-08	26123
ZNF746	0.270955	1.38E-08	155061
VMP1	0.270955	1.38E-08	81671
UTP23	0.270928	1.38E-08	84294
ICA1L	0.270892	1.39E-08	130026
CDC42BPA	0.270844	1.40E-08	8476
LPCAT2	0.270825	1.40E-08	54947
DCAF5	0.270819	1.40E-08	8816
PPP1R21	0.270805	1.41E-08	129285
CBWD2	0.270791	1.41E-08	150472
PARG	0.270758	1.41E-08	8505
CXCL11	0.270747	1.42E-08	6373
RHOT1	0.270743	1.42E-08	55288
LRP11	0.270739	1.42E-08	84918
SMOX	0.270724	1.42E-08	54498
IL3RA	0.270713	1.42E-08	3563
ARMCX4	0.270687	1.43E-08	100131755
KIAA0040	0.270653	1.43E-08	9674
CTSD	0.270598	1.44E-08	1509
SGPL1	0.270593	1.44E-08	8879
RAPGEF4	0.27058	1.45E-08	11069
TPBGL	0.270561	1.45E-08	100507050
NFXL1	0.270524	1.46E-08	152518
C11orf65	0.270475	1.47E-08	160140

TDRKH	0.270448	1.47E-08	11022
MON2	0.270443	1.47E-08	23041
USP24	0.27042	1.48E-08	23358
ATF5	0.270382	1.48E-08	22809
WDR33	0.270353	1.49E-08	55339
ANKRD36	0.270274	1.50E-08	375248
PGM2	0.270266	1.51E-08	55276
PPIG	0.270201	1.52E-08	9360
TGIF2	0.270194	1.52E-08	60436
ADAM10	0.270188	1.52E-08	102
RFC1	0.270129	1.53E-08	5981
APOD	0.270116	1.53E-08	347
C11orf57	0.270065	1.54E-08	NA
PIP5K1A	0.270064	1.54E-08	8394
RBSN	0.270063	1.54E-08	64145
MYO18B	0.270045	1.55E-08	84700
TSHR	0.270026	1.55E-08	7253
FAM13C	0.269991	1.56E-08	220965
CTDSPL2	0.269868	1.58E-08	51496
C9orf131	0.269854	1.59E-08	138724
TNFRSF12A	0.269818	1.59E-08	51330
SS18	0.269784	1.60E-08	6760
RBM26	0.269767	1.60E-08	64062
EYA4	0.26975	1.61E-08	2070
KLHDC7B	0.269695	1.62E-08	113730
MCMBP	0.269687	1.62E-08	79892
DICER1	0.26959	1.64E-08	23405
NAB2	0.26957	1.64E-08	4665
TAF1B	0.269554	1.65E-08	9014
CSTF2T	0.269548	1.65E-08	23283
ACAP2	0.269536	1.65E-08	23527
CDK13	0.269486	1.66E-08	8621
SMARCAD1	0.26946	1.67E-08	56916
CAPRIN1	0.269422	1.68E-08	4076
MTHFD1L	0.269391	1.68E-08	25902
NPHP3	0.26939	1.68E-08	27031
SERINC3	0.269373	1.69E-08	10955
GZMK	0.26936	1.69E-08	3003
GYPC	0.269288	1.70E-08	2995
BATF	0.269287	1.70E-08	10538
HOMER1	0.269282	1.71E-08	9456
ATG14	0.269265	1.71E-08	22863
KIAA1279	0.269237	1.71E-08	NA
TRAPPC11	0.269222	1.72E-08	60684
IL15RA	0.269184	1.73E-08	3601
CHRM5	0.269168	1.73E-08	1133
N4BP1	0.269157	1.73E-08	9683
SYNE3	0.269151	1.73E-08	161176
C5orf15	0.269141	1.74E-08	56951
TMEM165	0.269134	1.74E-08	55858
KCNAB1	0.269085	1.75E-08	7881
EXOG	0.269036	1.76E-08	9941
FBXO3	0.268985	1.77E-08	26273
ZBTB37	0.268984	1.77E-08	84614
ZNF518B	0.268897	1.79E-08	85460
KCTD21	0.268888	1.79E-08	283219
LRRTM3	0.268885	1.79E-08	347731
MAP1LC3B	0.268853	1.80E-08	81631
ANP32E	0.268831	1.80E-08	81611
NAA30	0.268812	1.81E-08	122830

SERPINB4	0.268802	1.81E-08	6318
RIC8B	0.26877	1.82E-08	55188
DARS	0.268744	1.82E-08	NA
GATS	0.268731	1.83E-08	NA
LYSMD3	0.268692	1.84E-08	116068
POMK	0.268685	1.84E-08	84197
METTL16	0.268633	1.85E-08	79066
LIPJ	0.268631	1.85E-08	142910
IRAK4	0.268529	1.87E-08	51135
CGNL1	0.268459	1.89E-08	84952
ARAP2	0.268455	1.89E-08	116984
TEX10	0.268433	1.90E-08	54881
APH1B	0.268414	1.90E-08	83464
BTBD10	0.268337	1.92E-08	84280
RGPD8	0.268297	1.93E-08	727851
AKTIP	0.268296	1.93E-08	64400
CRYBA1	0.268284	1.93E-08	1411
SMG6	0.268271	1.94E-08	23293
ZNF71	0.268207	1.95E-08	58491
CPPED1	0.268205	1.95E-08	55313
NUPL1	0.268202	1.95E-08	NA
ANKUB1	0.2682	1.95E-08	389161
HEATR1	0.26818	1.96E-08	55127
INTS8	0.268105	1.98E-08	55656
FADS3	0.268016	2.00E-08	3995
CXCL8	0.268012	2.00E-08	3576
RRAGB	0.268011	2.00E-08	10325
MSL3	0.267976	2.01E-08	10943
PCF11	0.267976	2.01E-08	51585
SMAD1	0.267973	2.01E-08	4086
LSM11	0.267937	2.02E-08	134353
AP1S3	0.267916	2.03E-08	130340
MICAL2	0.267915	2.03E-08	9645
ZNF597	0.267903	2.03E-08	146434
SNRNP27	0.267887	2.03E-08	11017
BAZ1B	0.267823	2.05E-08	9031
FOXN3	0.267823	2.05E-08	1112
SPRR3	0.267784	2.06E-08	6707
ZNF480	0.267779	2.06E-08	147657
APPL1	0.26776	2.07E-08	26060
GIMAP8	0.267752	2.07E-08	155038
RAP1B	0.267746	2.07E-08	5908
PAPOLA	0.267744	2.07E-08	10914
LCA5L	0.267701	2.08E-08	150082
HSP90B1	0.26767	2.09E-08	7184
EIF1AD	0.267665	2.09E-08	84285
EP400NL	0.267635	2.10E-08	NA
VAV1	0.267634	2.10E-08	7409
CEP57	0.267613	2.10E-08	9702
TBC1D15	0.26752	2.13E-08	64786
OTUD6B	0.267512	2.13E-08	51633
FXYD5	0.26751	2.13E-08	53827
GPRASP1	0.267476	2.14E-08	9737
CRTAM	0.267458	2.14E-08	56253
TAF12	0.267406	2.16E-08	6883
ASAH1	0.267322	2.18E-08	427
CYTIP	0.26728	2.19E-08	9595
A1BG	0.267276	2.19E-08	1
NDST1	0.267267	2.20E-08	3340
SETDB2	0.267256	2.20E-08	83852

RTN2	0.267203	2.21E-08	6253
SLCO2B1	0.267122	2.24E-08	11309
AGBL3	0.267111	2.24E-08	340351
TBCCD1	0.267082	2.25E-08	55171
CRLF3	0.26698	2.28E-08	51379
REV1	0.266959	2.28E-08	51455
COL4A3BP	0.266949	2.29E-08	NA
CAPN14	0.266943	2.29E-08	440854
RBM18	0.26694	2.29E-08	92400
AC242988.1	0.266923	2.29E-08	NA
MMP7	0.266853	2.31E-08	4316
SLC39A1	0.266839	2.32E-08	27173
C1QA	0.266802	2.33E-08	712
LOH12CR1	0.266797	2.33E-08	NA
ARR3	0.266756	2.34E-08	407
UBAP1	0.26671	2.36E-08	51271
WDR75	0.266687	2.36E-08	84128
GOLGA8N	0.266669	2.37E-08	643699
RAPGEF6	0.266666	2.37E-08	51735
PPP5D1	0.266648	2.37E-08	100506012
GHRL	0.266628	2.38E-08	51738
MLKL	0.266627	2.38E-08	197259
HEATR6	0.26662	2.38E-08	63897
SLC24A1	0.266614	2.38E-08	9187
KNOP1	0.26661	2.39E-08	400506
HHEX	0.266595	2.39E-08	3087
FAM188B	0.266591	2.39E-08	NA
ANAPC10	0.26655	2.40E-08	10393
C11orf74	0.266549	2.40E-08	NA
NXF1	0.266522	2.41E-08	10482
BRAP	0.266487	2.42E-08	8315
CNFN	0.266446	2.43E-08	84518
MPHOSPH10	0.266444	2.44E-08	10199
DPY19L2	0.266444	2.44E-08	283417
TCF15	0.266439	2.44E-08	6939
CEP63	0.266393	2.45E-08	80254
MFSD12	0.266388	2.45E-08	126321
PCDHA6	0.266318	2.47E-08	56142
BIRC6	0.266266	2.49E-08	57448
LDLRAD3	0.266245	2.50E-08	143458
HSP90AB1	0.266228	2.50E-08	3326
PLTP	0.266226	2.50E-08	5360
FIP1L1	0.266192	2.51E-08	81608
AC253572.1	0.266106	2.54E-08	NA
SP7	0.266091	2.54E-08	121340
RNASE7	0.266037	2.56E-08	84659
TPP2	0.266034	2.56E-08	7174
RB1	0.266021	2.57E-08	5925
PCDHA10	0.266013	2.57E-08	56139
SGMS1	0.266011	2.57E-08	259230
WDR92	0.265998	2.57E-08	116143
SLC9A3R2	0.265959	2.59E-08	9351
EP400	0.265945	2.59E-08	57634
SMIM8	0.265908	2.60E-08	57150
NOLC1	0.265896	2.61E-08	9221
MYLK3	0.265865	2.62E-08	91807
TTLL9	0.265779	2.65E-08	164395
RGCC	0.265757	2.65E-08	28984
ZNF804A	0.265713	2.67E-08	91752
ZNF766	0.265678	2.68E-08	90321

LMO2	0.265676	2.68E-08	4005
CCL22	0.265604	2.70E-08	6367
RAB5A	0.2656	2.71E-08	5868
EIF5B	0.265555	2.72E-08	9669
KIAA1143	0.26553	2.73E-08	57456
DERL1	0.265412	2.77E-08	79139
SNAPC3	0.265389	2.78E-08	6619
C9orf72	0.265376	2.78E-08	203228
GPR87	0.265368	2.78E-08	53836
LIMD2	0.265355	2.79E-08	80774
EDRF1	0.26533	2.80E-08	26098
ZBTB40	0.265322	2.80E-08	9923
FAM227A	0.265275	2.82E-08	646851
PSMD12	0.265178	2.85E-08	5718
MYOZ1	0.265176	2.85E-08	58529
TTYH3	0.265162	2.86E-08	80727
YME1L1	0.265144	2.86E-08	10730
TNKS	0.265138	2.86E-08	8658
UIMC1	0.265096	2.88E-08	51720
PPRC1	0.265082	2.89E-08	23082
RP11-766F14.2	0.265082	2.89E-08	NA
LRRTM2	0.265073	2.89E-08	26045
FOXI2	0.265068	2.89E-08	399823
ZNF674	0.265044	2.90E-08	641339
PPP4R2	0.265027	2.90E-08	151987
HMGXB4	0.265017	2.91E-08	10042
MAML1	0.264989	2.92E-08	9794
TMEM123	0.264975	2.92E-08	114908
CDKL4	0.264965	2.93E-08	344387
USP42	0.264955	2.93E-08	84132
SPDYE5	0.264844	2.97E-08	442590
RP11-136C24.3	0.264837	2.97E-08	NA
BCAS3	0.264819	2.98E-08	54828
DDX3X	0.264814	2.98E-08	1654
RPTN	0.264629	3.05E-08	126638
PPFIA1	0.264626	3.05E-08	8500
ANAPC1	0.264618	3.06E-08	64682
C1QB	0.264579	3.07E-08	713
THAP5	0.264537	3.09E-08	168451
ZFX	0.264526	3.09E-08	7543
CDK12	0.264521	3.09E-08	51755
ZNF852	0.264491	3.10E-08	285346
TTC27	0.264447	3.12E-08	55622
ZNF106	0.264439	3.12E-08	64397
KIF13A	0.264434	3.13E-08	63971
RYR2	0.264425	3.13E-08	6262
TMC1	0.264404	3.14E-08	117531
ELMO1	0.264363	3.15E-08	9844
RBM48	0.26436	3.16E-08	84060
PTPN1	0.26434	3.16E-08	5770
CMTR2	0.264282	3.19E-08	55783
TCEANC2	0.264219	3.21E-08	127428
TAF8	0.264111	3.25E-08	129685
FEZ2	0.264067	3.27E-08	9637
SEC23A	0.264058	3.28E-08	10484
THUMPD2	0.264015	3.29E-08	80745
LRFN5	0.264007	3.30E-08	145581
CDK19	0.263996	3.30E-08	23097
PTPRB	0.263896	3.34E-08	5787
ZNF708	0.263888	3.34E-08	7562

MEGF10	0.263791	3.39E-08	84466
MBTPS2	0.26379	3.39E-08	51360
XPO1	0.263759	3.40E-08	7514
WDR43	0.263692	3.43E-08	23160
RNF113B	0.263679	3.43E-08	140432
SLC25A17	0.26366	3.44E-08	10478
PDIA6	0.263638	3.45E-08	10130
BRWD1	0.2636	3.47E-08	54014
MTF2	0.263577	3.48E-08	22823
GJC1	0.263539	3.49E-08	10052
GEMIN5	0.263539	3.49E-08	25929
DIS3	0.263476	3.52E-08	22894
TEX9	0.263459	3.53E-08	374618
CPSF2	0.263428	3.54E-08	53981
SMARCC1	0.263412	3.55E-08	6599
NFIX	0.263319	3.59E-08	4784
METTL9	0.2633	3.60E-08	51108
DRAXIN	0.263291	3.60E-08	374946
HNRNPU	0.263262	3.61E-08	3192
THAP6	0.263257	3.62E-08	152815
UTP11L	0.263218	3.63E-08	NA
PYURF	0.263209	3.64E-08	100996939
ZNF197	0.263196	3.64E-08	10168
TUBD1	0.263164	3.66E-08	51174
COPS2	0.263058	3.71E-08	9318
TXLNA	0.263046	3.71E-08	200081
TMX1	0.263002	3.73E-08	81542
PHF12	0.262963	3.75E-08	57649
IFIH1	0.262922	3.77E-08	64135
FAM89B	0.262894	3.78E-08	23625
NEBL	0.262881	3.79E-08	10529
ARMC3	0.262867	3.79E-08	219681
CLEC12B	0.262827	3.81E-08	387837
TLR7	0.262821	3.82E-08	51284
FBXW7	0.262795	3.83E-08	55294
SPRYD3	0.262792	3.83E-08	84926
POLR3GL	0.262672	3.89E-08	84265
DGKH	0.262666	3.89E-08	160851
DYNLT3	0.262663	3.89E-08	6990
TP53BP1	0.262658	3.89E-08	7158
HSPH1	0.26264	3.90E-08	10808
VAMP7	0.262597	3.92E-08	6845
MAN2B1	0.262507	3.97E-08	4125
TP53BP2	0.262469	3.98E-08	7159
IMPG1	0.26242	4.01E-08	3617
HAS1	0.262414	4.01E-08	3036
ARMC8	0.262412	4.01E-08	25852
ARHGAP12	0.262393	4.02E-08	94134
PDCD11	0.262392	4.02E-08	22984
HMBOX1	0.262325	4.06E-08	79618
UFM1	0.262313	4.06E-08	51569
CC2D2B	0.262279	4.08E-08	387707
MORC2	0.262273	4.08E-08	22880
HMGN4	0.262236	4.10E-08	10473
CAPN7	0.262194	4.12E-08	23473
IL20RA	0.262179	4.13E-08	53832
ZNF619	0.262127	4.15E-08	285267
AHCTF1	0.262123	4.16E-08	25909
ETV6	0.26211	4.16E-08	2120
NAP1L5	0.262089	4.17E-08	266812

CDV3	0.262074	4.18E-08	55573
PRR3	0.262069	4.18E-08	80742
RAD51D	0.262033	4.20E-08	5892
SH3TC2	0.262	4.22E-08	79628
GPR135	0.261922	4.26E-08	64582
S100A4	0.26192	4.26E-08	6275
ZNF564	0.261907	4.27E-08	163050
RNF166	0.261895	4.27E-08	115992
ENC1	0.261867	4.29E-08	8507
LDLRAD2	0.261805	4.32E-08	401944
CCDC7	0.261787	4.33E-08	79741
HLA-DRB1	0.261706	4.37E-08	3123
KBTBD6	0.26155	4.46E-08	89890
ZNF230	0.26155	4.46E-08	7773
EIF2S1	0.261535	4.47E-08	1965
PDE6C	0.261531	4.47E-08	5146
NAA16	0.261515	4.48E-08	79612
SUMO4	0.261441	4.52E-08	387082
PREPL	0.26144	4.52E-08	9581
ACLY	0.261373	4.56E-08	47
PELI3	0.261359	4.56E-08	246330
ABCA9	0.261358	4.56E-08	10350
DCLK2	0.261349	4.57E-08	166614
PAN3	0.261336	4.58E-08	255967
UBE2E3	0.261314	4.59E-08	10477
KPNA7	0.261297	4.60E-08	402569
PYHIN1	0.261277	4.61E-08	149628
METTL21A	0.261248	4.63E-08	151194
CTTNBP2NL	0.261245	4.63E-08	55917
LDHAL6A	0.2612	4.65E-08	160287
PSMC1	0.261196	4.66E-08	5700
HLA-DRA	0.261179	4.67E-08	3122
MB21D1	0.261106	4.71E-08	NA
CHURC1-FNTB	0.26106	4.73E-08	100529261
STXBP5	0.261054	4.74E-08	134957
ACSS3	0.261033	4.75E-08	79611
PMS1	0.260926	4.81E-08	5378
DHX8	0.260906	4.82E-08	1659
EAF1	0.260873	4.84E-08	85403
ANO3	0.260867	4.85E-08	63982
TNFAIP8	0.260845	4.86E-08	25816
MAPKAPK2	0.260812	4.88E-08	9261
SLC36A4	0.260807	4.88E-08	120103
RLIM	0.260783	4.90E-08	51132
XPA	0.260744	4.92E-08	7507
TRIM62	0.260738	4.92E-08	55223
SUPT7L	0.26068	4.96E-08	9913
SLC29A1	0.260657	4.97E-08	2030
CD274	0.260595	5.01E-08	29126
IL6	0.260583	5.02E-08	3569
ZNF474	0.260581	5.02E-08	133923
OLFML3	0.260578	5.02E-08	56944
ZNF326	0.260571	5.02E-08	284695
VKORC1L1	0.260565	5.03E-08	154807
ZNF827	0.260547	5.04E-08	152485
NFIB	0.260523	5.05E-08	4781
SMARCAL1	0.260481	5.08E-08	50485
REC114	0.260462	5.09E-08	283677
SLC2A12	0.260447	5.10E-08	154091
CRKL	0.260428	5.11E-08	1399

ZXDA	0.260427	5.11E-08	7789
ZNF582	0.26042	5.12E-08	147948
ALMS1	0.26042	5.12E-08	7840
SNRNP48	0.260379	5.14E-08	154007
TTLL5	0.260365	5.15E-08	23093
CHST2	0.260334	5.17E-08	9435
DMXL2	0.26032	5.18E-08	23312
KIF20B	0.260309	5.19E-08	9585
TMEM237	0.260304	5.19E-08	65062
RP11-690P14.4	0.260299	5.19E-08	NA
JAG1	0.260296	5.20E-08	182
LRRC8E	0.260288	5.20E-08	80131
FANCM	0.26027	5.21E-08	57697
ZNF445	0.260248	5.23E-08	353274
USP11	0.260213	5.25E-08	8237
SYT15	0.260168	5.28E-08	83849
TDRD3	0.2601	5.32E-08	81550
RBPMS	0.260082	5.33E-08	11030
LGALS8	0.26008	5.33E-08	3964
CEPT1	0.260028	5.37E-08	10390
C1GALT1	0.26001	5.38E-08	56913
RP5-966M1.6	0.260003	5.38E-08	NA
ZNF184	0.259977	5.40E-08	7738
KSR1	0.259972	5.41E-08	8844
CHD3	0.259881	5.46E-08	1107
CSNK1G1	0.259879	5.47E-08	53944
FUBP3	0.25986	5.48E-08	8939
RGS12	0.259842	5.49E-08	6002
TMEM104	0.259838	5.49E-08	54868
IQCG	0.25982	5.51E-08	84223
TUBB	0.259743	5.56E-08	203068
IRX6	0.259705	5.58E-08	79190
EML3	0.259651	5.62E-08	256364
CELF2	0.259642	5.63E-08	10659
A2ML1	0.259594	5.66E-08	144568
ERCC8	0.259585	5.67E-08	1161
PRIM2	0.259584	5.67E-08	5558
WDR53	0.259576	5.67E-08	348793
PPP1R2	0.259561	5.68E-08	5504
MTURN	0.25956	5.68E-08	222166
SDE2	0.25955	5.69E-08	163859
ASNSD1	0.259548	5.69E-08	54529
SUZ12	0.259547	5.69E-08	23512
ARPC2	0.259514	5.71E-08	10109
RYBP	0.259507	5.72E-08	23429
EIF3A	0.259477	5.74E-08	8661
DNAJC3	0.259434	5.77E-08	5611
C16orf45	0.259418	5.78E-08	NA
ENOX2	0.259358	5.82E-08	10495
C3orf38	0.259354	5.83E-08	285237
DTX3	0.259312	5.86E-08	196403
ATRN	0.259263	5.89E-08	8455
DDX5	0.259212	5.93E-08	1655
DCLRE1C	0.259189	5.94E-08	64421
GZMH	0.259173	5.95E-08	2999
PKD1	0.259124	5.99E-08	5310
TSPAN2	0.259115	6.00E-08	10100
BMP2K	0.259101	6.01E-08	55589
WRN	0.259024	6.06E-08	7486
CD99	0.259023	6.06E-08	4267

PHAX	0.259009	6.08E-08	51808
ZBTB32	0.258975	6.10E-08	27033
PUS3	0.258973	6.10E-08	83480
ABHD4	0.258942	6.12E-08	63874
NAA15	0.25894	6.13E-08	80155
ZNF100	0.25894	6.13E-08	163227
GID4	0.258912	6.15E-08	79018
ZNF573	0.258896	6.16E-08	126231
ULK1	0.258853	6.19E-08	8408
THOC5	0.258846	6.20E-08	8563
TBC1D32	0.258796	6.23E-08	221322
API5	0.258795	6.23E-08	8539
CCNY	0.25876	6.26E-08	219771
ZDBF2	0.258741	6.27E-08	57683
C12orf60	0.25874	6.28E-08	144608
MPHOSPH9	0.258708	6.30E-08	10198
ZNF562	0.258689	6.31E-08	54811
STRN3	0.25868	6.32E-08	29966
GAFA3	0.258645	6.35E-08	NA
WWOX	0.258643	6.35E-08	51741
KDM4D	0.25863	6.36E-08	55693
LCOR	0.258625	6.36E-08	84458
ELMOD2	0.258618	6.37E-08	255520
SPARCL1	0.258576	6.40E-08	8404
TTC26	0.258527	6.44E-08	79989
ST13	0.258513	6.45E-08	6767
SYPL1	0.258487	6.47E-08	6856
IFNAR1	0.258432	6.51E-08	3454
DNAJC13	0.258404	6.54E-08	23317
GXYLT1	0.258375	6.56E-08	283464
FAM92A1	0.258366	6.57E-08	NA
MTMR7	0.258356	6.57E-08	9108
CUTC	0.258329	6.59E-08	51076
CR1	0.25832	6.60E-08	1378
SH3GL1	0.258308	6.61E-08	6455
ZBTB14	0.258243	6.66E-08	7541
UBE4A	0.258223	6.68E-08	9354
ZNF585A	0.258208	6.69E-08	199704
NCK1	0.258189	6.71E-08	4690
PSMD7	0.258175	6.72E-08	5713
AP000866.1	0.258133	6.75E-08	NA
CEP83	0.258131	6.75E-08	51134
FAF2	0.258131	6.75E-08	23197
ENY2	0.258109	6.77E-08	56943
NUDT13	0.258092	6.79E-08	25961
RNASEH1	0.258084	6.79E-08	246243
ZSCAN21	0.258059	6.81E-08	7589
AMPD2	0.258045	6.83E-08	271
DIRC3	0.258021	6.84E-08	729582
NTRK1	0.257985	6.87E-08	4914
PRKDC	0.257976	6.88E-08	5591
ZNF518A	0.257948	6.91E-08	9849
SLU7	0.257921	6.93E-08	10569
TTC14	0.257893	6.95E-08	151613
RGS8	0.257888	6.96E-08	85397
UBAP2L	0.257886	6.96E-08	9898
SEC24B	0.257753	7.07E-08	10427
ARMCX6	0.257741	7.08E-08	54470
NATD1	0.25773	7.09E-08	256302
C7orf55-LUC7L2	0.257712	7.10E-08	NA

ZNF800	0.257709	7.11E-08	168850
PTAR1	0.257588	7.21E-08	375743
SLC33A1	0.257528	7.26E-08	9197
AMMECR1	0.257528	7.26E-08	9949
EXOC8	0.257526	7.27E-08	149371
TMEM255A	0.257501	7.29E-08	55026
SECISBP2L	0.257487	7.30E-08	9728
SLC25A53	0.257467	7.32E-08	401612
ST7L	0.257442	7.34E-08	54879
SLCO3A1	0.257429	7.35E-08	28232
IQGAP1	0.25738	7.39E-08	8826
PGF	0.257363	7.41E-08	5228
TMEM167B	0.25736	7.41E-08	56900
SNX9	0.257354	7.42E-08	51429
PPP4R1	0.25733	7.44E-08	9989
FAM186B	0.257326	7.44E-08	84070
HINT3	0.25732	7.45E-08	135114
CCDC92	0.257303	7.46E-08	80212
RNF219	0.257289	7.48E-08	NA
SEC22B	0.257276	7.49E-08	9554
SUMO2	0.257269	7.49E-08	6613
CCDC173	0.25726	7.50E-08	129881
TARSL2	0.257249	7.51E-08	NA
CCDC127	0.257229	7.53E-08	133957
SEMA4D	0.257202	7.55E-08	10507
SPTAN1	0.257172	7.58E-08	6709
PAIP2	0.257153	7.60E-08	51247
FOXP1	0.25708	7.67E-08	27086
DVL3	0.257048	7.69E-08	1857
PRPF40A	0.257001	7.74E-08	55660
TBC1D20	0.256993	7.75E-08	128637
SLC18A2	0.256965	7.77E-08	6571
SALL4	0.256903	7.83E-08	57167
IFNK	0.256889	7.84E-08	56832
BST2	0.256849	7.88E-08	684
KAT7	0.25684	7.89E-08	11143
HNRNPA1L2	0.25684	7.89E-08	144983
CWC27	0.256826	7.90E-08	10283
FKBP1A	0.256749	7.98E-08	2280
FAM89A	0.256745	7.98E-08	375061
STAG3	0.256741	7.98E-08	10734
FOXO3	0.256737	7.99E-08	2309
E2F6	0.256707	8.02E-08	1876
TCTN1	0.256701	8.02E-08	79600
NPR1	0.256669	8.05E-08	4881
SIX5	0.256655	8.07E-08	147912
RELA	0.25662	8.10E-08	5970
NKAPL	0.256605	8.12E-08	222698
GNL1	0.256582	8.14E-08	2794
UFSP2	0.256566	8.15E-08	55325
NAF1	0.256544	8.17E-08	92345
FAM126B	0.256531	8.19E-08	285172
PI15	0.256511	8.21E-08	51050
PDZD9	0.256474	8.24E-08	255762
GOLGA5	0.25647	8.25E-08	9950
C4orf26	0.256459	8.26E-08	NA
CCZ1	0.256427	8.29E-08	51622
EXOC1	0.256418	8.30E-08	55763
DGCR14	0.256367	8.35E-08	NA
NINJ1	0.256321	8.40E-08	4814

NRD1	0.256314	8.40E-08	NA
EPDR1	0.256254	8.46E-08	54749
SOGA3	0.256223	8.50E-08	387104
TBC1D8	0.256204	8.51E-08	11138
C10orf2	0.256191	8.53E-08	NA
PRSS37	0.256155	8.56E-08	136242
ZKSCAN1	0.256138	8.58E-08	7586
TRPM7	0.256059	8.66E-08	54822
DLEU1	0.256021	8.70E-08	10301
TET2	0.255996	8.73E-08	54790
MAP2K1	0.255957	8.77E-08	5604
ERLEC1	0.255873	8.86E-08	27248
MED6	0.255841	8.89E-08	10001
POT1	0.255803	8.93E-08	25913
POLH	0.255707	9.03E-08	5429
ZNF623	0.255666	9.08E-08	9831
AC010642.1	0.255665	9.08E-08	NA
C1orf109	0.25564	9.11E-08	54955
RBM4B	0.255609	9.14E-08	83759
LRIG1	0.255513	9.25E-08	26018
RAD23B	0.255511	9.25E-08	5887
OTOA	0.255464	9.30E-08	146183
C14orf28	0.255459	9.31E-08	122525
CDS2	0.255452	9.31E-08	8760
MB21D2	0.25532	9.46E-08	151963
YY2	0.255297	9.49E-08	404281
HIATL1	0.255293	9.49E-08	NA
N4BP3	0.255254	9.54E-08	23138
NAA50	0.255231	9.56E-08	80218
GGPS1	0.255221	9.57E-08	9453
ZSCAN22	0.255157	9.65E-08	342945
MPHOSPH8	0.255152	9.65E-08	54737
FCHO2	0.255102	9.71E-08	115548
C6	0.25509	9.73E-08	729
THRAP3	0.255083	9.73E-08	9967
ZMAT2	0.255019	9.81E-08	153527
CCDC62	0.255017	9.81E-08	84660
SRSF11	0.254943	9.90E-08	9295
TWF1	0.254937	9.90E-08	5756
SETD2	0.254926	9.92E-08	29072
MLANA	0.254903	9.94E-08	2315
BFAR	0.254876	9.98E-08	51283
RAB10	0.254873	9.98E-08	10890
XG	0.254796	1.01E-07	7499
TRMT44	0.254787	1.01E-07	152992
NBPF10	0.254773	1.01E-07	100132406
C6orf89	0.254724	1.02E-07	221477
TRIM56	0.254689	1.02E-07	81844
CNTN4	0.254683	1.02E-07	152330
ZNF302	0.254615	1.03E-07	55900
DMWD	0.254578	1.03E-07	1762
FER1L5	0.254523	1.04E-07	90342
AHI1	0.254522	1.04E-07	54806
DPP9	0.254516	1.04E-07	91039
FRMD5	0.25451	1.04E-07	84978
PKN1	0.254507	1.04E-07	5585
SLC35F6	0.25448	1.05E-07	54978
HMG20A	0.254471	1.05E-07	10363
XRCC5	0.254461	1.05E-07	7520
KIAA0753	0.254438	1.05E-07	9851

RDH11	0.254422	1.05E-07	51109
LACC1	0.254369	1.06E-07	144811
ZNF611	0.254332	1.06E-07	81856
BRD4	0.254331	1.06E-07	23476
COPA	0.254289	1.07E-07	1314
BAIAP2	0.254282	1.07E-07	10458
REEP5	0.25428	1.07E-07	7905
SPRR1A	0.254268	1.07E-07	6698
ZNF398	0.254216	1.08E-07	57541
ZNF540	0.254187	1.08E-07	163255
MMS22L	0.254127	1.09E-07	253714
GAN	0.25412	1.09E-07	8139
GTPBP4	0.254102	1.09E-07	23560
BTBD7	0.254018	1.10E-07	55727
ZNF117	0.253992	1.11E-07	51351
UGGT2	0.253981	1.11E-07	55757
ZNF853	0.253944	1.11E-07	54753
ORAI3	0.253936	1.12E-07	93129
STAT5A	0.253917	1.12E-07	6776
IPO9	0.253914	1.12E-07	55705
C6orf1	0.253904	1.12E-07 NA	
LPAR6	0.253886	1.12E-07	10161
UBN2	0.253884	1.12E-07	254048
PGR	0.253876	1.12E-07	5241
SNX19	0.253846	1.13E-07	399979
ATR	0.253834	1.13E-07	545
RALGAPB	0.253823	1.13E-07	57148
GPIHBP1	0.253816	1.13E-07	338328
PORCN	0.253795	1.13E-07	64840
U2SURP	0.253771	1.14E-07	23350
PCDHB14	0.253759	1.14E-07	56122
ZCCHC6	0.253731	1.14E-07 NA	
ZNF678	0.253693	1.15E-07	339500
TATDN1	0.253624	1.16E-07	83940
MET	0.253605	1.16E-07	4233
EDN1	0.253547	1.17E-07	1906
TRMT10B	0.253537	1.17E-07	158234
C1QC	0.253526	1.17E-07	714
RHOA	0.253488	1.18E-07	387
KIF7	0.253473	1.18E-07	374654
SMIM3	0.25342	1.19E-07	85027
ZNF878	0.253369	1.19E-07	729747
PEX3	0.253334	1.20E-07	8504
PPIC	0.25333	1.20E-07	5480
UBD	0.253305	1.20E-07	10537
ZNF275	0.2533	1.20E-07	10838
TTN	0.253176	1.22E-07	7273
C17orf49	0.253138	1.23E-07	124944
ZACN	0.253097	1.23E-07	353174
PDGFD	0.253096	1.23E-07	80310
C9orf41	0.253089	1.23E-07 NA	
SC5D	0.253079	1.23E-07	6309
CAPNS2	0.253048	1.24E-07	84290
IL12B	0.253037	1.24E-07	3593
CBWD5	0.253031	1.24E-07	220869
SLAIN2	0.25302	1.24E-07	57606
TMEM182	0.252991	1.25E-07	130827
TEAD2	0.252914	1.26E-07	8463
KLHDC8B	0.2529	1.26E-07	200942
NUP50	0.252891	1.26E-07	10762

COA1	0.252838	1.27E-07	55744
C8orf37	0.252831	1.27E-07	157657
DNAJC14	0.252827	1.27E-07	85406
CENPP	0.252826	1.27E-07	401541
HNRNPH3	0.252718	1.29E-07	3189
FAM71D	0.252704	1.29E-07	161142
SLC26A5	0.252688	1.29E-07	375611
PHGR1	-0.25268	1.29E-07	644844
C4orf46	0.252641	1.30E-07	201725
DPF2	0.252639	1.30E-07	5977
KDM1A	0.252633	1.30E-07	23028
KCTD18	0.252622	1.30E-07	130535
GRB2	0.252605	1.31E-07	2885
SH3GLB1	0.252602	1.31E-07	51100
RND3	0.2526	1.31E-07	390
GTF3C1	0.252564	1.31E-07	2975
SERTAD2	0.252529	1.32E-07	9792
TFCP2	0.252474	1.33E-07	7024
ZNF791	0.252449	1.33E-07	163049
ZNF23	0.252425	1.33E-07	7571
DHX9	0.252371	1.34E-07	1660
FUT10	0.252356	1.34E-07	84750
FAM81B	0.252295	1.35E-07	153643
MBTD1	0.252287	1.36E-07	54799
SENP5	0.252259	1.36E-07	205564
RNF11	0.252251	1.36E-07	26994
GCFC2	0.252251	1.36E-07	6936
CCDC28B	0.252244	1.36E-07	79140
SNIP1	0.252209	1.37E-07	79753
ITGAL	0.252195	1.37E-07	3683
TMA16	0.252194	1.37E-07	55319
ZNF680	0.252127	1.38E-07	340252
C6orf163	0.252078	1.39E-07	206412
SRP72	0.252013	1.40E-07	6731
KEL	0.251993	1.40E-07	3792
E2F3	0.251926	1.41E-07	1871
TRRAP	0.251907	1.42E-07	8295
ZNF652	0.251862	1.42E-07	22834
KATNA1	0.25186	1.43E-07	11104
COBLL1	0.251852	1.43E-07	22837
RP11-295P9.3	0.251848	1.43E-07 NA	
TAF6	0.251828	1.43E-07	6878
XIAP	0.251799	1.44E-07	331
SPDL1	0.251797	1.44E-07	54908
LA16c-306E5.2	0.251695	1.45E-07 NA	
NMUR1	0.251677	1.46E-07	10316
PAPD4	0.251669	1.46E-07 NA	
UBN1	0.251636	1.46E-07	29855
C9orf78	0.251622	1.47E-07	51759
USP4	0.25162	1.47E-07	7375
ELL	0.251617	1.47E-07	8178
JADE1	0.251599	1.47E-07	79960
ABHD8	0.251586	1.47E-07	79575
USP45	0.251552	1.48E-07	85015
THRSP	0.251481	1.49E-07	7069
MAK	0.251467	1.49E-07	4117
SFPQ	0.251438	1.50E-07	6421
UBR3	0.251434	1.50E-07	130507
OSGEPL1	0.251417	1.50E-07	64172
MMGT1	0.251376	1.51E-07	93380

TTC8	0.251367	1.51E-07	123016
CDYL	0.251359	1.51E-07	9425
FAM69C	0.251352	1.51E-07	NA
TRMT5	0.251347	1.51E-07	57570
EIF4G2	0.251339	1.51E-07	1982
EIF4B	0.251337	1.52E-07	1975
KATNBL1	0.251332	1.52E-07	79768
C17orf80	0.251328	1.52E-07	55028
RIOK1	0.25132	1.52E-07	83732
GPBP1	0.251317	1.52E-07	65056
CBLL1	0.2513	1.52E-07	79872
RBL2	0.251296	1.52E-07	5934
ING1	0.251244	1.53E-07	3621
HUWE1	0.251244	1.53E-07	10075
AKIRIN2	0.251228	1.53E-07	55122
PCDHGC4	0.251158	1.55E-07	56098
PKP4	0.251151	1.55E-07	8502
PAPD5	0.251145	1.55E-07	NA
RBM8A	0.251134	1.55E-07	9939
ZNF559-ZNF177	0.251114	1.56E-07	100529215
GPR174	0.251107	1.56E-07	84636
DENR	0.251087	1.56E-07	8562
ATAD5	0.251076	1.56E-07	79915
KHDRBS1	0.251048	1.57E-07	10657
ATP6V0A2	0.251045	1.57E-07	23545
C11orf68	0.251039	1.57E-07	83638
C17orf104	0.250971	1.58E-07	NA
C1D	0.25093	1.59E-07	10438
ID1	-0.25089	1.60E-07	3397
AL139333.1	0.250875	1.60E-07	NA
RNASE6	0.25087	1.60E-07	6039
CFAP36	0.25081	1.61E-07	112942
SRCAP	0.250773	1.62E-07	10847
FZD3	0.250773	1.62E-07	7976
RAG1	0.250728	1.63E-07	5896
C2orf76	0.250698	1.63E-07	130355
MSL2	0.250683	1.64E-07	55167
MED15	0.25067	1.64E-07	51586
KMT2D	0.250528	1.67E-07	8085
APP	0.250522	1.67E-07	351
VAMP5	0.250499	1.67E-07	10791
LRRC40	0.250495	1.67E-07	55631
ZCWPW2	0.250473	1.68E-07	152098
FAM35A	0.250425	1.69E-07	NA
NUP210L	0.250405	1.69E-07	91181
RAB28	0.250399	1.69E-07	9364
PRRC2B	0.250397	1.69E-07	84726
GPR146	0.250374	1.70E-07	115330
SAMD11	0.250362	1.70E-07	148398
AAK1	0.250342	1.70E-07	22848
UTP20	0.250315	1.71E-07	27340
MORF4L2	0.250229	1.72E-07	9643
ASCC3	0.250224	1.73E-07	10973
NUP62	0.250211	1.73E-07	23636
GVQW1	0.250194	1.73E-07	NA
STAG1	0.250175	1.74E-07	10274
ITK	0.250121	1.75E-07	3702
IQCH	0.250071	1.76E-07	64799
PPIP5K2	0.250056	1.76E-07	23262
UEVLD	0.250034	1.76E-07	55293

KCNH7	0.250009	1.77E-07	90134
VPRBP	0.249968	1.78E-07	NA
EPHB4	0.249939	1.78E-07	2050
TECPR2	0.249929	1.79E-07	9895
ZNF846	0.249883	1.80E-07	162993
CELSR1	0.249831	1.81E-07	9620
ZFP69	0.249827	1.81E-07	339559
IFNG	0.249813	1.81E-07	3458
MFN1	0.24981	1.81E-07	55669
SCFD1	0.249765	1.82E-07	23256
ADCYAP1	0.24967	1.84E-07	116
KIF2A	0.249568	1.86E-07	3796
ZNF547	0.24955	1.87E-07	284306
ZNF268	0.249531	1.87E-07	10795
WDR48	0.249528	1.87E-07	57599
FNTA	0.249522	1.87E-07	2339
ZNF462	0.249505	1.88E-07	58499
SMG9	0.249491	1.88E-07	56006
LRRC4	0.249487	1.88E-07	64101
IQCK	0.249486	1.88E-07	124152
NEDD4	0.24947	1.88E-07	4734
OSTC	0.249458	1.89E-07	58505
TMEM78	0.249425	1.89E-07	677790
RBM7	0.249414	1.90E-07	10179
SPCS2	0.249399	1.90E-07	9789
PIBF1	0.249399	1.90E-07	10464
ARMC10	0.249388	1.90E-07	83787
AFF1	0.249387	1.90E-07	4299
IMMP1L	0.249338	1.91E-07	196294
RAB5C	0.249326	1.92E-07	5878
DNAJB7	0.249315	1.92E-07	150353
DCTN4	0.249308	1.92E-07	51164
THOC2	0.249292	1.92E-07	57187
STAT3	0.249287	1.92E-07	6774
ITCH	0.249279	1.93E-07	83737
HAUS2	0.249212	1.94E-07	55142
EIF3E	0.249204	1.94E-07	3646
SUN1	0.249146	1.96E-07	23353
ARL8A	0.249114	1.96E-07	127829
ZMYM1	0.249073	1.97E-07	79830
SRP54	0.249038	1.98E-07	6729
ACTRT3	0.249033	1.98E-07	84517
BCORL1	0.249019	1.99E-07	63035
WHSC1L1	0.249012	1.99E-07	NA
PCDHGA5	0.248962	2.00E-07	56110
FSD1L	0.248952	2.00E-07	83856
ISCA1	0.248949	2.00E-07	81689
LRRC69	0.248947	2.00E-07	100130742
BCOR	0.248929	2.01E-07	54880
APOL6	0.248912	2.01E-07	80830
ABI3	0.248892	2.02E-07	51225
ZNF391	0.248834	2.03E-07	346157
SHQ1	0.248832	2.03E-07	55164
BRD3	0.248827	2.03E-07	8019
PPP3R1	0.248789	2.04E-07	5534
PELI1	0.248787	2.04E-07	57162
SEMA3G	0.248735	2.05E-07	56920
RP11-11N7.5	0.248711	2.06E-07	NA
HSPA5	0.248703	2.06E-07	3309
MCL1	0.248646	2.07E-07	4170

PLK2	0.248633	2.08E-07	10769
C19orf12	0.248599	2.08E-07	83636
HECTD4	0.248568	2.09E-07	283450
DAPK3	0.248543	2.10E-07	1613
FAM20B	0.248531	2.10E-07	9917
RNF20	0.248518	2.10E-07	56254
IL1B	0.248514	2.11E-07	3553
ZKSCAN3	0.24847	2.12E-07	80317
FAT1	0.248457	2.12E-07	2195
RPE	0.248446	2.12E-07	6120
ZNF248	0.248442	2.12E-07	57209
MED20	0.248441	2.12E-07	9477
SMAP2	0.248434	2.12E-07	64744
RCVRN	0.248428	2.13E-07	5957
CD28	0.248357	2.14E-07	940
CCNT2	0.248344	2.15E-07	905
PRSS53	0.248342	2.15E-07	339105
SLC25A46	0.248303	2.16E-07	91137
DAPP1	0.2483	2.16E-07	27071
ZFC3H1	0.248266	2.17E-07	196441
TMEM91	0.248257	2.17E-07	641649
PAQR7	0.248254	2.17E-07	164091
ZNF599	0.248234	2.17E-07	148103
DHFRL1	0.248227	2.18E-07 NA	
PDLIM3	0.248212	2.18E-07	27295
WWP1	0.248172	2.19E-07	11059
CLDN1	0.248146	2.20E-07	9076
NUP160	0.248143	2.20E-07	23279
N4BP2L2	0.248141	2.20E-07	10443
CEBPZ	0.248121	2.20E-07	10153
ALG13	0.248098	2.21E-07	79868
METTL15	0.248086	2.21E-07	196074
VIPAS39	0.248086	2.21E-07	63894
MDC1	0.248063	2.22E-07	9656
RPL7L1	0.247924	2.25E-07	285855
CREBZF	0.247906	2.26E-07	58487
EPS15L1	0.247906	2.26E-07	58513
CKLF	0.247897	2.26E-07	51192
EXOC6B	0.24788	2.27E-07	23233
GTPBP8	0.247828	2.28E-07	29083
TBX21	0.247813	2.28E-07	30009
ZNF277	0.247764	2.30E-07	11179
MBD4	0.247755	2.30E-07	8930
PLAA	0.247736	2.30E-07	9373
SLC16A10	0.247731	2.30E-07	117247
PDS5A	0.247631	2.33E-07	23244
FBXL5	0.247602	2.34E-07	26234
MEF2BNB-MEF2B	0.247578	2.35E-07 NA	
GLCCI1	0.247567	2.35E-07	113263
ZNF81	0.247546	2.35E-07	347344
SERPINB8	0.247535	2.36E-07	5271
WIZ	0.247496	2.37E-07	58525
GRK5	0.247472	2.37E-07	2869
FAM19A2	0.247466	2.38E-07 NA	
NR2F1	0.247465	2.38E-07	7025
HECTD1	0.247464	2.38E-07	25831
QTRTD1	0.247421	2.39E-07 NA	
CCDC181	0.247412	2.39E-07	57821
CCDC134	0.247407	2.39E-07	79879
CD200R1	0.247401	2.39E-07	131450

EIF4ENIF1	0.247399	2.39E-07	56478
CEP89	0.247397	2.40E-07	84902
UFD1L	0.247371	2.40E-07	NA
SSB	0.247369	2.40E-07	6741
IBTK	0.24736	2.41E-07	25998
GOSR2	0.247304	2.42E-07	9570
LCLAT1	0.247303	2.42E-07	253558
TRUB1	0.247276	2.43E-07	142940
LHX6	0.247266	2.43E-07	26468
ZNF222	0.247211	2.45E-07	7673
FBXO21	0.247152	2.46E-07	23014
RARS2	0.24712	2.47E-07	57038
ADPGK	0.247082	2.48E-07	83440
HNRNPC	0.247031	2.50E-07	3183
ZNF671	0.247015	2.50E-07	79891
ANKRD2	0.247015	2.50E-07	26287
POFUT2	0.246999	2.51E-07	23275
LARP7	0.24697	2.52E-07	51574
ZNF567	0.246931	2.53E-07	163081
RIMS1	0.246904	2.54E-07	22999
GPR52	0.246863	2.55E-07	9293
KIAA0922	0.246849	2.55E-07	NA
TNNI3K	0.246839	2.55E-07	51086
BET1	0.246811	2.56E-07	10282
ZNF280B	0.246774	2.57E-07	140883
ULK2	0.246738	2.58E-07	9706
IPO8	0.24672	2.59E-07	10526
PCDHGB5	0.246707	2.59E-07	56101
BAZ2A	0.246696	2.60E-07	11176
NUDT21	0.246685	2.60E-07	11051
DNAJC27	0.246656	2.61E-07	51277
SAP30	0.246612	2.62E-07	8819
CCT6A	0.246601	2.63E-07	908
CUL7	0.246578	2.63E-07	9820
DCC	0.246572	2.63E-07	1630
ALG11	0.246546	2.64E-07	440138
SFSWAP	0.246529	2.65E-07	6433
SPON1	0.246518	2.65E-07	10418
ZKSCAN4	0.246512	2.65E-07	387032
STT3A	0.246494	2.66E-07	3703
ZFP57	0.246488	2.66E-07	346171
TCEA1	0.246428	2.68E-07	6917
ZSCAN12	0.24637	2.70E-07	9753
ZNF665	0.246362	2.70E-07	79788
PEX13	0.246351	2.70E-07	5194
WDR89	0.246343	2.70E-07	112840
TLR4	0.246332	2.71E-07	7099
KLHL41	0.24632	2.71E-07	10324
TMEM241	0.246309	2.71E-07	85019
TGM3	0.246286	2.72E-07	7053
TYW3	0.246269	2.73E-07	127253
NUDT11	0.24625	2.73E-07	55190
LTA4H	0.246243	2.73E-07	4048
ZNF80	0.246202	2.75E-07	7634
PDE8B	0.246161	2.76E-07	8622
PRDM10	0.246121	2.77E-07	56980
TRMT1L	0.246047	2.80E-07	81627
RAB11FIP3	0.246037	2.80E-07	9727
UXS1	0.245939	2.83E-07	80146
ZC3H15	0.245857	2.86E-07	55854

TTPAL	0.245853	2.86E-07	79183
UBA3	0.245847	2.86E-07	9039
TPD52L2	0.245819	2.87E-07	7165
MBTPS1	0.245807	2.88E-07	8720
SET	0.245788	2.88E-07	6418
CD81	0.245772	2.89E-07	975
DDX10	0.245767	2.89E-07	1662
CBX5	0.245764	2.89E-07	23468
MIER2	0.245674	2.92E-07	54531
CADPS2	0.245654	2.93E-07	93664
KMT2E	0.24563	2.93E-07	55904
MDM4	0.245568	2.95E-07	4194
EXOC4	0.245537	2.97E-07	60412
ZMAT5	0.245516	2.97E-07	55954
ENO4	0.245447	3.00E-07	387712
QRICH1	0.245437	3.00E-07	54870
FAM217B	0.245433	3.00E-07	63939
PCDHGA6	0.245406	3.01E-07	56109
SPIDR	0.245379	3.02E-07	23514
ZNF318	0.245379	3.02E-07	24149
PTPRQ	0.245371	3.02E-07	374462
AQP1	0.245348	3.03E-07	358
CTD-2574D22.6	0.245333	3.04E-07	NA
JADE2	0.245294	3.05E-07	23338
ANXA2	0.245249	3.06E-07	302
IFT46	0.245211	3.08E-07	56912
ZFYVE1	0.245194	3.08E-07	53349
ECT2L	0.245168	3.09E-07	345930
DCLRE1B	0.245146	3.10E-07	64858
MAPK1	0.245112	3.11E-07	5594
AGO1	0.245105	3.12E-07	26523
TM2D3	0.245083	3.12E-07	80213
TLL1	0.245063	3.13E-07	7092
TMEM158	0.245008	3.15E-07	25907
LLPH	0.244974	3.16E-07	84298
GIN1	0.24494	3.17E-07	54826
LIMCH1	0.244934	3.18E-07	22998
XPO4	0.244915	3.18E-07	64328
PIGM	0.244886	3.19E-07	93183
ARHGEF1	0.244881	3.20E-07	9138
ARG1	0.244877	3.20E-07	383
RAPGEF1	0.244873	3.20E-07	2889
ASF1A	0.244865	3.20E-07	25842
SMC4	0.244851	3.21E-07	10051
CRIPT	0.244836	3.21E-07	9419
SIX1	0.244757	3.24E-07	6495
SLC22A3	0.244749	3.24E-07	6581
HEATR5B	0.244733	3.25E-07	54497
LCAT	0.244697	3.26E-07	3931
LYRM2	0.244678	3.27E-07	57226
GTPBP10	0.244672	3.27E-07	85865
ZNF550	0.244668	3.27E-07	162972
CBX3	0.244665	3.28E-07	11335
EID2	0.244662	3.28E-07	163126
C5orf28	0.244655	3.28E-07	NA
CCDC67	0.244612	3.30E-07	NA
B4GALT5	0.244606	3.30E-07	9334
CFAP61	0.244511	3.33E-07	26074
RNF144B	0.244503	3.34E-07	255488
SGCB	0.24449	3.34E-07	6443

DCTN5	0.244436	3.36E-07	84516
GALNT10	0.244408	3.37E-07	55568
VPS26A	0.244402	3.38E-07	9559
SIK3	0.244387	3.38E-07	23387
UTP6	0.244373	3.39E-07	55813
PTGS2	0.244371	3.39E-07	5743
MPHOSPH6	0.244299	3.41E-07	10200
HFE	0.244296	3.42E-07	3077
LRRC46	0.244295	3.42E-07	90506
ZFHX2	0.244286	3.42E-07	85446
CALCOCO1	0.244257	3.43E-07	57658
RTN4	0.24425	3.43E-07	57142
PPP2R3C	0.24423	3.44E-07	55012
TNFRSF1B	0.244229	3.44E-07	7133
RCOR3	0.244187	3.46E-07	55758
EOMES	0.244186	3.46E-07	8320
KAT6B	0.244174	3.46E-07	23522
NR2C2	0.244166	3.47E-07	7182
HNRNPK	0.244157	3.47E-07	3190
TERF2	0.244107	3.49E-07	7014
CCDC40	0.244069	3.51E-07	55036
ZNF616	0.244041	3.52E-07	90317
KBTBD7	0.244031	3.52E-07	84078
HIATL2	0.243998	3.53E-07	NA
ARL15	0.243995	3.54E-07	54622
KCND1	0.243987	3.54E-07	3750
UBE2V2	0.243983	3.54E-07	7336
ZNF35	0.24396	3.55E-07	7584
RBAK-RBAKDN	0.243952	3.55E-07	100533952
ECD	0.243952	3.55E-07	11319
PCBP3	0.24391	3.57E-07	54039
PAWR	0.2439	3.57E-07	5074
KIAA0391	0.243867	3.59E-07	NA
COTL1	0.243854	3.59E-07	23406
KIAA1407	0.243841	3.60E-07	NA
FAM209B	0.243819	3.61E-07	388799
ZNF836	0.24381	3.61E-07	162962
GOLGA6L4	0.243801	3.61E-07	643707
MRPS10	0.243751	3.63E-07	55173
RDH10	0.243741	3.64E-07	157506
GLRA3	0.243738	3.64E-07	8001
CBWD1	0.243731	3.64E-07	55871
ZBTB8B	0.243697	3.66E-07	728116
ETV5	0.243641	3.68E-07	2119
EFCAB13	0.243566	3.71E-07	124989
ZC3H7B	0.243565	3.71E-07	23264
GLUL	0.243543	3.72E-07	2752
PIP5K1C	0.243517	3.73E-07	23396
ZMPSTE24	0.243453	3.76E-07	10269
PLIN2	0.243446	3.76E-07	123
SPTBN1	0.243443	3.76E-07	6711
SPOP	0.24344	3.76E-07	8405
SART3	0.24341	3.78E-07	9733
SEC24C	0.243409	3.78E-07	9632
ANKRD13C	0.243376	3.79E-07	81573
FERMT3	0.243363	3.80E-07	83706
ACTBL2	0.243358	3.80E-07	345651
SF3A3	0.243332	3.81E-07	10946
KIAA1715	0.243289	3.83E-07	NA
TAF1	0.243281	3.83E-07	6872

ANKRD28	0.243226	3.86E-07	23243
METAP2	0.243212	3.86E-07	10988
ZNF589	0.243168	3.88E-07	51385
CTBS	0.243163	3.88E-07	1486
GBP2	0.243158	3.89E-07	2634
LTN1	0.243147	3.89E-07	26046
BTBD11	0.243117	3.90E-07	121551
R3HDM1	0.24309	3.92E-07	23518
CCDC43	0.243019	3.95E-07	124808
TIFA	0.243016	3.95E-07	92610
CEP41	0.243012	3.95E-07	95681
KIAA1841	0.242968	3.97E-07	84542
SPATA13	0.242923	3.99E-07	221178
SNTB1	0.24288	4.01E-07	6641
CFHR3	0.242872	4.01E-07	10878
SYDE2	0.242832	4.03E-07	84144
DOPEY1	0.242828	4.03E-07 NA	
PCGF5	0.242793	4.05E-07	84333
ARHGEF7	0.24275	4.07E-07	8874
BRD2	0.242716	4.09E-07	6046
TMEM140	0.242707	4.09E-07	55281
RP11-545J16.1	0.242707	4.09E-07 NA	
HLA-DPA1	0.242688	4.10E-07	3113
OPRL1	0.242679	4.10E-07	4987
PXYLP1	0.242669	4.11E-07	92370
FAM76A	0.242659	4.11E-07	199870
DNAJB11	0.24264	4.12E-07	51726
TACC1	0.242626	4.13E-07	6867
GALNT5	0.242578	4.15E-07	11227
RWDD1	0.242573	4.15E-07	51389
CDADC1	0.242557	4.16E-07	81602
NKTR	0.242534	4.17E-07	4820
ALOX12	0.242508	4.18E-07	239
MYLK4	0.242478	4.20E-07	340156
SPOPL	0.242471	4.20E-07	339745
SYNE2	0.242468	4.20E-07	23224
SLC10A5	0.242424	4.22E-07	347051
RIMBP3	0.242375	4.25E-07	85376
HNRNPH2	0.242326	4.27E-07	3188
KDM2B	0.24231	4.28E-07	84678
PSIP1	0.242299	4.28E-07	11168
KCNJ1	0.242241	4.31E-07	3758
ZSCAN26	0.242158	4.35E-07	7741
ANAPC13	0.242147	4.36E-07	25847
CLHC1	0.242125	4.37E-07	130162
RWDD4	0.242116	4.37E-07	201965
ZNF496	0.242113	4.37E-07	84838
COPS7A	0.242104	4.38E-07	50813
NCOA7	0.242077	4.39E-07	135112
SPIRE1	0.242059	4.40E-07	56907
H3F3B	0.24203	4.41E-07 NA	
SSX2IP	0.242023	4.42E-07	117178
SAMD12	0.242013	4.42E-07	401474
DYRK1A	0.241989	4.43E-07	1859
LARP1	0.241987	4.44E-07	23367
RNF168	0.241951	4.45E-07	165918
AHNAK	0.241938	4.46E-07	79026
ZBED5	0.241908	4.48E-07	58486
CEP295	0.241879	4.49E-07	85459
PTK7	0.241865	4.50E-07	5754

CSNK1A1	0.241865	4.50E-07	1452
EED	0.241863	4.50E-07	8726
STXBP3	0.241859	4.50E-07	6814
PIK3CD	0.241858	4.50E-07	5293
RALA	0.241845	4.51E-07	5898
NFIA	0.241829	4.52E-07	4774
RP11-571M6.15	0.241797	4.53E-07	NA
DMXL1	0.241792	4.53E-07	1657
TAF9B	0.241779	4.54E-07	51616
VPS54	0.241778	4.54E-07	51542
AC008686.1	0.241751	4.56E-07	NA
LDOC1L	0.241717	4.57E-07	NA
CCRN4L	0.2417	4.58E-07	NA
ZNF557	0.24169	4.59E-07	79230
ZNF512	0.241671	4.60E-07	84450
IWS1	0.24167	4.60E-07	55677
LONP2	0.241651	4.61E-07	83752
ATP6V1G1	0.241612	4.63E-07	9550
ZMYND15	0.241596	4.64E-07	84225
ZNF565	0.241587	4.64E-07	147929
FAM104A	0.241536	4.67E-07	84923
TRIM37	0.241483	4.69E-07	4591
KIAA1919	0.241416	4.73E-07	NA
BTN3A1	0.241413	4.73E-07	11119
TRPV5	0.241413	4.73E-07	56302
FAM210B	0.241393	4.74E-07	116151
PDC	0.241387	4.75E-07	5132
GALNT18	0.241386	4.75E-07	374378
PPP6R3	0.241372	4.75E-07	55291
MAPKAP1	0.24135	4.77E-07	79109
FOXJ3	0.241347	4.77E-07	22887
PRTG	0.241341	4.77E-07	283659
CAMLG	0.241339	4.77E-07	819
FAM199X	0.241337	4.77E-07	139231
YY1AP1	0.241332	4.77E-07	55249
TAL1	0.241318	4.78E-07	6886
ZNF354A	0.241314	4.78E-07	6940
ANKRD65	0.24129	4.80E-07	441869
ZNF778	0.241282	4.80E-07	197320
ZNF140	0.241269	4.81E-07	7699
ATL2	0.241254	4.82E-07	64225
CCDC79	0.241247	4.82E-07	NA
SIGLEC15	0.241233	4.83E-07	284266
POLN	0.241233	4.83E-07	353497
MAP3K5	0.241224	4.83E-07	4217
XPNPEP3	0.241215	4.84E-07	63929
FAM102B	0.241186	4.85E-07	284611
GALNT11	0.241148	4.87E-07	63917
PUS10	0.241141	4.88E-07	150962
CCDC65	0.241111	4.89E-07	85478
STX4	0.241103	4.90E-07	6810
SRBD1	0.241091	4.91E-07	55133
EXT2	0.241065	4.92E-07	2132
SNX13	0.240999	4.96E-07	23161
DCP1A	0.240975	4.97E-07	55802
POLR3A	0.240972	4.97E-07	11128
FNBP4	0.240969	4.97E-07	23360
ZNF792	0.24094	4.99E-07	126375
ATXN7	0.240931	4.99E-07	6314
METTL14	0.240927	5.00E-07	57721

CST6	0.240916	5.00E-07	1474
RSPO1	0.240907	5.01E-07	284654
AC069063.2	0.240869	5.03E-07	NA
FGD3	0.240868	5.03E-07	89846
METTL10	0.240801	5.07E-07	NA
ZNF134	0.240717	5.12E-07	7693
ZW10	0.240713	5.12E-07	9183
RRAGA	0.240664	5.15E-07	10670
ZC3H8	0.24054	5.22E-07	84524
PLIN4	0.240532	5.22E-07	729359
CACNB4	0.240526	5.23E-07	785
LPIN1	0.240525	5.23E-07	23175
LRRC55	0.240495	5.24E-07	219527
TTC4	0.240491	5.25E-07	7268
DENND3	0.240448	5.27E-07	22898
ARPC1B	0.240447	5.27E-07	10095
DDX42	0.240439	5.28E-07	11325
PPWD1	0.240437	5.28E-07	23398
RANBP6	0.240422	5.29E-07	26953
MMS19	0.240401	5.30E-07	64210
ABTB2	0.240396	5.30E-07	25841
ZNF559	0.240332	5.34E-07	84527
ARHGEF10	0.240289	5.37E-07	9639
ZNF30	0.240264	5.38E-07	90075
TBC1D16	0.240237	5.40E-07	125058
SUSD6	0.240223	5.41E-07	9766
DAXX	0.240187	5.43E-07	1616
EIF4E	0.240155	5.45E-07	1977
RBM15	0.240102	5.48E-07	64783
ZNF253	0.240056	5.51E-07	56242
LRIG2	0.24002	5.53E-07	9860
REPS1	0.240017	5.53E-07	85021
SLTM	0.239971	5.56E-07	79811
HAUS3	0.239965	5.56E-07	79441
ZNF493	0.239904	5.60E-07	284443
ZNF317	0.239849	5.64E-07	57693
NIP7	0.239839	5.64E-07	51388
HP1BP3	0.239815	5.66E-07	50809
TBKBP1	0.239776	5.68E-07	9755
DIXDC1	0.23977	5.69E-07	85458
RHEB	0.239767	5.69E-07	6009
PNISR	0.239759	5.69E-07	25957
FBXL3	0.239739	5.71E-07	26224
ZNF670	0.239725	5.72E-07	93474
ACTR10	0.239706	5.73E-07	55860
HSP90AA1	0.239677	5.75E-07	3320
MMP17	0.239672	5.75E-07	4326
BROX	0.239662	5.76E-07	148362
NEU3	0.239593	5.80E-07	10825
MRFAP1L1	0.239567	5.82E-07	114932
JRKL	0.239532	5.84E-07	8690
SHROOM4	0.239513	5.85E-07	57477
DNAH5	0.239459	5.89E-07	1767
SRPR	0.239455	5.89E-07	NA
RCL1	0.239422	5.91E-07	10171
FAM161A	0.239421	5.91E-07	84140
NMT1	0.239378	5.94E-07	4836
CLLU1OS	0.239352	5.96E-07	NA
C6orf106	0.239312	5.98E-07	NA
BCL9	0.239271	6.01E-07	607

RBP1	0.239241	6.03E-07	5947
SLC22A17	0.239191	6.06E-07	51310
MICU1	0.239157	6.09E-07	10367
SHOX2	0.2391	6.13E-07	6474
TRMT11	0.23907	6.15E-07	60487
C19orf71	0.239064	6.15E-07	100128569
INTS3	0.238989	6.20E-07	65123
USP13	0.23897	6.22E-07	8975
STAM2	0.238947	6.23E-07	10254
UBA5	0.238938	6.24E-07	79876
DTHD1	0.238908	6.26E-07	401124
TMEM185B	0.238882	6.28E-07	79134
DNTTIP2	0.238861	6.29E-07	30836
EML4	0.238853	6.30E-07	27436
ZNF788	0.238845	6.30E-07	NA
MAGED2	0.238837	6.31E-07	10916
ASCL1	0.238807	6.33E-07	429
TMCO3	0.238806	6.33E-07	55002
BTN3A2	0.238791	6.34E-07	11118
PDZRN3	0.238759	6.36E-07	23024
ARFGEF1	0.238716	6.39E-07	10565
TMEM57	0.238688	6.41E-07	NA
EVA1C	0.238666	6.43E-07	59271
CUX1	0.238664	6.43E-07	1523
POLR2B	0.238629	6.46E-07	5431
BTN2A1	0.238625	6.46E-07	11120
CD300LG	0.238618	6.46E-07	146894
TMEM51	0.238596	6.48E-07	55092
PLD2	0.238594	6.48E-07	5338
COQ10A	0.238584	6.49E-07	93058
NES	0.23858	6.49E-07	10763
TNNI2	0.238573	6.50E-07	7136
IKBKAP	0.23856	6.51E-07	NA
ZSWIM8	0.238537	6.52E-07	23053
MAU2	0.238527	6.53E-07	23383
RCN1	0.238526	6.53E-07	5954
ARHGEF28	0.238505	6.55E-07	64283
MKRN2	0.238503	6.55E-07	23609
CSTF3	0.23848	6.56E-07	1479
UGGT1	0.238466	6.57E-07	56886
TM2D2	0.238456	6.58E-07	83877
KLHL22	0.238435	6.60E-07	84861
CCDC148	0.238413	6.61E-07	130940
MEOX1	0.238405	6.62E-07	4222
USP8	0.238405	6.62E-07	9101
FFAR3	0.23839	6.63E-07	2865
IL2RB	0.238365	6.65E-07	3560
AGPS	0.23835	6.66E-07	8540
PPP1R13L	0.238348	6.66E-07	10848
RAB2A	0.238341	6.66E-07	5862
GABPB2	0.238323	6.68E-07	126626
ZNF717	0.238305	6.69E-07	100131827
C7orf49	0.238282	6.71E-07	NA
ZNF347	0.238279	6.71E-07	84671
ZSCAN30	0.238268	6.72E-07	100101467
NT5C1B	0.238256	6.73E-07	93034
PLAG1	0.238218	6.76E-07	5324
THUMPD3	0.238151	6.81E-07	25917
BTBD1	0.23815	6.81E-07	53339
SRSF1	0.23814	6.82E-07	6426

ZNF329	0.238124	6.83E-07	79673
SQLE	0.238108	6.84E-07	6713
VAC14	0.238094	6.85E-07	55697
NDUFAF7	0.238081	6.86E-07	55471
MSANTD2	0.238061	6.87E-07	79684
EPM2AIP1	0.238047	6.89E-07	9852
ZNF587B	0.238005	6.92E-07	100293516
DLG1	0.238004	6.92E-07	1739
TRMT61B	0.237995	6.93E-07	55006
ZNF181	0.237989	6.93E-07	339318
RIN3	0.237984	6.93E-07	79890
MYBL1	0.237957	6.95E-07	4603
HELZ2	0.237933	6.97E-07	85441
COPB1	0.237909	6.99E-07	1315
SYCE1	0.237868	7.02E-07	93426
HTR1F	0.237848	7.04E-07	3355
SRP19	0.237845	7.04E-07	6728
OR2D2	0.237841	7.04E-07	120776
GPRIN3	0.237807	7.07E-07	285513
DAG1	0.237781	7.09E-07	1605
ZNF107	0.237771	7.10E-07	51427
SMEK1	0.23773	7.13E-07	NA
CFAP57	0.237707	7.15E-07	149465
LIN52	0.237703	7.15E-07	91750
ERCC3	0.23769	7.16E-07	2071
DZANK1	0.237656	7.19E-07	55184
RCAN3	0.237605	7.23E-07	11123
TAB2	0.237579	7.25E-07	23118
TMED2	0.237473	7.34E-07	10959
MSRB3	0.237464	7.34E-07	253827
ZNF124	0.237464	7.34E-07	7678
IKBKG	0.237385	7.41E-07	8517
DEF8	0.237353	7.43E-07	54849
USP28	0.237308	7.47E-07	57646
MYNN	0.237279	7.50E-07	55892
POU2F2	0.237256	7.51E-07	5452
BOK	0.237252	7.52E-07	666
GOLGA4	0.23725	7.52E-07	2803
MAD1L1	0.237247	7.52E-07	8379
GTF2H3	0.237197	7.56E-07	2967
RAB1A	0.237162	7.59E-07	5861
ABCC5	0.237156	7.60E-07	10057
ZNF830	0.237139	7.61E-07	91603
CCL21	0.237126	7.62E-07	6366
NFX1	0.237107	7.64E-07	4799
SLC25A51	0.237068	7.67E-07	92014
WBP5	0.237018	7.71E-07	NA
NUP133	0.237011	7.72E-07	55746
STIP1	0.236993	7.74E-07	10963
ACTR1A	0.23697	7.75E-07	10121
PRPF39	0.236965	7.76E-07	55015
NOX5	0.236944	7.78E-07	79400
RENBP	0.236925	7.79E-07	5973
SPDYE3	0.236881	7.83E-07	441272
ZNF519	0.236871	7.84E-07	162655
ASXL1	0.236868	7.84E-07	171023
VCP	0.236864	7.85E-07	7415
ITPKC	0.236857	7.85E-07	80271
EPT1	0.236823	7.88E-07	NA
ERI1	0.236811	7.89E-07	90459

FTSJ3	0.236778	7.92E-07	117246
ZNF740	0.236774	7.92E-07	283337
PKNOX1	0.236774	7.92E-07	5316
DHX58	0.236724	7.97E-07	79132
SNW1	0.236714	7.98E-07	22938
ADNP	0.236697	7.99E-07	23394
IGFBP6	0.236686	8.00E-07	3489
RAB7A	0.23667	8.02E-07	7879
TAGAP	0.236656	8.03E-07	117289
MTPAP	0.236597	8.08E-07	55149
TGFBRAP1	0.236549	8.12E-07	9392
IFT20	0.236515	8.15E-07	90410
AARS	0.236512	8.16E-07 NA	
MAK16	0.236486	8.18E-07	84549
ZNF782	0.236476	8.19E-07	158431
STAG2	0.236405	8.25E-07	10735
UBR4	0.236392	8.26E-07	23352
PRRG3	0.236339	8.31E-07	79057
SPATA6L	0.236328	8.32E-07	55064
FBXL18	0.236319	8.33E-07	80028
MAPK1IP1L	0.23617	8.47E-07	93487
HPS4	0.23617	8.47E-07	89781
KCNE5	0.23615	8.49E-07	23630
TMEM64	0.236145	8.49E-07	169200
TXLNB	0.236141	8.50E-07	167838
HDAC9	0.236122	8.51E-07	9734
KCNV2	0.236119	8.52E-07	169522
KANSL3	0.236113	8.52E-07	55683
TBX18	0.236094	8.54E-07	9096
AC069063.1	0.236	8.63E-07 NA	
ERVFRD-1	0.23598	8.65E-07	405754
ZNF189	0.235975	8.65E-07	7743
ZNF283	0.235955	8.67E-07	284349
TNFAIP8L1	0.235948	8.68E-07	126282
CRABP2	0.235926	8.70E-07	1382
ANGPTL6	0.235911	8.71E-07	83854
ZNF571	0.23591	8.71E-07	51276
NRSN2	0.235893	8.73E-07	80023
TINF2	0.235878	8.74E-07	26277
C3orf49	0.235878	8.74E-07	132200
SLC35A5	0.235858	8.76E-07	55032
NOL10	0.23583	8.79E-07	79954
DCAF13	0.235819	8.80E-07	25879
MEX3A	0.235802	8.82E-07	92312
DNAJC21	0.2358	8.82E-07	134218
SMARCE1	0.235762	8.86E-07	6605
ERV3-1	0.235742	8.87E-07	2086
UBE2R2	0.235729	8.89E-07	54926
MINA	0.235713	8.90E-07 NA	
TRIM47	0.235691	8.92E-07	91107
ZNF98	0.235683	8.93E-07	148198
DDX47	0.235658	8.96E-07	51202
ATP2A2	0.235641	8.97E-07	488
DUSP22	0.235627	8.99E-07	56940
STAC3	0.235607	9.01E-07	246329
ZBTB39	0.235601	9.01E-07	9880
SYCP3	0.235594	9.02E-07	50511
RBM28	0.235534	9.08E-07	55131
PDCD1	0.235508	9.11E-07	5133
TOP3B	0.235487	9.13E-07	8940

PAK1IP1	0.235391	9.22E-07	55003
TAS2R3	0.235381	9.23E-07	50831
NSL1	0.235358	9.26E-07	25936
EIF3C	0.235335	9.28E-07	8663
KIAA0020	0.235315	9.30E-07 NA	
TLK2	0.235263	9.35E-07	11011
CRHR2	0.235228	9.39E-07	1395
NUP98	0.23522	9.40E-07	4928
CST2	0.235218	9.40E-07	1470
RNPC3	0.235217	9.40E-07	55599
ACTC1	0.235213	9.40E-07	70
TSR1	0.235206	9.41E-07	55720
EIF2AK4	0.235201	9.42E-07	440275
USP33	0.235174	9.44E-07	23032
OTUD7B	0.235118	9.50E-07	56957
UBAP2	0.235067	9.55E-07	55833
GANAB	0.235057	9.56E-07	23193
GMEB1	0.235039	9.58E-07	10691
STK19	0.235023	9.60E-07	8859
ZNF812	0.235009	9.62E-07 NA	
MNAT1	0.23495	9.68E-07	4331
ATP11A	0.234867	9.77E-07	23250
SPDYE1	0.234865	9.77E-07	285955
MROH9	0.234861	9.77E-07	80133
HNRNPH1	0.234789	9.85E-07	3187
LRRC49	0.234789	9.85E-07	54839
PPP1R3D	0.234736	9.91E-07	5509
TXNDC2	0.234735	9.91E-07	84203
SGOL2	0.234722	9.92E-07 NA	
NDN	0.234697	9.95E-07	4692
WDR3	0.234696	9.95E-07	10885
ROR1	0.234694	9.95E-07	4919
MRPL19	0.234686	9.96E-07	9801
SH2D4B	0.234579	1.01E-06	387694
ESF1	0.234565	1.01E-06	51575
ZNF491	0.234562	1.01E-06	126069
FUT8	0.234553	1.01E-06	2530
AP3M1	0.234544	1.01E-06	26985
AK5	0.234539	1.01E-06	26289
SLC30A5	0.234519	1.01E-06	64924
C10orf32	0.234505	1.02E-06 NA	
LRRC8B	0.234483	1.02E-06	23507
AGPAT6	0.234463	1.02E-06 NA	
MTO1	0.234455	1.02E-06	25821
RNF8	0.234432	1.02E-06	9025
ZRANB2	0.234427	1.02E-06	9406
TOP3A	0.234409	1.03E-06	7156
HDC	0.234373	1.03E-06	3067
ADORA1	0.234351	1.03E-06	134
ACSM5	0.2343	1.04E-06	54988
ANKRD11	0.23427	1.04E-06	29123
C1orf116	0.234243	1.05E-06	79098
KIAA1109	0.234216	1.05E-06	84162
SPATS2L	0.234206	1.05E-06	26010
GALNT2	0.234178	1.05E-06	2590
NFKB1	0.234159	1.05E-06	4790
SCN4A	0.234159	1.05E-06	6329
FAM185A	0.234141	1.06E-06	222234
ANLN	0.234131	1.06E-06	54443
ZNF649	0.234127	1.06E-06	65251

DNAJC8	0.2341	1.06E-06	22826
TBC1D9	0.234095	1.06E-06	23158
EGFL7	0.234082	1.06E-06	51162
CCDC186	0.233961	1.08E-06	55088
CHD7	0.233955	1.08E-06	55636
OMG	0.233905	1.08E-06	4974
ZNF182	0.233863	1.09E-06	7569
TVP23A	0.233859	1.09E-06	780776
RNF41	0.233823	1.09E-06	10193
PAFAH1B1	0.233813	1.10E-06	5048
CYP20A1	0.233781	1.10E-06	57404
FAM200A	0.233781	1.10E-06	221786
HSPA12A	0.233735	1.10E-06	259217
ZFAND4	0.233734	1.10E-06	93550
HSPA8	0.233709	1.11E-06	3312
GAB2	0.233709	1.11E-06	9846
GRIK1	0.233665	1.11E-06	2897
UBA2	0.233656	1.11E-06	10054
CSNK2A1	0.233656	1.11E-06	1457
TPST2	0.233619	1.12E-06	8459
TLK1	0.233612	1.12E-06	9874
TBC1D24	0.233589	1.12E-06	57465
LGMN	0.233589	1.12E-06	5641
DNTTIP1	0.233584	1.12E-06	116092
ANGPTL3	0.233572	1.12E-06	27329
ZBTB26	0.233545	1.13E-06	57684
PTPRA	0.233543	1.13E-06	5786
ESYT1	0.233515	1.13E-06	23344
ERO1L	0.233513	1.13E-06	NA
ZKSCAN7	0.233496	1.13E-06	55888
YY1	0.233476	1.14E-06	7528
TM9SF1	0.233456	1.14E-06	10548
ZNF174	0.233443	1.14E-06	7727
BRPF1	0.233421	1.14E-06	7862
LANCL1	0.233417	1.14E-06	10314
SERBP1	0.233382	1.15E-06	26135
NUB1	0.233372	1.15E-06	51667
ZMIZ1	0.233335	1.15E-06	57178
WDR11	0.233334	1.15E-06	55717
SAMD15	0.233285	1.16E-06	161394
C11orf95	0.233266	1.16E-06	65998
HLA-DMB	0.233258	1.16E-06	3109
ZNF528	0.23325	1.16E-06	84436
AL023806.1	0.23325	1.16E-06	NA
MSH6	0.233229	1.17E-06	2956
FKTN	0.233219	1.17E-06	2218
ATP6V1H	0.233199	1.17E-06	51606
SPG21	0.233177	1.17E-06	51324
ZNF549	0.233177	1.17E-06	256051
AL158801.1	0.233164	1.18E-06	NA
DNAJA2	0.233152	1.18E-06	10294
UPF3B	0.233152	1.18E-06	65109
ZNF845	0.233128	1.18E-06	91664
SMC5	0.233113	1.18E-06	23137
ATG3	0.23309	1.18E-06	64422
TMEM87A	0.233027	1.19E-06	25963
ZNF19	0.233026	1.19E-06	7567
C12orf76	0.233018	1.19E-06	400073
ORC3	0.233014	1.19E-06	23595
IFI6	0.232979	1.20E-06	2537

LTV1	0.232954	1.20E-06	84946
TSNAX	0.232946	1.20E-06	7257
NRAS	0.232944	1.20E-06	4893
ASPM	0.232932	1.21E-06	259266
UBE3C	0.232904	1.21E-06	9690
IRF5	0.232885	1.21E-06	3663
RCN2	0.232857	1.21E-06	5955
SIN3A	0.232692	1.24E-06	25942
B3GAT2	0.232639	1.24E-06	135152
KRT7	0.232631	1.24E-06	3855
UBE3A	0.232612	1.25E-06	7337
TEX2	0.232595	1.25E-06	55852
UCHL5	0.232558	1.25E-06	51377
SZT2	0.232528	1.26E-06	23334
PPP1R8	0.232516	1.26E-06	5511
ERRFI1	0.232423	1.27E-06	54206
UBP1	0.23241	1.27E-06	7342
LIG4	0.232408	1.28E-06	3981
EIF4G3	0.232387	1.28E-06	8672
MTMR12	0.232381	1.28E-06	54545
RRAGC	0.232327	1.29E-06	64121
TMED10	0.232289	1.29E-06	10972
TRIM16	0.23226	1.30E-06	10626
EMC1	0.232237	1.30E-06	23065
ZNF24	0.232233	1.30E-06	7572
ISG20L2	0.232227	1.30E-06	81875
TIAM1	0.232166	1.31E-06	7074
SUDS3	0.232126	1.31E-06	64426
ALG9	0.232118	1.32E-06	79796
TIAL1	0.232117	1.32E-06	7073
AP1G1	0.232098	1.32E-06	164
G3BP2	0.232091	1.32E-06	9908
TMEM248	0.232084	1.32E-06	55069
TMEM189	0.232066	1.32E-06	NA
USP14	0.23206	1.32E-06	9097
NEK4	0.232058	1.32E-06	6787
ARHGEF33	0.232018	1.33E-06	100271715
DHX15	0.232017	1.33E-06	1665
TEDDM1	0.23198	1.34E-06	127670
TRAPPC2B	0.231969	1.34E-06	10597
TMEM150A	0.231908	1.35E-06	129303
RAET1L	0.231901	1.35E-06	154064
ANKRD32	0.231886	1.35E-06	NA
DDB1	0.231828	1.36E-06	1642
AVL9	0.231823	1.36E-06	23080
RPGR	0.231786	1.36E-06	6103
GANC	0.23172	1.37E-06	2595
SPEN	0.231719	1.37E-06	23013
SLCO1A2	0.231718	1.37E-06	6579
PPM1K	0.2317	1.38E-06	152926
TSN	0.231661	1.38E-06	7247
FBXO15	0.231574	1.39E-06	201456
ZNF526	0.231523	1.40E-06	116115
PDZD8	0.231517	1.40E-06	118987
PRKAA1	0.231502	1.41E-06	5562
TCF20	0.231446	1.41E-06	6942
IL12RB2	0.231445	1.41E-06	3595
ZNF627	0.231402	1.42E-06	199692
SCYL2	0.231295	1.44E-06	55681
LYG2	0.231277	1.44E-06	254773

BTBD8	0.231249	1.44E-06	284697
DDX59	0.231216	1.45E-06	83479
TUBGCP3	0.231193	1.45E-06	10426
NLRC3	0.231127	1.46E-06	197358
DDX46	0.231121	1.46E-06	9879
TAS2R4	0.231073	1.47E-06	50832
GDI1	0.231048	1.48E-06	2664
PCYT1A	0.231044	1.48E-06	5130
ARHGAP42	0.231014	1.48E-06	143872
KPNB1	0.230999	1.48E-06	3837
RAD21	0.23099	1.49E-06	5885
RAB40A	0.23098	1.49E-06	142684
UPP2	0.230923	1.50E-06	151531
SIRT2	0.230884	1.50E-06	22933
SNX2	0.23088	1.50E-06	6643
CPN2	0.23087	1.50E-06	1370
BCL11B	0.230866	1.51E-06	64919
PRKRIR	0.230862	1.51E-06 NA	
SCGB3A2	0.230847	1.51E-06	117156
FAM114A1	0.230843	1.51E-06	92689
CAMKMT	0.230842	1.51E-06	79823
B4GALT1	0.230802	1.52E-06	2683
CXorf58	0.230798	1.52E-06	254158
PTPN13	0.230733	1.53E-06	5783
CCDC121	0.230717	1.53E-06	79635
GALNT1	0.230706	1.53E-06	2589
ZNF33B	0.230688	1.53E-06	7582
PRPF4	0.230686	1.53E-06	9128
ITPKB	0.230638	1.54E-06	3707
TVP23C-CDRT4	0.23061	1.55E-06	100533496
ZNF41	0.230592	1.55E-06	7592
BBS1	0.230564	1.55E-06	582
ETV3	0.230526	1.56E-06	2117
VPS37C	0.230505	1.56E-06	55048
PCM1	0.230483	1.57E-06	5108
UBTF	0.23048	1.57E-06	7343
ARHGAP18	0.230423	1.58E-06	93663
IRAK1BP1	0.230408	1.58E-06	134728
ITGA10	0.230403	1.58E-06	8515
ANXA2R	0.230379	1.59E-06	389289
XRN2	0.230356	1.59E-06	22803
GLIPR1	0.23035	1.59E-06	11010
ABCC1	0.23034	1.59E-06	4363
FAIM	0.230337	1.59E-06	55179
ZMYM6NB	0.23032	1.60E-06 NA	
DYNC2LI1	0.230311	1.60E-06	51626
DNM1L	0.230282	1.60E-06	10059
CDON	0.230275	1.60E-06	50937
TFAP2A	0.230256	1.61E-06	7020
HNRNPDL	0.230236	1.61E-06	9987
SPTLC1	0.230229	1.61E-06	10558
ATP6V1E1	0.230202	1.62E-06	529
SLX4	0.230187	1.62E-06	84464
TAS2R10	0.230169	1.62E-06	50839
ZNF354B	0.230105	1.63E-06	117608
LTA	0.230078	1.64E-06	4049
SMIM18	0.230064	1.64E-06	100507341
ZFP82	0.230044	1.64E-06	284406
OCSTAMP	0.230033	1.65E-06	128506
VPS37A	0.230022	1.65E-06	137492

GORASP2	0.230013	1.65E-06	26003
NCOA5	0.229962	1.66E-06	57727
PRMT3	0.22995	1.66E-06	10196
PDE6G	0.229947	1.66E-06	5148
DDX60L	0.22994	1.66E-06	91351
GPN1	0.229928	1.66E-06	11321
DHX33	0.229913	1.67E-06	56919
AMMECR1L	0.229896	1.67E-06	83607
SLC10A7	0.229884	1.67E-06	84068
ALS2CR11	0.229869	1.67E-06	NA
CPE	0.229856	1.68E-06	1363
CYB5R4	0.229847	1.68E-06	51167
SUPT5H	0.229838	1.68E-06	6829
TNPO3	0.229833	1.68E-06	23534
HLTF	0.229831	1.68E-06	6596
PITRM1	0.229773	1.69E-06	10531
ZSCAN9	0.229751	1.70E-06	7746
TSPAN5	0.229714	1.70E-06	10098
BAG2	0.229653	1.71E-06	9532
FBXO7	0.229589	1.73E-06	25793
CNPY3	0.229589	1.73E-06	10695
CCDC122	0.229559	1.73E-06	160857
AKAP10	0.229514	1.74E-06	11216
L1CAM	0.229503	1.74E-06	3897
SBNO2	0.229399	1.76E-06	22904
SLC35F5	0.229396	1.76E-06	80255
CABIN1	0.22936	1.77E-06	23523
RBM39	0.229295	1.78E-06	9584
POLR1B	0.229265	1.79E-06	84172
TAF1D	0.229264	1.79E-06	79101
TGFB1I1	0.229245	1.79E-06	7041
PSTPIP2	0.229241	1.79E-06	9050
AP2M1	0.229212	1.80E-06	1173
DRAP1	0.22919	1.80E-06	10589
UBE2E1	0.229187	1.80E-06	7324
VHL	0.229151	1.81E-06	7428
HSF2	0.229147	1.81E-06	3298
SF3B2	0.229139	1.81E-06	10992
ZNF646	0.229097	1.82E-06	9726
VPS45	0.229084	1.82E-06	11311
DNMT3A	0.229073	1.82E-06	1788
CRNKL1	0.229061	1.82E-06	51340
PTH1R	0.229046	1.83E-06	5745
C5orf22	0.229031	1.83E-06	55322
ARHGAP9	0.229008	1.84E-06	64333
SH3BP4	0.228983	1.84E-06	23677
TCEA2	0.228967	1.84E-06	6919
TUBG2	0.22893	1.85E-06	27175
UNC45B	0.228913	1.85E-06	146862
FNBP1L	0.228882	1.86E-06	54874
USP12	0.228875	1.86E-06	219333
STARD3NL	0.22884	1.87E-06	83930
ZNF443	0.228816	1.87E-06	10224
ZDHHC21	0.228784	1.88E-06	340481
MEIS2	0.228776	1.88E-06	4212
ZNF551	0.228767	1.88E-06	90233
SELM	0.228765	1.88E-06	NA
ZNF765	0.228762	1.88E-06	91661
LIG3	0.228746	1.89E-06	3980
COPB2	0.228721	1.89E-06	9276

KLHL29	0.228709	1.89E-06	114818
CD1A	0.228701	1.90E-06	909
ZNF490	0.228677	1.90E-06	57474
BUB3	0.228648	1.91E-06	9184
PSMC2	0.228608	1.91E-06	5701
SLC9A7	0.228507	1.94E-06	84679
RERG	0.228495	1.94E-06	85004
LMAN1	0.228458	1.95E-06	3998
ZFAND1	0.228452	1.95E-06	79752
TBRG1	0.228427	1.95E-06	84897
FAM65C	0.228412	1.96E-06	NA
RBM33	0.22839	1.96E-06	155435
LRRC7	0.228374	1.96E-06	57554
CERS6	0.228341	1.97E-06	253782
CALR	0.228307	1.98E-06	811
RNF170	0.228301	1.98E-06	81790
PKM	0.228287	1.98E-06	5315
TVP23B	0.228284	1.98E-06	51030
PDE12	0.22827	1.98E-06	201626
ZKSCAN2	0.228254	1.99E-06	342357
GORAB	0.22823	1.99E-06	92344
CCZ1B	0.228225	1.99E-06	221960
MARCKS	0.22822	2.00E-06	4082
MFSD8	0.228219	2.00E-06	256471
POM121	0.228218	2.00E-06	9883
GPR111	0.228209	2.00E-06	NA
UBQLN4	0.228187	2.00E-06	56893
DISP1	0.228182	2.00E-06	84976
AKAP8L	0.228168	2.01E-06	26993
COG6	0.228156	2.01E-06	57511
H6PD	0.2281	2.02E-06	9563
ARL17B	0.228093	2.02E-06	100506084
ANXA7	0.228071	2.03E-06	310
SH3TC1	0.228044	2.03E-06	54436
NRIP1	0.227975	2.05E-06	8204
CCNI	0.227963	2.05E-06	10983
HLCS	0.227939	2.06E-06	3141
CDK5R1	0.22791	2.06E-06	8851
TACR1	0.227902	2.06E-06	6869
HELLS	0.227899	2.06E-06	3070
MED12	0.227896	2.06E-06	9968
BBS7	0.227865	2.07E-06	55212
CDH20	0.227842	2.08E-06	28316
VPS39	0.227812	2.08E-06	23339
FHOD1	0.22779	2.09E-06	29109
RP11-849H4.2	0.227764	2.09E-06	NA
YWHAQ	0.227756	2.10E-06	10971
NOM1	0.227748	2.10E-06	64434
CYP26C1	0.227734	2.10E-06	340665
CEP104	0.22769	2.11E-06	9731
PROS1	0.22768	2.11E-06	5627
ATP6V0E1	0.227644	2.12E-06	8992
ZNF34	0.227622	2.13E-06	80778
GNAL	0.227596	2.13E-06	2774
CEACAM6	0.227529	2.15E-06	4680
LONRF1	0.227471	2.16E-06	91694
UNC50	0.22747	2.16E-06	25972
XPO5	0.227439	2.17E-06	57510
PRDM15	0.227434	2.17E-06	63977
ZNF75A	0.227394	2.18E-06	7627

TAF4B	0.227363	2.18E-06	6875
UBE2F	0.227354	2.19E-06	140739
SRRM1	0.227354	2.19E-06	10250
TAOK3	0.227328	2.19E-06	51347
POM121C	0.227324	2.19E-06	100101267
WDR70	0.227318	2.19E-06	55100
ING3	0.227294	2.20E-06	54556
C12orf43	0.227284	2.20E-06	64897
POLR3D	0.227267	2.21E-06	661
NPHP4	0.227243	2.21E-06	261734
ZMYM3	0.227225	2.22E-06	9203
CPSF7	0.227213	2.22E-06	79869
CLDN16	0.227199	2.22E-06	10686
SMU1	0.227098	2.25E-06	55234
HK1	0.227074	2.25E-06	3098
POLR1A	0.227073	2.25E-06	25885
EBAG9	0.227061	2.26E-06	9166
TTC37	0.226989	2.27E-06	9652
DDIT4	0.226985	2.27E-06	54541
DCTD	0.226978	2.27E-06	1635
TRIT1	0.226969	2.28E-06	54802
LA16c-431H6.6	0.226929	2.29E-06	NA
BNIPL	0.226905	2.29E-06	149428
RGPD3	0.226886	2.30E-06	653489
CADM4	0.226862	2.30E-06	199731
CCDC18	0.226819	2.31E-06	343099
KDM1B	0.226775	2.32E-06	221656
RP11-574K11.31	0.22674	2.33E-06	NA
RSBN1	0.226722	2.34E-06	54665
RARG	0.226713	2.34E-06	5916
EXOSC3	0.22671	2.34E-06	51010
GPR75	0.226698	2.34E-06	10936
COQ10B	0.226669	2.35E-06	80219
ZBP1	0.22666	2.35E-06	81030
SRPX2	0.226596	2.37E-06	27286
CCDC15	0.226588	2.37E-06	80071
AGTRAP	0.226578	2.37E-06	57085
TCP11L2	0.226576	2.37E-06	255394
TRA2A	0.226573	2.37E-06	29896
TADA1	0.226557	2.38E-06	117143
AC105009.1	0.226518	2.39E-06	NA
DGKE	0.226516	2.39E-06	8526
CAV1	0.226488	2.40E-06	857
ITGB3	0.226474	2.40E-06	3690
CCDC93	0.226426	2.41E-06	54520
CHD1	0.226422	2.41E-06	1105
CBX6	0.226414	2.41E-06	23466
KLHL6	0.226369	2.43E-06	89857
TRA2B	0.22635	2.43E-06	6434
CEP78	0.226339	2.43E-06	84131
ADAM29	0.226287	2.45E-06	11086
SCARA3	0.226245	2.46E-06	51435
SLC7A6	0.226244	2.46E-06	9057
PTPRU	0.226215	2.46E-06	10076
CTAGE9	0.226198	2.47E-06	643854
YTHDC2	0.226166	2.48E-06	64848
GINM1	0.226158	2.48E-06	116254
LRRC27	0.226142	2.48E-06	80313
SEMA3C	0.226131	2.49E-06	10512
CTCF	0.226101	2.49E-06	10664

PCMTD2	0.22609	2.50E-06	55251
SNCG	0.226075	2.50E-06	6623
ADSS	0.226067	2.50E-06	NA
CDCP2	0.22604	2.51E-06	200008
CD180	0.226033	2.51E-06	4064
TMEM217	0.226003	2.52E-06	221468
CUEDC1	0.226002	2.52E-06	404093
MYO6	0.225986	2.52E-06	4646
ZBTB49	0.225962	2.53E-06	166793
GLIPR1L2	0.225947	2.54E-06	144321
VPS13A	0.225912	2.54E-06	23230
ZNF45	0.225908	2.55E-06	7596
ZCCHC8	0.22585	2.56E-06	55596
RWDD3	0.225838	2.56E-06	25950
URB1	0.225816	2.57E-06	9875
CCDC171	0.225801	2.57E-06	203238
GFM1	0.225784	2.58E-06	85476
PCDHB5	0.225774	2.58E-06	26167
CTR9	0.22577	2.58E-06	9646
POLR2K	0.225761	2.59E-06	5440
PPP2R5C	0.225752	2.59E-06	5527
GPR171	0.225747	2.59E-06	29909
GGNBP2	0.225746	2.59E-06	79893
NUDCD3	0.225719	2.60E-06	23386
ZNF561	0.225688	2.60E-06	93134
TDRD9	0.225655	2.61E-06	122402
JKAMP	0.225579	2.63E-06	51528
ERF	0.225557	2.64E-06	2077
ZNF500	0.225518	2.65E-06	26048
MTRR	0.22548	2.66E-06	4552
MTBP	0.22548	2.66E-06	27085
RHEBL1	0.225478	2.66E-06	121268
FAM120B	0.225476	2.66E-06	84498
TTC17	0.225457	2.67E-06	55761
AC024060.1	0.225439	2.67E-06	NA
TTC21B	0.225438	2.67E-06	79809
SLC25A18	0.225431	2.68E-06	83733
TGS1	0.225416	2.68E-06	96764
EXOC6	0.225303	2.71E-06	54536
GPR89A	0.22529	2.72E-06	653519
C12orf29	0.225268	2.72E-06	91298
TRAFD1	0.225264	2.72E-06	10906
ANKHD1	0.225247	2.73E-06	54882
APC	0.225245	2.73E-06	324
RBM19	0.225234	2.73E-06	9904
RMDN1	0.22522	2.74E-06	51115
EDIL3	0.225217	2.74E-06	10085
RP3-461F17.3	0.225157	2.75E-06	NA
PIK3R3	0.225147	2.76E-06	8503
KLHL15	0.225136	2.76E-06	80311
IFITM3	0.225126	2.76E-06	10410
C1orf52	0.225103	2.77E-06	148423
SLC13A4	0.225083	2.77E-06	26266
ANTXR2	0.225072	2.78E-06	118429
LRRC74A	0.225053	2.78E-06	145497
LAMA3	0.225048	2.78E-06	3909
GPATCH4	0.225015	2.79E-06	54865
HNRNPUL1	0.225002	2.80E-06	11100
FAM150A	0.224919	2.82E-06	NA
GMPS	0.224903	2.83E-06	8833

CBWD3	0.224894	2.83E-06	445571
SELENBP1	-0.22487	2.84E-06	8991
PGLYRP3	0.224854	2.84E-06	114771
TWF2	0.224843	2.85E-06	11344
GNL2	0.224825	2.85E-06	29889
ZNF343	0.224818	2.85E-06	79175
RBBP4	0.224815	2.85E-06	5928
ILK	0.224751	2.87E-06	3611
IRGQ	0.22475	2.87E-06	126298
TMEM5	0.224727	2.88E-06 NA	
GRM7	0.224652	2.90E-06	2917
EPB41L2	0.224594	2.92E-06	2037
GGN	0.224573	2.93E-06	199720
ABCF1	0.224533	2.94E-06	23
MRFAP1	0.22448	2.95E-06	93621
ZNF227	0.224421	2.97E-06	7770
CMIP	0.224409	2.98E-06	80790
WDR7	0.224378	2.99E-06	23335
ITGA9	0.224356	2.99E-06	3680
ZNF345	0.224338	3.00E-06	25850
HDAC2	0.224281	3.02E-06	3066
SCARB2	0.224273	3.02E-06	950
MYEF2	0.224257	3.02E-06	50804
RPP30	0.224212	3.04E-06	10556
XPOT	0.224196	3.04E-06	11260
SLC12A5	0.224187	3.05E-06	57468
CCL3L3	0.22418	3.05E-06	414062
ZNF74	0.224136	3.06E-06	7625
CD38	0.224129	3.06E-06	952
ZFP30	0.224115	3.07E-06	22835
TMEM33	0.22411	3.07E-06	55161
WDR60	0.224056	3.09E-06 NA	
APBB1	0.224048	3.09E-06	322
SLC1A4	0.224027	3.10E-06	6509
KLHDC10	0.223995	3.11E-06	23008
ARL9	0.223995	3.11E-06	132946
SYS1-DBNDD2	0.223928	3.13E-06	767557
S100A3	0.223921	3.13E-06	6274
URB2	0.223895	3.14E-06	9816
SF3A1	0.223865	3.15E-06	10291
H1F0	0.22381	3.17E-06 NA	
RP11-872D17.8	0.223801	3.17E-06 NA	
RNF141	0.223767	3.18E-06	50862
IFIT1	0.223753	3.19E-06	3434
RRP36	0.223712	3.20E-06	88745
CIDEB	0.223688	3.21E-06	27141
WDR27	0.223679	3.21E-06	253769
TFPT	0.223671	3.21E-06	29844
FBXL20	0.223635	3.23E-06	84961
WT1	0.223624	3.23E-06	7490
FARP1	0.223596	3.24E-06	10160
RAPGEF5	0.223518	3.27E-06	9771
PCMT1	0.22351	3.27E-06	5110
RRP8	0.223493	3.27E-06	23378
USP10	0.22349	3.28E-06	9100
SLC22A1	0.223478	3.28E-06	6580
IL10	0.223439	3.29E-06	3586
TOM1	0.223439	3.29E-06	10043
TMEM121	0.223414	3.30E-06	80757
SNAP47	0.223394	3.31E-06	116841

HAS3	0.223373	3.31E-06	3038
PABPC4	0.223373	3.32E-06	8761
SYF2	0.223357	3.32E-06	25949
MAPK14	0.223336	3.33E-06	1432
EIF2B2	0.223335	3.33E-06	8892
WDYHV1	0.223324	3.33E-06	NA
DCLRE1A	0.223323	3.33E-06	9937
RBM45	0.223304	3.34E-06	129831
PHLDA1	0.223301	3.34E-06	22822
MAPK10	0.223269	3.35E-06	5602
USP16	0.22326	3.35E-06	10600
PNMA1	0.223201	3.37E-06	9240
EFCAB10	0.2232	3.37E-06	100130771
ABCD2	0.223167	3.39E-06	225
ANKRD31	0.223149	3.39E-06	256006
ZSCAN25	0.223146	3.39E-06	221785
NSUN3	0.223077	3.42E-06	63899
DEDD	0.22304	3.43E-06	9191
NMI	0.22296	3.46E-06	9111
SEMA3A	0.222946	3.46E-06	10371
GSTA4	0.222941	3.47E-06	2941
IRF2BP2	0.222932	3.47E-06	359948
DCAF7	0.222894	3.48E-06	10238
FNTB	0.222889	3.48E-06	2342
ACTB	0.222887	3.49E-06	60
CTC-454I21.3	0.222874	3.49E-06	NA
NOL11	0.222866	3.49E-06	25926
RNF31	0.22283	3.51E-06	55072
URI1	0.222785	3.52E-06	8725
EIF2A	0.222782	3.52E-06	83939
TOB2	0.222732	3.54E-06	10766
PPM1H	0.222722	3.55E-06	57460
ITGB4	0.222721	3.55E-06	3691
ARMCX5	0.222709	3.55E-06	64860
RMND1	0.222707	3.55E-06	55005
PDE5A	0.222651	3.57E-06	8654
IMMP2L	0.222638	3.58E-06	83943
AQP3	0.222636	3.58E-06	360
STAMBPL1	0.222632	3.58E-06	57559
TRMT12	0.222627	3.58E-06	55039
PLOD2	0.222622	3.58E-06	5352
ATN1	0.222611	3.59E-06	1822
KIAA0556	0.222562	3.60E-06	NA
CD1E	0.222541	3.61E-06	913
MED29	0.222533	3.62E-06	55588
ERICH1	0.222503	3.63E-06	157697
CIR1	0.222501	3.63E-06	9541
RRP12	0.2225	3.63E-06	23223
ZNF22	0.222471	3.64E-06	7570
SEMA4A	0.222448	3.65E-06	64218
STRIP1	0.222434	3.65E-06	85369
UBE2B	0.22242	3.66E-06	7320
LRRN4	0.222395	3.67E-06	164312
ZNF681	0.222389	3.67E-06	148213
UCP3	0.222387	3.67E-06	7352
ZSCAN29	0.222361	3.68E-06	146050
RELB	0.222355	3.68E-06	5971
SRF	0.222338	3.69E-06	6722
GOSR1	0.222332	3.69E-06	9527
ZNF786	0.222295	3.71E-06	136051

NCL	0.222282	3.71E-06	4691
ATG16L1	0.22227	3.72E-06	55054
IL27	0.222261	3.72E-06	246778
TEX29	0.222232	3.73E-06	121793
ZNF251	0.222199	3.74E-06	90987
TMED7	0.222184	3.75E-06	51014
PCGF2	0.222133	3.77E-06	7703
CD52	0.222126	3.77E-06	1043
PSMD6	0.222124	3.77E-06	9861
PCDHB6	0.222122	3.77E-06	56130
MAP3K19	0.222099	3.78E-06	80122
POGZ	0.222095	3.78E-06	23126
TRIM52	0.222061	3.80E-06	84851
MBLAC2	0.222032	3.81E-06	153364
YTHDF2	0.222	3.82E-06	51441
RIC8A	0.221904	3.86E-06	60626
ZNF75D	0.221867	3.87E-06	7626
NONO	0.221857	3.88E-06	4841
RGS5	0.221848	3.88E-06	8490
ZNF583	0.221822	3.89E-06	147949
MED28	0.221742	3.92E-06	80306
HES5	-0.22173	3.93E-06	388585
ZUFSP	0.22172	3.93E-06 NA	
ARHGEF40	0.221682	3.95E-06	55701
TMEM107	0.221647	3.96E-06	84314
RNF139	0.221639	3.96E-06	11236
POLR3C	0.221635	3.97E-06	10623
ERBB2IP	0.221614	3.97E-06 NA	
AGAP3	0.221507	4.02E-06	116988
CETN2	0.221416	4.06E-06	1069
CASP8	0.221405	4.06E-06	841
PMM2	0.221342	4.09E-06	5373
SPIN3	0.22129	4.11E-06	169981
TMEM218	0.221283	4.11E-06	219854
LIPT1	0.221252	4.12E-06	51601
LCORL	0.221249	4.13E-06	254251
MGAT5	0.221236	4.13E-06	4249
MTOR	0.221224	4.14E-06	2475
MYO1H	0.221221	4.14E-06	283446
MDM1	0.221175	4.16E-06	56890
RAB14	0.221158	4.16E-06	51552
CDH3	0.221138	4.17E-06	1001
WDR45B	0.221088	4.19E-06	56270
RRP1B	0.221063	4.21E-06	23076
SCAPER	0.221014	4.23E-06	49855
DDX20	0.221003	4.23E-06	11218
TNPO2	0.220969	4.25E-06	30000
ERMARD	0.220938	4.26E-06	55780
FRG1	0.220897	4.28E-06	2483
ADAMTS18	0.220881	4.28E-06	170692
ZNF234	0.220875	4.29E-06	10780
KIAA1377	0.220854	4.30E-06 NA	
LEMD3	0.220846	4.30E-06	23592
GIMAP1	0.220844	4.30E-06	170575
SEC61A1	0.220835	4.30E-06	29927
EDARADD	0.220829	4.31E-06	128178
CCDC132	0.220828	4.31E-06 NA	
GS1-114I9.3	0.220827	4.31E-06 NA	
ELP2	0.220797	4.32E-06	55250
PSMD11	0.220786	4.33E-06	5717

GPN3	0.22078	4.33E-06	51184
ZNF384	0.220776	4.33E-06	171017
CXCR2	0.220752	4.34E-06	3579
INO80	0.220752	4.34E-06	54617
IRF7	0.220743	4.35E-06	3665
EXOC7	0.220705	4.36E-06	23265
MAPK8IP1	0.2207	4.36E-06	9479
UNKL	0.220688	4.37E-06	64718
P2RY13	0.220686	4.37E-06	53829
ECT2	0.220674	4.38E-06	1894
OBSL1	0.220673	4.38E-06	23363
TCEAL1	0.220624	4.40E-06	9338
NRDE2	0.220621	4.40E-06	55051
PPP1R3B	0.220591	4.41E-06	79660
MTF1	0.220579	4.42E-06	4520
TMEM108	0.220563	4.43E-06	66000
IFT88	0.220555	4.43E-06	8100
LSM12	0.220548	4.43E-06	124801
SUPT16H	0.220536	4.44E-06	11198
SEC61A2	0.220517	4.45E-06	55176
RABL2B	0.220499	4.46E-06	11158
PRKCE	0.220476	4.47E-06	5581
CLTC	0.220419	4.49E-06	1213
ZFYVE9	0.220415	4.49E-06	9372
ANKH	0.220377	4.51E-06	56172
SNX14	0.220368	4.52E-06	57231
AC006116.20	0.220362	4.52E-06	NA
WIPI2	0.22032	4.54E-06	26100
RUFY1	0.220304	4.55E-06	80230
DNAJC2	0.220293	4.55E-06	27000
REXO2	0.220267	4.56E-06	25996
GRPEL2	0.22026	4.57E-06	134266
SIX4	0.220237	4.58E-06	51804
DCAF6	0.220236	4.58E-06	55827
CHTF8	0.220227	4.58E-06	54921
KIF5B	0.220197	4.60E-06	3799
MITD1	0.220191	4.60E-06	129531
GCA	0.220189	4.60E-06	25801
C15orf27	0.22018	4.60E-06	NA
NSUN6	0.220159	4.61E-06	221078
WRB	0.220131	4.63E-06	NA
TGIF2-C20orf24	0.220119	4.63E-06	NA
KIF1B	0.220077	4.65E-06	23095
LAMP1	0.220057	4.66E-06	3916
ATP9B	0.220044	4.67E-06	374868
NUPL2	0.220017	4.68E-06	NA
NSMCE2	0.220012	4.68E-06	286053
RSPH3	0.219977	4.70E-06	83861
TYW1	0.219916	4.73E-06	55253
TAS2R14	0.219874	4.75E-06	50840
CENPQ	0.219858	4.76E-06	55166
SNX6	0.219825	4.77E-06	58533
NAA35	0.219795	4.79E-06	60560
TECTB	0.219769	4.80E-06	6975
RNF6	0.21971	4.83E-06	6049
CACHD1	0.219681	4.84E-06	57685
PCDHGA7	0.21965	4.86E-06	56108
SRGAP3	0.219646	4.86E-06	9901
ZNF3	0.219643	4.86E-06	7551
C22orf46	0.219621	4.87E-06	79640

MICA	0.219613	4.88E-06	100507436
RP11-295K3.1	0.219603	4.88E-06	NA
CNTF	0.219502	4.93E-06	1270
CYP3A43	0.219498	4.93E-06	64816
SOX18	0.219479	4.94E-06	54345
RAB38	0.219469	4.95E-06	23682
CD74	0.219452	4.96E-06	972
LRRC63	0.219425	4.97E-06	220416
TRIM29	0.219411	4.98E-06	23650
SQSTM1	0.219388	4.99E-06	8878
PLA2G4E	0.219386	4.99E-06	123745
SLC35B2	0.219377	5.00E-06	347734
DENND4C	0.21937	5.00E-06	55667
ARNTL	0.219368	5.00E-06	406
TFAM	0.21935	5.01E-06	7019
PROSER2	0.219324	5.02E-06	254427
CA11	0.219316	5.03E-06	770
PSMC6	0.219268	5.05E-06	5706
NAPB	0.219213	5.08E-06	63908
PRKAG1	0.219179	5.10E-06	5571
MESDC2	0.21915	5.11E-06	NA
ANKRD35	0.219115	5.13E-06	148741
MGARP	0.219112	5.13E-06	84709
C12orf65	0.21911	5.13E-06	91574
ZNF346	0.219081	5.15E-06	23567
ZNF629	0.218996	5.19E-06	23361
TBC1D31	0.218976	5.20E-06	93594
TCEAL8	0.21897	5.21E-06	90843
TMEM214	0.218945	5.22E-06	54867
ASB1	0.218921	5.23E-06	51665
TCEAL7	0.21892	5.23E-06	56849
WDR5B	0.21891	5.24E-06	54554
MED26	0.218893	5.25E-06	9441
AK8	0.218872	5.26E-06	158067
CCL17	0.218866	5.26E-06	6361
MYLIP	0.218851	5.27E-06	29116
CKB	-0.21882	5.29E-06	1152
CWF19L1	0.218766	5.32E-06	55280
ELK1	0.218756	5.32E-06	2002
POLG2	0.218752	5.32E-06	11232
NUFIP1	0.218715	5.34E-06	26747
FPGT	0.218663	5.37E-06	8790
SLFN14	0.218663	5.37E-06	342618
ODF2	0.218644	5.38E-06	4957
DAAM2	0.218624	5.39E-06	23500
E2F5	0.218583	5.41E-06	1875
GCN1L1	0.21857	5.42E-06	NA
FLCN	0.218524	5.45E-06	201163
CFAP43	0.218524	5.45E-06	80217
STRAP	0.21846	5.48E-06	11171
GNB1	0.218445	5.49E-06	2782
SLMAP	0.218429	5.50E-06	7871
KRBA2	0.218331	5.55E-06	124751
NPTN	0.218319	5.56E-06	27020
C21orf62	0.218309	5.57E-06	56245
ZNF606	0.218303	5.57E-06	80095
LY6E	0.218296	5.57E-06	4061
YOD1	0.218288	5.58E-06	55432
POLR3F	0.218252	5.60E-06	10621
NAPG	0.218233	5.61E-06	8774

AZIN2	0.218213	5.62E-06	113451
NUP93	0.21819	5.63E-06	9688
RPRD2	0.218188	5.64E-06	23248
DIABLO	0.21818	5.64E-06	56616
MOGAT1	0.218133	5.67E-06	116255
HEPHL1	0.218127	5.67E-06	341208
KCNMB3	0.218084	5.70E-06	27094
PPP1CB	0.218064	5.71E-06	5500
ALKBH5	0.21805	5.71E-06	54890
ZC3H12B	0.218028	5.73E-06	340554
ACVR2B	0.218019	5.73E-06	93
NCF1	0.218017	5.73E-06	653361
FBXO45	0.218016	5.73E-06	200933
C22orf23	0.218014	5.74E-06	84645
NUP205	0.217998	5.74E-06	23165
KANSL2	0.217982	5.75E-06	54934
TRAF5	0.217964	5.76E-06	7188
FAM217A	0.217943	5.78E-06	222826
LACE1	0.217901	5.80E-06	NA
ZNF142	0.217794	5.86E-06	7701
AIG1	0.217762	5.88E-06	51390
KCNC3	0.217754	5.89E-06	3748
DIAPH2	0.217753	5.89E-06	1730
RPGRIP1	0.217752	5.89E-06	57096
RGS10	0.217743	5.89E-06	6001
RAC2	0.217738	5.90E-06	5880
ANKRD61	0.21773	5.90E-06	100310846
FBXO27	0.217724	5.91E-06	126433
KLHL9	0.217715	5.91E-06	55958
YKT6	0.217676	5.93E-06	10652
HYOU1	0.217665	5.94E-06	10525
CADM1	0.217664	5.94E-06	23705
PNO1	0.217664	5.94E-06	56902
SLC35E2	0.217662	5.94E-06	NA
PPP1R3C	0.217649	5.95E-06	5507
PISD	0.217641	5.96E-06	23761
PIGA	0.21764	5.96E-06	5277
AMD1	0.217631	5.96E-06	262
C16orf46	0.217627	5.96E-06	123775
ZNF577	0.217611	5.97E-06	84765
HDLBP	0.217582	5.99E-06	3069
PRKRA	0.217545	6.01E-06	8575
ANKHD1-EIF4EBP3	0.217522	6.03E-06	404734
COMMD9	0.217488	6.05E-06	29099
TAS2R13	0.217482	6.05E-06	50838
NUP54	0.21744	6.08E-06	53371
SF3B3	0.217428	6.08E-06	23450
ZPR1	0.2174	6.10E-06	8882
LINS	0.217399	6.10E-06	NA
VPS72	0.217396	6.10E-06	6944
TCERG1	0.217347	6.13E-06	10915
6-3月	0.217346	6.14E-06	NA
PDE3B	0.217321	6.15E-06	5140
EHBP1L1	0.217282	6.17E-06	254102
NOMO2	0.217279	6.18E-06	283820
PRKACA	0.217278	6.18E-06	5566
CCNA1	0.217276	6.18E-06	8900
CLP1	0.217275	6.18E-06	10978
KLHL10	0.217266	6.18E-06	317719
CCDC178	0.217246	6.20E-06	374864

C9orf85	0.217241	6.20E-06	138241
RPRD1B	0.217233	6.21E-06	58490
OPHN1	0.217217	6.22E-06	4983
CAPRIN2	0.217195	6.23E-06	65981
CDIPT	0.217175	6.24E-06	10423
SLC26A11	0.217172	6.24E-06	284129
SWI5	0.21713	6.27E-06	375757
PPFIA4	0.217099	6.29E-06	8497
ATP11B	0.217088	6.30E-06	23200
TARDBP	0.217076	6.30E-06	23435
VAMP1	0.217033	6.33E-06	6843
RIOK2	0.217019	6.34E-06	55781
CERS2	0.217018	6.34E-06	29956
CD2	0.217012	6.34E-06	914
HEXB	0.216975	6.37E-06	3074
ARSJ	0.216971	6.37E-06	79642
CASP2	0.216962	6.38E-06	835
UPK3B	0.216961	6.38E-06	105375355
EPB41L1	0.216941	6.39E-06	2036
ELMOD3	0.216916	6.41E-06	84173
ZNF202	0.216914	6.41E-06	7753
MPPED2	0.216899	6.42E-06	744
WBP11	0.216892	6.42E-06	51729
DCAF12	0.216881	6.43E-06	25853
CWC25	0.216877	6.43E-06	54883
SCOC	0.216859	6.44E-06	60592
KDM3B	0.216853	6.45E-06	51780
NBPF11	0.216824	6.47E-06	200030
IREB2	0.216814	6.47E-06	3658
SRP68	0.216798	6.48E-06	6730
LRRD1	0.216758	6.51E-06	401387
PKN2	0.216755	6.51E-06	5586
SKIV2L2	0.21675	6.51E-06	NA
MEGF8	0.216742	6.52E-06	1954
TTLL7	0.216701	6.55E-06	79739
ANKRD29	0.216674	6.56E-06	147463
GRIP1	0.216651	6.58E-06	23426
KIAA1147	0.216633	6.59E-06	NA
CLCC1	0.216625	6.60E-06	23155
MFAP1	0.2166	6.61E-06	4236
GOLGB1	0.216595	6.62E-06	2804
ZNF473	0.216589	6.62E-06	25888
RBM22	0.216572	6.63E-06	55696
TOP1	0.216565	6.64E-06	7150
NCOA6	0.216544	6.65E-06	23054
MOB4	0.216432	6.73E-06	25843
SPIN4	0.216426	6.73E-06	139886
DBT	0.216422	6.73E-06	1629
RPF2	0.216405	6.74E-06	84154
POLR3G	0.216402	6.75E-06	10622
VPS11	0.216379	6.76E-06	55823
UBE2I	0.216374	6.76E-06	7329
SMARCC2	0.216364	6.77E-06	6601
AMZ2	0.216255	6.85E-06	51321
CHERP	0.21623	6.86E-06	10523
KCTD2	0.21622	6.87E-06	23510
AASDH	0.216218	6.87E-06	132949
PPP6R1	0.216205	6.88E-06	22870
GDF7	0.216181	6.90E-06	151449
ZNF284	0.216177	6.90E-06	342909

BRD7	0.216171	6.90E-06	29117
TAP2	0.216166	6.91E-06	6891
ARHGEF2	0.216163	6.91E-06	9181
MORN4	0.216161	6.91E-06	118812
CD97	0.216112	6.94E-06	NA
DHX16	0.216101	6.95E-06	8449
TMC3	0.216093	6.96E-06	342125
PRF1	0.216035	7.00E-06	5551
TPCN2	0.216035	7.00E-06	219931
ATAD2	0.216018	7.01E-06	29028
TIPRL	0.216002	7.02E-06	261726
SASH3	0.215997	7.03E-06	54440
CDK15	0.215983	7.04E-06	65061
BTG3	0.215981	7.04E-06	10950
USP9X	0.215962	7.05E-06	8239
KANK1	0.215935	7.07E-06	23189
DDX31	0.215928	7.07E-06	64794
RNF103-CHMP3	0.21592	7.08E-06	100526767
KDELR3	0.215848	7.13E-06	11015
STK38L	0.215818	7.15E-06	23012
MAPRE1	0.215755	7.20E-06	22919
HERC2	0.215744	7.21E-06	8924
ZNF273	0.215718	7.22E-06	10793
SNAPC5	0.215702	7.24E-06	10302
CMTM2	0.215693	7.24E-06	146225
SMIM7	0.215671	7.26E-06	79086
CPOX	0.215629	7.29E-06	1371
KIF14	0.215623	7.29E-06	9928
ST3GAL5	0.2156	7.31E-06	8869
TCAIM	0.215592	7.32E-06	285343
WDSUB1	0.215589	7.32E-06	151525
ZNF217	0.215564	7.34E-06	7764
AC003002.6	0.215533	7.36E-06	NA
SUMO1	0.215492	7.39E-06	7341
SYPL2	0.215489	7.39E-06	284612
CBX4	0.215463	7.41E-06	8535
ZNF300	0.215463	7.41E-06	91975
DNASE1L1	0.21546	7.41E-06	1774
VPS33A	0.215453	7.42E-06	65082
KANK4	0.215358	7.49E-06	163782
PTPRJ	0.215334	7.51E-06	5795
FBXO32	0.215328	7.51E-06	114907
ANXA3	0.215327	7.51E-06	306
METTL4	0.215317	7.52E-06	64863
STAU1	0.215298	7.53E-06	6780
RP11-468E2.4	0.215246	7.57E-06	NA
SLC12A2	0.215242	7.58E-06	6558
TXNDC9	0.215227	7.59E-06	10190
CSDE1	0.215222	7.59E-06	7812
PCDHB3	0.215215	7.60E-06	56132
TBP	0.215212	7.60E-06	6908
SLCO1B1	0.2152	7.61E-06	10599
ZNF383	0.21517	7.63E-06	163087
GPBAR1	0.215156	7.64E-06	151306
SKAP2	0.215119	7.67E-06	8935
ANAPC4	0.215117	7.67E-06	29945
ITFG1	0.215117	7.67E-06	81533
PTPN2	0.215116	7.67E-06	5771
PSMD1	0.2151	7.68E-06	5707
ZNF26	0.215043	7.73E-06	7574

MED4	0.215043	7.73E-06	29079
RSL1D1	0.214961	7.79E-06	26156
TRIM34	0.214916	7.83E-06	53840
ZMYND8	0.214907	7.83E-06	23613
PDE6D	0.214883	7.85E-06	5147
FTSJ2	0.214879	7.85E-06 NA	
TMEM181	0.214866	7.86E-06	57583
BIN2	0.214852	7.88E-06	51411
MUC16	0.214828	7.89E-06	94025
PMP22	0.214818	7.90E-06	5376
SENP2	0.214807	7.91E-06	59343
ZNF200	0.214791	7.92E-06	7752
CCDC168	0.214763	7.95E-06	643677
NR2E3	0.214731	7.97E-06	10002
PDE10A	0.214683	8.01E-06	10846
RAB35	0.214665	8.02E-06	11021
AKAP8	0.214632	8.05E-06	10270
IL9R	0.214595	8.08E-06	3581
PDE4A	0.214585	8.09E-06	5141
BRSK1	0.214558	8.11E-06	84446
NAMPT	0.214527	8.13E-06	10135
LIN7C	0.214516	8.14E-06	55327
USP1	0.2145	8.16E-06	7398
6-9月	0.214484	8.17E-06 NA	
SFR1	0.214471	8.18E-06	119392
LAMP3	0.214448	8.20E-06	27074
PSG9	0.214448	8.20E-06	5678
DTD2	0.214439	8.21E-06	112487
USPL1	0.214411	8.23E-06	10208
RBMX	0.214398	8.24E-06	27316
TAF13	0.214367	8.26E-06	6884
MSH3	0.214351	8.28E-06	4437
CDK5RAP2	0.214351	8.28E-06	55755
PCDHA9	0.214328	8.30E-06	9752
UTP14C	0.214254	8.36E-06	9724
ATP6V0C	0.214249	8.36E-06	527
CAD	0.214234	8.37E-06	790
IKBKB	0.214233	8.38E-06	3551
SRD5A1	0.214203	8.40E-06	6715
PIM2	0.214195	8.41E-06	11040
DIO1	0.214194	8.41E-06	1733
HAP1	0.214107	8.48E-06	9001
LRRC37A2	0.214089	8.50E-06	474170
PROX2	0.214055	8.52E-06	283571
FYCO1	0.213986	8.58E-06	79443
PGS1	0.213978	8.59E-06	9489
PDCD2	0.213913	8.65E-06	5134
C1orf74	0.213891	8.66E-06	148304
IPPK	0.213866	8.69E-06	64768
RAB40AL	0.213863	8.69E-06	282808
MRPL42	0.213843	8.71E-06	28977
CCDC138	0.213821	8.72E-06	165055
PDPR	0.213756	8.78E-06	55066
ZSCAN5A	0.213715	8.82E-06	79149
NCBP2	0.21358	8.94E-06	22916
CCNH	0.213578	8.94E-06	902
RWDD2A	0.213564	8.95E-06	112611
NHS	0.21353	8.98E-06	4810
STPG2	0.213515	8.99E-06	285555
THAP9	0.213485	9.02E-06	79725

TOR1AIP2	0.213475	9.03E-06	163590
PGAM1	0.213457	9.05E-06	5223
IGFLR1	0.213427	9.07E-06	79713
AC092881.1	0.213412	9.08E-06	NA
NEK6	0.213402	9.09E-06	10783
ZRANB3	0.213397	9.10E-06	84083
C6orf165	0.21339	9.11E-06	NA
DERL2	0.213372	9.12E-06	51009
TSPAN16	0.21337	9.12E-06	26526
ZNF84	0.213353	9.14E-06	7637
CTD-2616J11.11	0.213302	9.18E-06	NA
UTP14A	0.213301	9.19E-06	10813
FANCL	0.21329	9.20E-06	55120
SSC4D	0.213179	9.30E-06	136853
OARD1	0.213146	9.33E-06	221443
PM20D2	0.213122	9.35E-06	135293
EIF4G1	0.213114	9.36E-06	1981
HMGXB3	0.213101	9.37E-06	22993
ZDHHC5	0.213097	9.37E-06	25921
RASAL3	0.213092	9.38E-06	64926
NOP9	0.213081	9.39E-06	161424
ABCC4	0.21307	9.40E-06	10257
PTMA	0.213063	9.40E-06	5757
LBR	0.213044	9.42E-06	3930
HTRA2	0.213041	9.42E-06	27429
NME6	0.213037	9.43E-06	10201
DPH6	0.213033	9.43E-06	89978
CPEB2	0.213033	9.43E-06	132864
HNRNPL	0.213	9.46E-06	3191
MIOS	0.212999	9.46E-06	54468
KRBOX4	0.212998	9.46E-06	55634
LIF	0.21299	9.47E-06	3976
EIF4H	0.212987	9.47E-06	7458
PRPF18	0.212983	9.48E-06	8559
DDX23	0.212979	9.48E-06	9416
AKIP1	0.212933	9.53E-06	56672
ERN1	0.212903	9.55E-06	2081
GSTCD	0.212896	9.56E-06	79807
AP000721.4	-0.21279	9.66E-06	NA
CLPX	0.212784	9.67E-06	10845
KDM4C	0.212759	9.69E-06	23081
ITGB3BP	0.212719	9.73E-06	23421
ZNF418	0.212704	9.74E-06	147686
SYNRG	0.212653	9.79E-06	11276
TRIM65	0.212641	9.80E-06	201292
IL12RB1	0.212625	9.82E-06	3594
CXCL10	0.212618	9.83E-06	3627
ZNF630	0.212617	9.83E-06	57232
OPTN	0.212612	9.83E-06	10133
PIK3R1	0.212604	9.84E-06	5295
FLYWCH1	0.212587	9.86E-06	84256
PCDHB15	0.212515	9.93E-06	56121
CD37	0.212479	9.96E-06	951
MPZL3	0.212467	9.97E-06	196264
SUCO	0.212392	1.00E-05	51430
CRAMP1L	0.212391	1.00E-05	NA
DYX1C1	0.212364	1.01E-05	NA
C20orf144	0.212349	1.01E-05	128864
RBM5	0.212347	1.01E-05	10181
RBMX2	0.212305	1.01E-05	51634

AUTS2	0.212294	1.01E-05	26053
TMEM14A	0.21229	1.01E-05	28978
CENPBD1	0.212281	1.02E-05	92806
SMARCD1	0.212273	1.02E-05	6602
CEP95	0.21227	1.02E-05	90799
EMB	0.21226	1.02E-05	133418
PATZ1	0.212259	1.02E-05	23598
SLC38A1	0.212252	1.02E-05	81539
TCEB3	0.212219	1.02E-05	NA
DNAH14	0.212182	1.03E-05	127602
ROCK2	0.212167	1.03E-05	9475
TXNRD1	0.212138	1.03E-05	7296
ARL14EPL	0.212119	1.03E-05	644100
DYNLL1	0.212113	1.03E-05	8655
ZNF419	0.212097	1.03E-05	79744
ANKRD27	0.212095	1.03E-05	84079
TSKU	0.212085	1.04E-05	25987
DCAF16	0.21204	1.04E-05	54876
ZCCHC2	0.212002	1.04E-05	54877
DHX37	0.211999	1.04E-05	57647
POLR1E	0.211995	1.04E-05	64425
COA7	0.211982	1.05E-05	65260
EIF3J	0.211981	1.05E-05	8669
TDG	0.211979	1.05E-05	6996
ZC3H18	0.2119	1.05E-05	124245
OCM	0.211862	1.06E-05	654231
NANP	0.211845	1.06E-05	140838
PACRGL	0.211825	1.06E-05	133015
IARS	0.211804	1.06E-05	NA
RRN3	0.211785	1.07E-05	54700
POLE3	0.211774	1.07E-05	54107
GPR63	0.211746	1.07E-05	81491
LRBA	0.211744	1.07E-05	987
GART	0.211651	1.08E-05	2618
ANO2	0.211594	1.09E-05	57101
TMOD3	0.211589	1.09E-05	29766
CRBN	0.211585	1.09E-05	51185
MIEF1	0.211561	1.09E-05	54471
TM9SF3	0.211538	1.09E-05	56889
NUP35	0.211536	1.09E-05	129401
CRMP1	0.211535	1.09E-05	1400
ANKRD1	0.211525	1.09E-05	27063
DDX52	0.211504	1.10E-05	11056
ZNF516	0.211467	1.10E-05	9658
MAP1S	0.211438	1.10E-05	55201
ZCCHC4	0.211429	1.10E-05	29063
GALK2	0.211426	1.10E-05	2585
ISY1	0.211419	1.11E-05	57461
TMEM194A	0.211407	1.11E-05	NA
TMEM260	0.211369	1.11E-05	54916
GCNT7	0.211364	1.11E-05	140687
ITIH4	0.211354	1.11E-05	3700
EPN2	0.211353	1.11E-05	22905
NFE2L3	0.211328	1.12E-05	9603
ORC2	0.211326	1.12E-05	4999
PIGB	0.211311	1.12E-05	9488
HNRNPA3	0.211301	1.12E-05	220988
PJA2	0.211273	1.12E-05	9867
WFDC3	0.211271	1.12E-05	140686
PAF1	0.211251	1.12E-05	54623

PHF1	0.211217	1.13E-05	5252
LGR5	0.211202	1.13E-05	8549
APOL2	0.211179	1.13E-05	23780
GPR162	0.211157	1.13E-05	27239
RBM17	0.211152	1.13E-05	84991
RNF4	0.211148	1.14E-05	6047
PALM	0.211132	1.14E-05	5064
CXCR1	0.211131	1.14E-05	3577
MAGED1	0.211104	1.14E-05	9500
CPVL	0.210938	1.16E-05	54504
AMN1	0.210911	1.16E-05	196394
AGRN	0.210896	1.16E-05	375790
PPP2R4	0.210877	1.17E-05	NA
PTCRA	0.210837	1.17E-05	171558
TAOK2	0.21083	1.17E-05	9344
ASPRV1	0.210797	1.17E-05	151516
UTP15	0.210786	1.18E-05	84135
RGS18	0.210783	1.18E-05	64407
FOXQ1	0.210753	1.18E-05	94234
DAGLB	0.210739	1.18E-05	221955
FBXO18	0.210739	1.18E-05	NA
TAB3	0.210734	1.18E-05	257397
KIAA1549	0.210723	1.18E-05	57670
SNX12	0.210708	1.18E-05	29934
BSCL2	0.21066	1.19E-05	26580
LIN37	0.210633	1.19E-05	55957
PRR12	0.210531	1.21E-05	57479
ZNF487	0.210528	1.21E-05	642819
TFAP2C	0.210434	1.22E-05	7022
SYBU	0.210419	1.22E-05	55638
PAQR3	0.21036	1.23E-05	152559
CUEDC2	0.21036	1.23E-05	79004
TMEM130	0.210348	1.23E-05	222865
PIK3CB	0.210263	1.24E-05	5291
TNFRSF21	0.210262	1.24E-05	27242
SPEF2	0.210261	1.24E-05	79925
VMA21	0.210256	1.24E-05	203547
ZNF263	0.210214	1.24E-05	10127
DFNB59	0.2102	1.25E-05	NA
ZNF835	0.210196	1.25E-05	90485
ADAMTS1	0.210187	1.25E-05	9510
CAPZA1	0.210186	1.25E-05	829
CAMKK2	0.210176	1.25E-05	10645
RAD17	0.210174	1.25E-05	5884
EPHA4	0.210147	1.25E-05	2043
ZNF780B	0.210104	1.26E-05	163131
PLA2R1	0.210092	1.26E-05	22925
SLAMF1	0.210085	1.26E-05	6504
PSPC1	0.210055	1.26E-05	55269
DROSHA	0.210049	1.26E-05	29102
CYP8B1	0.210049	1.26E-05	1582
FTSJ1	0.210047	1.26E-05	24140
IL31RA	0.210039	1.26E-05	133396
NPTXR	0.210034	1.27E-05	23467
CWC15	0.210002	1.27E-05	51503
AP3B1	0.210002	1.27E-05	8546
FAM83D	0.209999	1.27E-05	81610
PIGF	0.209977	1.27E-05	5281
ZNF224	0.20997	1.27E-05	7767
USB1	0.209955	1.28E-05	79650

NCOA1	0.209938	1.28E-05	8648
YIPF6	0.2099	1.28E-05	286451
NUDT17	0.209841	1.29E-05	200035
SETDB1	0.209834	1.29E-05	9869
NRF1	0.209828	1.29E-05	4899
ZNF727	0.209811	1.29E-05	442319
ABCB4	0.209792	1.30E-05	5244
SMG7	0.209775	1.30E-05	9887
RTF1	0.209744	1.30E-05	23168
AP4S1	0.20973	1.30E-05	11154
PPTC7	0.209705	1.31E-05	160760
SGK494	0.209695	1.31E-05	NA
PDE4D	0.209635	1.32E-05	5144
GALNT6	0.209629	1.32E-05	11226
DVL2	0.209625	1.32E-05	1856
SUMF2	0.209622	1.32E-05	25870
COL27A1	0.209622	1.32E-05	85301
CARS	0.209603	1.32E-05	NA
GPATCH1	0.209584	1.32E-05	55094
ARPC5	0.209573	1.32E-05	10092
INTS12	0.209567	1.32E-05	57117
IK	0.209566	1.32E-05	3550
EXO5	0.209565	1.32E-05	64789
KIAA0100	0.209559	1.33E-05	9703
SNCA	0.209556	1.33E-05	6622
PROCR	0.209552	1.33E-05	10544
PARP1	0.209548	1.33E-05	142
MAGT1	0.209533	1.33E-05	84061
CSNK1E	0.209499	1.33E-05	1454
ZNF584	0.209497	1.33E-05	201514
SPRY3	0.209495	1.33E-05	10251
ZFAND6	0.209485	1.33E-05	54469
ZNF776	0.209481	1.34E-05	284309
SYT2	0.209465	1.34E-05	127833
CFAP53	0.209457	1.34E-05	220136
KRBA1	0.209422	1.34E-05	84626
C6orf211	0.209406	1.35E-05	NA
STT3B	0.209368	1.35E-05	201595
KLHDC8A	0.209363	1.35E-05	55220
ALKBH1	0.209342	1.35E-05	8846
PRPF3	0.209296	1.36E-05	9129
NDNL2	0.209279	1.36E-05	NA
NEK10	0.20926	1.36E-05	152110
GCLM	0.209245	1.37E-05	2730
CDC14B	0.209239	1.37E-05	8555
RFWD3	0.209238	1.37E-05	55159
GDPD5	0.209233	1.37E-05	81544
SHANK2	0.209232	1.37E-05	22941
KIAA1107	0.209219	1.37E-05	NA
TAF15	0.209215	1.37E-05	8148
GATAD1	0.20921	1.37E-05	57798
RBBP9	0.209204	1.37E-05	10741
MICB	0.209145	1.38E-05	4277
RNF40	0.209124	1.38E-05	9810
TTC23L	0.209116	1.38E-05	153657
C17orf47	0.20911	1.38E-05	NA
MRPL50	0.209092	1.39E-05	54534
C6orf201	0.20908	1.39E-05	404220
EWSR1	0.209016	1.40E-05	2130
PPAT	0.209007	1.40E-05	5471

UBE2G1	0.208977	1.40E-05	7326
PCDHGA8	0.208954	1.41E-05	9708
ANKRD53	0.20892	1.41E-05	79998
GCLC	0.208848	1.42E-05	2729
TRMT112	0.208815	1.42E-05	51504
EIF3H	0.208774	1.43E-05	8667
PLEKHN1	0.208751	1.43E-05	84069
PCDHGA2	0.208732	1.44E-05	56113
ANKRD18A	0.208704	1.44E-05	253650
IP6K1	0.20869	1.44E-05	9807
CLDN20	0.208626	1.45E-05	49861
ACIN1	0.208611	1.45E-05	22985
NIFK	0.208605	1.45E-05	84365
FAM72D	0.208567	1.46E-05	728833
CSTF1	0.208562	1.46E-05	1477
ANKRD46	0.208556	1.46E-05	157567
ADCY2	0.208531	1.46E-05	108
ANKS1A	0.208515	1.47E-05	23294
WDHD1	0.208489	1.47E-05	11169
PBXIP1	0.208463	1.47E-05	57326
FHOD3	0.208443	1.48E-05	80206
CYP21A2	0.208418	1.48E-05	1589
CD1B	0.208417	1.48E-05	910
FUNDC2	0.208413	1.48E-05	65991
LRRC20	0.208401	1.48E-05	55222
WDR12	0.208366	1.49E-05	55759
ARL6IP1	0.208334	1.49E-05	23204
TRAK2	0.208332	1.49E-05	66008
SLC37A3	0.208329	1.49E-05	84255
TMEM242	0.208234	1.51E-05	729515
SLC47A1	0.208224	1.51E-05	55244
MMP3	0.208168	1.52E-05	4314
WASF2	0.208156	1.52E-05	10163
LRRC39	0.208154	1.52E-05	127495
CNOT8	0.208154	1.52E-05	9337
ANGEL1	0.208137	1.52E-05	23357
IL17RD	0.208122	1.52E-05	54756
SNX8	0.208101	1.53E-05	29886
SLC4A5	0.208095	1.53E-05	57835
SPAG8	0.208024	1.54E-05	26206
PRR4	0.207969	1.55E-05	11272
RP11-613M10.8	0.2079	1.56E-05	NA
MAP3K9	0.20788	1.56E-05	4293
ZNF736	0.207837	1.57E-05	728927
GFOD1	0.207788	1.57E-05	54438
MNT	0.207753	1.58E-05	4335
PTDSS1	0.207747	1.58E-05	9791
HSD11B2	-0.20774	1.58E-05	3291
ZNF701	0.207718	1.58E-05	55762
TDRP	0.207708	1.58E-05	157695
ZNF195	0.207666	1.59E-05	7748
LEP	0.207656	1.59E-05	3952
CEP192	0.207643	1.59E-05	55125
FOXK1	0.20761	1.60E-05	221937
MAP3K13	0.207574	1.61E-05	9175
RCC2	0.207573	1.61E-05	55920
CHIC1	0.207548	1.61E-05	53344
RPTOR	0.207531	1.61E-05	57521
NRG3	0.207523	1.61E-05	10718
TSPYL6	0.207516	1.61E-05	388951

ARL1	0.207493	1.62E-05	400
RTEL1	0.207475	1.62E-05	51750
ZWILCH	0.207454	1.62E-05	55055
C16orf96	0.207452	1.62E-05	342346
UNK	0.207451	1.62E-05	85451
SLC22A15	0.207438	1.63E-05	55356
TEAD3	0.207433	1.63E-05	7005
PARP2	0.207383	1.64E-05	10038
F3	0.207346	1.64E-05	2152
UBE2L6	0.207337	1.64E-05	9246
LRIF1	0.2073	1.65E-05	55791
ATG13	0.207299	1.65E-05	9776
GPSM2	0.207267	1.65E-05	29899
ZNF441	0.207259	1.65E-05	126068
ZNHIT3	0.207243	1.66E-05	9326
DNAAF2	0.207217	1.66E-05	55172
FBXL17	0.207191	1.67E-05	64839
LARS	0.207176	1.67E-05	NA
ENPP2	0.207153	1.67E-05	5168
ANKFY1	0.207132	1.67E-05	51479
TMEM54	-0.20711	1.68E-05	113452
CCNL1	0.207108	1.68E-05	57018
DRG1	0.20709	1.68E-05	4733
RPA2	0.207062	1.69E-05	6118
GPR22	0.20704	1.69E-05	2845
EXD2	0.207018	1.69E-05	55218
VTCN1	0.207005	1.70E-05	79679
ZBTB10	0.207002	1.70E-05	65986
ADAMTSL2	0.206981	1.70E-05	9719
S100A13	0.206962	1.70E-05	6284
GOLPH3	0.206955	1.70E-05	64083
DCUN1D5	0.206952	1.70E-05	84259
HDAC8	0.206952	1.70E-05	55869
CLCN7	0.206946	1.71E-05	1186
TOMM70A	0.206945	1.71E-05	NA
DUSP18	0.206926	1.71E-05	150290
FNDC9	0.206916	1.71E-05	408263
EZH1	0.206836	1.72E-05	2145
METTL23	0.206827	1.72E-05	124512
GSPT1	0.206822	1.73E-05	2935
GPX1	0.206805	1.73E-05	2876
PRPS1	0.206802	1.73E-05	5631
TIMMDC1	0.206793	1.73E-05	51300
ZNF737	0.20678	1.73E-05	100129842
TCHP	0.206775	1.73E-05	84260
CTPS1	0.206748	1.74E-05	1503
MLLT1	0.206738	1.74E-05	4298
HLA-DRB5	0.20673	1.74E-05	3127
RANBP17	0.20673	1.74E-05	64901
RALB	0.206722	1.74E-05	5899
GPR89B	0.206717	1.74E-05	51463
FOCAD	0.206707	1.74E-05	54914
NKAP	0.206695	1.75E-05	79576
VPS13D	0.206675	1.75E-05	55187
COPS5	0.206617	1.76E-05	10987
CRTC2	0.20661	1.76E-05	200186
SBDS	0.206594	1.76E-05	51119
AGPAT1	0.206581	1.77E-05	10554
NUP107	0.206579	1.77E-05	57122
PHF8	0.206577	1.77E-05	23133

ETF1	0.206552	1.77E-05	2107
CUL1	0.20654	1.77E-05	8454
MOB3C	0.20653	1.77E-05	148932
MCPH1	0.20653	1.77E-05	79648
TTC23	0.206429	1.79E-05	64927
SETD1A	0.206413	1.79E-05	9739
HDAC4	0.206409	1.79E-05	9759
C19orf68	0.206343	1.81E-05	NA
DUSP1	0.206337	1.81E-05	1843
GOLGA8H	0.206317	1.81E-05	728498
EGFR	0.206289	1.82E-05	1956
PIAS2	0.206281	1.82E-05	9063
TSPAN18	0.206281	1.82E-05	90139
CYP24A1	0.20627	1.82E-05	1591
DEF6	0.20623	1.83E-05	50619
FGD1	0.206151	1.84E-05	2245
ST6GAL1	0.206017	1.86E-05	6480
TRMT6	0.20599	1.87E-05	51605
GMCL1	0.205981	1.87E-05	64395
POLR3H	0.205975	1.87E-05	171568
R3HDM2	0.205956	1.87E-05	22864
MYEOV	0.205935	1.88E-05	26579
OLFML2A	0.205931	1.88E-05	169611
HILPDA	0.205917	1.88E-05	29923
ENO2	0.205916	1.88E-05	2026
SCYL3	0.205916	1.88E-05	57147
PRRC2A	0.205911	1.88E-05	7916
AC092835.2	0.205901	1.88E-05	NA
RP11-446E24.4	0.205889	1.89E-05	NA
IFIT2	0.205882	1.89E-05	3433
CRY2	0.20584	1.90E-05	1408
CSNK1G3	0.205826	1.90E-05	1456
BBOX1	0.2058	1.90E-05	8424
FAM72A	0.205789	1.90E-05	729533
NR2C1	0.205782	1.91E-05	7181
MRPL33	0.20577	1.91E-05	9553
PYGL	0.205758	1.91E-05	5836
EBF4	0.20575	1.91E-05	57593
ARIH2	0.205743	1.91E-05	10425
ATXN7L3B	0.205737	1.91E-05	552889
RP11-437B10.1	0.20572	1.92E-05	NA
UBXN1	0.205713	1.92E-05	51035
KARS	0.205692	1.92E-05	NA
DDX27	0.205676	1.93E-05	55661
LUC7L3	0.205625	1.93E-05	51747
PLEC	0.205608	1.94E-05	5339
SUV420H1	0.205581	1.94E-05	NA
INTS7	0.205565	1.95E-05	25896
BIVM	0.20555	1.95E-05	54841
SNX33	0.205545	1.95E-05	257364
HSD3B7	0.205515	1.95E-05	80270
ZNF773	0.205462	1.96E-05	374928
TSC1	0.205461	1.96E-05	7248
GRWD1	0.205452	1.97E-05	83743
SRSF4	0.205415	1.97E-05	6429
SDC1	0.205394	1.98E-05	6382
CHMP3	0.205382	1.98E-05	51652
SLX4IP	0.205374	1.98E-05	128710
RP11-864I4.1	0.205359	1.98E-05	NA
KXD1	0.205345	1.99E-05	79036

SF1	0.205344	1.99E-05	7536
STC2	0.205322	1.99E-05	8614
ANKRD22	0.205312	1.99E-05	118932
RRH	0.205294	2.00E-05	10692
ISCU	0.205294	2.00E-05	23479
BARD1	0.20527	2.00E-05	580
TBC1D12	0.205256	2.00E-05	23232
ZDHHC8	0.2052	2.01E-05	29801
RAD50	0.205133	2.03E-05	10111
GPALPP1	0.205076	2.04E-05	55425
GLA	0.205066	2.04E-05	2717
PHLDB3	0.205052	2.04E-05	653583
PTPN6	0.205021	2.05E-05	5777
KLF10	0.204992	2.05E-05	7071
C15orf52	0.204922	2.07E-05	NA
WEE1	0.20492	2.07E-05	7465
PARP4	0.204905	2.07E-05	143
AFTPH	0.204802	2.09E-05	54812
CAMP	0.20477	2.10E-05	820
ACTL6A	0.204764	2.10E-05	86
BACH2	0.204757	2.10E-05	60468
IL13RA1	0.204721	2.11E-05	3597
IPO13	0.20471	2.11E-05	9670
ZNF558	0.204703	2.11E-05	148156
FAM159A	0.204702	2.11E-05	NA
GZF1	0.204696	2.11E-05	64412
LSG1	0.20468	2.12E-05	55341
CKAP5	0.204673	2.12E-05	9793
PRX	0.204635	2.13E-05	57716
ODF3B	0.204609	2.13E-05	440836
PTP4A2	0.204569	2.14E-05	8073
CRYAB	0.204564	2.14E-05	1410
ZBTB12	0.20455	2.14E-05	221527
TMEM209	0.204547	2.14E-05	84928
KIAA0895	0.204534	2.15E-05	23366
SYS1	0.204486	2.16E-05	90196
TEC	0.204406	2.17E-05	7006
PIEZO1	0.204396	2.17E-05	9780
ENHO	-0.20438	2.18E-05	375704
ANKRD13D	0.204368	2.18E-05	338692
BEX4	0.20434	2.19E-05	56271
CDC42BPB	0.204334	2.19E-05	9578
CYP11A1	0.204323	2.19E-05	1583
LMO7	0.204321	2.19E-05	4008
EI24	0.204309	2.19E-05	9538
TMEM39B	0.20428	2.20E-05	55116
GOLGA7B	0.204258	2.20E-05	401647
SNRNP200	0.204245	2.21E-05	23020
PARVB	0.204201	2.21E-05	29780
ACP2	0.204183	2.22E-05	53
LAP3	0.204176	2.22E-05	51056
PDIA5	0.204157	2.22E-05	10954
UAP1L1	0.204123	2.23E-05	91373
RNFT1	0.204122	2.23E-05	51136
PDPK1	0.204106	2.23E-05	5170
ATP6AP1	0.204027	2.25E-05	537
COIL	0.204014	2.25E-05	8161
TNF	0.203998	2.26E-05	7124
KLF9	0.203967	2.26E-05	687
AL138706.2	0.203936	2.27E-05	NA

C8orf44	0.203925	2.27E-05	56260
CCDC38	0.203878	2.28E-05	120935
C5orf49	0.203867	2.29E-05	134121
GNL3	0.203861	2.29E-05	26354
TSEN15	0.203842	2.29E-05	116461
FBXO48	0.203813	2.30E-05	554251
INPP5D	0.203753	2.31E-05	3635
TAF11	0.203724	2.32E-05	6882
HNRNPA0	0.203705	2.32E-05	10949
CTNNBL1	0.203683	2.33E-05	56259
CSNK2A2	0.203671	2.33E-05	1459
NPEPPS	0.203666	2.33E-05	9520
ZNF7	0.203664	2.33E-05	7553
EPRS	0.203651	2.33E-05	NA
AP2A1	0.20363	2.34E-05	160
KLRK1	0.203588	2.35E-05	22914
TXNRD3	0.203584	2.35E-05	114112
TOPBP1	0.203546	2.36E-05	11073
CAP1	0.203475	2.37E-05	10487
DDX1	0.203464	2.37E-05	1653
SMYD4	0.203433	2.38E-05	114826
MLLT4	0.20337	2.40E-05	NA
ZMIZ2	0.203368	2.40E-05	83637
COX7A1	0.203326	2.41E-05	1346
LYPLA1	0.203299	2.41E-05	10434
MADD	0.203279	2.42E-05	8567
DCK	0.203246	2.42E-05	1633
CDK2	0.203205	2.43E-05	1017
DKK1	0.203182	2.44E-05	22943
CHTOP	0.203161	2.44E-05	26097
DPH3	0.20315	2.45E-05	285381
TMEM232	0.203146	2.45E-05	642987
FAM124A	0.203093	2.46E-05	220108
CROCC	0.203073	2.46E-05	9696
RARS	0.203028	2.47E-05	NA
AIM2	0.203026	2.47E-05	9447
SYTL4	0.203015	2.48E-05	94121
EPB41L5	0.203006	2.48E-05	57669
SLC35F4	0.202974	2.49E-05	341880
MED22	0.202971	2.49E-05	6837
TYW1B	0.202944	2.49E-05	441250
PARP6	0.202936	2.50E-05	56965
DEXI	0.202918	2.50E-05	28955
AREL1	0.202902	2.50E-05	9870
FBXO22	0.202865	2.51E-05	26263
ZC3HAV1L	0.202833	2.52E-05	92092
NUP155	0.202833	2.52E-05	9631
PREB	0.2027	2.55E-05	10113
CNPY2	0.202665	2.56E-05	10330
TBC1D4	0.20266	2.56E-05	9882
PGAP1	0.202637	2.57E-05	80055
CCL28	-0.20264	2.57E-05	56477
FASTKD2	0.202633	2.57E-05	22868
SLC39A7	0.202632	2.57E-05	7922
ARL6IP6	0.202632	2.57E-05	151188
NBEAL1	0.202624	2.57E-05	65065
MTHFSD	0.202574	2.58E-05	64779
ABHD16A	0.202571	2.58E-05	7920
CLIP3	0.20255	2.59E-05	25999
GPR82	0.202498	2.60E-05	27197

FAM216A	0.202477	2.60E-05	29902
METTL20	0.202449	2.61E-05	NA
L3MBTL2	0.202446	2.61E-05	83746
EIF2B1	0.202429	2.62E-05	1967
ZNF467	0.202414	2.62E-05	168544
ACSL3	0.202379	2.63E-05	2181
HTATSF1	0.202375	2.63E-05	27336
ARID1A	0.202369	2.63E-05	8289
BCL2L13	0.202356	2.63E-05	23786
SUGP2	0.202311	2.65E-05	10147
C16orf95	0.202285	2.65E-05	100506581
RANBP3	0.20226	2.66E-05	8498
FAM32A	0.202253	2.66E-05	26017
RTFDC1	0.202216	2.67E-05	NA
ZNF77	0.202211	2.67E-05	58492
AP2B1	0.202202	2.67E-05	163
TBL1X	0.202197	2.67E-05	6907
TM4SF18	0.202182	2.68E-05	116441
NKRF	0.202179	2.68E-05	55922
GTF3C2	0.202175	2.68E-05	2976
TAMM41	0.202172	2.68E-05	132001
U2AF1	0.202161	2.68E-05	7307
SLC2A10	0.202159	2.68E-05	81031
CLEC16A	0.202121	2.69E-05	23274
SMIM17	0.202109	2.70E-05	147670
PAXBP1	0.202108	2.70E-05	94104
WDR46	0.202101	2.70E-05	9277
NUP43	0.20209	2.70E-05	348995
PBDC1	0.202056	2.71E-05	51260
TSSK4	0.202015	2.72E-05	283629
AQR	0.202009	2.72E-05	9716
ZFP64	0.202001	2.72E-05	55734
MTHFD2L	0.20199	2.73E-05	441024
PSMA2	0.201983	2.73E-05	5683
PRR14L	0.201957	2.73E-05	253143
VTA1	0.201938	2.74E-05	51534
FBXO9	0.201927	2.74E-05	26268
ELP4	0.201877	2.76E-05	26610
PIGW	0.201869	2.76E-05	284098
ZNF691	0.201867	2.76E-05	51058
DNAJC7	0.201827	2.77E-05	7266
AOAH	0.201819	2.77E-05	313
TOX4	0.201774	2.78E-05	9878
PDIK1L	0.201765	2.78E-05	149420
MS4A2	0.201746	2.79E-05	2206
SCYL1	0.201719	2.80E-05	57410
ZNF707	0.201713	2.80E-05	286075
ORMDL1	0.201665	2.81E-05	94101
APOLD1	0.201645	2.82E-05	81575
PSMD10	0.20162	2.82E-05	5716
MEA1	0.201563	2.84E-05	4201
RAP1GDS1	0.201544	2.84E-05	5910
KCTD14	0.201514	2.85E-05	65987
RINT1	0.201503	2.85E-05	60561
GUF1	0.201491	2.86E-05	60558
EDEM2	0.201445	2.87E-05	55741
PPP1R3F	0.20144	2.87E-05	89801
MICAL3	0.201425	2.87E-05	57553
XKRX	0.201413	2.88E-05	402415
TIMM10B	0.201399	2.88E-05	26515

RALGPS2	0.201381	2.89E-05	55103
VPS37D	0.201371	2.89E-05	155382
NUP214	0.201359	2.89E-05	8021
CD3EAP	0.201352	2.89E-05	NA
ZNF655	0.20134	2.90E-05	79027
MFAP3L	0.201322	2.90E-05	9848
HSPA1L	0.2013	2.91E-05	3305
MAP6	0.201217	2.93E-05	4135
STX5	0.201186	2.94E-05	6811
ZDHHC16	0.201185	2.94E-05	84287
AVEN	0.201178	2.94E-05	57099
OXSR1	0.201159	2.95E-05	9943
DCLK1	0.201116	2.96E-05	9201
KIAA2018	0.2011	2.96E-05	NA
FAM19A3	0.201087	2.97E-05	NA
PLRG1	0.20104	2.98E-05	5356
SCFD2	0.201031	2.98E-05	152579
CREG2	0.200989	2.99E-05	200407
IL13	0.200955	3.00E-05	3596
RUFY4	0.200939	3.01E-05	285180
GNPTAB	0.200897	3.02E-05	79158
SLC30A1	0.200893	3.02E-05	7779
CDKN2AIP	0.200863	3.03E-05	55602
TCF7L1	0.200856	3.03E-05	83439
ABCD1	0.200799	3.05E-05	215
RSPH4A	0.200798	3.05E-05	345895
MDM2	0.20076	3.06E-05	4193
AL365181.2	0.200757	3.06E-05	NA
MAP1LC3B2	0.200732	3.07E-05	643246
RANBP2	0.200715	3.07E-05	5903
HAT1	0.200704	3.07E-05	8520
MID1	0.200686	3.08E-05	4281
NVL	0.20068	3.08E-05	4931
MAGOHB	0.200661	3.09E-05	55110
WNT7B	0.200641	3.09E-05	7477
CYB5D1	0.200613	3.10E-05	124637
YTHDF1	0.200609	3.10E-05	54915
GNRHR	0.200602	3.10E-05	2798
SNX20	0.200598	3.10E-05	124460
KLHL36	0.20056	3.12E-05	79786
BATF3	0.200559	3.12E-05	55509
ASH2L	0.20054	3.12E-05	9070
ZNF20	0.200512	3.13E-05	7568
BCCIP	0.200504	3.13E-05	56647
FAN1	0.200503	3.13E-05	22909
DIMT1	0.200492	3.14E-05	27292
NGDN	0.200485	3.14E-05	25983
ADORA2A	0.200459	3.14E-05	135
HNMT	0.200457	3.15E-05	3176
ITGB6	0.20039	3.16E-05	3694
RALGAPA1	0.200388	3.17E-05	253959
ANKRD52	0.200384	3.17E-05	283373
USP18	0.200356	3.17E-05	11274
SAP30BP	0.200355	3.18E-05	29115
TMEM230	0.200318	3.19E-05	29058
DUS4L	0.200287	3.20E-05	11062
MYH2	0.200255	3.20E-05	4620
C17orf62	0.200235	3.21E-05	NA
ARL13A	0.200231	3.21E-05	392509
FCRL5	0.20019	3.22E-05	83416

C7orf73	0.200186	3.23E-05	NA
TRIQK	0.200134	3.24E-05	286144
SERTM1	0.20013	3.24E-05	400120
RTTN	0.200125	3.24E-05	25914
TIGD2	0.20012	3.25E-05	166815
MMADHC	0.200115	3.25E-05	27249
WDR49	0.200112	3.25E-05	151790
SNX1	0.200091	3.25E-05	6642
YWHAB	0.200059	3.26E-05	7529
BAG5	0.200045	3.27E-05	9529
THPO	0.200021	3.28E-05	7066
